# Chemoselective palladium-catalyzed deprotonative arylation/[1,2]-Wittig rearrangement of pyridylmethyl ethers†
†Electronic supplementary information (ESI) available: Experimental details, full characterization of new compounds, and calculated structures and energies. See DOI: 10.1039/c5sc02739j


**DOI:** 10.1039/c5sc02739j

**Published:** 2015-10-27

**Authors:** Feng Gao, Byeong-Seon Kim, Patrick J. Walsh

**Affiliations:** a Department of Medicinal Plants , Agronomy College , Sichuan Agricultural University , 211, Huimin Rd, Wenjiang Region , Chengdu 611130 , PR China; b Roy and Diana Vagelos Laboratories , Penn/Merck Laboratory for High-Throughput Experimentation , Department of Chemistry , University of Pennsylvania , 231 S, 34th St. , Philadelphia , PA 19104-6323 , USA . Email: pwalsh@sas.upenn.edu ; https://sites.google.com/site/titaniumupenn/ ; Fax: +1-215-573-6743

## Abstract

Control of chemoselectivity is one of the most challenging problems facing chemists and is particularly important in the synthesis of bioactive compounds and medications. Herein, the first highly chemoselective tandem C(sp^3^)–H arylation/[1,2]-Wittig rearrangement of pyridylmethyl ethers is presented. The efficient and operationally simple protocols enable generation of either arylation products or tandem arylation/[1,2]-Wittig rearrangement products with remarkable selectivity and good to excellent yields (60–99%). Choice of base, solvent, and reaction temperature play a pivotal role in tuning the reactivity of intermediates and controlling the relative rates of competing processes. The novel arylation step is catalyzed by a Pd(OAc)_2_/NIXANTPHOS-based system *via* a deprotonative cross-coupling process. The method provides rapid access to skeletally diverse aryl(pyridyl)methanol core structures, which are central components of several medications.

## Abstract 2

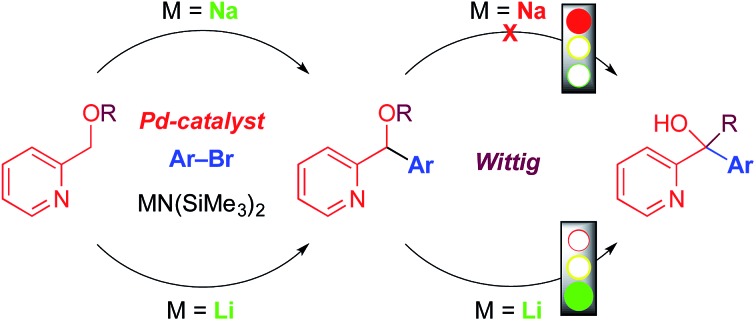
Control of chemoselectivity is one of the most challenging problems facing chemists and is particularly important in the synthesis of bioactive compounds and medications.

## 1. Introduction

The synthesis of diverse chemical structures is an important endeavor in the discovery of new medications. Forging structural diversity from a common set of organic substrates is extremely desirable in this venture, but is quite difficult to achieve with high selectivity.^1^ This is particularly challenging when tandem reactions are employed, wherein several chemical transformations are performed without isolation of intermediates, addition of new reagents, or modification of reaction parameters.

**Fig. 1:**
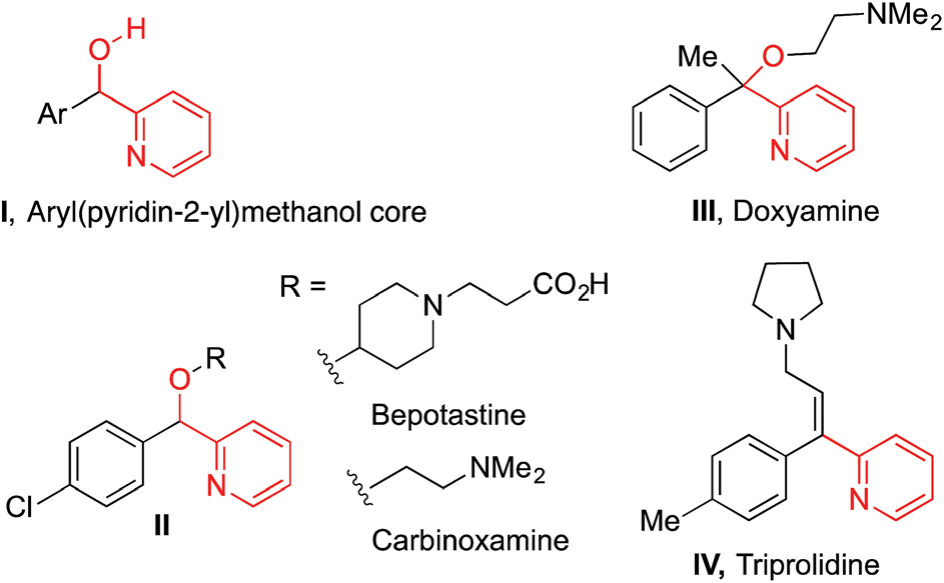
Structures of drugs derived from the aryl(pyridin-2-yl) core.

**Fig. 2:**
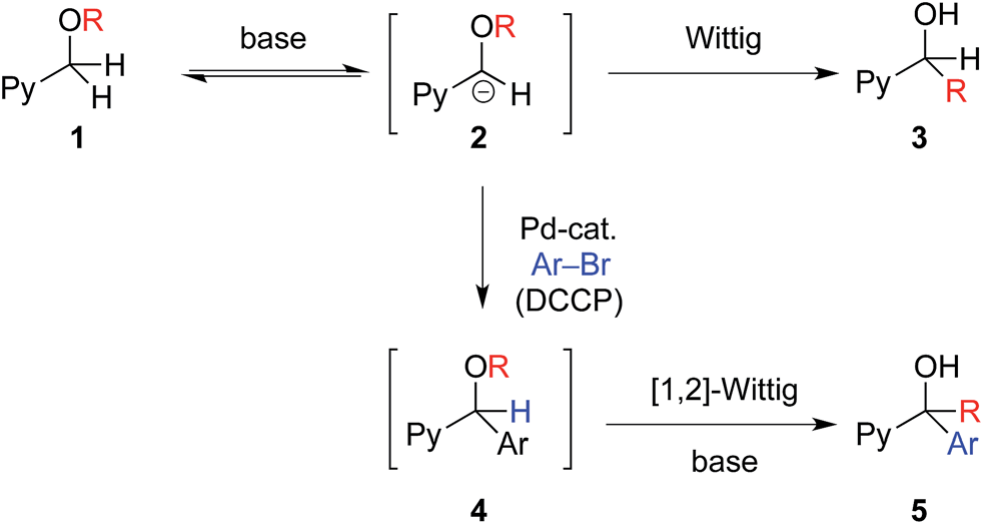
Chemoselective [1,2]-Wittig rearrangement to give **3**, arylation to give **4**, and tandem arylation/[1,2]-Wittig rearrangement to afford **5**.

In considering the synthesis of a family of drug-like molecules based on the aryl(pyridin-2-yl)methanol core (**I**, Fig. 1), we envisioned a unified approach to access these important structural motifs based on a non-traditional umpolung approach.^2^ Our strategy (Fig. 2) entailed a palladium-catalyzed deprotonative cross-coupling process (DCCP) with pyridylmethyl ethers **1** and aryl bromides to generate arylated secondary ethers **4** while the tertiary alcohols **5** would be accessed *via* a tandem DCCP/[1,2]-Wittig rearrangement.

Upon examination of the reactions in Fig. 2, several challenges are evident. Reversible deprotonation of ether **1** generates the intermediate carbanion **2**.^3^ α-Alkoxy carbanions are known to undergo [1,2]-Wittig rearrangements to generate alcohols **3**.^4^ If anion **2** could be intercepted by a palladium catalyst *via* rapid transmetallation,^5^ arylation may outcompete the [1,2]-Wittig rearrangement of **2** to generate the desired aryl(pyridin-2-yl)methyl ether core, **4**. Compound **4**, possessing a more acidic benzylic hydrogen than **2**, may undergo deprotonation under the basic reaction conditions followed by [1,2]-Wittig rearrangement to form tertiary alcohols **5**. Because structures **4** and **5** are both valuable intermediates to furnish bioactive molecules and commercialized drugs (Fig. 1), conditions that inhibit and facilitate the tandem [1,2]-Wittig rearrangement of intermediate **4** are crucial for obtaining high selectivity and for the realization of this strategy. If the selectivity could be controlled in the DCCP/[1,2]-Wittig rearrangement in Fig. 2, the core structures of the antihistamines doxylamine,^6^ carbinoxamine,^7^ bepotastine,^8^ triprolidine^9^ (Fig. 1) and related pyridines^10^ could be accessed followed by further organic transformations.

Herein we disclose general and highly selective methods for the arylation of pyridylmethyl ethers **1** to give **4** as well as the tandem arylation/[1,2]-Wittig rearrangement to furnish tertiary alcohols **5**.

## 2. Results and discussion

### 2.1. Optimization of reactions

Given the importance of pyridines in medicinal chemistry and materials science,^11^ it is not surprising that palladium catalyzed C–H functionalization reactions involving benzylic arylation of pyridines and derivatives have attracted significant attention.^12^ Difficulties in the direct benzylic arylation of 2-substituted pyridines, however, have been encountered and are attributed to the ability of pyridyl nitrogens to act as ligands.12j,^13^ Successful strategies to circumvent this potential problem include addition of Lewis acids to bind the pyridyl nitrogen and increase the reactivity of the benzylic C–H's.12*j* Notably, acceleration of palladium catalyzed reactions of pyridine derivatives by addition of Lewis acids has been described by Nolan,^14^ Hartwig,^15^ and Trost.^16^ Other workarounds include use of substrates with directing groups, such as 2-(2-pyridyl)acetic acids,12*h* 2-(2-pyridyl)ethanols,12*b* pyridine *N*-oxides,12d,12f and *N*-iminopyridines.12*e* We previously used 2-benzyl pyridine (p*K*_a_ = 28.3–28.7 in THF), where the benzylic hydrogens are estimated to be about 6 orders of magnitude more acidic than those in 2-picoline (p*K*_a_ = 34 in THF).^17^ 2-Benzyl pyridines are also expected to be at least four orders of magnitude more acidic than the pyridylmethyl ether substrates that are the focus of the current investigation.^18^ Over the last decade, direct C–H functionalization of benzylic ethers has been reported *via* cross dehydrogenative-coupling (CDC) reactions and photoredox-mediated reactions.^19^

In the course of our studies^20^ in the DCCP of a variety of weakly acidic substrates, we discovered that palladium complexes of van Leeuwen's NIXANTPHOS ligand^21^ (Table 1) enable a wide variety of transformations whereas other ligands are much less effective. Thus, we employed a Pd(OAc)_2_ (5 mol%)/NIXANTPHOS (7.5 mol%) based catalyst to initiate the direct arylation of 2-pyridylmethyl ethyl ether **1a** with bromobenzene in combination with 6 bases [LiO–*t*Bu, NaO–*t*Bu, KO–*t*Bu, LiN(SiMe_3_)_2_, NaN(SiMe_3_)_2_, and KN(SiMe_3_)_2_]. Both LiO–*t*Bu and NaO–*t*Bu resulted in no product while KO–*t*Bu afforded a low yield of the arylation/[1,2]-Wittig rearrangement product. Interestingly, LiN(SiMe_3_)_2_ generated the arylation/[1,2]-Wittig rearrangement product **5aa** exclusively in 48% assay yield (AY, entry 1). Although NaN(SiMe_3_)_2_ gave a mixture of arylation **4aa** and arylation/[1,2]-Wittig rearrangement **5aa** in 25 and 38% AY, respectively (entry 2), KN(SiMe_3_)_2_ afforded traces of **4aa** and the arylation/[1,2]-Wittig rearrangement product **5aa** in 33% AY (entry 3).

**Table 1:** Optimization of the reactions of 2-pyridylmethyl ethyl ether **1a** with bromobenzene^*a*^

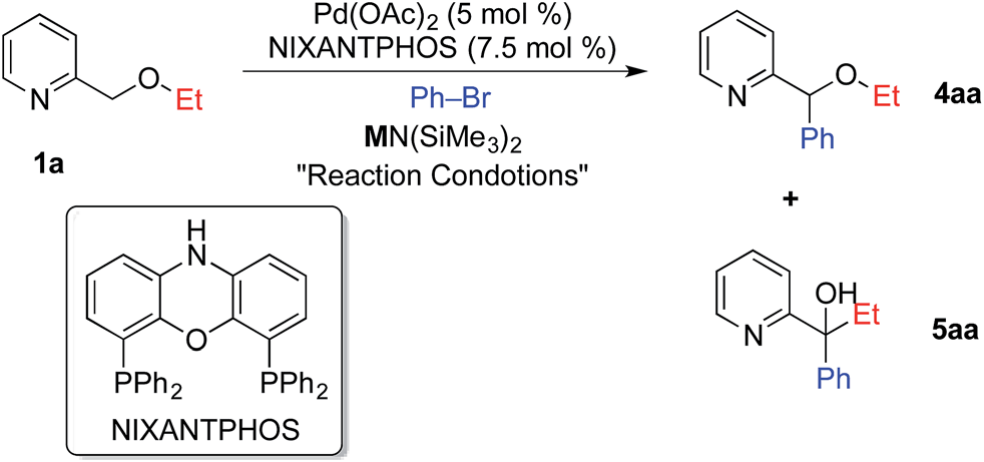						
Entry	M	Solvent^*b*^	Time (h)	Temp. (°C)	**4aa** ^*c*^ (%)	**5aa** ^*c*^ (%)
1	Li	THF	12	45	0	48
2	Na	THF	12	45	25	38
3	K	THF	12	45	Trace	33
4	Na	Dioxane	12	45	31	17
5	Na	CPME	12	45	33	65
6	Na	DME	12	45	64	30
**7**	**Na**	**DME**	**12**	**23**	**95** ^*d*^	**0**
8	Li	Dioxane	12	45	16	15
9	Li	DME	12	45	68	25
10	Li	CPME	12	45	38	56
11	Li	CPME	24	45	0	85^*d*^
**12** ^*e*^	**Li**	**CPME**	**24**	**45**	**0**	**83** ^*d*^

^*a*^Reaction conditions: **1a** (0.1 mmol), Ph–Br (0.12 mmol), base (0.3 mmol), Pd(OAc)_2_/NIXANTPHOS (5 mol%/7.5 mol%), solvent (1.0 mL).

^*b*^Tetrahydrofuran (THF), 1,4-dioxane (dioxane), cyclopentyl methyl ether (CPME), 1,2-dimethoxyethane (DME).

^*c*^Yield determined by ^1^H NMR spectroscopy of the crude reaction mixture.

^*d*^Isolated yield.

^*e*^Pd(OAc)_2_/NIXANTPHOS (2.5 mol%/3.75 mol%).

Given the higher yield afforded by NaN(SiMe_3_)_2_, we set out to examine different solvents with this base. Employing NaN(SiMe_3_)_2_ at 45 °C in dioxane, CPME and DME (entries 4–6) resulted in assay yields of up to 64% arylation (**4aa**) and 30% arylation/[1,2]-Wittig rearrangement (**5aa**) in DME. To our surprise, lowering the temperature to 23 °C in DME to minimize the [1,2]-Wittig rearrangement actually prevented it, providing the arylation product in 95% isolated yield (entry 7). With a highly selective protocol for the arylation in place, we desired to perform the tandem arylation/[1,2]-Wittig rearrangement.

The [1,2]-Wittig rearrangement of α-alkoxy carbanions has attracted much attention.^22^ Despite significant interest, relatively harsh reaction conditions are still the norm.^23^ There are only a few examples of the [1,2]-Wittig rearrangement in tandem reactions. Recently, Wolfe and co-workers introduced highly stereoselective tandem [1,2]-Wittig rearrangement/aldol or Mannich reactions.^24^ Dudley and co-workers reported pyridine-directed organolithium additions to enol ethers. The resulting intermediates then undergo anionic rearrangements to afford tertiary pyridyl carbinols.^25^ These examples indicate that the use of α-alkoxy carbanions in tandem reactions can be an effective strategy to generate multiple C–C bonds and forge complex molecular structures.

To develop the tandem arylation/[1,2]-Wittig rearrangement process, we focused on LiN(SiMe_3_)_2_ base in dioxane, DME and CPME (entries 8–10). Of these solvents, CPME gave the best results (**4aa** : **5aa**, 38 : 56), but exhibited incomplete [1,2]-Wittig rearrangement after 12 h (entry 10). With a 24 h reaction time, the tandem arylation/[1,2]-Wittig rearrangement product **5aa** was isolated in 85% yield (entry 11). As shown in entry 12, the palladium/NIXANTPHOS loading could be lowered to 2.5 and 3.75 mol%, respectively, without a significant drop in yield (83%).

### 2.2. Arylation of 2-pyridylmethyl ethers

Based on the optimized arylation conditions (Table 1, entry 7), we examined a variety of aryl bromides in the DCCP (Table 2). In general, the direct arylation of 2-pyridylmethyl ethyl ether (**1a**) exhibited excellent selectivity and good yields with a range of aryl bromides. Aryl bromides bearing electron-donating groups, such as 4-*t*Bu, 4-OMe, or 4-NMe_2_, resulted in 88–99% yields. 1-Bromo-4-fluorobenzene provided the product in 82% yield. Sterically hindered 2-bromotoluene coupled in 75% yield. Heterocyclic *N*-methyl 5-bromo indole and 5-bromo benzofuran underwent coupling to provide the products in 82–83% yield. It is noteworthy that when the reaction of **1a** and 4-*tert*-butyl bromobenzene was conducted on a 5 mmol scale, the arylation product **4ab** was isolated in 83% yield (1.12 g).

**Table 2:** Scope of aryl bromides in the arylation of 2-pyridylmethyl ethyl ether **1a**^*a*^

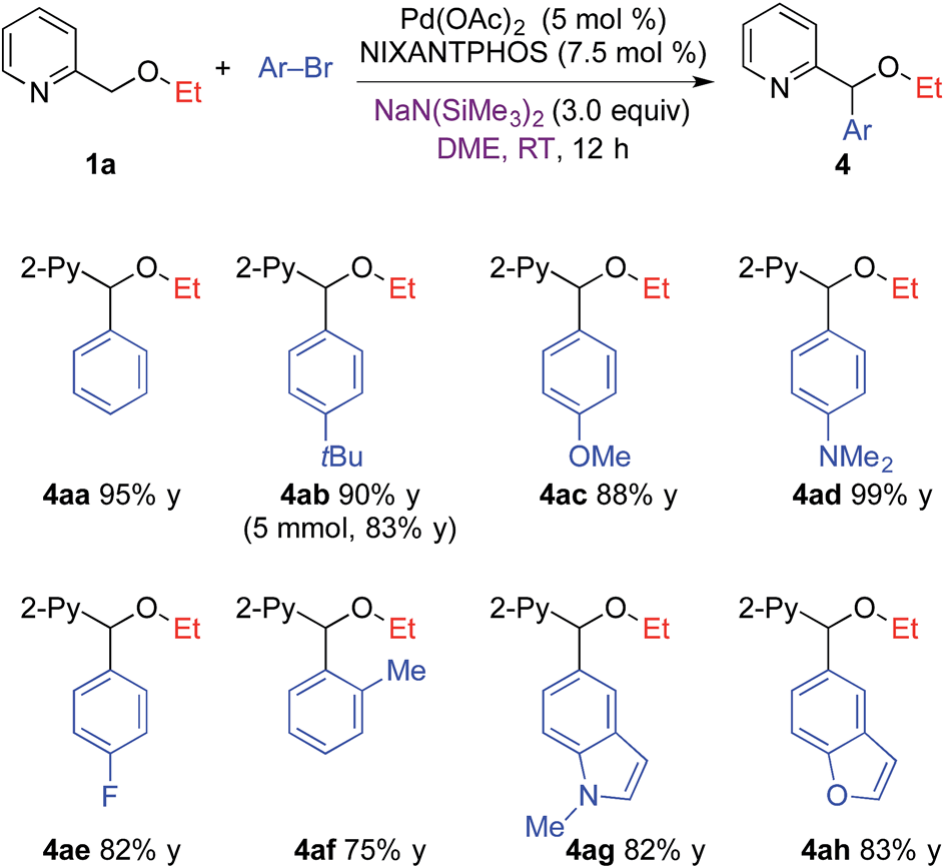

^*a*^Reaction conditions: **1a** (0.2 mmol), Ar–Br (0.24 mmol), NaN(SiMe_3_)_2_ (0.6 mmol), Pd(OAc)_2_/NIXANTPHOS (5 mol%/7.5 mol%), DME (2.0 mL); isolated yield.

The substrate scope of 2-pyridylmethyl ethers was next explored (Table 3) with electron-donating 4-bromo-*N*,*N*-dimethylaniline (entries 1–4) and with 1-bromo-4-fluorobenzene (entries 5–9). In general, all the 2-pyridylmethyl alkyl or aryl ethers exhibited 80–92% yield and excellent selectivity, with no [1,2]-Wittig product observed by ^1^H NMR of the crude reaction mixtures. 2-Pyridylmethyl alkyl ethers, including methyl ether (**1b**), cyclohexyl ether (**1c**) and *tert*-butyl ether (**1d**) showed good reactivity in the arylation reaction with 4-bromo-*N*,*N*-dimethylaniline and 1-bromo-4-fluorobenzene to furnish arylated ether products **4bd–4dd** (83–92%) and **4be–4de** (80–88%). In addition, 2-pyridylmethyl aryl ethers, including phenyl (**1e**), 4-fluorophenyl (**1f**) and 4-*tert*-butylphenyl ether (**1g**) coupled *via* DCCP to provide the corresponding products **4ed** (82%), **4fe** (82%) and **4ge** (80%).

**Table 3:** Scope of 2-pyridylmethyl ethers in the chemoselective arylation^*a*^

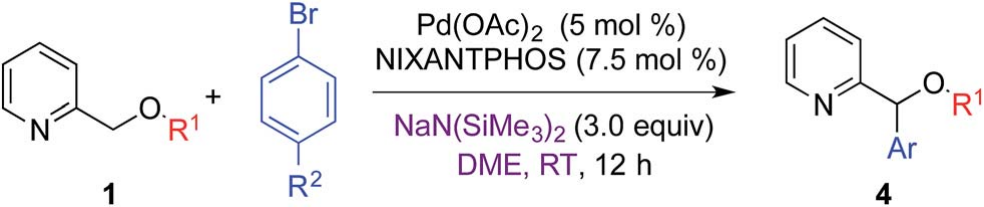						
Entry	**1**	R^1^	Py	R^2^	Product	Yield^*b*^ (%)
1	**1b**	Me	2-Py	NMe_2_	**4bd**	83
2	**1c**	Cy	2-Py	NMe_2_	**4cd**	92
3	**1d**	*t*Bu	2-Py	NMe_2_	**4dd**	88
4	**1e**	Ph	2-Py	NMe_2_	**4ed**	82
5	**1b**	Me	2-Py	F	**4be**	85
6	**1c**	Cy	2-Py	F	**4ce**	88
7	**1d**	*t*Bu	2-Py	F	**4de**	80
8	**1f**	4-F-C_6_H_4_	2-Py	F	**4fe**	82
9	**1g**	4-*t*Bu-C_6_H_4_	2-Py	F	**4ge**	80

^*a*^Reaction conditions: **1** (0.2 mmol), Ar–Br (0.24 mmol), NaN(SiMe_3_)_2_ (0.6 mmol), Pd(OAc)_2_/NIXANTPHOS (5 mol%/7.5 mol%), DME (2.0 mL).

^*b*^Isolated yield.

### 2.3. Tandem arylation/[1,2]-Wittig rearrangement

Having demonstrated the broad scope of the arylation, we then focused on the tandem arylation/[1,2]-Wittig rearrangement. Employing the optimized conditions in Table 1 (entry 12) with LiN(SiMe_3_)_2_ in CPME at 45 °C, reaction of 2-pyridylmethyl ethyl ether with aryl bromides was investigated (Table 4). Bromobenzene and 1-bromo-4-*tert*-butylbenzene furnished the desired products **5aa** and **5ab** in 85 and 78% yields, respectively. Aryl bromides bearing electron-donating 4-OMe and 4-NMe_2_ groups led to the coupled/rearranged products **5ac** and **5ad** in 75 and 72% yield, respectively. Aryl bromides with electron-withdrawing substituents, such as 4-F, 4-Cl and 3-CF_3_, were well tolerated, providing products **5ae**, **5ai** and **5aj** in 78–88% yields. Heterocyclic 5-bromo-1-methyl-1*H*-indole and 5-bromobenzofuran, also gave the compounds **5ag** and **5ah** in 72 and 70% yield. When the reaction of **1a** and 1-bromo-4-fluorobenzene was performed on 5 mmol scale with LiN(SiMe_3_)_2_ in CPME, the arylation/[1,2]-Wittig rearrangement product **5ae** was isolated in 74% yield (0.86 g). 3-Bromophenol was also examined, however, analysis of ^1^H NMR of the crude reaction mixtures showed no sign of arylation product or tandem product. Less than 10% of the pyridylmethyl ether **1a** was recovered, indicating decomposition of starting materials and/or products.

**Table 4:** Tandem arylation/[1,2]-Wittig rearrangement of 2-pyridylmethyl ethyl ether **1a** with aryl bromides^*a*^

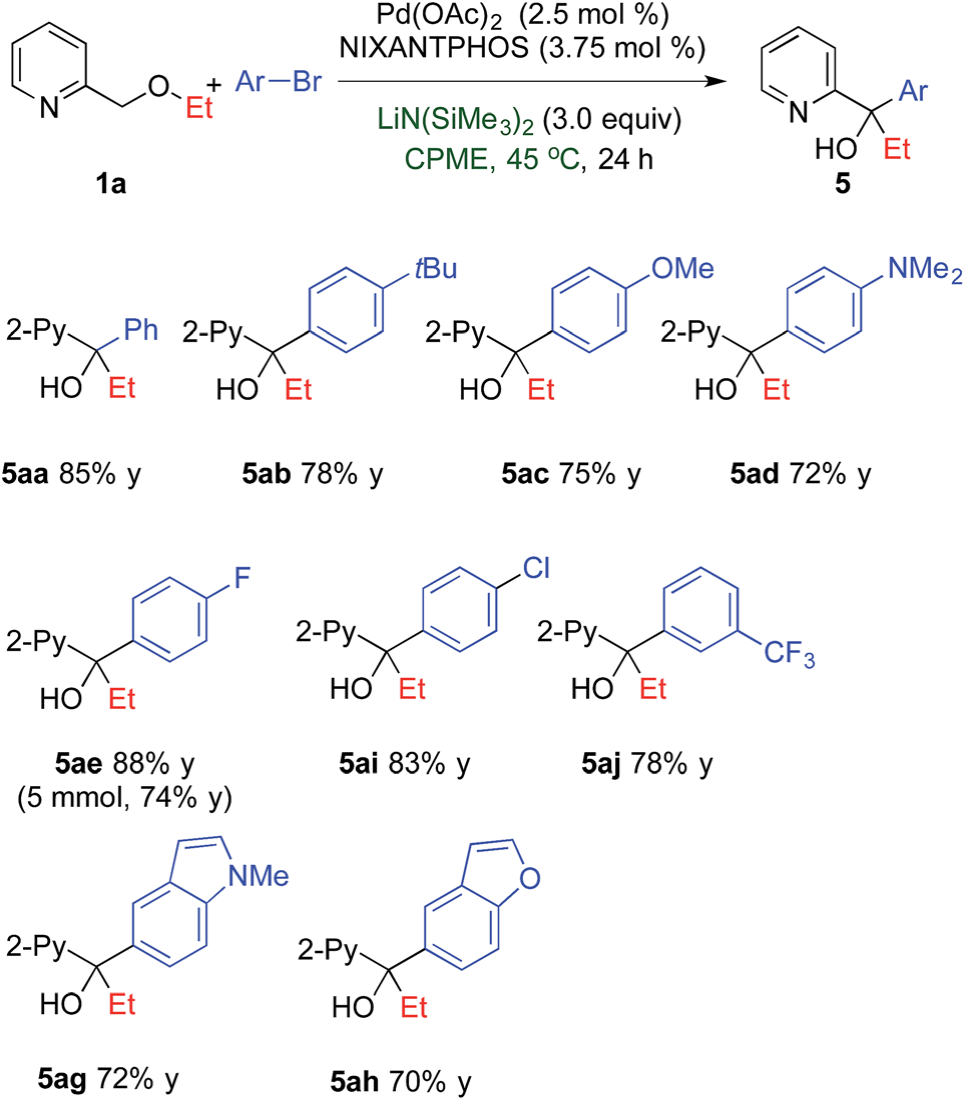

^*a*^Reaction conditions: **1a** (0.2 mmol), Ar–Br (0.24 mmol), LiN(SiMe_3_)_2_ (0.6 mmol), Pd(OAc)_2_/NIXANTPHOS (2.5 mol%/3.75 mol%), CPME (2.0 mL); isolated yields.

The scope in the migrating O–R group in the tandem arylation/[1,2]-Wittig rearrangement was investigated with various aryl groups. The 2-pyridylmethyl ethers with primary migrating groups (Table 4), methyl, secondary, and tertiary alkyl ethers (Table 5, entries 1–6) gave arylation/[1,2]-Wittig rearranged products in 60–85% yield. To examine selectivity in the oxidative addition step (aryl bromide *vs.* chloride) 2-pyridylmethyl *tert*-butyl ether was coupled with 1-bromo-4-chlorobenzene (entry 6). The arylation/[1,2]-Wittig rearrangement product **5di** was obtained in 60% isolated yield, despite the known ability of the catalyst to activate aryl chlorides at rt.20*p* Amino ethyl-containing R groups also migrated, giving the desired amines in 64–74% yield (entries 7–10).

**Table 5:** Tandem arylation/[1,2]-Wittig rearrangement of 2-pyridylmethyl alkyl ethers with aryl bromides^*a*^

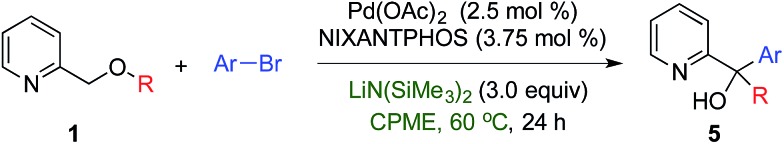				
Entry	R	Ar	Product	Yield (%)
1^*b*^	Me	4-C_6_H_4_-NMe_2_	**5bd**	72
2^*b*^	Me	4-C_6_H_4_-F	**5be**	75
3	Cy	4-C_6_H_4_-NMe_2_	**5cd**	82
4	Cy	4-C_6_H_4_-F	**5ce**	85
5	*t*Bu	4-C_6_H_4_-NMe_2_	**5dd**	65
6	*t*Bu	4-C_6_H_4_-Cl	**5di**	60
7	CH_2_CH_2_NMe_2_	C_6_H_5_	**5ha**	74
8	CH_2_CH_2_NMe_2_	4-C_6_H_4_-Cl	**5hi**	74
9	CH_2_CH_2_N(CH_2_)_4_	C_6_H_5_	**5ia**	64
10	CH_2_CH_2_N(CH_2_)_4_	4-C_6_H_4_-Me	**5ik**	71

^*a*^Reaction conditions: **1** (0.2 mmol), Ar–Br (0.24 mmol), LiN(SiMe_3_)_2_ (0.6 mmol), Pd(OAc)_2_/NIXANTPHOS (2.5 mol%/3.75 mol%), CPME (2.0 mL); isolated yields; no arylated ethers observed by ^1^H NMR of crude reaction mixture.

^*b*^45 °C.

### 2.4. Arylation of 4-pyridylmethyl ethers

Our focus on the arylation and tandem arylation/[1,2]-Wittig rearrangement of 2-pyridylmethyl ethers stems from their utility in the preparation of biologically active compounds (Fig. 1). At this juncture, we were curious if the 2-pyridyl group was required for the deprotonation and arylation outlined above. To probe this question, as well as broaden the scope of our coupling chemistry, we examined reactions of 4-pyridylmethyl alkyl ethers (**1j** and **1k**, Table 6) with various aryl bromides.

**Table 6:** Scope of 4-pyridylmethyl ethers in the chemoselective arylation

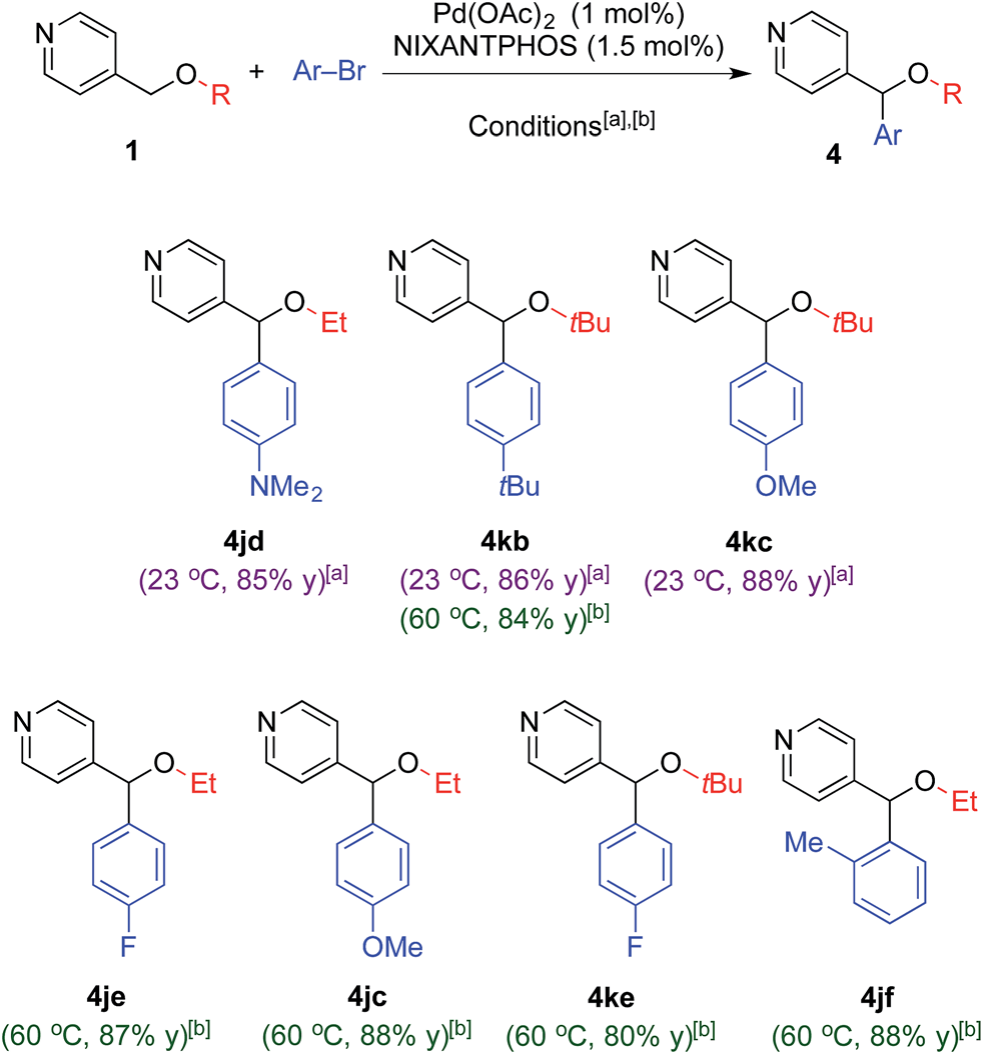

^*a*^Conditions A: **1** (0.2 mmol), Ar–Br (0.24 mmol), NaN(SiMe_3_)_2_ (0.6 mmol), Pd(OAc)_2_/NIXANTPHOS (1 mol%/1.5 mol%), DME (2.0 mL) at 23 °C.

^*b*^Conditions B: **1** (0.2 mmol), Ar–Br (0.24 mmol), LiN(SiMe_3_)_2_ (0.6 mmol), Pd(OAc)_2_/NIXANTPHOS (1 mol%/1.5 mol%), CPME (2.0 mL) at 60 °C.

Under our standard arylation conditions [NaN(SiMe_3_)_2_ in DME at 23 °C], arylation of 4-pyridylmethyl alkyl ethers (**1j** and **1k**) with aryl bromides provided the arylated products **4jd**, **4kb** and **4kc** in 85–88% yield (Table 6) demonstrating that chelation is not a prerequisite to deprotonation or arylation. The increased reactivity of the 4-pyridyl derivatives over the 2-pyridyl analogues enabled us to reduce the palladium loading to 1 mol% in these cases.

To our surprise, conducting the reaction under the tandem arylation/[1,2]-Wittig rearrangement conditions [LiN(SiMe_3_)_2_ in CPME at 60 °C] did not provide the tandem arylation/rearranged products **5**, but instead furnished the arylated ether products in 80–88% yield. While the tandem arylation/[1,2]-Wittig reaction of **1j** with LiN(SiMe_3_)_2_ in CPME at 60 °C resulted in arylation product **4je** in Table 6, the same condition at 80 °C for 24 h showed 10% of tandem arylation/[1,2]-Wittig rearrangement product and 72% of arylation product **4je**. Higher reaction temperatures resulted in decomposition. These results suggest that [1,2]-Wittig rearrangement of 4-pyridylmethyl ethers is possible, but require higher activation energy. This difference in reactivity between 2- and 4-pyridylmethyl ethers most likely stems from the increased stability of the deprotonated 4-pyridylmethyl ethers. We are aware of a few examples of [1,2]-Wittig rearrangements of 4-pyridyl methyl ethers in the literature.^26^ The mechanism of [1,2]-Wittig rearrangement proceeds *via* radical intermediates. The radical intermediates derived from 4-pyridylmethyl ethers are expected to be higher in energy than those generated from 2-pyridylmethyl ethers.^27^

### 2.5. Comparative studies

**Scheme 1:**
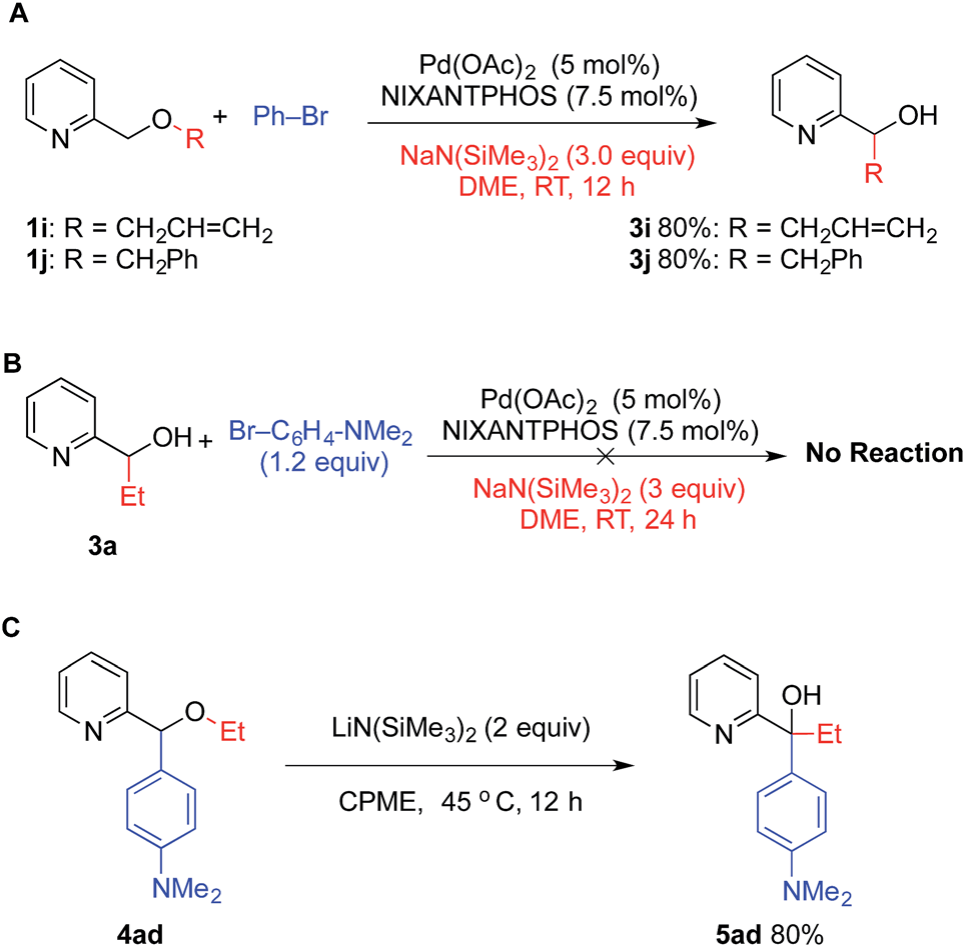
Probing the order of the tandem arylation/[1,2]-Wittig rearrangement. (A) [1,2]-Wittig rearrangement of allyl and benzyl ethers is faster than Pd-catalyzed arylation reactions. (B) [1,2]-Wittig rearrangement product does not undergo arylation. (C) [1,2]-Wittig rearrangement takes place in the absence of the Pd-catalyst.

While the scope of the substrates in Tables 2–6 is very good, some substrates were not compatible with the optimized conditions. This occurred when the O–R substituent was allyl or benzyl (Scheme 1A). These substrates underwent [1,2]-Wittig rearrangement faster than arylation, highlighting the fine line in chemoselectivity between the [1,2]-Wittig rearrangement of the carbanion **2** relative to transmetallation to palladium (Fig. 2). Additionally, 2-pyridylmethyl aryl ethers did not undergo the [1,2]-Wittig rearrangement. These observations are in accord with the mechanism of [1,2]-Wittig rearrangement, which proceeds *via* free radical intermediates.22*a* The general order of reactivity of migrating groups in the Wittig rearrangement is dictated by the stability of the corresponding radicals: allyl ∼ benzyl > alkyl > aryl.^28^

To probe the order of the reactions leading to the tertiary alcohols **5**, (pyridin-2-yl)propan-1-ol **3a** was prepared by [1,2]-Wittig rearrangement of **1a** with *n*-BuLi in THF at –40 °C.4*d* When alcohol **3a** was subjected to our arylation conditions, no arylation product was observed (Scheme 1B) and starting material was recovered. In contrast, ether **4ad** underwent [1,2]-Wittig rearrangement upon exposure to LiN(SiMe_3_)_2_ in the absence of the Pd(NIXANTPHOS)-based catalyst in 80% yield (Scheme 1C). Thus, the [1,2]-Wittig rearrangement does not require palladium to occur. Taken together, these results suggest that arylation occurs before [1,2]-Wittig rearrangement.

### 2.6. Impact of Li and Na on [1,2]-Wittig rearrangements

[1,2]-Wittig rearrangements are known to be influenced by combinations of alkali metals and solvents.23d,^29^ Although a full mechanistic study of the [1,2]-Wittig rearrangement is beyond the scope of this manuscript, we hoped to gain some insight into the reactivity differences in the [1,2]-Wittig rearrangement with lithium and sodium silylamide bases. Thus, we decided to explore the rearrangement of isolated arylation product **4ab** (Table 7). In this study, 2 bases [LiN(SiMe_3_)_2_ and NaN(SiMe_3_)_2_], 2 solvents [DME and CPME], and 2 additives [12-crown-4 and 15-crown-5] were examined. Reactions were conducted at 45 °C to give higher amounts of [1,2]-Wittig rearrangement product than reactions conducted at room temperature (as in Tables 1–3). Reactions were intentionally quenched before completion to assess reaction progress. The quantities of starting materials and products were determined by ^1^H NMR integration of reaction mixtures against an internal standard (dibromomethane, see ESI† for details).

**Table 7:** Effect of alkali metals, solvents, and additives in [1,2]-Wittig rearrangement of **4ab**^*a*^

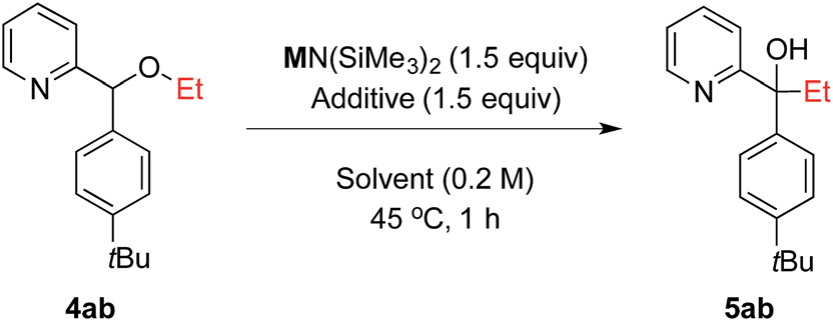					
Entry	Solvent	M	Additive	**4ab** ^*b*^ (%)	**5ab** ^*b*^ (%)
1	CPME	Li	—	37	64
2	CPME	Na	—	24	75
3	DME	Li	—	72	27
4	DME	Na	—	85	9
5	CPME	Li	12-Crown-4	53	47
6	CPME	Na	12-Crown-4	90	7
7	CPME	Na	15-Crown-5	86	13

^*a*^Reaction conditions: **4ab** (0.05 mmol), MN(SiMe_3_)_2_ (0.075 mmol), additive (0.075 mmol) in solvent (0.25 mL) at 45 °C.

^*b*^Yield determined by ^1^H NMR spectroscopy of the crude reaction mixture.

The results of this study are illustrated in Table 7. Reactions in CPME promoted by LiN(SiMe_3_)_2_ and NaN(SiMe_3_)_2_ undergo relatively fast [1,2]-Wittig rearrangements at 45 °C (entries 1 and 2). In DME both bases are less capable of promoting the rearrangement, but NaN(SiMe_3_)_2_ is significantly slower than LiN(SiMe_3_)_2_ (9 *vs.* 27% [1,2]-Wittig rearrangement product, entries 3 and 4, respectively). This result suggested that coordinating DME retards the rearrangement with the sodium counterion more so than with the lithium counterion. To probe this hypothesis, reactions were conducted in the presence of 12-crown-4 and 15-crown-5 in the less coordinating CPME. The impact of 12-crown-4/CPME on the LiN(SiMe_3_)_2_ reaction is a slight decrease in the conversion (47 *vs.* 64%, entry 5 *vs.* 1). In contrast, crown ethers 12-crown-4 and 15-crown-5 strongly inhibited the [1,2]-Wittig rearrangement with NaN(SiMe_3_)_2_, resulting in only 7 and 13% product formation (entries 6–7, respectively) compared to 75% in CPME in the absence of crown ether (entry 2). Thus, we conclude that coordinating solvent (DME) and coordinating additives (12-crown-4 and 15-crown-5) both inhibit the reaction with the sodium base to a much greater extent than with the lithium base. These observations are consistent with the results in Table 1.

## 3. Conclusions

In summary, we have developed a unified approach for the synthesis of structurally diverse pyridines, which are among the most important heterocycles and are found in many natural products and pharmaceutically relevant molecules (Fig. 1). The key to achieving skeletal diversity from a single starting material is to affect either the arylation or tandem arylation/[1,2]-Wittig rearrangement with high selectivity. Despite the remarkable similarity of the reaction parameters for these processes, both being promoted by silylamide bases and a (NIXANTPHOS) Pd-based catalyst, we have successfully identified conditions to strongly favor either reaction. The exceptional selectivity between secondary pyridyl ethers and their rearranged tertiary alcohol isomers is govern by the choice of main group metal (Na *vs.* Li), solvent, and temperature. We anticipate that these practical methods will be applicable for the synthesis of a broad array of pyridyl-containing ethers and alcohols.

## Supplementary Material

Supplementary information
