# Supplementary material for: Chemoselective palladium-catalyzed deprotonative arylation/[1,2]-Wittig rearrangement of pyridylmethyl ethers
Source: Chem Sci. 2015 Oct 27;7(2):976–83. doi: 10.1039/c5sc02739j (PMC4800319; doi:10.1039/c5sc02739j)
Supplement: Supplementary file 1 [file SC-007-C5SC02739J-s001.pdf]

Electronic Supplementary Material (ESI) for Chemical Science.
This journal is © The Royal Society of Chemistry 2015

SUPPORTING INFORMATION

Chemoselective Palladium-Catalyzed Deprotonative Arylation/[1,2]-Wittig Rearrangement of Pyridylmethyl Ethers

Feng Gao,[†,‡,§] Byeong-Seon Kim,[‡,§] and Patrick J. Walsh,[‡,] *

[†]Department of Medicinal Plants, Agronomy College, Sichuan Agricultural University, 211, Huimin Rd, Wenjiang Region, Chengdu 611130, PR China

[‡] Roy and Diana Vagelos Laboratories, Penn/Merck Laboratory for High-Throughput Experimentation, Department of Chemistry, University of Pennsylvania, 231 S, 34th St., Philadelphia, PA 19104-6323 (USA)

[§]These authors contributed equally.

E-mail: pwalsh@sas.upenn.edu

TABLE OF CONTENTS

1. General Methods……………………………………………………...……………..…S2
2. Preparation of pyridylmethyl ethers…..…………………………………...……..S2
3. Procedure and characterization of Pd-catalyzed selective C(sp^3)–H arylation of 2-pyridylmethyl ethers (Table 2 and 3) ……………………..……...…..…..…...S9
4. Procedure and characterization of Pd-catalyzed tandem arylation/[1,2]-Wittig rearrangement of 2-pyridylmethyl ethers (Table 4 and 5) ……..……...…..…...S21
5. Procedure and characterization of Pd-catalyzed selective C(sp^3)–H arylation of 4-pyridylmethyl ethers (Table 6) ……………………………......………..…...S34
6. Confirmation of the order of tandem arylation/[1,2]-Wittig rearrangement (Scheme 1).....................………...………...………...………...………...………...……….......S38
7. Effect of Alkali Metals, Solvents and Additives in [1,2]-Wittig Rearrangement of Arylation product (Table 7) ......………..........………......………....………....……..S40
8. Reference…………………………………………………………….…………S42
9. NMR spectra………………………………………………………………………S43

1. General Methods

All reactions were conducted under an inert atmosphere of dry nitrogen. Anhydrous dioxane and cyclopentyl methyl ether (CPME) were purchased from Sigma-Aldrich and used without further purification. Dimethoxyethane (DME) and tetrahydrofuran (THF) were dried through activated alumina columns under nitrogen. Unless otherwise stated, Silica gel (Silicaflash, P60, 40-63 μm, Silicycle) was used for air-flashed chromatography. Solvents were commercially available and used as received without further purification. Chemicals were purchased from Sigma-Aldrich, Acros, Fisher Scientific or Matrix Scientific and solvents were obtained from Fisher Scientific. Thin-layer chromatography was performed on Whatman precoated silica gel 60 F-254 plates and visualized by ultraviolet light. Flash chromatography was performed with Silica gel (Silicaflash, P60, 40-63 μm, Silicycle). NMR spectra were obtained using a Brüker 500 MHz Fourier-transform NMR spectrometer at the University of Pennsylvania NMR facility. ^{1}H and ^{13}C chemical shifts in parts per million (δ) were referenced to internal tetramethylsilane (TMS). The designation "ABq" for a ^{1}H NMR resonances indicates that the particular peak was one partner of an AB quartet; if additional splittings were evident, they are noted following the ABq designation (e.g., ABqq). The infrared spectra were obtained with KBr plates using a Perkin-Elmer Spectrum 1600 Series spectrometer. High-resolution mass spectrometry (HRMS) data were obtained on a Waters LC-TOF mass spectrometer (model LCT-XE Premier) using chemical ionization (CI) or electrospray ionization (ESI) in positive or negative mode, depending on the analyte. Melting points were determined on a Unimelt Thomas-Hoover melting point apparatus and are uncorrected. 2-(Chloromethyl)pyridine hydrochloride (98%) was purchased from Matrix Scientific and used as received.

2. Preparation of pyridylmethyl ethers

General Procedure A using sodium alkoxide: To an oven-dried 25 mL two-neck flask equipped with a stir bar was charged with NaOEt (2.04 g, 30 mmol) inside a

glove box and the flask was capped with a reflux condenser and a septum and brought out of the glove box. To the flask was added EtOH (5 mL, 86 mmol) and 2-(chloromethyl)pyridine hydrochloride (2.0 g, 12 mmol) under positive pressure of N_2 at room temperature and the reaction mixture was stirred and heated to refluxed for 4–8 h until TLC showed complete consumption of 2-(chloromethyl)pyridine. Next, the remaining EtOH was removed under reduced pressure and the residue was purified by flash column chromatography on silica gel to afford the desired products.[1]

***General Procedure B using sodium hydride and alcohols**:* To an oven-dried 100 mL one-necked flask with a stir bar was charged with NaH (dry, 95%) (0.73 g, 30 mmol) under N_2. The flask was capped with a septum. To the flask was added dry THF (25 mL) at rt, and the reaction mixture was cooled to 0 °C in an ice bath. To this white suspension was added dropwise cyclohexanol (5 mL, 48 mmol). Upon addition of the cyclohexanol, rapid evolution of a gas was observed. Once the H_2 evolution ceased, 2-(chloromethyl)pyridine hydrochloride (2.0 g, 12 mmol) was added and the resulting solution was heated to reflux for 8 h until TLC showed complete consumption of 2-(chloromethyl)pyridine. Next, the remaining cyclohexanol was removed under reduced pressure and the residue was purified by flash column chromatography on silica gel to afford the desired products.[1]

General Procedure C using sodium hydroxide and phenol derivatives: In a 25 mL two-necked flask equipped with a stir bar was added the 2-(chloromethyl)pyridine hydrochloride (2.0 g, 12 mmol), sodium hydroxide (1.2 g, 30 mmol), phenol (12 mmol) and toluene (5 mL) at room temperature. The flask was capped with a reflux condenser and a septum. The resulting biphasic mixture was stirred and heated refluxed for 4–8 h until TLC showed complete consumption of 2-(chloromethyl)pyridine. Then toluene was removed under reduced pressure. The residue was purified by flash column chromatography on silica gel to afford desired products.[2]

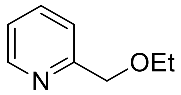

2-(Ethoxymethyl)pyridine (1a): The reaction was performed following General Procedure A with 2-(chloromethyl)pyridine hydrochloride (2.0 g, 12 mmol), sodium ethoxide (2.04 g, 30 mmol) and ethanol (5 mL, 86 mmol). The reaction mixture was stirred and refluxed for 4 h. The crude product was purified by flash chromatography on silica gel (eluted with EtOAc:hexanes = 1:6) to afford the product (1.32 g, 80% yield) as a yellow oil. The ^{1}H and $^{13}C\{^{1}H\}$ NMR data for this compound match the literature data.[3]

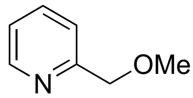

2-(Methoxymethyl)pyridine (1b): The reaction was performed following General Procedure A with 2-(chloromethyl)pyridine hydrochloride (2.0 g, 12 mmol), sodium methoxide (1.62 g, 30 mmol) and methanol (5 mL). The reaction mixture was stirred and refluxed for 4 h. The crude product was purified by flash chromatography on silica gel (eluted with EtOAc:hexanes = 1:6) to afford the product (1.25 g, 85% yield) as a colorless oil. The ^{1}H and $^{13}C\{^{1}H\}$ NMR data for this compound match the literature data.[4]

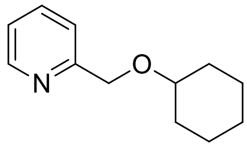

2-((Cyclohexyloxy)methyl)pyridine (1c): The reaction was performed following General Procedure B with 2-(chloromethyl)pyridine hydrochloride (2.0 g, 12 mmol), sodium hydride (0.73 g, 30 mmol) and cyclohexanol (5 mL). The reaction mixture was stirred and refluxed for 8 h. The crude product was purified by flash chromatography on silica gel (eluted with EtOAc:hexanes = 1:6) to afford the product (2.00 g, 88% yield) as a yellow oil. ^{1}H NMR (500 MHz, $CDCl_3$): δ 8.47 (d, J = 4.0 Hz, 1H), 7.62 (t, J = 8.0 Hz, 1H), 7.44 (d, J = 8.0 Hz, 1H), 7.10 (t, J = 6.5 Hz, 1H), 4.61 (s, 2H), 3.37 – 3.34 (m, 1H), 1.95 – 1.90 (m, 2H), 1.69 – 1.72 (m, 2H), 1.49 – 1.21 (m, 6H) ppm; $^{13}C\{^{1}H\}$ NMR (125 MHz, $CDCl_3$): δ 159.5, 148.7, 136.3, 121.9, 121.1, 77.6, 70.7, 32.1, 25.7, 23.9 ppm; HRMS m/z 192.1383 [$(M+H)^+$;

calcd for $C_{12}H_{18}NO$: 192.1388].

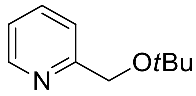

2-(*tert*-Butoxymethyl)pyridine (1d): The reaction was performed following General Procedure A with 2-(chloromethyl)pyridine hydrochloride (2.0 g, 12 mmol), sodium *tert*-butoxide (2.88 g, 30 mmol) and *tert*-butanol (5 mL). The reaction mixture was stirred and refluxed for 8 h. The crude product was purified by flash chromatography on silica gel (eluted with EtOAc:hexanes = 1:6) to afford the product (1.78 g, 90% yield) as a yellow oil. ^{1}H NMR (500 MHz, $CDCl_3$): δ 8.46 (d, J = 5.0 Hz, 1H), 7.60 (t, J = 8.0 Hz, 1H), 7.43 (d, J = 8.0 Hz, 1H), 7.09 – 7.06 (m, 1H), 4.54 (s, 2H), 1.28 (s, 9H) ppm; $^{13}C\{^1H\}$ NMR (125 MHz, $CDCl_3$): δ 160.1, 148.6, 136.3, 121.7, 121.0, 73.6, 65.2, 27.5 ppm; HRMS *m/z* 166.1226 [$(M+H)^+$; calcd for $C_{10}H_{16}NO$: 166.1232].

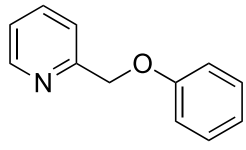

2-(Phenoxymethyl)pyridine (1e): The reaction was performed following General Procedure C with 2-(chloromethyl)pyridine hydrochloride (2.0 g, 12 mmol), phenol (1.13 g, 12 mmol), sodium hydroxide (1.2 g, 30 mmol) and toluene (5 mL). The reaction mixture was stirred and refluxed for 4 h. The crude product was purified by flash chromatography on silica gel (eluted with EtOAc:hexanes = 1:6) to afford the product (2.0 g, 90% yield) as a yellow oil. The ^{1}H and $^{13}C\{^1H\}$ NMR data for this compound match the literature data.[5]

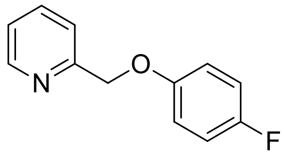

2-((4-Fluorophenoxy)methyl)pyridine (1f): The reaction was performed following General Procedure C with 2-(chloromethyl)pyridine hydrochloride (2.0 g, 12 mmol), 4-fluorophenol (1.34 g, 12 mmol), sodium hydroxide (1.2 g, 30 mmol) and toluene (5 mL). The reaction mixture was stirred and refluxed

for 6 h. The crude product was purified by flash chromatography on silica gel (eluted with EtOAc:hexanes = 1:6) to afford the product (2.09 g, 86% yield) as a white solid. m.p. = 52 – 55 °C; ^{1}H NMR (500 MHz, $CDCl_3$): δ 8.57 (d, *J* = 5.0 Hz, 1H), 7.69 (t, *J* = 8.0 Hz, 1H), 7.48 (d, *J* = 8.0 Hz, 1H), 7.21 – 7.19 (m, 1H), 6.96 – 6.88 (m, 4H), 5.14 (s, 2H) ppm; ^{13}C{^{1}H} NMR (125 MHz, $CDCl_3$): δ 157.4 (d, *J* = 250 Hz), 157.0, 154.4 (d, *J* = 1.75 Hz), 149.1, 136.7, 122.6, 121.2, 115.9, 115.7 (d, *J* = 25 Hz), 71.1 ppm; HRMS *m/z* 204.0819 [(M+H)$^+$; calcd for $C_{12}H_{11}NOF$: 204.0825].

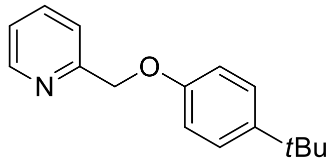

2-((4-(*tert*-Butyl)phenoxy)methyl)pyridine (1g): The reaction was performed following General Procedure C with 2-(chloromethyl)pyridine hydrochloride (2.0 g, 12 mmol), 4-*tert*-butylphenol (1.8 g, 12 mmol), sodium hydroxide (1.2 g, 30 mmol) and toluene (5 mL). The reaction mixture was stirred and refluxed for 8 h. The crude product was purified by flash chromatography on silica gel (eluted with EtOAc:hexanes = 1:6) to afford the product (2.17 g, 76% yield) as a thin yellow oil. ^{1}H NMR (500 MHz, $CDCl_3$): δ 8.56 (br. s, 1H), 7.66 (br s, 1H), 7.51 (br s, 1H), 7.28 (d, *J* = 9.0 Hz, 2H), 7.18 (br s. 1H), 6.90 – (d, *J* = 9.0 Hz, 2H), 5.17 (s, 2H), 1.27 (s, 9H) ppm; ^{13}C{^{1}H} NMR (125 MHz, $CDCl_3$): δ 157.5, 156.0, 149.0, 143.7, 136.7, 126.2, 122.4, 121.2, 114.1, 70.5, 34.5, 31.4 ppm. The ^{1}H NMR data for this compound match the literature data.[6]

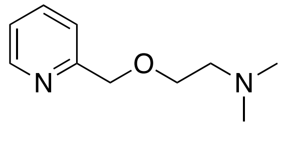

***N,N*-Dimethyl-2-(pyridin-2-ylmethoxy)ethan-1-amine (1h):** To an oven-dried round-bottomed flask equipped with a stir bar was added sodium *tert*-butoxide (3.36 g. 30 mmol) and THF (20 mL) in a dry box under N_2 at room temperature and the flask was then capped with a septum. The flask was brought out of the glove box and the reaction vessel was cooled to 0 °C in an ice bath and stirred for 5 min under a nitrogen atmosphere. *N,N*-Dimethylethanolamine (1.81 mL, 18

mmol) was added dropwise, after which the reaction vessel was stirred for 10 min at 0 °C. To the reaction vessel was added dropwise 2-(chloromethyl)pyridine (2.55 g, 20 mmol) in THF (10 mL). The reaction mixture was stirred at 0 °C for 10 min, the ice bath was removed and the reaction mixture stirred for 12 h at room temperature. The reaction mixture was diluted with EtOAc (20 mL) and quenched with saturated aqueous NH_4Cl (30 mL). The organic layer was separated and the aqueous solution was extracted with EtOAc (4 x 20 mL). The combined organic layer was washed with saturated aqueous $NaHCO_3$ solution (30 mL) and then brine (30 mL), dried over $MgSO_4$, filtered, and the solvent was removed *in vacuo* to yield a yellow oil. The crude product was purified by flash column chromatography on silica gel (dichloromethane to dichloromethane:methanol = 10:1) to afford the product as a brown oil (2.34 g, 72% yield). ^{1}H NMR (500 MHz, $CDCl_3$) δ 8.52 (ddd, J = 5.0, 1.5, 1.0 Hz, 1H), 7.66 (td, J = 7.5, 1.2 Hz, 1H), 7.43 (d, J = 5.0 Hz, 1H), 7.18 – 7.13 (m, 1H), 4.64 (s, 2H), 3.64 (t, J = 5.8 Hz, 2H), 2.56 (t, J = 5.8 Hz, 2H), 2.26 (s, 6H) ppm; ^{13}C{^{1}H} NMR (125 MHz, $CDCl_3$) δ 158.7, 149.2, 136.8, 122.5, 121.6, 74.2, 69.0, 59.0, 46.0 ppm; IR (neat) 3438, 3067, 3012, 2942, 2862, 2819, 2769, 1591, 1572, 1457, 1435, 1353, 1269, 1192, 1046 cm^{-1}; HRMS *m/z* 180.1341 [$(M+H)^+$; calcd for $C_{10}H_{17}N_2O$: 181.1341].

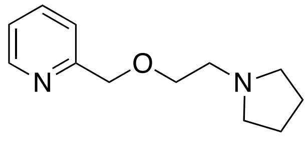

2-((2-(Pyrrolidin-1-yl)ethoxy)methyl)pyridine (1i): The reaction was performed following the procedure for **1h** with sodium *tert*-butoxide (3.36 g. 30 mmol), *N*-(2-hydroxyethyl)pyrrolidine (2.10 mL, 18 mmol), 2-(chloromethyl)pyridine (2.55 g, 20 mmol) and THF (30 mL). The crude product was purified by flash column chromatography on silica gel (dichloromethane to dichloromethane:methanol = 10:1) to afford the product (2.52 g, 72% yield). ^{1}H NMR (500 MHz, $CDCl_3$) δ 8.52 (ddd, J = 5.0, 1.5, 1.0 Hz, 1H), 7.66 (td, J = 7.5, 1.5 Hz, 1H), 7.43 (d, J = 5.0 Hz, 1H), 7.18 – 7.12 (m, 1H), 4.66 (s, 2H), 3.68 (t, J = 6.0 Hz, 2H), 2.75 (t, J = 6.0 Hz, 2H), 2.61 – 2.51 (m, 4H), 1.82 – 1.72 (m, 4H) ppm; ^{13}C{^{1}H}

NMR (125 MHz, $CDCl_3$) δ 158.8, 149.2, 136.7, 122.4, 121.5, 74.2, 70.0, 55.8, 54.8, 23.6 ppm; IR (neat) 3468, 3065, 3011, 2963, 2931, 2874, 2782, 1683, 1591, 1571, 1476, 1460, 1435, 1351, 1292, 1147, 1121, 1088, 1047 cm^{-1}; HRMS *m/z* 207.1498 $[(M+H)^+]$; calcd for $C_{12}H_{19}N_2O$: 207.1497].

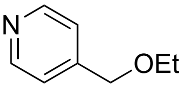
4-(Ethoxymethyl)pyridine (1j): The reaction was performed following General Procedure A with 4-(chloromethyl)pyridine hydrochloride (2.0 g, 12 mmol), sodium ethoxide (2.04 g, 30 mmol) and ethanol (5 mL). The reaction mixture was stirred and refluxed for 4 h. The crude product was purified by flash chromatography on silica gel (eluted with EtOAc:hexanes = 1:2) to afford the product (1.32 g, 80% yield) as a thin red oil. ^{1}H NMR (500 MHz, $CDCl_3$): δ 8.23 (m, 2H), 7.23 (m, 2H), 4.48 (s, 2H), 3.56 – 3.52 (m, 2H), 1.25 – 1.22 (m, 3H) ppm; $^{13}C\{^1H\}$ NMR (125 MHz, $CDCl_3$): δ 149.8, 147.8, 121.7, 70.9, 65.3, 15.1 ppm.

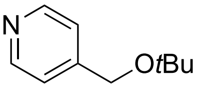
4-(*tert*-Butoxymethyl)pyridine (1k): The reaction was performed following General Procedure A with 4-(chloromethyl)pyridine hydrochloride (2.0 g, 12 mmol), sodium *tert*-butoxide (2.9 g, 30 mmol) and *tert*-butanol (5 mL). The reaction mixture was stirred and refluxed for 8 h. The crude product was purified by flash chromatography on silica gel (eluted with EtOAc:hexanes = 1:2) to afford the product (1.43 g, 72% yield) as a yellow oil. ^{1}H NMR (500 MHz, $CDCl_3$): δ 8.50 (dd, J = 4.5, 1.5 Hz, 2H), 7.24 (m, 2H), 4.43 (s, 2H), 1.25 (s, 9H) ppm; $^{13}C\{^1H\}$ NMR (125 MHz, $CDCl_3$): δ 149.5, 149.2, 121.6, 73.8, 62.5, 27.5 ppm. IR (neat) 3029, 2975, 2934, 2874, 1722, 1605, 1562, 1472, 1415, 1391, 1364, 1322, 1298, 1235, 1195, 1099, 1075, 1066, 1027, 993 cm^{-1}

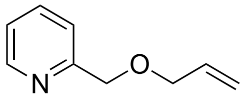
2-(Allyloxymethyl)pyridine (1l): The reaction was performed following General Procedure C with 2-(chloromethyl)pyridine hydrochloride (2.0 g,

12 mmol), prop-2-en-1-ol (0.7 g, 12 mmol), sodium hydroxide (1.2 g, 30 mmol) and toluene (5 mL). The reaction mixture was stirred and refluxed for 6 h. The crude product was purified by flash chromatography on silica gel (eluted with EtOAc:hexanes = 1:5) to afford the product (1.16 g, 65% yield) as a yellow oil. ^{1}H NMR (500 MHz, $CDCl_3$): δ 8.54 (ddd, *J* = 5.0, 1.5, 1.0 Hz, 1H), 7.70 – 7.67 (m, 1H), 7.46 (d, *J* = 8.0 Hz, 1H), 7.18 (dt, *J* = 8.0, 5.0 Hz, 1H), 6.01 – 5.94 (m, 1H), 5.34 (dd, *J* = 17.5, 1.5 Hz, 1H), 5.34 (dd, *J* = 10.0, 1.5 Hz, 1H), 4.65 (s, 2H), 4.13 (d, *J* = 5.5 Hz, 2H) ppm; ^{13}C{^{1}H} NMR (125 MHz, $CDCl_3$): δ 158.4, 149.0, 136.5, 134.3, 122.2, 121.2, 117.2, 72.9, 71.7 ppm. The ^{1}H NMR data for this compound match the literature data.[7]

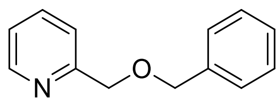

2-(Benzyloxymethyl)pyridine (1m): The reaction was performed following General Procedure C with 2-(chloromethyl)pyridine hydrochloride (2.0 g, 12 mmol), benzyl alcohol (1.3 g, 12 mmol), sodium hydroxide (1.2 g, 30 mmol) and toluene (5 mL). The reaction mixture was stirred and refluxed for 4 h. The crude product was purified by flash chromatography on silica gel (eluted with EtOAc:hexanes = 1:4) to afford the product (2.0 g, 85% yield) as a yellow oil. The ^{1}H and ^{13}C{^{1}H} NMR data for this compound match the literature data.[8]

3. Procedure and characterization of Pd-catalyzed chemoselective C(sp^3)–H arylation of 2-pyridylmethyl ethers (Table 2 and 3).

General Procedure D for the Pd-catalyzed chemoselective* C(*sp3*)–H *arylation of pyridylmethyl ethers:

An oven-dried 10 mL reaction vial equipped with a stir bar was charged with pyridylmethyl ether (**1**, 0.2 mmol, 1.0 equiv), aryl bromide (**6**, 0.24 mmol, 1.2 equiv) and dry DME (1 mL) in a glove box under a nitrogen atmosphere at room temperature. $NaN(SiMe_3)_2$ (110 mg, 0.6 mmol, 3.0 equiv) was added to the reaction mixture. A

solution (from a stock solution) of $Pd(OAc)_2$ (2.24 mg, 0.01 mmol, 5 mol %) and NIXANTPHOS (8.28 mg, 0.015 mmol, 7.5 mol %) in 1 mL of dry DME was taken up by syringe and added to the reaction vial. The vial was capped, removed from the glove box, and stirred for 12 h at room temperature (23 °C) until TLC showed complete consumption of pyridylmethyl ether. The reaction mixture was quenched with four drops of H_2O, diluted with 3 mL of ethyl acetate, and filtered over a pad of $MgSO_4$ and silica. The pad was rinsed with additional ethyl acetate (2 mL), and the solution was concentrated *in vacuo*. The crude material was loaded onto a silica gel column and purified by flash chromatography.

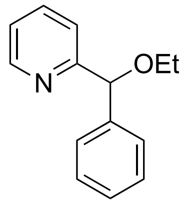

2-(Ethoxy(phenyl)methyl)pyridine (4aa): The reaction was performed following General Procedure D with 2-(ethoxymethyl)pyridine (**1a**, 27.4 mg, 28 μL, 0.2 mmol) and bromobenzene (**7a**, 25.2 μL, 0.24 mmol). The crude product was purified by flash chromatography on silica gel (eluted with EtOAc:hexanes = 10:90) to afford the product (48.5 mg, 95% yield) as a yellow oil. The ^1H and $^{13}C\{^1H\}$ NMR data for this compound match the literature data.[3]

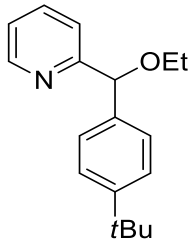

2-((4-(*tert*-Butyl)phenyl)(ethoxy)methyl)pyridine (4ab): The reaction was performed following General Procedure D with 2-(ethoxymethyl)pyridine (**1a**, 27.4 mg, 28 μL, 0.2 mmol) and 1-bromo-4-*tert*-butylbenzene (**7b**, 42.0 μL, 0.24 mmol). The crude product was purified, by flash chromatography on silica gel (eluted with EtOAc:hexanes = 10:90) to afford the product (48.4 mg, 90% yield) as a yellow oil. ^1H NMR (500 MHz,

$CDCl_3$): δ 8.52 (ddd, *J* = 5.0, 1.5, 1.0 Hz, 1H), 7.66 (ddd, *J* = 8.0, 7.5, 1.5 Hz, 1H), 7.54 (ddd, *J* = 8.0, 1.5, 1.0 Hz, 1H), 7.37 – 7.32 (m, 4H), 7.12 (ddd, *J* = 7.5, 5.0, 1.0 Hz, 1H), 5.47 (s, 1H), 3.56 (ABqq, $\Delta\nu_{AB}$ = 22.2 Hz, *J* = 9.0, 7.0 Hz, 2H), 1.28 (s, 9H), 1.27 (t, *J* = 7.0 Hz, 3H) ppm; $^{13}C\{^1H\}$ NMR (125 MHz, $CDCl_3$): δ 162.2, 150.3, 148.9, 138.3, 136.7, 126.6, 125.3, 122.2, 120.6, 84.6, 64.7, 34.4, 31.3, 15.3 ppm; IR (thin film): 2965, 2869, 1589, 1512, 1469, 1433, 1363, 1269, 1102, 993, 815, 749, 615 cm^{-1}; HRMS calculated for $C_{18}H_{24}NO$, 270.1858, found 270.1860 $[M+H]^+$.

5 mmol Scale: An oven-dried 100 mL round bottom flask equipped with a stir bar was charged with 2-(ethoxymethyl)pyridine (**1a**, 686 mg, 5.0 mmol), 1-bromo-4-*tert*-butylbenzene (**7b**, 1.04 mL, 6.0 mmol) and DME (25 mL) in a glove box under a nitrogen atmosphere at room temperature. To the mixture was added $NaN(SiMe_3)_2$ (2.75 g, 15 mmol) and the flask was stirred for 10 min. In a separate 50 mL round bottomed flask equipped with a stir bar was added $Pd(OAc)_2$ (41.5 mg, 0.185 mmol) and NIXANTPHOS (154 mg, 0.28 mmol) in DME (20 mL). The Pd/NIXANTPHOS solution was stirred for 10 min and was transferred to the reaction flask by a syringe and the flask was rinsed with additional DME (5 mL). The flask was capped with a rubber septum, removed from the glove box, and stirred for 12 h at room temperature (23 °C) until TLC showed complete consumption of **1a**. The reaction mixture was quenched with H_2O (300 μL), diluted with 30 mL of ethyl acetate, and filtered over a pad of celite. The pad was rinsed with additional ethyl acetate (60 mL) and the solution was concentrated *in vacuo*. The crude material was loaded onto a silica gel column and purified by flash chromatography (eluted with EtOAc:hexanes = 5:95) to afford the product **4ab** (1.12 g, 83%) yield as a yellow oil.

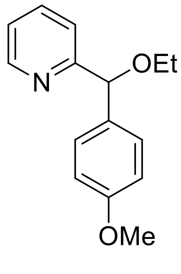

2-(Ethoxy(4-methoxyphenyl)methyl)pyridine (4ac): The reaction was

performed following General Procedure D with 2-(ethoxymethyl)pyridine (**1a**, 27.4 mg, 28 μL, 0.2 mmol) and 4-bromoanisole (**7c**, 30.0 μL, 0.24 mmol). The crude product was purified by flash chromatography on silica gel (eluted with EtOAc:hexanes = 10:90) to afford the product (42.7 mg, 88% yield) as a yellow oil. ^{1}H NMR (500 MHz, $CDCl_3$): δ 8.51 (ddd, *J* = 5.0, 1.5, 1.0 Hz, 1H), 7.66 (td, *J* = 7.5, 1.5 Hz, 1H), 7.53 (ddd, *J* = 7.5, 1.5, 1.0 Hz, 1H), 7.37 – 7.32 (m, 2H), 7.12 (ddd, *J* = 7.5, 5.0, 1.0 Hz, 1H), 6.85 (app. d, *J* = 9.0 Hz, 2H), 5.44 (s, 1H), 3.76 (s, 3H), 3.57 (ABqq, $\Delta\nu_{AB}$ = 18.4 Hz, *J* = 9.0, 7.0 Hz, 2H) 1.27 (t, *J* = 7.0 Hz, 3H) ppm; $^{13}C\{^{1}H\}$ NMR (125 MHz, $CDCl_3$): δ 162.3, 159.0, 149.0, 136.7, 133.5, 128.2, 122.1, 120.4, 113.8, 84.2, 64.6, 55.2, 15.3 ppm; IR (thin film): 2973, 1610, 1588, 1510, 1468, 1433, 1302, 1247, 1171, 1098, 1034, 791, 752 cm^{-1}; HRMS calculated for $C_{15}H_{18}NO_2$, 244.1338, found 244.1336 $[M+H]^+$.

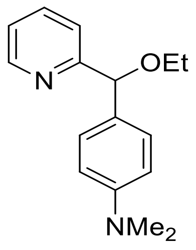

4-(Ethoxy(pyridin-2-yl)methyl)-N,N-dimethylaniline (4ad): The reaction was performed following General Procedure D with 2-(ethoxymethyl)pyridine (**1a**, 27.4 mg, 28 μL, 0.2 mmol) and 4-bromo-*N,N*-dimethylaniline (**7d**, 48.0 mg, 0.24 mmol). The crude product was purified by flash chromatography on silica gel (eluted with EtOAc:hexanes = 20:80) to afford the product (50.6 mg, 99% yield) as a yellow solid. m.p. = 55 – 57 °C; ^{1}H NMR (500 MHz, $CDCl_3$): δ 8.51 (ddd, *J* = 5.0, 1.5, 1.0 Hz, 1H), 7.64 (ddd, *J* = 8.0, 7.5, 1.5 Hz, 1H), 7.53 (ddd, *J* = 8.0, 1.5, 1.0 Hz, 1H), 7.32 – 7.22 (m, 2H), 7.10 (ddd, *J* = 7.5, 5.0, 1.0 Hz, 1H), 6.69 (app. d, *J* = 8.5 Hz, 2H), 5.41 (s, 1H), 3.55 (ABqq, $\Delta\nu_{AB}$ = 26.2 Hz, *J* = 9.5, 7.0 Hz, 2H), 2.90 (s, 6H), 1.27 (t, *J* = 7.0 Hz, 3H) ppm; $^{13}C\{^{1}H\}$ NMR (125 MHz, $CDCl_3$): δ 162.6, 150.1, 148.9, 136.6, 129.1, 127.9, 121.9, 120.4, 112.4, 84.5, 64.4, 40.5, 15.4 ppm; IR (thin film): 1613, 1588, 1433, 1346, 1162, 1098, 806 cm^{-1}; HRMS calculated for $C_{16}H_{21}N_2O$, 257.1654, found 257.1655

[M+H]$^+$.

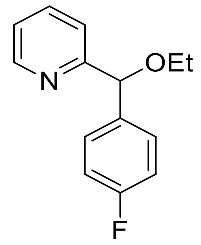

2-(Ethoxy(4-fluorophenyl)methyl)pyridine (4ae): The reaction was performed following General Procedure D with 2-(ethoxymethyl)pyridine (**1a**, 27.4 mg, 28 μL, 0.2 mmol) and 1-bromo-4-fluorobenzene (**7e**, 31.2 μL, 0.24 mmol). The crude product was purified by flash chromatography on silica gel (eluted with EtOAc:hexanes = 10:90) to afford the product (37.8 mg, 82% yield) as a yellow oil. ^{1}H NMR (500 MHz, $CDCl_3$): δ 8.50 (ddd, J = 5.0, 1.5, 1.0 Hz, 1H), 7.66 (td, J = 8.0, 1.5 Hz, 1H), 7.49 (d, J = 8.0 Hz, 1H), 7.37 (dd, J = 8.5, 5.0 Hz, 2H), 7.12 (ddd, J = 8.0, 5.0, 1.0 Hz, 1H), 6.97 (app. t, J = 8.5 Hz, 2H), 5.44 (s, 1H), 3.54 (m, 2H), 1.25 (t, J = 7.0 Hz, 3H) ppm; ^{13}C{^{1}H} NMR (125 MHz, $CDCl_3$): δ 162.1 (d, J = 250.0 Hz), 161.8, 148.9, 137.1 (d, J = 3.2 Hz), 136.7, 128.5 (d, J = 12.5 Hz), 122.2, 120.3, 115.1 (d, J = 25.0 Hz), 83.8, 64.7, 15.2 ppm; IR (thin film): 2974, 1609, 1588, 1433, 1337, 1104, 699 cm^{-1}; HRMS calculated for $C_{14}H_{15}FNO$, 232.1138, found 232.1141 [M+H]$^+$.

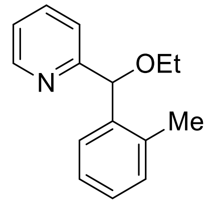

2-(Ethoxy(o-tolyl)methyl)pyridine (4af): The reaction was performed following General Procedure D with 2-(ethoxymethyl)pyridine (**1a**, 27.4 mg, 28 μL, 0.2 mmol) and 1-bromo-2-methylbenzene (**7f**, 28.8 μL, 0.24 mmol). The crude product was purified by flash chromatography on silica gel (eluted with EtOAc:hexanes = 10:90) to afford the product (34.0 mg, 75% yield) as a yellow oil. ^{1}H NMR (500 MHz, $CDCl_3$): δ 8.50 (ddd, J = 5.0, 1.5, 1.0 Hz, 1H), 7.64 (ddd, J = 8.0, 7.5, 1.5 Hz, 1H), 7.43 (app. d, J = 9.0 Hz, 1H), 7.41 (dd, J = 7.5, 1.5 Hz, 1H), 7.20 –

7.12 (m, 4H), 5.68 (s, 1H), 3.58 (ABqq, $\Delta\nu_{AB}$ = 30.6 Hz, *J* = 9.0, 7.0 Hz, 2H), 2.35 (s, 3H), 1.27 (t, *J* = 7.0 Hz, 3H) ppm; $^{13}C\{^1H\}$ NMR (125 MHz, $CDCl_3$): δ 161.5, 148.9, 139.3, 136.6, 136.3, 130.5, 127.5, 126.6, 126.0, 122.2, 121.4, 82.0, 64.9, 19.6, 15.4 ppm; IR (thin film): 2973, 1589, 1571, 1467, 1433, 1113, 1083, 993, 750, 723, 661cm^{-1}; HRMS calculated for $C_{15}H_{18}NO$, 228.1388, found 228.1390 $[M+H]^+$.

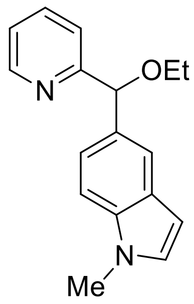

5-(Ethoxy(pyridin-2-yl)methyl)-1-methyl-1*H*-indole (4ag): The reaction was performed following General Procedure D with 2-(ethoxymethyl)pyridine (**1a**, 28 μL, 0.2 mmol) and 5-bromo-1-methyl-1*H*-indole (**7g**, 50.4 mg, 0.24 mmol). The crude product was purified by flash chromatography on silica gel (eluted with EtOAc:hexanes = 25:75) to afford the product (43.6 mg, 82% yield) as a white solid. m.p. = 163 – 165 °C; ^1H NMR (500 MHz, $CDCl_3$): δ 8.53 (d, *J* = 4.5 Hz, 1H), 7.72 (s, 1H), 7.65 (d, *J* = 7.5 Hz, 1H), 7.60 (d, *J* = 7.5 Hz, 1H), 7.31 (d, *J* = 8.5 Hz, 1H), 7.27 (d, *J* = 8.5 Hz, 1H), 7.11 (dd, *J* = 7.5, 4.5 Hz, 1H), 7.02 (d, *J* = 3.0 Hz, 1H), 6.46 (d, *J* = 3.0 Hz, 1H), 5.62 (s, 1H), 3.73 (s, 3H), 3.61 (m, 2H), 1.31 (t, *J* = 7.2 Hz, 3H) ppm; $^{13}C\{^1H\}$ NMR (125 MHz, $CDCl_3$): δ 162.9, 148.9, 136.6, 136.3, 132.2, 129.1, 128.4, 121.9, 120.8, 120.5, 119.6, 109.2, 101.1, 85.3, 64.5, 32.8, 15.4 ppm; IR (thin film): 2972, 2927, 2872, 1589, 1570, 1433, 1326, 1100, 721 cm^{-1}; HRMS calculated for $C_{17}H_{19}N_2O$, 267.1497 found 267.1499 $[M+H]^+$.

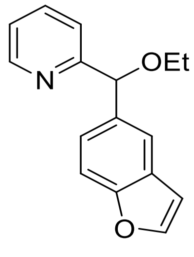

2-(Benzofuran-5-yl(ethoxy)methyl)pyridine (4ah): The reaction was

performed following General Procedure D with 2-(ethoxymethyl)pyridine (**1a**, 27.4 mg, 28 μL, 0.2 mmol) and 5-bromobenzofuran (**7h**, 30.1 μL, 47.3 mg, 0.24 mmol). The crude product was purified by flash chromatography on silica gel (eluted with EtOAc:hexanes = 25:75) to afford the product (42.0 mg, 82% yield) as a white solid. m.p. = 158 – 160 °C; ^{1}H NMR (500 MHz, $CDCl_3$): δ 8.53 (d, J = 5.0 Hz, 1H), 7.68 (s, 1H), 7.67 (d, J = 8.0 Hz, 1H), 7.59 – 7.57 (m, 2H), 7.44 (d, J = 8.5 Hz, 1H), 7.37 (d, J = 8.5 Hz, 1H), 7.12 (dd, J = 8.0, 4.5 Hz, 1H), 6.73 (s, 1H), 5.59 (s, 1H), 3.65 – 3.59 (m, 2H), 1.29 (t, J = 7.2 Hz, 3H) ppm; ^{13}C{^{1}H} NMR (125 MHz, $CDCl_3$): δ 162.2, 154.5, 149.0, 145.3, 136.8, 136.0, 127.5, 123.4, 122.2, 120.5, 119.7, 111.3, 106.7, 84.7, 64.7, 15.3 ppm; IR (thin film): 2974, 2871, 1588, 1467, 1434, 1263, 1107, 1031, 884, 754, 676 cm^{-1}; HRMS calculated for $C_{16}H_{16}NO_2$, 254.1181 found 254.1186 [M+H]$^+$.

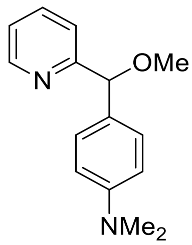

4-(Methoxy(pyridin-2-yl)methyl)-*N*,*N*-dimethylaniline (4bd): The reaction was performed following General Procedure D with 2-(methoxymethyl)pyridine (**1b**, 24.6 mg, 0.2 mmol) and 4-bromo-*N*,*N*-dimethylaniline (**7d**, 48.0 mg, 0.24 mmol). The crude product was purified by flash chromatography on silica gel (eluted with EtOAc:hexanes = 10:90) to afford the product (44.7 mg, 92% yield) as a yellow oil. ^{1}H NMR (500 MHz, $CDCl_3$): δ 8.50 (ddd, J = 5.0, 1.5, 1.0 Hz, 1H), 7.63 (ddd, J = 8.0, 7.5, 1.5 Hz, 1H), 7.47 (ddd, J = 8.0, 1.5, 1.0 Hz, 1H), 7.25 (d, J = 9.0 Hz, 2H), 7.09 (ddd J = 7.5, 5.0, 1.0 Hz, 1H), 6.76 (d, J = 9.0 Hz, 2H), 5.27 (s, 1H), 3.38 (s, 3H), 2.89 (s, 6H) ppm; ^{13}C{^{1}H} NMR (125 MHz, $CDCl_3$): δ 162.1, 150.1, 148.9, 136.5, 128.4, 127.9, 121.9, 120.3, 112.3, 86.2, 56.8, 40.4 ppm; IR (thin film): 2928, 2819, 1613, 1521, 1470, 1432, 1347, 1163, 1097, 946, 809, 615 cm^{-1}; HRMS calculated for $C_{15}H_{19}N_2O$, 243.1497 found 243.1499 [M+H]$^+$.

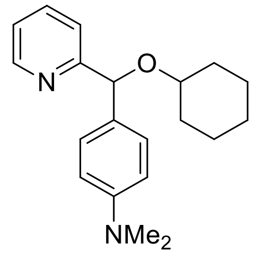

4-((Cyclohexyloxy)(pyridin-2-yl)methyl)-*N*,*N*-dimethylaniline (4cd): The reaction was performed following General Procedure D with 2-((cyclohexyloxy)methyl)pyridine (**1c**, 38.2 mg, 0.2 mmol) and 4-bromo-*N,N*-dimethylaniline (**7d**, 48.0 mg, 0.24 mmol). The crude product was purified by flash chromatography on silica gel (eluted with EtOAc:hexanes = 15:85) to afford the product (51.5 mg, 83% yield) as a yellow solid. m.p. = 81 – 83 °C . ^{1}H NMR (500 MHz, $CDCl_3$): δ 8.50 (ddd, *J* = 5.0, 1.5, 1.0 Hz, 1H), 7.64 (ddd, *J* = 8.0, 7.5, 1.5 Hz, 1H), 7.59 (ddd, *J* = 8.0, 1.5, 1.0 Hz, 1H), 7.28 (d, *J* = 8.5 Hz, 2H), 7.10 (ddd, *J* = 7.5, 5.0, 1.0 Hz, 1H), 6.68 (d, *J* = 8.5 Hz, 2H), 5.60 (s, 1H), 3.42–3.37 (m, 1H), 2.90 (s, 6H), 1.94 – 1.92 (m, 2H), 1.76 – 1.72 (m, 2H), 1.45 – 1.40 (m, 3H), 1.18 – 1.12 (m, 3H) ppm; $^{13}C\{^1H\}$ NMR (125 MHz, $CDCl_3$): δ 163.5, 149.9, 148.7, 136.6, 129.9, 127.9, 121.8, 120.6, 112.4, 81.1, 75.0, 40.6, 32.6, 25.9, 24.2 ppm; IR (thin film): 2930, 2855, 1613, 1520, 1433, 1343, 1162, 1074, 947, 809 cm^{-1}; HRMS calculated for $C_{20}H_{27}N_2O$, 311.2123 found 311.2128 $[M+H]^+$.

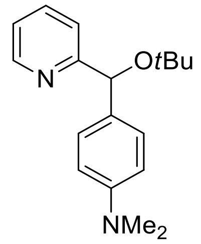

4-(*tert*-Butoxy(pyridin-2-yl)methyl)-*N*,*N*-dimethylaniline (4dd): The reaction was performed following General Procedure D with 2-(*tert*-butoxymethyl)pyridine (**1d**, 33.4 mg, 0.2 mmol) and 4-bromo-*N,N*-dimethylaniline (**7d**, 48.0 mg, 0.24 mmol). The crude product was purified by flash chromatography on silica gel (eluted with EtOAc:hexanes = 15:85) to afford the product (50.0 mg, 88% yield) as a yellow solid. m.p. = 65 – 66 °C. ^{1}H

NMR (500 MHz, $CDCl_3$): δ 8.45 (ddd, *J* = 5.0, 1.5, 1.0 Hz, 1H), 7.59 (br. s, 2H), 7.27 (app. d, *J* = 8.5 Hz, 2H), 7.05 (ddd, *J* = 6.6, 4.7, 2.0 Hz, 1H), 6.65 (d, *J* = 8.5 Hz, 2H), 5.65 (s, 1H), 2.87 (s, 6H), 1.21 (s, 9H) ppm; $^{13}C\{^1H\}$ NMR (125 MHz, $CDCl_3$): δ 165.2, 149.5, 148.3, 136.3, 131.8, 127.4, 121.4, 120.9, 112.3, 76.9, 74.8, 40.5, 28.6 ppm; IR (thin film): 2973, 1613, 1589, 1520, 1470, 1346, 1162, 1063, 1022, 947, 808, 749, 616 cm^{-1}; HRMS calculated for $C_{18}H_{25}N_2O$, 285.1967 found 285.1970 $[M+H]^+$.

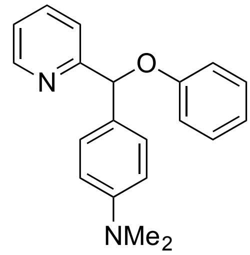

***N,N*-Dimethyl-4-(phenoxy(pyridin-2-yl)methyl)aniline (4ed):** The reaction was performed following General Procedure D with 2-(phenoxymethyl)pyridine (**1e**, 37.0 mg, 0.2 mmol) and 4-bromo-*N,N*-dimethylaniline (**7d**, 48.0 mg, 0.24 mmol). The crude product was purified by flash chromatography on silica gel (eluted with EtOAc:hexanes = 15:85) to afford the product (49.8 mg, 82% yield) as a white solid. m.p. = 84 – 86 °C; ^1H NMR (500 MHz, $CDCl_3$): δ 8.55 (d, *J* = 4.5 Hz, 1H), 7.62 (d, *J* = 8.0 Hz, 1H), 7.57 (d, *J* = 8.0 Hz, 1H), 7.35 (d, *J* = 8.5 Hz, 2H), 7.20 (d, *J* = 8.5 Hz, 2H), 7.12 (dd, *J* = 8.0, 4.5 Hz, 1H), 6.98 (d, *J* = 8.5 Hz, 2H), 6.88 (t, *J* = 8.5 Hz, 1H), 6.68 (d, *J* = 8.5 Hz, 2H), 6.27 (s, 1H), 2.90 (s, 6H) ppm; $^{13}C\{^1H\}$ NMR (125 MHz, $CDCl_3$): δ 161.3, 157.9, 150.1, 149.1, 136.8, 129.2, 127.9, 127.7, 122.2, 120.7, 120.5, 115.9, 112.4, 82.4, 40.4 ppm; IR (thin film): 3402, 2974, 2886, 1613, 1599, 1521, 1445, 1410, 1351, 1163, 1095, 796 cm^{-1}; HRMS calculated for $C_{20}H_{21}N_2O$, 305.1654 found 305.1658 $[M+H]^+$.

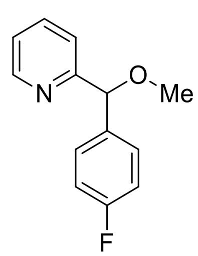

2-((4-Fluorophenyl)(methoxy)methyl)pyridine (4be): The reaction was performed following General Procedure D with 2-pyridinylmethyl methyl ether (**1b**, 24.6 mg, 0.2 mmol) and 1-bromo-4-fluorobenzene (**7e**, 31.2 μL, 42 mg, 0.24 mmol). The crude product was purified by flash chromatography on silica gel (eluted with EtOAc:hexanes = 10:90) to afford the product (36.8 mg, 85% yield) as a yellow oil. ^{1}H NMR (500 MHz, $CDCl_3$): δ 8.53 (ddd, *J* = 5.0, 1.5, 1.0 Hz, 1H), 7.66 (td, *J* = 8.0, 7.5, 1.5 Hz, 1H), 7.47 (ddd, *J* = 8.0, 1.5, 1.0 Hz, 1H), 7.38 (dd, *J* = 9.0, 5.5 Hz, 2H), 7.14 (ddd, *J* = 7.5, 5.0, 1.0 Hz, 1H), 6.99 (app. t, *J* = 9.0 Hz, 2H), 5.34 (s, 1H), 3.40 (s, 3H) ppm; ^{13}C{^{1}H} NMR (125 MHz, $CDCl_3$): δ 162.2 (d, *J* = 250.0 Hz), 161.3, 149.1, 136.8, 136.7 (d, *J* = 2.5 Hz), 128.6 (d, *J* = 12.5 Hz), 122.4, 120.4, 115.3 (d, *J* = 25.0 Hz), 83.7, 57.1 ppm; IR (thin film): 2934, 2825, 1604, 1589, 1507, 1471, 1434, 1221, 1194, 1156, 1105, 1091, 804, cm^{-1}; HRMS calculated for $C_{13}H_{11}FNO$, 216.0825, found 216.0822 $[M{-}H]^+$.

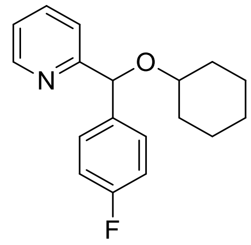

2-((Cyclohexyloxy)(4-fluorophenyl)methyl)pyridine (4ce): The reaction was performed following General Procedure D with 2-((cyclohexyloxy)methyl)pyridine (**1c**, 38.2 mg, 0.2 mmol) and 1-bromo-4-fluorobenzene (**7e**, 31.2 μL, 42 mg, 0.24 mmol). The crude product was purified by flash chromatography on silica gel (eluted with EtOAc:hexanes = 15:85) to afford the product (50.1 mg, 88% yield) as a yellow solid. m.p. = 82 – 84 °C; ^{1}H NMR (500 MHz, $CDCl_3$): d 8.50 (ddd, *J* = 5.0, 1.5, 1.0 Hz, 1H), 7.66 (td, *J* = 8.0, 7.5, 1.5 Hz, 1H), 7.56 (ddd, *J* = 7.5, 1.5, 1.0 Hz, 1H), 7.38 (app. d, *J* = 8.5, 5.0 Hz,

2H), 7.14 – 7.11 (m, 1H), 6.97 (app. t, *J* = 8.5 Hz, 2H), 5.62 (s, 1H), 3.38 (m, 1H), 1.94 – 1.87 (m, 2H), 1.74 – 1.72 (m, 2H), 1.49 – 1.41 (m, 3H), 1.24 – 1.21 (m, 3H) ppm; $^{13}C\{^{1}H\}$ NMR (125 MHz, $CDCl_3$): d 162.7, 162.2 (d, *J* = 250.0 Hz), 148.8, 137.9 (d, *J* = 2.5 Hz), 136.8, 128.5 (d, *J* = 12.5 Hz), 122.2, 120.6, 115.1 (d, *J* = 25.0 Hz), 80.7, 75.6, 32.4, 25.7, 24.0 ppm; IR (thin film): 3009, 2932, 2857, 1604, 1589, 1507, 1434, 1220, 1155, 1077, 959, 724 cm^{-1}; HRMS calculated for $C_{18}H_{21}FNO$, 286.1607, found 286.1611 $[M+H]^+$.

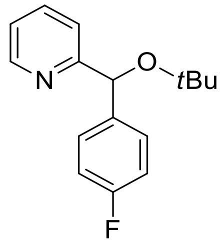

2-(*tert*-Butoxy(4-fluorophenyl)methyl)pyridine (4de): The reaction was performed following General Procedure D with 2-(*tert*-Butoxymethyl)pyridine (**7d**, 33.0 mg, 0.2 mmol) and 1-bromo-4-fluorobenzene (**6e**, 31.2 μL, 0.24 mmol). The crude product was purified by flash chromatography on silica gel (eluted with EtOAc:hexanes = 15:85) to afford the product (41.4 mg, 80% yield) as a yellow oil. ^{1}H NMR (500 MHz, $CDCl_3$): δ 8.47 (ddd, *J* = 5.0, 2.0, 1.0 Hz, 1H), 7.64 (td, *J* = 8.0, 2.0 Hz, 1H), 7.57 (d, *J* = 8.0, 2.0, 1.0 Hz, 1H), 7.40 (app. dd, *J* = 8.5, 5.0 Hz, 2H), 7.11 – 7.09 (m, 1H), 6.95 (app. t, *J* = 8.5 Hz, 2H), 5.69 (s, 1H), 1.22 (s, 9H) ppm; $^{13}C\{^{1}H\}$ NMR (125 MHz, $CDCl_3$): δ 164.4, 161.9 (d, *J* = 250.0 Hz), 148.5, 139.6 (d, *J* = 2.5 Hz), 136.6, 128.2 (d, *J* = 12.5 Hz), 121.9, 120.9, 115.3 (d, *J* = 25.0 Hz), 76.4, 75.4, 28.5 ppm; IR (thin film): 3054, 2975, 1932, 1604, 1589, 1507, 1469, 1434, 1390, 1367, 1220, 1155, 1102, 1072, 819, 804, 750, 650 cm^{-1}; HRMS calculated for $C_{16}H_{19}FNO$, 260.1451, found 260.1451 $[M–H]^+$.

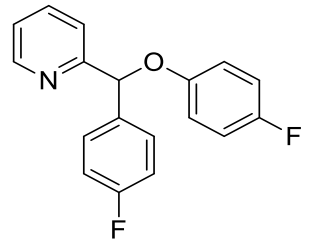

2-((4-Fluorophenoxy)(4-fluorophenyl)methyl)pyridine (4fe): The reaction was performed following General Procedure D with 2-((4-fluorophenoxy)methyl)pyridine (**1f**, 40.6 mg, 0.2 mmol) and 1-bromo-4-fluorobenzene (**7e**, 31.2 μL, 42 mg, 0.24 mmol). The crude product was purified by flash chromatography on silica gel (eluted with EtOAc:hexanes = 15:85) to afford the product (48.7 mg, 82% yield) as a yellow oil. ^{1}H NMR (500 MHz, $CDCl_3$): δ 8.58 (ddd, J = 5.0, 1.5, 1.0 Hz, 1H), 7.68 (td, J = 8.0, 1.5 Hz, 1H), 7.54 (d, J = 8.0 Hz, 1H), 7.49–7.41 (m, 2H), 7.20 – 7.17 (m, 1H), 7.02 (t, J = 8.5 Hz, 2H), 6.92 – 6.90 (m, 4H), 6.25 (s, 1H) ppm; ^{13}C{^{1}H} NMR (125 MHz, $CDCl_3$): δ 162.4 (d, J = 250.0 Hz), 161.4, 157.5 (d, J = 250.0 Hz), 153.6 (d, J = 2.5 Hz), 149.2, 137.2, 135.8 (d, J = 2.5 Hz), 128.5 (d, J = 12.5 Hz), 122.8, 120.6, 117.1 (d, J = 7.5 Hz), 115.9 (d, J = 25.0Hz), 115.5 (d, J = 25.0 Hz), 82.6 ppm; IR (thin film): 3055, 2918, 1604, 1589, 1504, 1471, 1435, 1220, 1205, 1157, 1097, 1048, 827, 774, 614 cm^{-1}; HRMS calculated for $C_{18}H_{14}F_2NO$, 298.1043, found 298.1039 $[M+H]^+$.

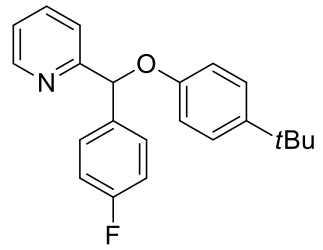

2-((4-(*tert*-Butyl)phenoxy)(4-fluorophenyl)methyl)pyridine (4ge): The reaction was performed following General Procedure D with 2-((4-(*tert*-butyl)phenoxy)methyl)pyridine (**1g**, 48.2 mg, 0.2 mmol) and 1-bromo-4-fluorobenzene (**7e**, 31.2 μL, 42 mg, 0.24 mmol). The crude product was purified by flash chromatography on silica gel (eluted with EtOAc:hexanes = 15:85) to afford the product (53.6 mg, 80% yield) as a yellow oil. ^{1}H NMR (500 MHz,

$CDCl_3$): δ 8.59 (ddd, *J* = 5.0, 1.5, 1.0 Hz, 1H), 7.68 (dt, *J* = 7.5, 1.5 Hz, 1H), 7.60 (d, *J* = 8.0 Hz, 1H), 7.51 (dd, *J* = 8.5, 5.5 Hz, 2H), 7.27 (d, *J* = 9.0 Hz, 2H), 7.18 (ddd, *J* = 7.5, 5.0, 1.0 Hz, 1H), 7.03 (app. t, *J* = 8.5 Hz, 2H), 6.91 (d, *J* = 9.0 Hz, 2H), 6.32 (s, 1H), 1.28 (s, 9H) ppm; $^{13}C\{^1H\}$ NMR (125 MHz, $CDCl_3$): δ 162.3 (d, *J* = 250.0 Hz), 160.8, 155.3, 149.1, 143.9, 137.2, 136.2 (d, *J* = 2.5 Hz), 128.5 (d, *J* = 12.5 Hz), 126.2, 122.7, 120.7, 115.5 (d, *J* = 25.0 Hz), 115.3, 81.8, 34.0, 31.5 ppm; IR (thin film): 2961, 1606, 1509, 1471, 1435, 1363, 1295, 1232, 1182, 1157, 1100, 1046, 828, 754 cm^{-1}; HRMS calculated for $C_{22}H_{23}FNO$, 336.1764, found 336.1762 $[M+H]^+$.

4. Procedure and characterization of Pd-catalyzed chemoselective tandem arylation/[1,2]-Wittig rearrangement reaction of 2-pyridylmethyl ethers (Table 4 and 5).

General Procedure E for the Pd-catalyzed chemoselective tandem arylation/*[1,2]*-Wittig rearrangement reaction of 2-pyridylmethyl ethers:

An oven-dried 10 mL reaction vial equipped with a stir bar was charged with pyridinylmethyl ether (**1**, 0.20 mmol, 1.0 equiv) and aryl bromide (**6**, 0.24 mmol, 1.2 equiv) and CPME (1 mL) in a glove box under a nitrogen atmosphere at room temperature. $LiN(SiMe_3)_2$ (100 mg, 0.6 mmol, 3.0 equiv) was added to the reaction mixture followed by a solution (from a stock solution) of $Pd(OAc)_2$ (1.12 mg, 0.05 mmol, 2.5 mol%) and NIXANTPHOS (4.14 mg, 0.075 mmol, 3.75 mol%) in 1.0 mL of dry CPME was taken up by syringe and added to the reaction vial. The vial was capped, removed from the glove box, and stirred for 24 h at 45 °C until TLC showed complete consumption of pyridylmethyl ether starting material and arylation intermediate. The reaction mixture was quenched with four drops of H_2O, diluted with 3 mL of ethyl acetate, and filtered over a pad of $MgSO_4$ and silica. The pad was rinsed with additional ethyl acetate and the solution was concentrated *in vacuo*. The crude material was loaded onto a silica gel column and purified by flash chromatography.

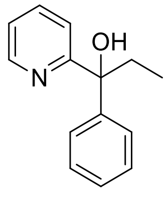

1-Phenyl-1-(pyridin-2-yl)propan-1-ol (5aa): The reaction was performed following General Procedure E with 2-(ethoxymethyl)pyridine (**1a**, 27.4 mg, 28 μL, 0.2 mmol) and bromobenzene (**7a**, 25.2 μL, 0.24 mmol). The crude product was purified by flash chromatography on silica gel (eluted with EtOAc:hexanes = 30:70) to afford the product (36.2 mg, 85% yield) as a white solid. m.p. = 72 – 74 °C; The ^{1}H and $^{13}C\{^{1}H\}$ NMR data for this compound match the literature data.[9]

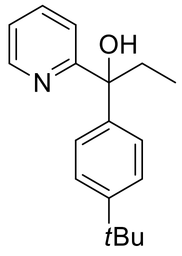

1-(4-(*tert*-Butyl)phenyl)-1-(pyridin-2-yl)propan-1-ol (5ab): The reaction was performed following General Procedure E with 2-(ethoxymethyl)pyridine (**1a,** 27.4 mg, 28 μL, 0.2 mmol) and 1-bromo-4-*tert*-butylbenzene (**7b**, 42.0 μL, 0.24 mmol). The crude product was purified by flash chromatography on silica gel (eluted with EtOAc:hexanes = 20:80) to afford the product (41.9 mg, 78% yield) as a white solid. m.p. = 45 – 47 °C; ^{1}H NMR (500 MHz, $CDCl_3$): δ 8.50 (ddd, *J* = 5.0, 1.5, 1.0 Hz, 1H), 7.64 (td, *J* = 8.0, 7.5, 1.5 Hz, 1H), 7.44 (d, *J* = 8.5 Hz, 2H), 7.35 – 7.31 (m, 3H), 7.15 (ddd, *J* = 7.5, 5.0, 1.0 Hz, 1H), 5.89 (s, 1H), 2.30 (ABqq, $\Delta\nu_{AB}$ = 37.8 Hz, *J* = 14.5, 7.5 Hz, 2H), 1.28 (s, 9H), 0.85 (t, *J* = 7.5 Hz, 3H) ppm; $^{13}C\{^{1}H\}$ NMR (125 MHz, $CDCl_3$): δ 163.7, 149.5, 147.1, 143.4, 136.8, 125.6, 125.0, 121.8, 120.6, 77.2, 34.3, 33.9, 31.3, 8.0 ppm; IR (thin film): 3367, 2964, 1592, 1509, 1467, 1433, 1363, 1269, 1113, 985, 829, 751, 597 cm^{-1}; HRMS calculated for $C_{18}H_{24}NO$, 270.1858, found 270.1845 $[M+H]^{+}$.

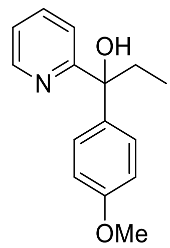

1-(4-Methoxyphenyl)-1-(pyridin-2-yl)propan-1-ol (5ac): The reaction was performed following General Procedure E with 2-(ethoxymethyl)pyridine (**1a**, 27.4 mg, 28 μL, 0.2 mmol) and 4-bromoanisole (**7c**, 30.0 μL, 0.24 mmol). The crude product was purified by flash chromatography on silica gel (eluted with EtOAc:hexanes = 20:80) to afford the product (36.4 mg, 75% yield) as a yellow solid. m.p. = 77 – 78 °C; ^{1}H NMR (500 MHz, $CDCl_3$): δ 8.50 (ddd, *J* = 4.9, 1.5, 0.9 Hz, 1H), 7.63 (td, *J* = 7.8, 1.5 Hz, 1H), 7.43 (app. d, *J* = 9.0 Hz, 2H), 7.28 (dt, *J* = 7.8, 0.9 Hz, 1H), 7.15 (ddd, *J* = 7.8, 4.9, 0.9 Hz, 1H), 6.84 (d, *J* = 9.0 Hz, 2H), 5.88 (s, 1H), 3.77 (s, 3H), 2.28 (ABqq, $\Delta\nu_{AB}$ = 44.7 Hz, *J* = 14.5, 7.5 Hz, 2H), 0.85 (t, *J* = 7.5 Hz, 3H) ppm; ^{13}C{^{1}H} NMR (125 MHz, $CDCl_3$): δ 163.9, 158.4, 147.1, 138.6, 136.9, 127.2, 121.8, 120.4, 113.5, 77.0, 55.2, 33.9, 8.0 ppm; IR (thin film): 3446, 2964, 2962, 1594, 1569, 1468, 1434, 1304, 1248, 1030, 985, 841, 591 cm^{-1}; HRMS calculated for $C_{15}H_{18}NO_2$, 244.1338, found 244.1330 [M+H]$^{+}$.

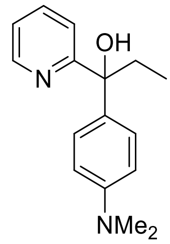

1-(4-(Dimethylamino)phenyl)-1-(pyridin-2-yl)propan-1-ol (5ad): The reaction was performed following General Procedure E with 2-(ethoxymethyl)pyridine (**1a**, 27.4 mg, 28 μL, 0.2 mmol) and 4-bromo-*N,N*-dimethylaniline (**7d**, 48.0 mg, 0.24 mmol). The crude product was purified by flash chromatography on silica gel (eluted with EtOAc:hexanes = 25:75) to afford the product (36.8 mg, 72% yield) as a yellow solid. m.p. = 108 – 110 °C; ^{1}H NMR (500 MHz, $CDCl_3$): δ 8.48 (ddd, *J* = 4.5, 1.7, 1.0 Hz, 1H), 7.59 (ddd, *J* = 8.0, 7.7, 1.7 Hz, 1H), 7.35 (app. d, *J* = 8.8 Hz, 2H), 7.29 (app. d, *J* = 8.0, 1.5, 1.0 Hz, 1H),

7.11 (ddd, J = 7.7, 5.0, 1.0 Hz, 1H), 6.66 (app. d, J = 9.0 Hz, 2H), 5.78 (s, 1H), 2.89 (s, 6H), 2.26 (ABqq, $\Delta\nu_{AB}$ = 49.2 Hz, J = 14.3, 7.3 Hz, 2H), 0.84 (t, J = 7.3 Hz, 3H) ppm; $^{13}C\{^{1}H\}$ NMR (125 MHz, $CDCl_3$): δ 164.2, 149.3, 146.9, 136.6, 134.3, 126.7, 121.5, 120.4, 112.1, 76.7, 40.4, 33.7, 7.9 ppm; IR (thin film): 2924, 1611, 1519, 1432, 1351, 1162, 816 cm^{-1}; HRMS calculated for $C_{16}H_{19}N_2$, 239.1548, found 239.1545 $[M-OH]^+$.

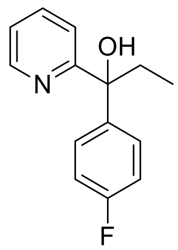

1-(4-Fluorophenyl)-1-(pyridin-2-yl)propan-1-ol (5ae): The reaction was performed following General Procedure E with 2-(ethoxymethyl)pyridine (**1a**, 27.4 mg, 28 μL, 0.2 mmol) and 1-bromo-4-fluorobenzene (**7e**, 31.2 μL, 0.24 mmol). The crude product was purified by flash chromatography on silica gel (eluted with EtOAc:hexanes = 20:80) to afford the product (40.6 mg, 88% yield) as a yellow oil. ^{1}H NMR (500 MHz, $CDCl_3$): δ 8.48 (ddd, J = 4.7, 1.7, 1.0 Hz, 1H), 7.63 (ddd, J = 8.0, 7.5, 1.7 Hz, 1H), 7.47 (app. d, J = 8.5, 5.0 Hz, 2H) , 7.26 (ddd, J = 8.0, 1.7, 1.0 Hz, 1H), 7.15 (ddd, J = 7.5, 4.7, 1.0 Hz, 1H), 6.96 (app. t, J = 8.5 Hz, 2H), 5.88 (s, 1H), 2.28 (ABqq, 2H, $\Delta\nu_{AB}$ = 38.9 Hz, J = 13.5, 7.5 Hz, 2H), 0.83 (t, J = 7.5 Hz, 3H) ppm; $^{13}C\{^{1}H\}$ NMR (125 MHz, $CDCl_3$): δ 163.3, 162.6 (d, J = 250.0 Hz), 147.1, 142.1, 136.9, 127.7 (d, J = 12.5 Hz), 121.9, 120.2, 114.8 (d, J = 25.0 Hz), 76.9, 33.8, 7.8 ppm; IR (thin film): 3364, 2935, 1593, 1506, 1467, 1433, 1392, 1223, 1159, 1087, 986, 832, 750 cm^{-1}; HRMS calculated for $C_{14}H_{13}FN$, 214.1032, found 214.1037 $[M-OH]^+$.

5 mmol Scale: An oven-dried 100 mL round bottom flask equipped with a stir bar was charged with $Pd(OAc)_2$ (28.1 mg, 0.125 mmol) and NIXANTPHOS (103.7 mg, 0.188 mmol) in CPME (50 mL). The Pd/NIXANTPHOS solution was stirred for 5 min and $LiN(SiMe_3)_2$ (2.51 g, 15 mmol) was added. After stirring for 5 min at 24 °C,

2-(ethoxymethyl)pyridine (**1a**, 686 mg, 5.0 mmol) and 1-bromo-4-fluorobenzene (**7e**, 0.66 mL, 6.0 mmol) were added to the solution. The flask was capped with a rubber septum, removed from the glove box, and stirred for 9 h at 45 °C until TLC showed complete consumption of **1a**. The reaction mixture was then cooled to rt, quenched with 30 mL of H_2O, and diluted with 30 mL of ethyl acetate. The organic layer was separated and the aqueous solution was extracted with 20 mL of EtOAc. The combined organic solution was washed with saturated aqueous $NaHCO_3$ (30 mL) and brine (30 mL). The organic solution was dried over $MgSO_4$, filtered, and the solvent was removed under reduced pressure. The crude material was loaded onto a silica gel column and purified by flash chromatography (eluted with EtOAc:hexanes = 5:95) to afford the product **5ae** (856 mg, 74%) yield as a yellow oil.

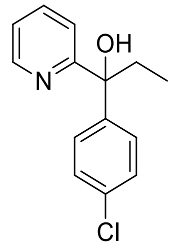

1-(4-Chlorophenyl)-1-(pyridin-2-yl)propan-1-ol (5ai): The reaction was performed following General Procedure E with 2-(ethoxymethyl)pyridine (**1a**, 27.4 mg, 28 μL, 0.2 mmol) and 1-bromo-4-chlorobenzene (**7i**, 45.8 mg, 0.24 mmol). The crude product was purified by flash chromatography on silica gel (eluted with EtOAc:hexanes = 20:80) to afford the product (41.0 mg, 83% yield) as a yellow oil. ^{1}H NMR (500 MHz, $CDCl_3$): δ 8.48 (ddd, J = 5.0, 1.7, 1.0 Hz, 1H), 7.63 (ddd, J = 7.9, 7.5, 1.7 Hz, 1H), 7.45 (app. d, J = 9.0 Hz, 2H), 7.27 (ddd, J = 7.9, 1.7, 1.0 Hz, 1H), 7.25 (app. d, J = 9.0 Hz, 2H), 7.15 (ddd, J = 7.5, 5.0, 1.0 Hz, 1H), 5.88 (s, 1H), 2.25 (ABqq, 2H, $\Delta\nu_{AB}$ = 37.5 Hz, J = 14.0, 7.3 Hz, 2H), 0.83 (t, J = 7.3 Hz, 3H) ppm; ^{13}C{^{1}H} NMR (125 MHz, $CDCl_3$): δ 163.0, 147.2, 144.9, 136.9, 132.6, 128.2, 127.5, 122.0, 120.0, 77.0, 33.7, 7.8 ppm; The ^{1}H NMR data for this compound match the literature data.[10]

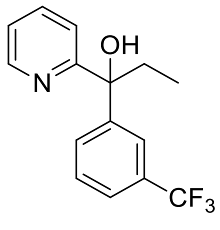

1-(Pyridin-2-yl)-1-(3-(trifluoromethyl)phenyl)propan-1-ol (5aj): The reaction was performed following General Procedure E with 2-(ethoxymethyl)pyridine (**1a**, 27.4 mg, 28 μL, 0.2 mmol) and 1-bromo-2-(trifluoromethyl)benzene (**7j**, 32.6 μL, 0.24 mmol). The crude product was purified by flash chromatography on silica gel (eluted with EtOAc:hexanes = 20:80) to afford the product (43.8 mg, 78% yield) as a yellow oil. ^{1}H NMR (500 MHz, $CDCl_3$): δ 8.50 (ddd, *J* = 4.9, 1.7, 1.0 Hz, 1H), 7.81 (br. s, 1H), 7.69 (app. d, *J* = 8.0 Hz, 1H), 7.65 (ddd, *J* = 7.9, 7.4, 1.7 Hz, 1H), 7.45 (app. d, *J* = 8.0 Hz, 1H), 7.40 (app. t, *J* = 8.0 Hz, 1H), 7.30 (ddd, *J* = 8.0, 1.7, 1.0 Hz, 1H), 7.18 (ddd, *J* = 7.4, 4.9, 1.0 Hz, 1H), 5.98 (s, 1H), 2.29 (ABqq, 2H, $\Delta\nu_{AB}$ = 28.2 Hz, *J* = 14.0, 7.3 Hz, 2H), 0.85 (t, *J* = 7.2 Hz, 3H) ppm; ^{13}C{^{1}H} NMR (125 MHz, $CDCl_3$): δ 162.5, 147.5, 147.3, 137.1, 130.4 (q, *J* = 32.5 Hz), 129.4, 128.5, 124.4 (q, *J* = 280.5 Hz), 123.6 (q, *J* = 2.5 Hz), 122.8 (q, *J* = 2.5 Hz), 122.1, 120.2, 77.1, 33.8, 7.7 ppm; IR (thin film): 3367, 2973, 2939, 1592, 1572, 1468, 1434, 1392, 1328, 1165, 1124, 1074, 988, 805, 703 cm^{-1}; HRMS calculated for $C_{15}H_{15}F_3NO$, 282.1106, found 282.1104 [M+H]$^+$.

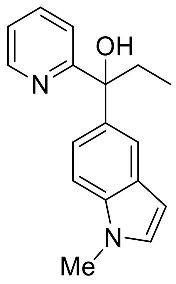

1-(1-Methyl-1H-indol-5-yl)-1-(pyridin-2-yl)propan-1-ol (5ag): The reaction was performed following General Procedure E with 2-(ethoxymethyl)pyridine (**1a**, 27.4 mg, 28 μL, 0.2 mmol) and 5-bromo-1-methyl-1*H*-indole (**7g**, 50.4 mg, 0.24 mmol). The crude product was purified by flash chromatography on silica gel (eluted with EtOAc:hexanes = 30:70) to afford the product (38.4 mg, 72% yield) as a white solid. m.p. = 105 – 106 °C; ^{1}H NMR (500 MHz, $CDCl_3$): δ 8.48 (ddd, *J* = 4.5, 1.5, 1.0 Hz, 1H), 7.81 – 7.78 (m, 1H),

7.57 (td, J = 8.0, 7.5, 1.5 Hz, 1H), 7.35 (app. d, J = 10.0 Hz, 1H), 7.32 (app. d, J = 8.0 Hz, 1H), 7.23 (app. d, J = 10.0 Hz, 1H), 7.10 (ddd, J = 7.5, 4.5, 1.0 Hz, 1H), 7.00 (d, J = 3.0 Hz, 1H), 6.44 (app. d, J = 3.0 Hz, 1H), 5.92 (s, 1H), 3.72 (s, 3H), 2.38 (ABqq, 2H, $\Delta\nu_{AB}$ = 60.2 Hz, J = 13.8, 7.2 Hz, 2H), 0.88 (t, J = 7.2 Hz, 3H) ppm; $^{13}C\{^{1}H\}$ NMR (125 MHz, $CDCl_3$): δ 164.5, 146.9, 137.3, 136.6, 135.6, 128.9, 128.1, 121.5, 120.6, 120.3, 118.0, 108.8, 101.1, 77.5, 34.0, 32.7, 8.0 ppm; IR (thin film): 3366, 3055, 2966, 2933, 1592, 1568, 1512, 1489, 1467, 1432, 1335, 1295, 1247, 1153, 1081, 984, 885, 721 cm^{-1}; HRMS calculated for $C_{17}H_{17}N_2$, 249.1392 found 249.1394 $[M{-}OH]^+$.

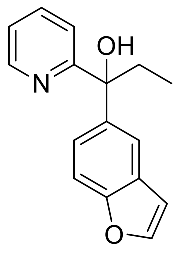

1-(Benzofuran-5-yl)-1-(pyridin-2-yl)propan-1-ol (5ah): The reaction was performed following General Procedure E with 2-(ethoxymethyl)pyridine (**1a**, 27.4 mg, 28 μL, 0.2 mmol) and 5-bromobenzofuran (**7h**, 30.1 μL, 0.24 mmol). The crude product was purified by flash chromatography on silica gel (eluted with EtOAc:hexanes = 25:75) to afford the product (35.5 mg, 70% yield) as a white solid. m.p. = 170 – 172 °C; ^{1}H NMR (500 MHz, $CDCl_3$): δ 8.49 (ddd, J = 4.5, 1.5, 1.0 Hz, 1H), 7.81 – 7.77 (m, 1H), 7.61 (dd, J = 8.0, 1.5 Hz, 1H), 7.56 (d, J = 2.0 Hz, 1H), 7.45 – 7.38 (m, 2H), 7.31 (app. d, J = 8.0 Hz, 1H), 7.13 (app. dd, J = 7.5, 5.0 Hz, 1H), 6.73 – 6.69 (m, 1H), 5.95 (s, 1H), 2.35 (ABqq, 2H, $\Delta\nu_{AB}$ = 48.5 Hz, J = 14.0, 7.3 Hz, 2H), 0.87 (t, J = 7.3 Hz, 3H) ppm; $^{13}C\{^{1}H\}$ NMR (125 MHz, $CDCl_3$): δ 163.9, 153.8, 147.1, 145.1, 141.1, 136.8, 127.1, 122.8, 121.7, 120.4, 118.6, 110.8, 106.7, 77.4, 34.0, 7.9 ppm; IR (thin film): 3366, 2969, 2935, 2877, 1592, 1570, 1465, 1433, 1392, 1327, 1261, 1132, 1109, 1030, 986, 892, 813, 771, 738 cm^{-1}; HRMS calculated for $C_{16}H_{16}NO_3$, 254.1181 found 254.1183 $[M+H]^+$.

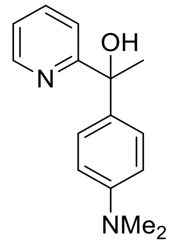

1-(4-(Dimethylamino)phenyl)-1-(pyridin-2-yl)ethanol (5bd): The reaction was performed following General Procedure E with 2-(methoxymethyl)pyridine (**1b**, 24.6 mg, 0.2 mmol) and 4-bromo-*N,N*-dimethylaniline (**7d**, 48.0 mg, 0.24 mmol). The crude product was purified by flash chromatography on silica gel (eluted with EtOAc:hexanes = 20:80) to afford the product (34.8 mg, 72% yield) as a yellow oil. ^{1}H NMR (500 MHz, $CDCl_3$): δ 8.51 (d, *J* = 4.9, 1.7, 1.0 Hz, 1H), 7.62 (ddd, *J* = 8.0, 7.5, 1.7 Hz, 1H), 7.32 (app. d, *J* = 9.0 Hz, 2H), 7.27 (ddd, *J* = 8.0, 1,7, 1.0 Hz, 1H), 7.15 (ddd, *J* = 7.5, 4.9, 1.0 Hz, 1H), 6.68 (d, *J* = 9.0 Hz, 2H), 5.72 (s, 1H), 2.91 (s, 6H), 1.89 (s, 3H) ppm; ^{13}C{^{1}H} NMR (125 MHz, $CDCl_3$): δ 165.5, 149.5, 147.2, 136.8, 135.1, 126.7, 121.7, 120.3, 112.2, 74.7, 40.6, 29.3 ppm; IR (thin film): 3393, 2976, 2928, 2800, 1613,1591, 1567, 1520, 1470, 1431, 1351, 1224, 1195, 1164, 1064, 947, 816, 785, 750,595 cm^{-1}; HRMS calculated for $C_{15}H_{17}N_2$, 225.1392 found 225.1398 [M–OH]$^+$.

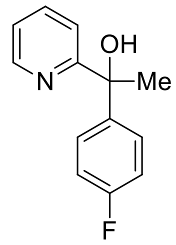

1-(4-Fluorophenyl)-1-(pyridin-2-yl)ethanol (5be): The reaction was performed following General Procedure E with 2-(methoxymethyl)pyridine (**1b**, 24.6 mg, 0.2 mmol) and 1-bromo-4-fluorobenzene (**7e**, 31.2 μL, 0.24 mmol). The crude product was purified by flash chromatography on silica gel (eluted with EtOAc:hexanes = 20:80) to afford the product (32.5 mg, 75% yield) as a yellow oil. ^{1}H NMR (500 MHz, $CDCl_3$): δ 8.52 (ddd, *J* = 5.0, 1.5, 1.0 Hz, 1H), 7.65 (ddd, *J* = 7.9, 7.5, 1.5 Hz, 1H), 7.44 (app. dd, *J* = 8.5, 5.0 Hz, 2H), 7.25 (ddd, *J* = 8.0, 1.5, 1.0 Hz, 1H), 7.19 (ddd, *J* = 7.5, 5.0, 1.0 Hz, 1H), 6.98 (app. dd, *J* = 9.0, 8.5 Hz, 2H), 5.83 (s,

1H), 1.90 (s, 3H) ppm; $^{13}C\{^1H\}$ NMR (125 MHz, $CDCl_3$): δ 164.5, 161.8 (d, J = 250.0 Hz), 147.4, 142.9 (d, J = 2.5 Hz), 137.0, 127.6 (d, J = 12.5 Hz), 122.1, 120.1, 115.3 (d, J = 25.0 Hz), 74.7, 29.3 ppm; IR (thin film): 3391, 3060, 2980, 2932, 1733, 1599, 1507, 1432, 1368, 1225, 1160, 1073, 925, 836, 750, 597 cm^{-1}; HRMS calculated for $C_{13}H_{11}FN$, 200.0876, found 200.0872 $[M–OH]^+$.

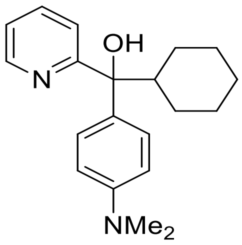

Cyclohexyl(4-(dimethylamino)phenyl)(pyridin-2-yl)methanol (5cd): The reaction was performed following General Procedure E with 2-((cyclohexyloxy)methyl)pyridine (**1c**, 38.2 mg, 0.2 mmol) and 4-bromo-*N,N*-dimethylaniline (**7d**, 48.0 mg, 0.24 mmol). The crude product was purified by flash chromatography on silica gel (eluted with EtOAc:hexanes = 20:80) to afford the product (50.8 mg, 82% yield) as a yellow oil. ^1H NMR (500 MHz, $CDCl_3$): δ 8.43 (ddd, J = 5.0, 1.5, 1.0 Hz, 1H), 7.61 (ddd, J = 7.9, 7.5, 1.5 Hz, 1H), 7.47 (app. d, J = 8.5 Hz, 2H), 7.42 (ddd, J = 7.9, 1.5, 1.0 Hz, 1H), 7.08 (ddd, J = 7.4, 5.0, 1.0 Hz, 1H), 6.69 (app. d, J = 8.5 Hz, 2H), 6.02 (s, 1H), 2.90 (s, 6H), 2.37 – 2.32 (m, 1H), 1.78 – 1.72 (m, 1H), 1.71 – 1.60 (m, 3H), 1.35 – 1.10 (m, 5H), 1.06 – 0.98 (m, 1H) ppm; $^{13}C\{^1H\}$ NMR (125 MHz, $CDCl_3$): δ 164.0, 149.1, 146.6, 136.8, 133.7, 126.7, 121.4, 120.3, 112.3, 79.0, 46.2, 40.5, 26.9, 26.8, 26.6, 26.4 ppm; IR (thin film): 3352, 2930, 2850, 1733, 1611, 1592, 1392, 1349, 1161, 1090, 998, 947, 823, 750, 647 cm^{-1}; HRMS calculated for $C_{20}H_{25}N_2$, 293.2018 found 293.2016 $[M+H]^+$.

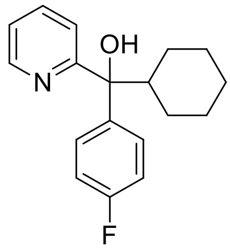

Cyclohexyl(4-fluorophenyl)(pyridin-2-yl)methanol (5ce): The

reaction was performed following General Procedure E with 2-((cyclohexyloxy)methyl)pyridine (**1c**, 38.2 mg, 0.2 mmol) and 1-bromo-4-fluorobenzene (**7e**, 31.2 *μ*L, 0.24 mmol). The crude product was purified by flash chromatography on silica gel (eluted with EtOAc:hexanes = 20:80) to afford the product (48.4, 85% yield) as a yellow oil. ^{1}H NMR (500 MHz, $CDCl_3$): δ 8.45 (ddd, *J* = 5.0, 1.5, 1.0 Hz, 1H), 7.65 (ddd, *J* = 7.9, 7.5, 1.5 Hz, 1H), 7.58 (app. dd, *J* = 8.5, 5.5 Hz, 2H), 7.41 (ddd, *J* = 8.0, 1.5, 1.0 Hz, 1H), 7.14 (ddd, *J* = 7.5, 5.0, 1.0 Hz, 1H), 6.98 (app. dd, *J* = 9.0, 8.5 Hz, 2H), 6.08 (s, 1H), 2.36 – 2.32 (m, 1H), 1.75 – 1.54 (m, 4H), 1.31 – 0.98 (m, 6H) ppm; $^{13}C\{^1H\}$ NMR (125 MHz, $CDCl_3$): δ 163.1, 162.6 (d, *J* = 250.0 Hz), 146.9, 141.6 (d, *J* = 2.5 Hz), 137.1, 127.6 (d, *J* = 12.5 Hz), 121.8, 120.3, 114.9 (d, *J* = 25.0 Hz), 79.1, 46.3, 26.8, 26.7, 26.6, 26.5, 26.3 ppm; IR (thin film): 3339, 2932, 2852, 1593, 1505, 1433, 1394, 1224, 1158, 1086, 999, 835, 751 cm^{-1}; HRMS calculated for $C_{18}H_{19}FN$, 268.1502, found 268.1499 $[M–OH]^+$.

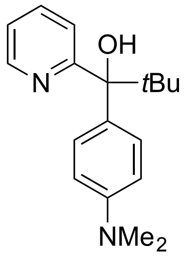

1-(4-(Dimethylamino)phenyl)-2,2-dimethyl-1-(pyridin-2-yl)propan-1-ol (5dd): The reaction was performed following General Procedure E with 2-(*tert*-butoxymethyl)pyridine (**1d**, 33.0 mg, 0.2 mmol) and 4-bromo-*N,N*-dimethylaniline (**7d**, 48.0 mg, 0.24 mmol). The crude product was purified by flash chromatography on silica gel (eluted with EtOAc:hexanes = 20:80) to afford the product (37.0 mg, 65% yield) as a yellow solid. m.p. = 103 – 105 °C, ^{1}H NMR (500 MHz, $CDCl_3$): δ 8.49 (d, *J* = 5.0 Hz, 1H), 7.75 (d, *J* = 8.1 Hz, 1H), 7.66 (td, *J* = 7.8, 1.0 Hz, 1H), 7.59 (d, *J* = 9.0 Hz, 2H), 7.16 (dd, *J* = 6.9, 5.4 Hz, 1H), 6.65 (d, *J* = 9.0 Hz, 2H), 6.31 (s, 1H), 2.90 (s, 6H), 1.06 (s, 9H) ppm; $^{13}C\{^1H\}$ NMR (125 MHz, $CDCl_3$): δ 163.2, 149.2, 146.8, 136.0, 132.5, 129.1, 123.0, 122.0, 111.6, 81.2, 40.7, 40.1, 27.2; HRMS calculated for $C_{18}H_{25}N_2O$, 285.1967, found 285.1965 [M+

H]$^+$.

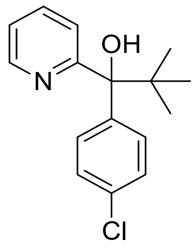

1-(4-Chlorophenyl)-2,2-dimethyl-1-(pyridin-2-yl)propan-1-ol (5di): The reaction was performed following General Procedure E with 2-(*tert*-butoxymethyl)pyridine (**1d**, 33.0 mg, 0.2 mmol) and 1-bromo-4-chlorobenzene (**7i**, 45.8 mg, 0.24 mmol). The crude product was purified by flash chromatography on silica gel (eluted with EtOAc:hexanes = 20:80) to afford the product (33.0 mg, 60% yield) as a yellow oil. ^{1}H NMR (500 MHz, $CDCl_3$): δ 8.51 (ddd, *J* = 4.5, 1.5, 1.0 Hz, 1H), 7.77 (d, *J* = 8.5 Hz, 1H), 7.76 – 7.69 (m, 1H), 7.70 (d, *J* = 9.0 Hz, 2H) 7.26 – 7.20 (m, 3H), 6.40 (s, 1H), 1.05 (s, 9H) ppm; ^{13}C{^{1}H} NMR (125 MHz, $CDCl_3$): δ 161.9, 146.8, 142.7, 136.2, 132.4, 129.6, 127.2, 122.6, 122.2, 81.0, 39.7, 26.8 ppm; IR (thin film): 3277, 2958, 1591, 1571, 1488, 1464, 1434, 1399, 1362, 1095, 1064, 1012, 831, 768 cm^{-1}; HRMS calculated for $C_{16}H_{17}NCl$, 258.1050, found 258.1053 [M–OH]$^+$.

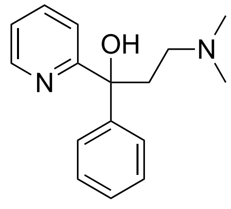

3-(Dimethylamino)-1-phenyl-1-(pyridin-2-yl)propan-1-ol (5ha): The reaction was performed following General Procedure E at 60 °C for 24 h with *N*,*N*-dimethyl-2-(pyridin-2-ylmethoxy)ethan-1-amine (**1h**, 36.1 mg, 0.2 mmol) and bromobenzene (**7a**, 25.2 *μ*L, 0.24 mmol). The crude product was purified by flash chromatography on silica gel (dichloromethane:methanol = 10:1) to afford the product (37.9 mg, 74% yield) as a white solid. m.p. = 98 – 100 °C; ^{1}H NMR (500 MHz, $CDCl_3$): δ 8.52 (ddd, *J* = 4.9, 1.8, 1.0 Hz, 1H), 7.70 – 7.59 (m, 4H), 7.32 – 7.26 (m, 2H), 7.21 – 7.15 (m, 1H), 7.11 (ddd, = 7.2, 4.9, 1.0 Hz, 1H), 2.74 – 2.66 (m, 1H),

2.62 – 2.48 (m, 3H), 2.32 (s, 6H) ppm; $^{13}C\{^1H\}$ NMR (125 MHz, $CDCl_3$): δ 165.4, 147.9, 146.6, 136.9, 128.2, 126.8, 125.7, 121.8, 121.1, 79.1, 56.1, 44.8, 35.9 ppm; IR (thin film): 3309, 3054, 2976, 2950, 2860, 2825, 2780, 1586, 1568, 1463, 1447, 1427, 1380, 1307, 1212, 1195, 1178, 1164, 1120, 1066, 1047 cm^{-1}; HRMS calculated for $C_{16}H_{21}N_2O$, 257.1654, found 257.1653 $[M+H]^+$.

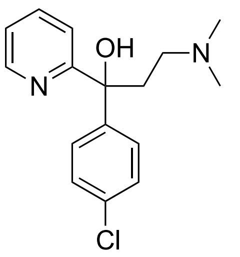

1-(4-Chlorophenyl)-3-(dimethylamino)-1-(pyridin-2-yl)propan-1-ol (5hi): The reaction was performed following General Procedure E at 60 °C for 24 h with *N*,*N*-dimethyl-2-(pyridin-2-ylmethoxy)ethan-1-amine (**1h**, 36.1 mg, 0.2 mmol) and 1-bromo-4-chlorobenzene (**7i**, 45.8 mg, 0.24 mmol). The crude product was purified by flash chromatography on silica gel (dichloromethane:methanol = 10:1) to afford the product (41.9 mg, 72% yield) as a white solid. m.p. = 87 – 90 °C; ^1H NMR (500 MHz, $CDCl_3$): δ 8.52 (ddd, J = 4.9, 1.8, 1.0 Hz, 1H), 7.70 (ddd, J = 8.0, 7.5, 1.8 Hz, 1H), 7.61 (ddd, J = 8.0, 1.5, 1.0 Hz, 1H), 7.55 (app. d, J = 9.0 Hz, 2H), 7.28 (app. d, J = 9.0 Hz, 2H), 7.19 (ddd = J = 7.5, 4.9, 1.0 Hz, 1H), 3.05 – 2.75 (m, 4H), 2.61 (s, 6H) ppm; $^{13}C\{^1H\}$ NMR (125 MHz, $CDCl_3$): δ 162.9, 147.9, 143.9, 137.7, 133.2, 128.7, 127.3, 122.6, 120.9, 77.2, 55.4, 43.9, 35.6 ppm; IR (thin film): 3307, 3056, 3011, 2953, 2826, 2679, 2468, 1588, 1569, 1489, 1467, 1400, 1292, 1250, 1091, 1056, 1013 cm^{-1}; HRMS calculated for $C_{16}H_{20}N_2OCl$, 291.1264, found 291.1265 $[M+H]^+$.

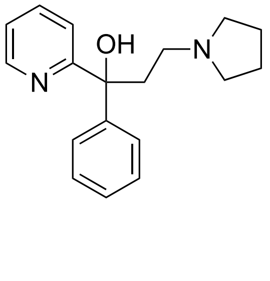

1-Phenyl-1-(pyridin-2-yl)-3-(pyrrolidin-1-yl)propan-1-ol

(5ia): The reaction was performed following General Procedure E at 60 °C for 24 h with 2-((2-(pyrrolidin-1-yl)ethoxy)methyl)pyridine (**1i**, 41.3 mg, 0.2 mmol) and bromobenzene (**7a**, 25.2 μL, 0.24 mmol). The crude product was purified by flash chromatography on silica gel (dichloromethane:methanol = 10:1) to afford the product (36.1 mg, 64% yield) as a white solid. m.p. = 90 – 93 °C; ^{1}H NMR (500 MHz, $CDCl_3$): δ 8.52 (dt, *J* = 4.9, 1.3 Hz, 1H), 7.71 – 7.64 (m, 2H), 7.63 – 7.58 (m, 2H), 7.34 – 7.28 (m, 2H), 7.23 – 7.18 (m, 2H), 7.14 (ddd, *J* = 5.9, 4.9. 2.7 Hz, 1H), 2.92 – 2.64 (m, 8H), 1.97 – 1.84 (m, 4H) ppm; $^{13}C\{^1H\}$ NMR (125 MHz, $CDCl_3$): δ 165.2, 147.9, 146.6, 137.0, 128.3, 126.8, 125.7, 121.9, 121.1, 78.9, 53.9, 52.5, 36.9, 23.5 ppm; IR (thin film): 3254, 3056, 2963, 2931, 2811, 1588, 1569, 1490, 1464, 1447, 1430, 1203, 1137, 1068 cm^{-1}; HRMS calculated for $C_{18}H_{23}N_2O$, 283.1810, found 283.1806 $[M+H]^+$.

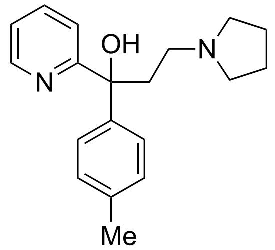

1-(Pyridin-2-yl)-3-(pyrrolidin-1-yl)-1-(*p*-tolyl)propan-1 (5ik): The reaction was performed following General Procedure E at 60 °C for 24 h with 2-((2-(pyrrolidin-1-yl)ethoxy)methyl)pyridine (**1i**, 41.3 mg, 0.2 mmol) and 4-bromotoluene (**7k**, 29.5 μL, 0.24 mmol). The crude product was purified by flash chromatography on silica gel (dichloromethane:methanol = 10:1) to afford the product (42.1 mg, 71% yield) as a white solid. m.p. = 116 – 118 °C; ^{1}H NMR (500 MHz, $CDCl_3$): δ 8.52 (ddd, *J* = 4.9, 1.8. 1.0 Hz, 1H), 7.66 (ddd, *J* = 8.0, 1.0. 1.0 Hz, 1H), 7.61 (ddd, *J* = 8.0, 7.5. 1.8 Hz, 1H), 7.49 (app. d, *J* = 8.0 Hz, 2H), 7.10 (app. d, *J* = 8 .0 Hz, 2H), 7.08 (ddd, *J* = 7.5, 4.9, 1.0 Hz, 1H), 2.75 – 2.50 (m, 4H), 2.28 (s, 3H), 1.84 – 1.74 (m, 4H) ppm; $^{13}C\{^1H\}$ NMR (125 MHz, $CDCl_3$): δ 166.1, 147.9, 144.1, 136.8, 136.1, 128.8, 125.6, 121.6, 121.0, 79.4, 53.9, 52.7, 37.0, 23.6, 21.1 ppm; IR (thin film): 3154, 3055, 2964, 2926, 2876, 2810, 1588, 1568, 1510, 1463, 1430, 1293, 1177, 1138, 1110, 1082 cm^{-1}; HRMS calculated for $C_{19}H_{25}N_2O$, 297.1967, found

297.1969 $[M+H]^+$.

5. Procedure and characterization of Pd-catalyzed selective C(sp^3)–H arylation of 4-pyridylmethyl ethers (Table 6).

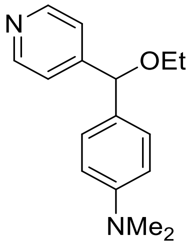

4-(Ethoxy(pyridin-4-yl)methyl)-*N,N*-dimethylaniline (4jd): The reaction was performed following General Procedure D with 4-(ethoxymethyl)pyridine (**1j**, 27.4 mg, 0.2 mmol) and 4-bromo-*N,N*-dimethylaniline (**7d**, 48.0 mg, 0.24 mmol) with $Pd(OAc)_2$ (1 mol %) and NIXANTPHOS (1.5 mol %) in 1 mL of dry DME for 12 h at 23 °C. The crude product was purified by flash chromatography on silica gel (eluted with EtOAc:hexanes = 10:90) to afford the product (43.6 mg, 85% yield) as a yellow oil. ^{1}H NMR (500 MHz, $CDCl_3$): δ 8.49 (dd, J = 5.5, 1.5 Hz, 2H), 7.26 (m, 2H), 7.13 (d, J = 9.0 Hz, 2H), 6.66 (d, J = 9.0 Hz, 2H), 5.21 (s, 1H), 3.47 (ABqq, 2H, $\Delta\nu_{AB}$ = 26.3 Hz, J = 9.2, 7.0 Hz, 2H), 2.90 (s, 6H), 1.23 (t, J = 7.0 Hz, 3H) ppm; ^{13}C{^{1}H} NMR (125 MHz, $CDCl_3$): δ 152.1, 150.2, 149.6, 128.3, 128.2, 121.4, 112.2, 81.8, 64.2, 40.3, 15.2 ppm; IR (thin film): 2889, 1614, 1586, 1522, 1492, 1433, 1350, 1228, 1186, 1164, 1032, 992, 946, 885, 804, 751, 690 cm^{-1}; HRMS calculated for $C_{16}H_{21}N_2O$, 257.1654, found 257.1650 $[M+H]^+$.

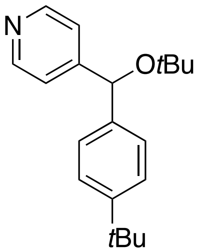

4-(*tert*-Butoxy(4-(*tert*-butyl)phenyl)methyl) (4kb): The reaction was performed following General Procedure D with 4-(butoxymethyl)pyridine (**1k**, 33.0 mg, 0.2 mmol) and 1-bromo-4-*ter*t-butylbenzene (**7b**, 42.0 μL, 0.24 mmol) with

Pd(OAc)$_2$ (1 mol %) and NIXANTPHOS (1.5 mol %) in 1 mL of dry DME for 12 h at 23 °C. The crude product was purified by flash chromatography on silica gel (eluted with EtOAc:hexanes = 20:80) to afford the product (51.2 mg, 86% yield) as a yellow oil. The same reaction in 1 mL of dry CPME for 6 h at 60 °C following General Procedure E resulted in the product (50.0 mg, 84% yield). ^{1}H NMR (500 MHz, CDCl$_3$): δ 8.50 – 8.48 (m, 2H), 7.31 – 7.26 (m, 4H), 7.19 (d, *J* = 8.5 Hz, 2H), 5.50 (s, 1H), 1.27 (s, 9H), 1.20 (s, 9H) ppm; ^{13}C{^{1}H} NMR (125 MHz, CDCl$_3$): δ 154.3, 150.4, 149.8, 140.6, 126.7, 125.5, 122.0, 75.5, 74.7, 34.6, 31.5, 28.8 ppm; IR (thin film): 3026, 2967, 2094, 2869, 1596, 1562, 1511, 1463, 1413, 1391, 1366, 1269, 1109, 1065, 1018 cm^{-1}; HRMS calculated for $C_{20}H_{27}NO$, 298.2171, found 298.2176 [M+H]$^+$.

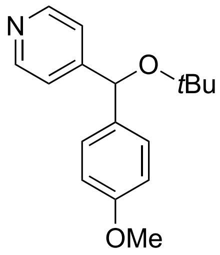

4-(*tert*-Butoxy(4-methoxyphenyl)methyl)pyridine (4kc): The reaction was performed following General Procedure D with 4-(butoxymethyl)pyridine (**1k**, 33.0 mg, 0.2 mmol) and 4-bromoanisole (**7c**, 30.0 μL, 0.24 mmol) with Pd(OAc)$_2$ (1 mol %) and NIXANTPHOS (1.5 mol %) in 1 mL of dry DME for 12 h at 23 °C. The crude product was purified by flash chromatography on silica gel (eluted with EtOAc:hexanes = 20:80) to afford the product (47.8 mg, 88% yield) as a yellow oil. ^{1}H NMR (500 MHz, CDCl$_3$): δ 8.48 (dd, *J* = 4.5, 1.5 Hz, 2H), 7.25 (m, 2H), 7.19 (d, *J* = 9.0 Hz, 2H), 6.81 (d, *J* = 9.0 Hz, 2H), 5.47 (s, 1H), 3.75 (s, 3H), 1.20 (s, 9H) ppm; ^{13}C{^{1}H} NMR (125 MHz, CDCl$_3$): δ 159.0, 154.3, 149.8, 136.0, 128.3, 121.8, 114.0, 75.5, 74.4, 5.4, 28.8 ppm; IR (thin film): 3074, 3027, 2974, 2934, 2906, 2836, 1610, 1597, 1561, 1511, 1464, 1477, 1391, 1367, 1303, 1248, 1172, 1110, 1089, 1064, 1035 cm^{-1}; HRMS calculated for $C_{17}H_{22}NO_2$, 272.1652, found 272.1636 (-5.5 ppm, only choice) [M+H]$^+$.

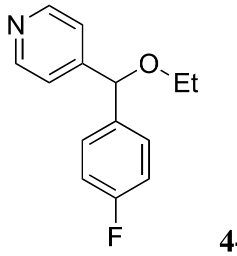

4-(Ethoxy(4-fluorophenyl)methyl)pyridine (4je): The reaction was performed following General Procedure E with 4-(ethoxymethyl)pyridine (**1j**, 27.4 mg, 0.2 mmol) and 1-bromo-4-fluorobenzene (**7e**, 31.2 μL, 0.24 mmol) with $Pd(OAc)_2$ (1 mol %) and NIXANTPHOS (1.5 mol %) in 1 mL of dry CPME for 12 h at 60 °C. The crude product was purified by flash chromatography on silica gel (eluted with EtOAc:hexanes = 10:90) to afford the product (40.2 mg, 87% yield) as a yellow oil. ^{1}H NMR (500 MHz, $CDCl_3$): δ 8.52 (dd, J = 4.5, 1.5 Hz, 1H), 7.29 – 7.25 (m, 2H), 7.25 – 7.22 (m, 2H), 7.00 (t, J = 8.5 Hz, 2H), 5.27 (s, 1H), 3.52 – 3.44 (m, 2H), 1.25 (t, J = 7.0 Hz, 3H) ppm; $^{13}C\{^1H\}$ NMR (125 MHz, $CDCl_3$): δ 162.6, (d, J = 245.0 Hz), 151.4, 150.1, 137.0 (d, J = 3.5 Hz), 129.0 (d, J = 8.1 Hz), 121.7, 115.7 (d, J = 21.2 Hz), 81.8, 65.0, 15.4 ppm; IR (thin film): 3030, 2976, 2872, 1600, 1561, 1508, 1443, 1411, 1305, 1223, 1187, 1157, 1104, 1091, 1015, 993 cm^{-1}; HRMS calculated for $C_{14}H_{15}NOF$, 232.1138, found 232.1134 $[M+H]^+$.

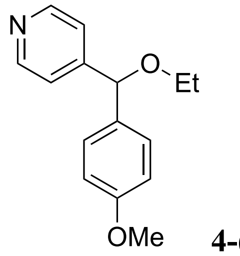

4-(Ethoxy(4-methoxyphenyl)methyl)pyridine (4jc): The reaction was performed following General Procedure E with 4-(ethoxymethyl)pyridine (**1j**, 27.4 mg 0.2 mmol) and 4-bromoanisole (**7c**, 30.0 μL, 0.24 mmol) with $Pd(OAc)_2$ (1 mol %) and NIXANTPHOS (1.5 mol %) in 1 mL of dry CPME for 12 h at 60 °C. The crude product was purified by flash chromatography on silica gel (eluted with EtOAc:hexanes = 20:80) to afford the product (42.8 mg, 88% yield) as a yellow oil. ^{1}H NMR (500 MHz, $CDCl_3$): δ 8.51 (m, 2H), 7.26 – 7. 23 (m, 2H), 7.20 (d, J = 8.5 Hz, 2H), 6.84 (d, J = 8.5 Hz, 2H), 5.24 (s, 1H), 3.76 (s, 3H), 3.53 – 3.41 (m, 2H), 1.24 (t,

J = 7.0 Hz, 3H) ppm; $^{13}C\{^1H\}$ NMR (125 MHz, $CDCl_3$): δ 159.6, 151.9, 150.0, 133.2, 128.6, 121.7, 114.2, 81.9, 64.7, 55.4, 15.4 ppm; IR (thin film): 3029, 2974, 2932, 2896, 2871, 2837, 1610, 1599, 1561, 1511, 1463, 1442, 1412, 1342, 1304, 1249, 1173, 1111, 1096, 1081, 1034, 992 cm^{-1}; HRMS calculated for $C_{15}H_{18}NO_2$, 244.1338, found 244.1314 9 (only choice) $[M+H]^+$.

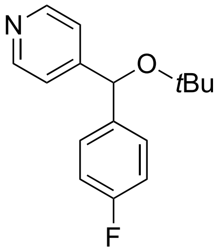

4-(*tert*-Butoxy(4-fluorophenyl)methyl) pyridine (4ke): The reaction was performed following General Procedure E with 4-(ethoxymethyl)pyridine (**1k**, 33.0 mg, 0.2 mmol) and 1-bromo-4-fluorobenzene (**7e**, 31.2 μL, 0.24 mmol) with $Pd(OAc)_2$ (1 mol %) and NIXANTPHOS (1.5 mol %) in 1 mL of dry CPME for 12 h at 60 °C. The crude product was purified by flash chromatography on silica gel (eluted with EtOAc:hexanes = 20:80) to afford the product (41.5 mg, 80% yield) as a yellow oil. ^1H NMR (500 MHz, $CDCl_3$): δ 8.49 (dd, J = 4.5, 2.0 Hz, 2H), 7.29 – 7.21 (m, 4H), 6.96 (t, J = 8.7 Hz, 2H), 5.49 (s, 1H), 1.19 (s, 9H) ppm; $^{13}C\{^1H\}$ NMR (125 MHz, $CDCl_3$): δ 162.2 (d, J = 244.2 Hz), 153.8, 149.9, 139.7 (d, J = 2.9 Hz), 128.7 (d, J = 8.0 Hz), 121.8, 115.5 (d, J = 21.1 Hz), 75.8, 74.2, 28.8 ppm; IR (thin film): 3029, 2975, 2934, 1597, 1561, 1508, 1471, 1412, 1391, 1368, 1295, 1223, 1183, 1156, 1099, 1087, 1065, 1025, 1015, 993 cm^{-1}; HRMS calculated for $C_{16}H_{19}NOF$, 260.1451, found 260.1457 $[M+H]^+$.

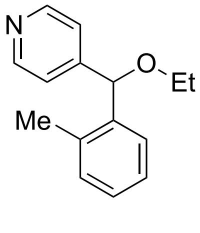

4-(Ethoxy(*o*-tolyl)methyl)pyridine (4jf): The reaction was performed following General Procedure D with 4-(ethoxymethyl)pyridine (**1j**, 27.4 mg, 0.2 mmol), 1-bromo-2-methylbenzene (**7f**, 28.8 μL, 0.24 mmol) with $Pd(OAc)_2$

(1 mol %) and NIXANTPHOS (1.5 mol %) in 1 mL of dry CPME for 12 h at 60 °C. The crude product was purified by flash chromatography on silica gel (eluted with EtOAc:hexanes = 20:80) to afford the product (40.0 mg, 88% yield) as a yellow oil. ^{1}H NMR (500 MHz, $CDCl_3$): δ 8.51 (dd, J = 5.0, 1.5 Hz, 2H), 7.32 – 7.10 (m, 6H), 5.49 (s, 1H), 3.57 – 3.44 (m, 2H), 2.26 (s, 3H), 1.25 (t, J = 7.0 Hz, 3H) ppm; $^{13}C\{^1H\}$ NMR (125 MHz, $CDCl_3$): δ 150.8, 150.0, 138.7, 136.4, 131.0, 128.2, 127.9, 126.4, 122.2, 80.0, 65.0, 19.6, 15.5 ppm; IR (thin film): 3025, 2975, 2871, 1597, 1560, 1491, 1460, 1411, 1308, 1180, 1112, 1081, 993 cm^{-1}; HRMS calculated for $C_{15}H_{18}NO$, 228.1388, found 228.1398 $[M+H]^+$.

6. Confirmation of the order of tandem arylation/[1,2]-Wittig rearrangement.

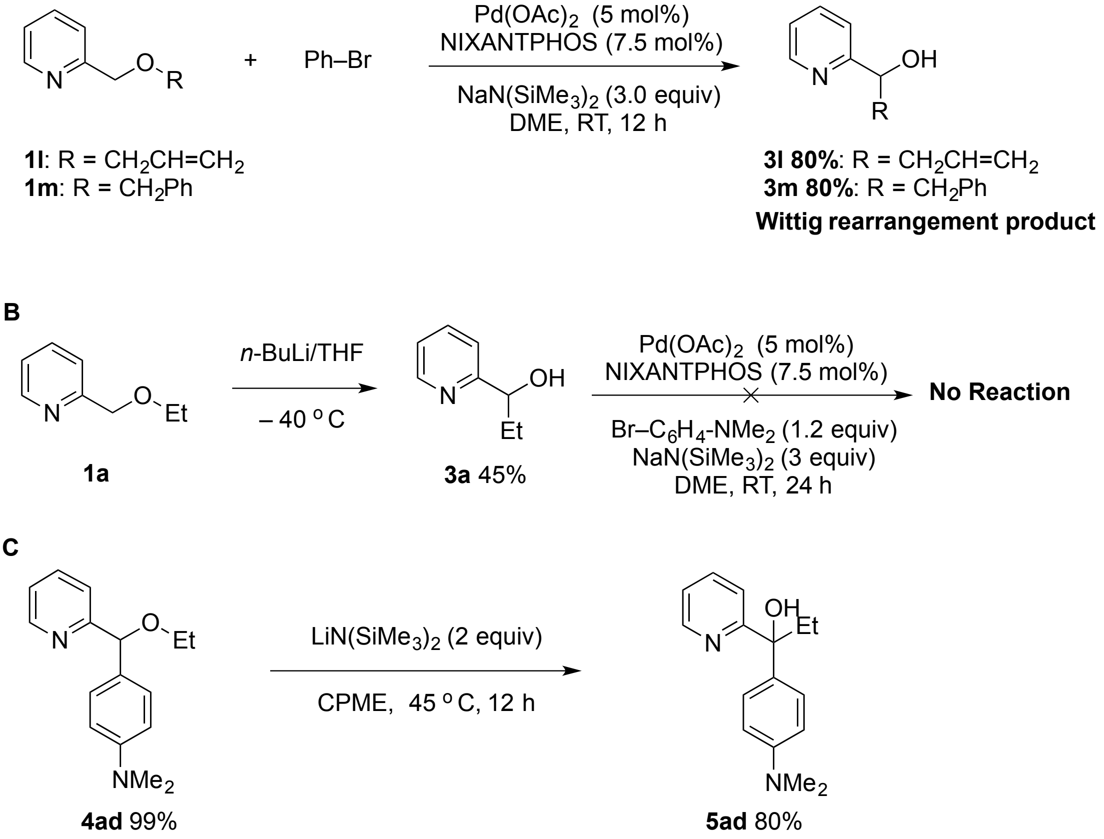

Scheme S1. Confirmation of the order of tandem arylation/Wittig rearrangement

Scheme S1-A:

The reaction was performed following General Procedure D with

2-((allyloxy)methyl)pyridine (**1l**, 32.6 mg, 0.2 mmol) or 2-((benzyloxy)methyl)pyridine (**1m**, 39.9 mg, 0.2 mmol) and bromobenzene (**7a**, 25.2 mL, 0.24 mmol). The crude products were purified by flash chromatography on silica gel to afford the Wittig rearrangement products: 1-(Pyridin-2-yl)but-3-en-1-ol (**3l**, 26.0 mg, 80% yield) or 2-phenyl-1-(pyridin-2-yl)ethan-1-ol (**3m**, 32.0 mg, 80% yield).

Scheme S1-B:

In a 10 mL dry one-necked flask under a nitrogen atmosphere was added 2-(ethoxymethyl)pyridine (**1a**, 109.6 mg, 112 μL, 0.8 mmol) and THF (5 mL) at room temperature. The reaction vessel was cooled to – 40 °C in a dry ice bath and stirred for 5 min. *n*BuLi (0.5 ml, 2.5 M in hexanes, 1.5 equiv) was added dropwise. The reaction mixture was stirred for 2 h at – 40 °C and warmed to room temperature. Next, the reaction mixture was quenched by addition of 1 mL of water and the solvent was removed *in vacuo* to yield the crude product. The crude product was dissolved in EtOAc (20 mL) and washed with brine (3 x 5 mL). The organic layer was dried over $MgSO_4$, filtered, the filtrate collected and the volatile materials removed *in vacuo* to yield a yellow oil. This oil was purified by flash column chromatography on silica gel (hexanes:EtOAc = 10:1) to afford 1-(pyridin-2-yl)propan-1-ol **3a** as a yellow oil (**3a**, 49.3 mg, 45% yield).[11] 1-(Pyridin-2-yl)propan-1-ol (**3a**, 28 mg, 0.2 mmol) was subjected to General Procedure C with 4-bromo-*N,N*-dimethylaniline (**7d**, 48.0 mg, 0.24 mmol), but no arylation product was observed. This result indicates that the arylation proceeds the Wittig rearrangement.

Scheme S1-C:

An oven-dried 10 mL reaction vial equipped with a stir bar was charged with $LiN(SiMe_3)_2$ (66.9 mg, 0.4 mmol, 2 equiv) under a nitrogen atmosphere at room temperature followed by 2 mL of dry CPME. The reaction mixture was stirred for 5 min at room temperature. Next, 4-(ethoxy(pyridin-2-yl)methyl)-*N*,*N*-dimethylaniline (**4ad**, 51.2 mg, 0.2 mmol, 1 equiv) was added to the reaction mixture under a purge of

nitrogen. The vial was capped, removed from the glove box, and stirred for 12 h at 45 °C until TLC showed complete consumption of **4ad**. The reaction mixture was cooled to room temperature, quenched with four drops of H_2O, diluted with 3 mL of ethyl acetate, and filtered over a pad of $MgSO_4$ and silica. The pad was rinsed with additional ethyl acetate (3 mL) and the solution was concentrated *in vacuo*. The crude product was purified by flash chromatography on silica gel (eluted with EtOAc:hexanes = 10:90) to afford 1-(4-(dimethylamino)phenyl)-1-(pyridin-2-yl)propan-1-ol (**5ad**, 40.9 mg, 80% yield) as a yellow oil.

7. Effect of Alkali Metals, Solvents and Additives in [1,2]-Wittig Rearrangement of Arylation product 4ab.

Experiments were set up inside a glove box under a nitrogen atmosphere.

1. Stock solutions of starting materials: **4ab** in CPME (0.4 M) and **4ab** in DME (0.4 M) were prepared.

2. Stock Solutions of bases: $LiN(SiMe_3)_2$ in CPME (0.6 M), $NaN(SiMe_3)_2$ in CPME (0.6 M), $LiN(SiMe_3)_2$ in DME (0.6 M), and $NaN(SiMe_3)_2$ in DME (0.6 M) were prepared.

General Procedure F for [1,2]-Wittig Rearrangement of 4ab (entry 5):

To a 4 mL reaction vial equipped with a stir bar was added 0.6 M $LiN(SiMe_3)_2$ in CPME (0.075 mmol, 125 μL) and 12-crown-4 (0.075 mmol, 12.1 μL) at room temperature. After the vial was stirred for 3 min, 0.4 M **4ab** in CPME (0.05 mmol, 125 μL) was added to the reaction vial at room temperature. The vial was capped, removed from the glove box, and stirred for 1 h at 45 °C, then cooled to room temperature. The reaction mixture was quenched with one drop of H_2O, diluted with ethyl acetate (3 mL), and filtered over a pad of celite. The pad was rinsed with additional ethyl acetate (3 mL) and the solution was concentrated *in vacuo*. The sample was dissolved in a mixture of 1 mL of 0.05 M CH_2Br_2 in $CDCl_3$ and analyzed by ^1H NMR.

The results are summarized below.

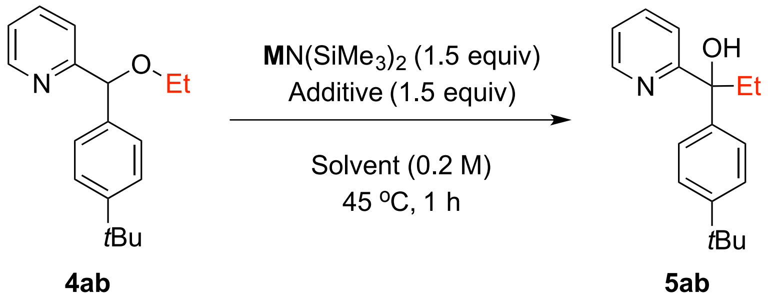

entry	solvent	M	additive	**4ab** (%)[b]	**5ab** (%)[b]
1	CPME	Li	–	37	64
2	CPME	Na	–	24	75
3	DME	Li	–	72	27
4	DME	Na	–	85	9
5	CPME	Li	12-Crown-4	53	47
6	CPME	Na	12-Crown-4	90	7
**7**	CPME	Na	15-Crown-5	86	13

[a] Reaction conditions: **4ab** (0.05 mmol), **M**$N(SiMe_3)_2$ (0.075 mmol), Additive (0.075 mmol) in solvent (0.25 mL) at 45 °C. [b] Yield determined by ^1H NMR spectroscopy of the crude reaction mixture.

Reference:

1. Dongjaw, T., William, P. W., *J. Org. Chem.*, **1981**, *46*, 265.
2. Hu Y. L., Ma X. Y., Lu M., *Can. J. Chem.*, **2011**, *89*, 471.
3. Belen, A., Rosa, A., Shamim, A., Rafael, B., Sonia, L.-M., *Arkivoc*, **2014**, 175.
4. Wallis, A., *J. Chem. Soc.*, **1979**, 584.
5. Cong, X., You, J., Gao, G., Lan, J., *Chem. Comm.*, **2013**, *49*, 662.
6. Kojiro, S., Masahiro, G., *Anal. Chem.*, **2004**, *76*, 5039.
7. Zeits, P. D., Rachiero, G. P., Hampel, F., Reibenspies, J. H., Gladysz, J. A. *Organometallics*, **2012**, *31*, 2854.
8. Legault, C. Y., Charette, A. B. *J. Am. Chem. Soc.*, **2005**, *127*, 8966.
9. Yang, J., Dudley, G. B., *J. Org. Chem.*, **2009**, *74*, 7998.
10. Froimowitz, M., Gu, Y., Dakin, L. A., Kelley, C. J., Parrish, D., Deschamps, J. R. *Bioorg. Med. Chem. Lett.*, **2005**, *15*, 3044.
11. Beckendorf, S., Mancheno, O. G. *Synthesis*, **2012**, *44*, 2162.

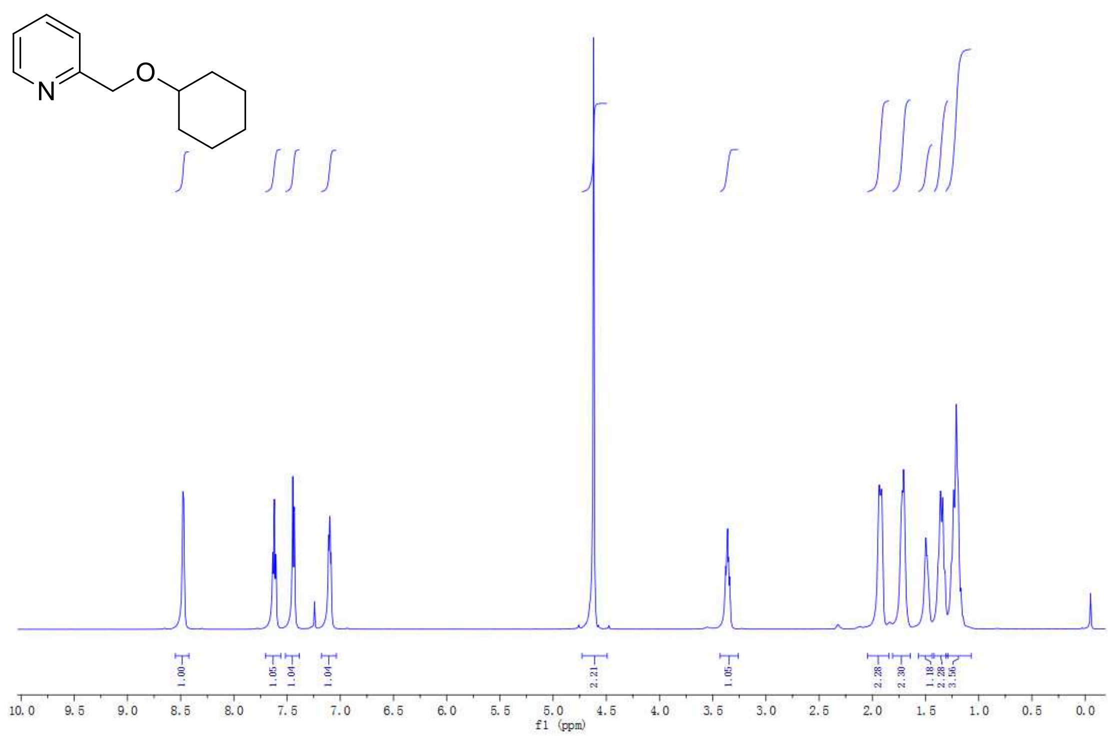

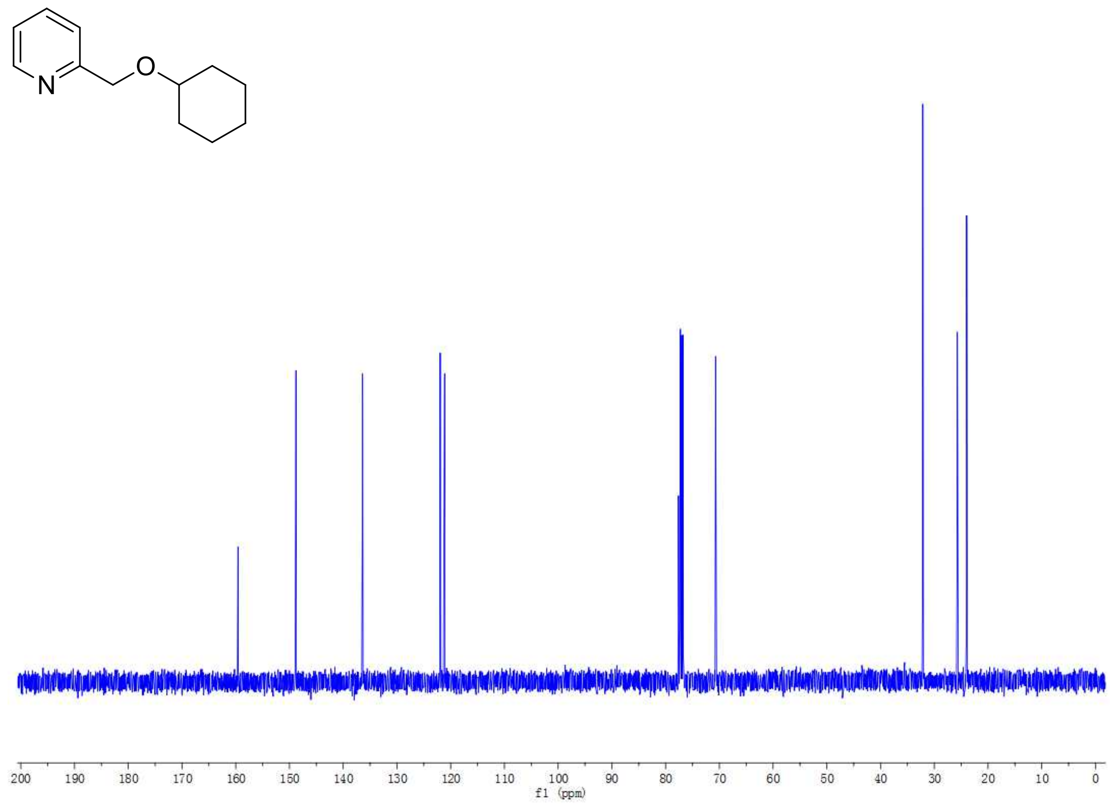

Figure S1. 500 MHz ^{1}H and 125 MHz ^{13}C{^{1}H} NMR spectra of **1c** in $CDCl_3$.

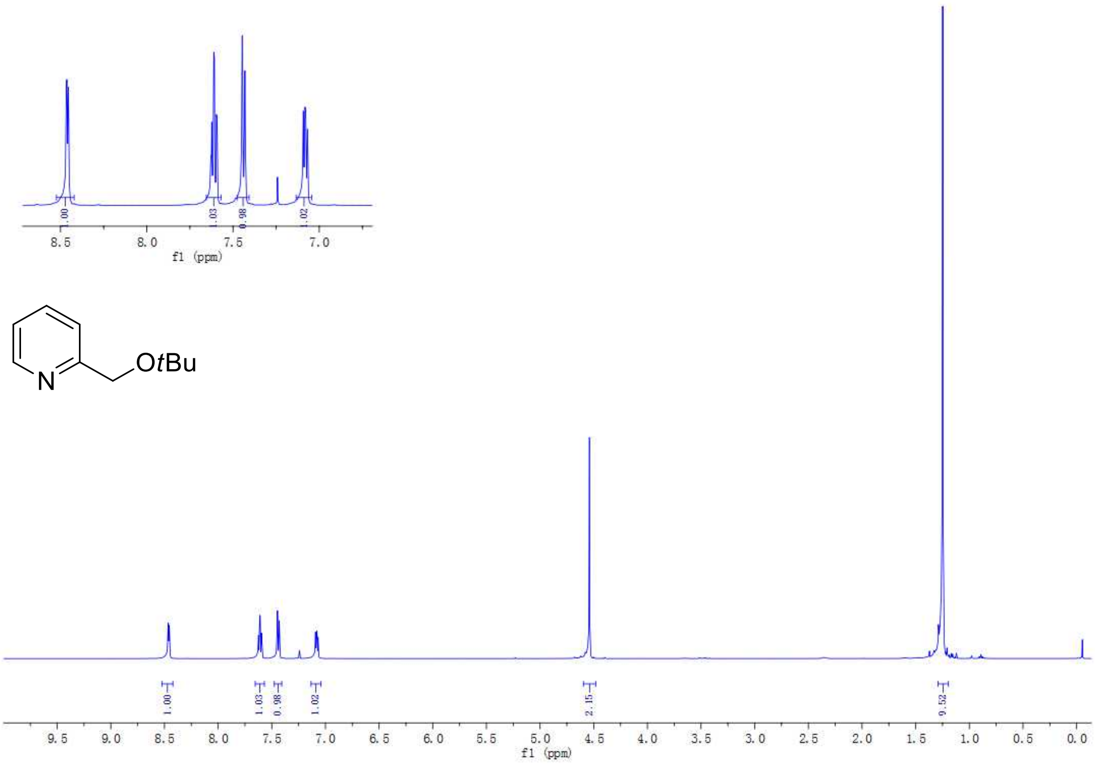

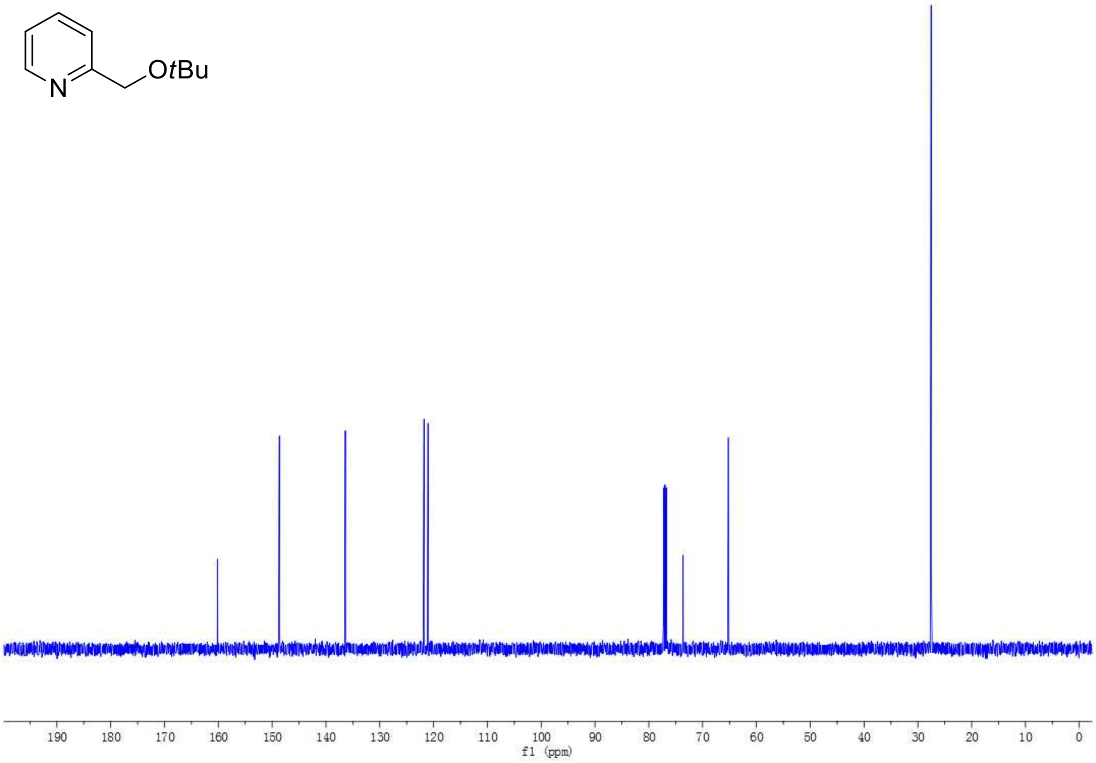

Figure S2. 500 MHz ^{1}H and 125 MHz ^{13}C{^{1}H} NMR spectra of **1d** in $CDCl_3$.

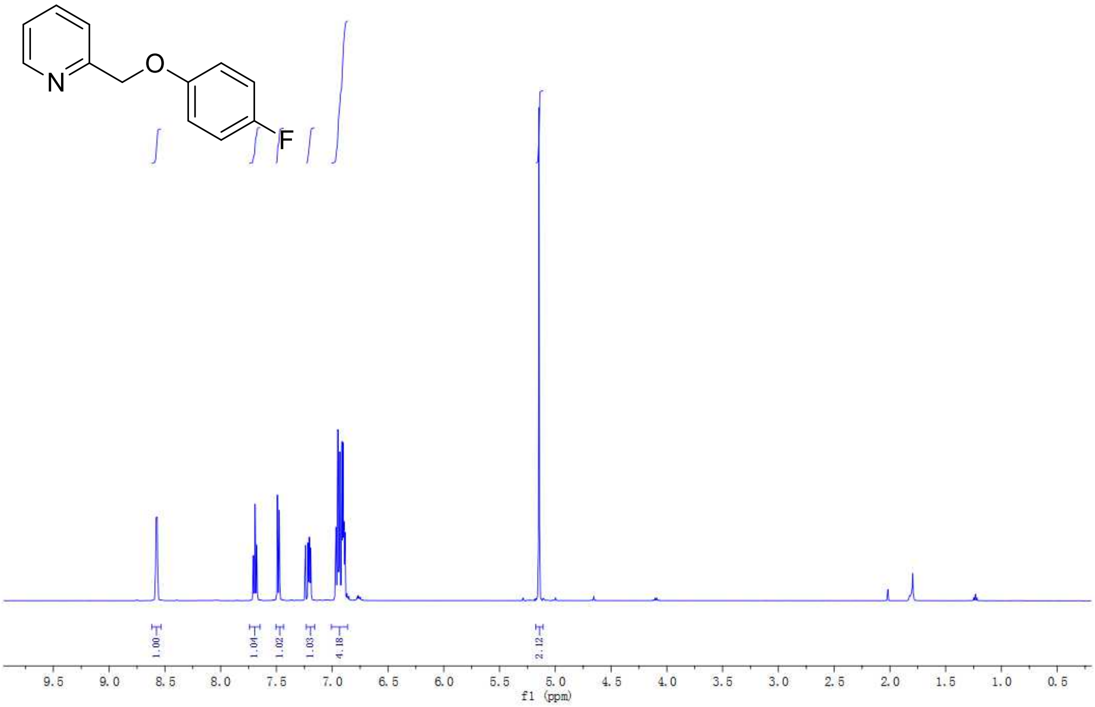

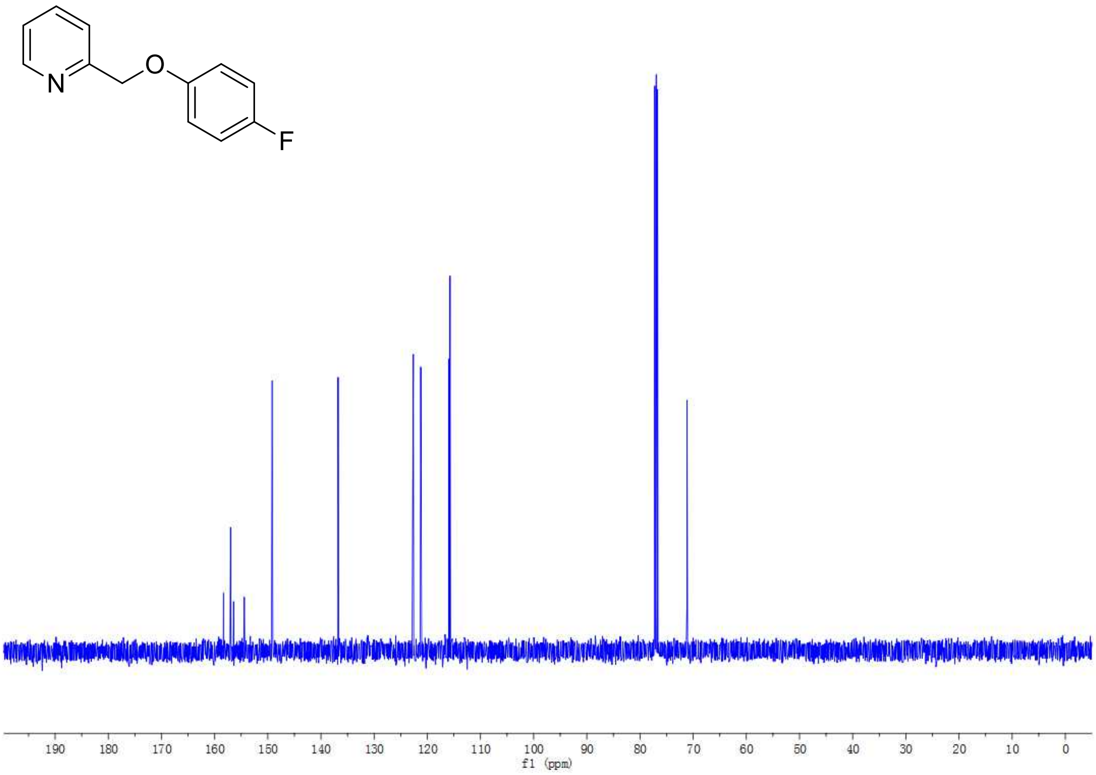

Figure S3. 500 MHz ^{1}H and 125 MHz $^{13}C\{^{1}H\}$ NMR spectra of **1f** in $CDCl_3$.

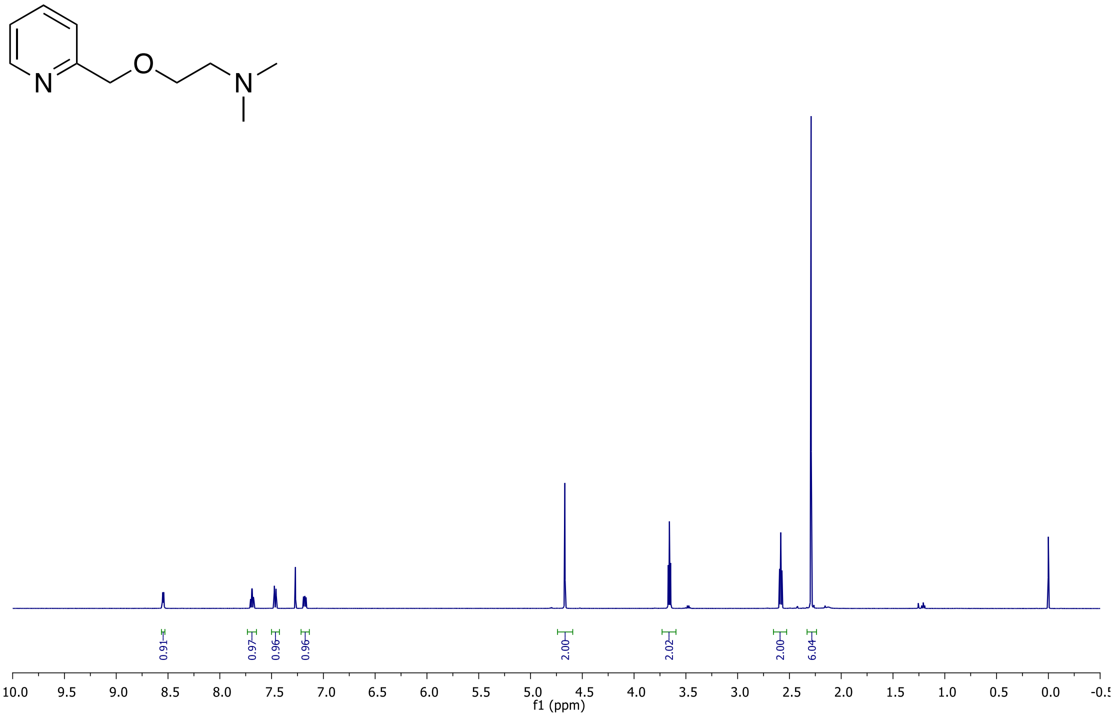

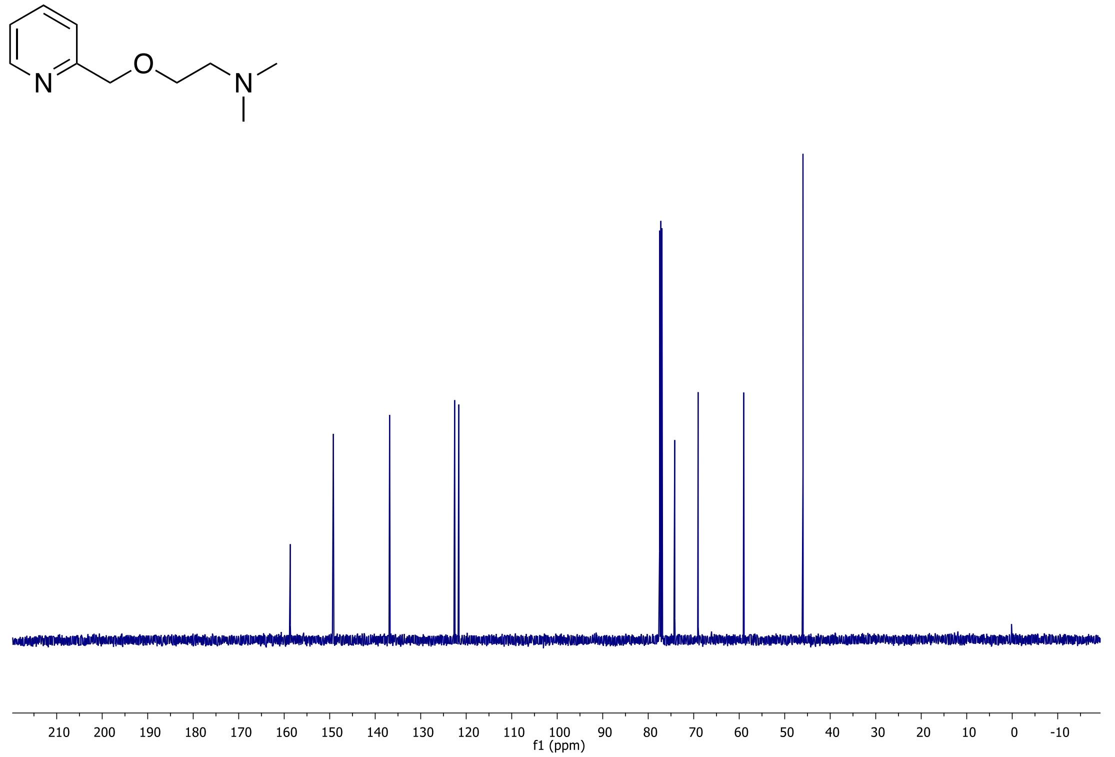

Figure S4. 500 MHz ^{1}H and 125 MHz $^{13}C\{^{1}H\}$ NMR spectra of **1h** in $CDCl_3$.

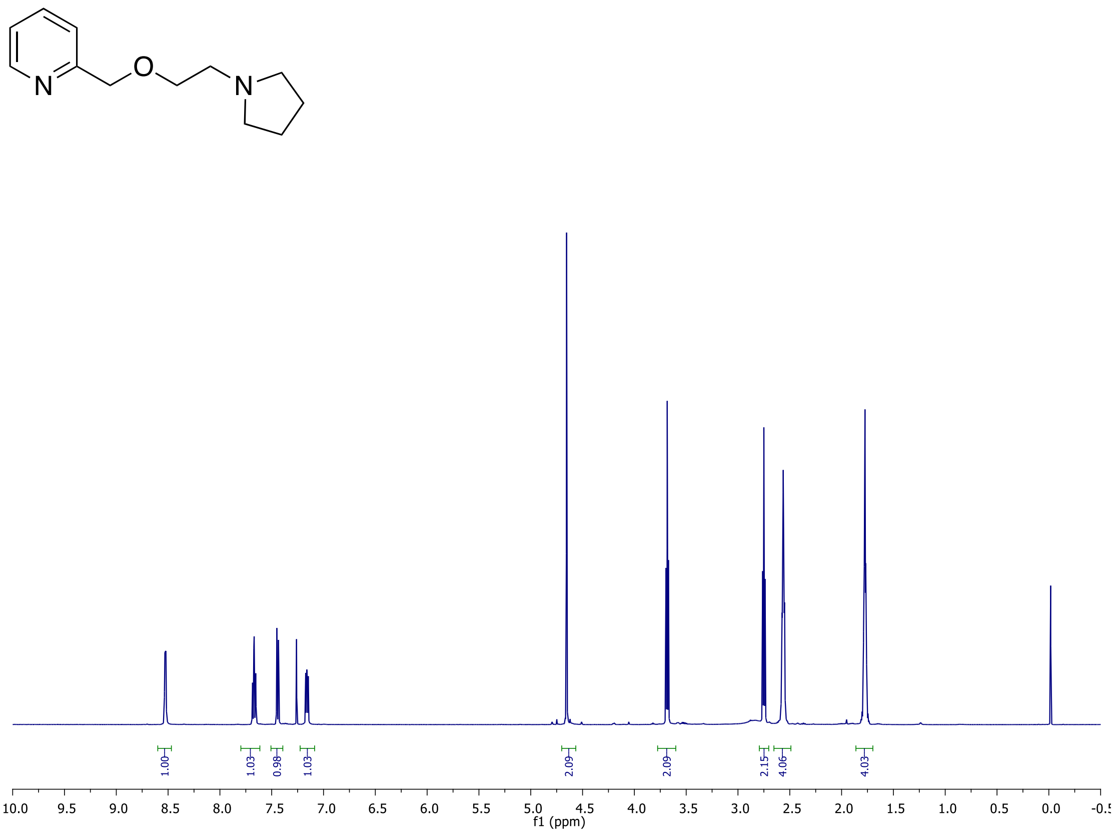

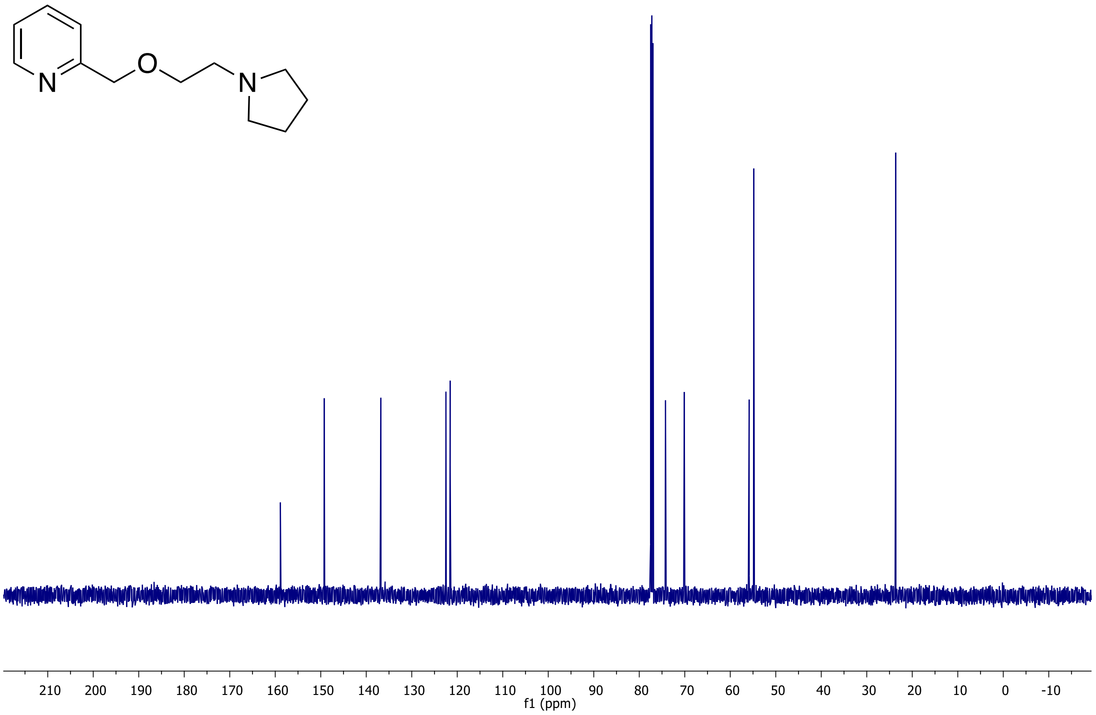

Figure S5. 500 MHz ^{1}H and 125 MHz ^{13}C{^{1}H} NMR spectra of **1i** in $CDCl_3$.

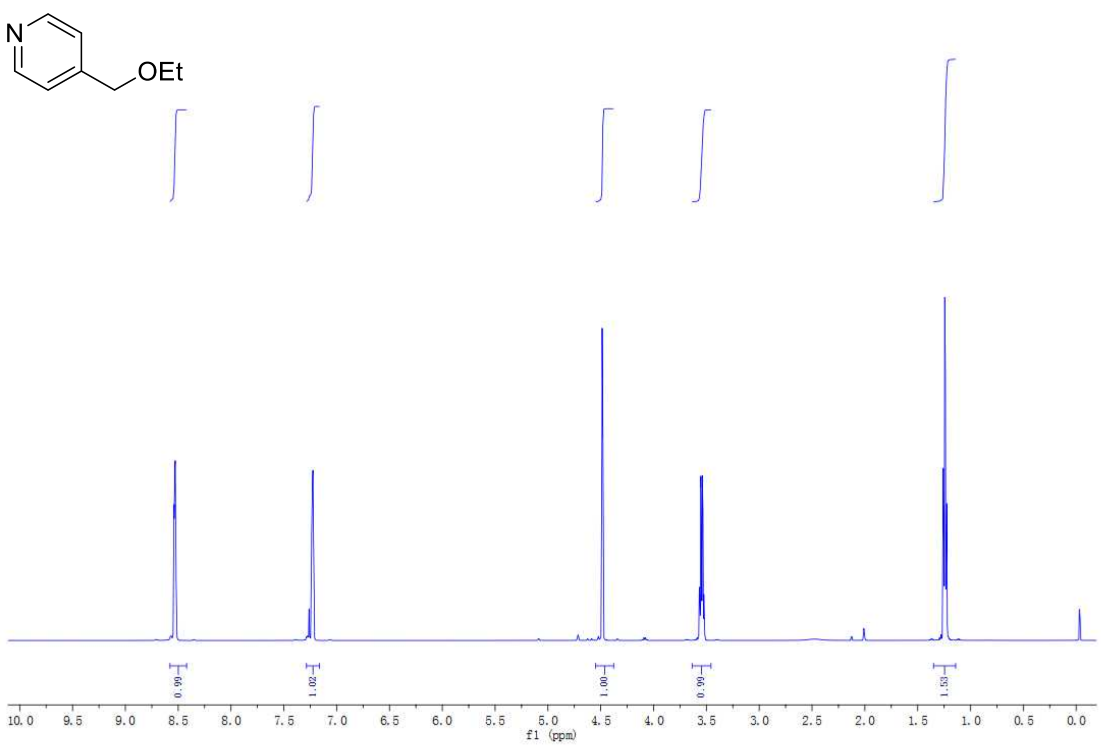

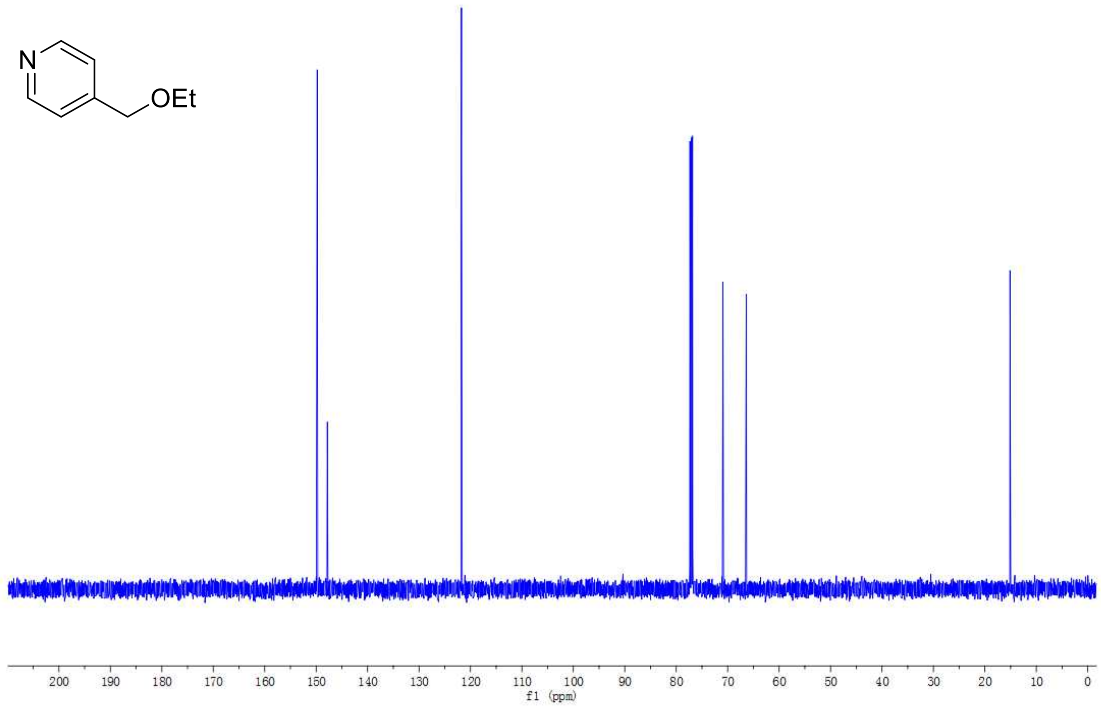

Figure S6. 500 MHz ^{1}H and 125 MHz $^{13}C\{^{1}H\}$ NMR spectra of **1j** in $CDCl_3$.

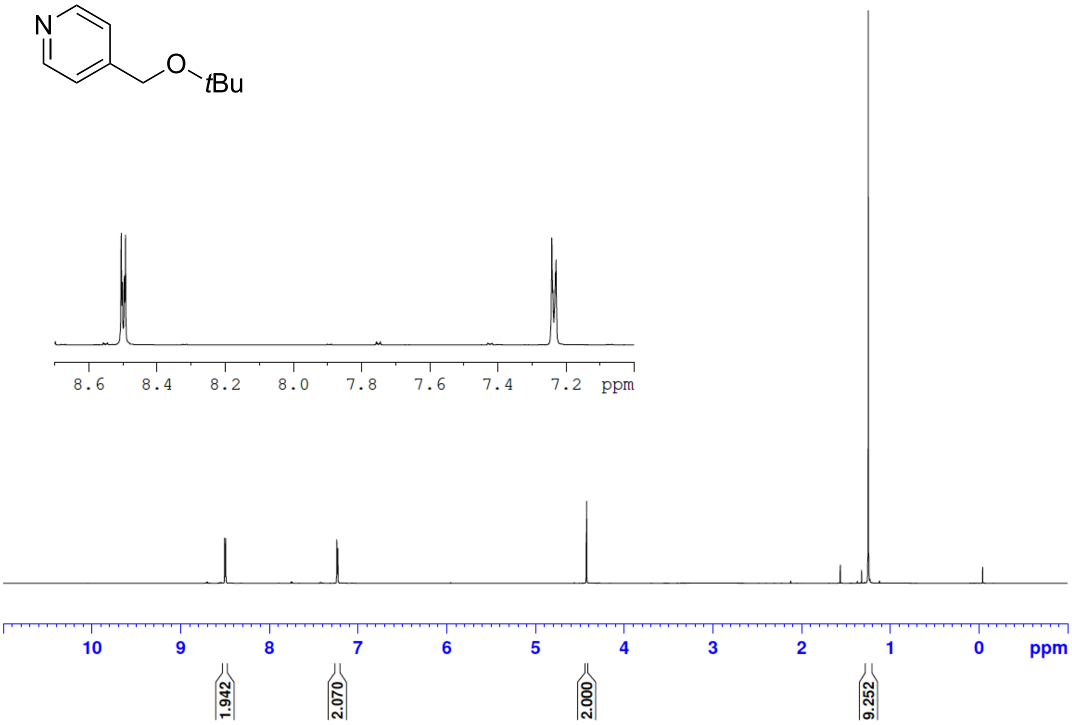

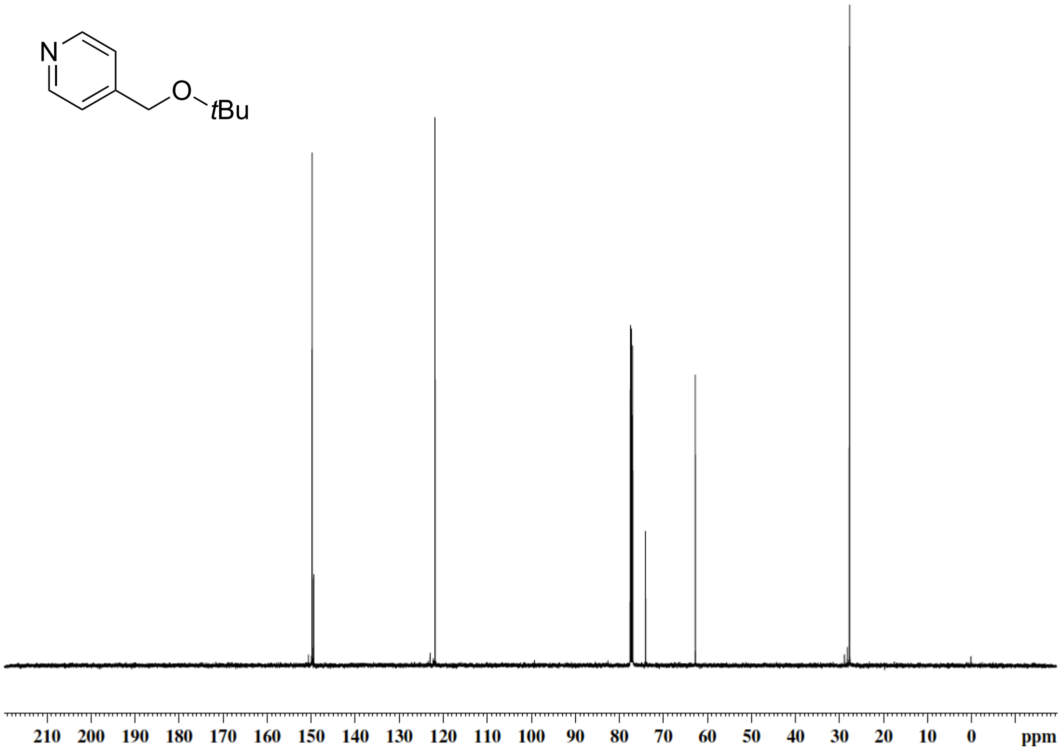

Figure S7. 500 MHz ^{1}H and 125 MHz ^{13}C{^{1}H} NMR spectra of **1k** in $CDCl_3$.

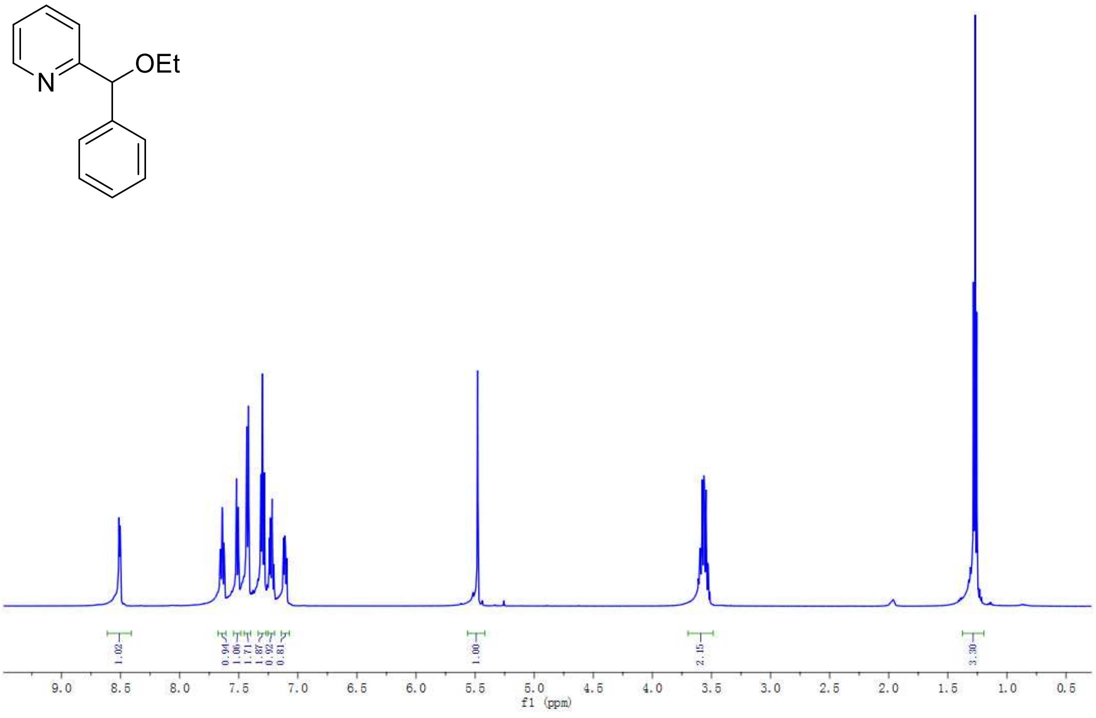

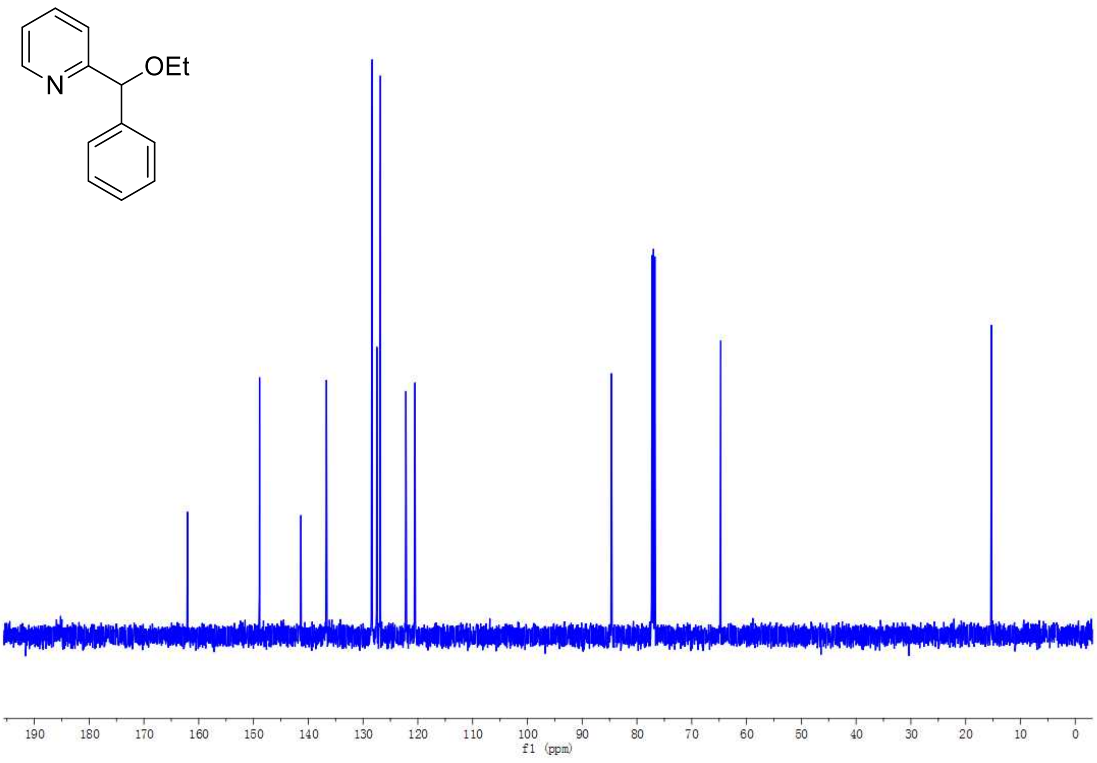

Figure S8. 500 MHz ^{1}H and 125 MHz $^{13}C\{^{1}H\}$ NMR spectra of **4aa** in $CDCl_3$.

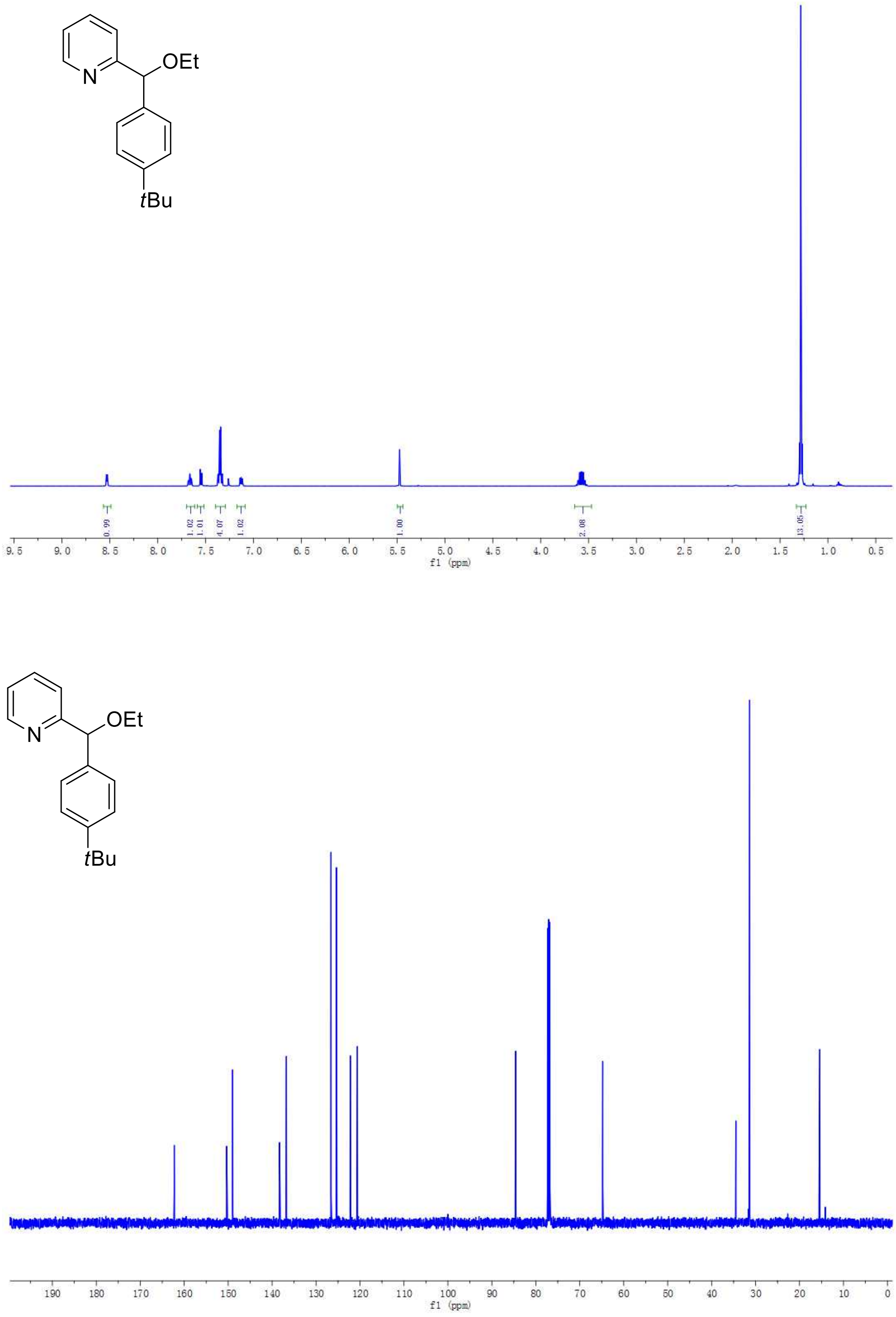

Figure S9. 500 MHz ^{1}H and 125 MHz $^{13}C\{^{1}H\}$ NMR spectra of **4ab** in $CDCl_3$.

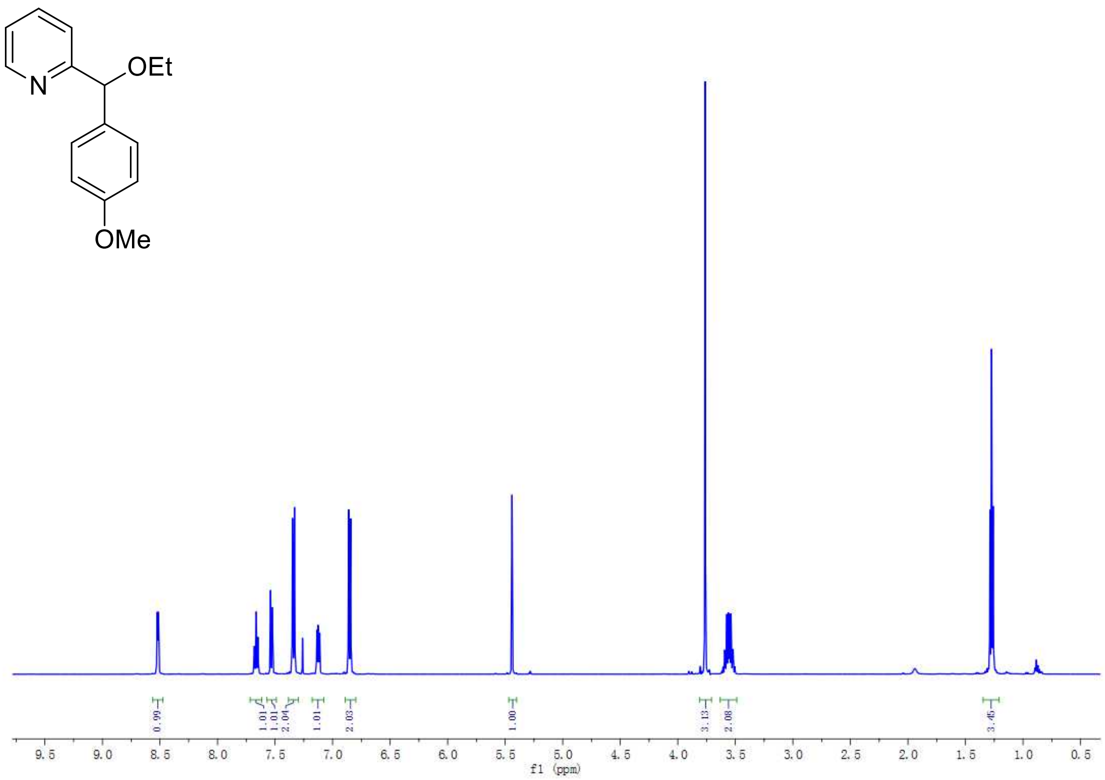

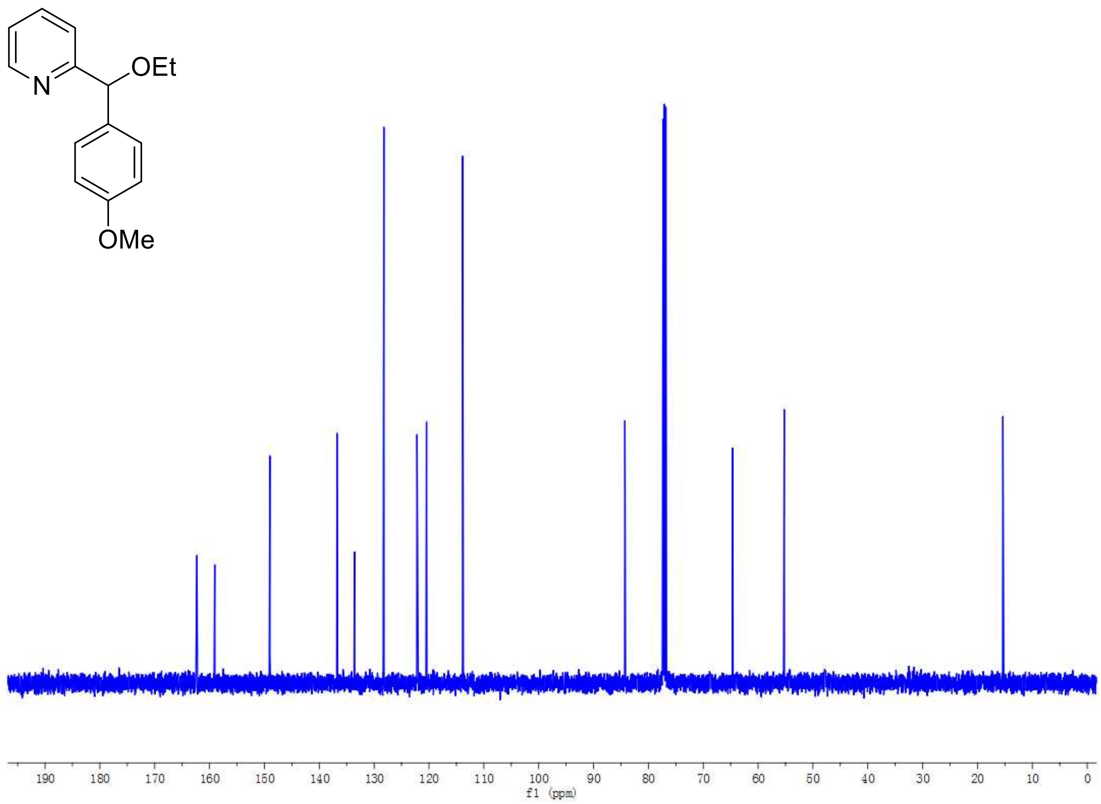

Figure S10. 500 MHz ^{1}H and 125 MHz $^{13}C\{^{1}H\}$ NMR spectra of **4ac** in $CDCl_3$.

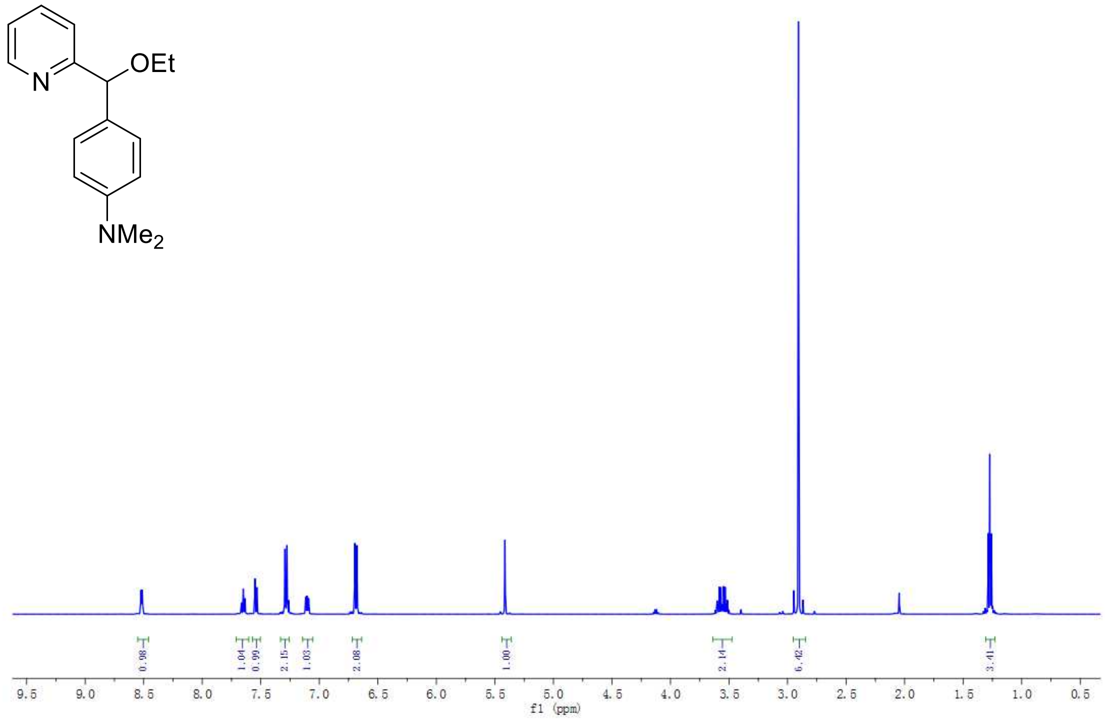

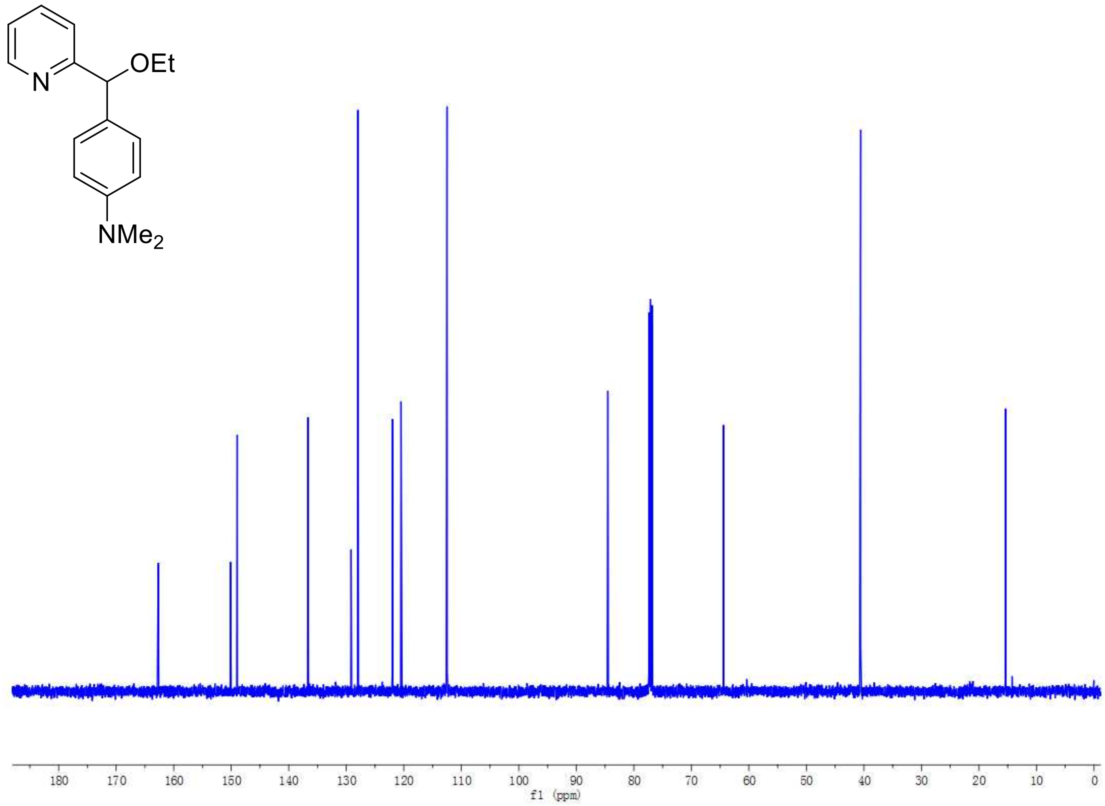

Figure S11. 500 MHz ^{1}H and 125 MHz $^{13}C\{^{1}H\}$ NMR spectra of **4ad** in $CDCl_3$.

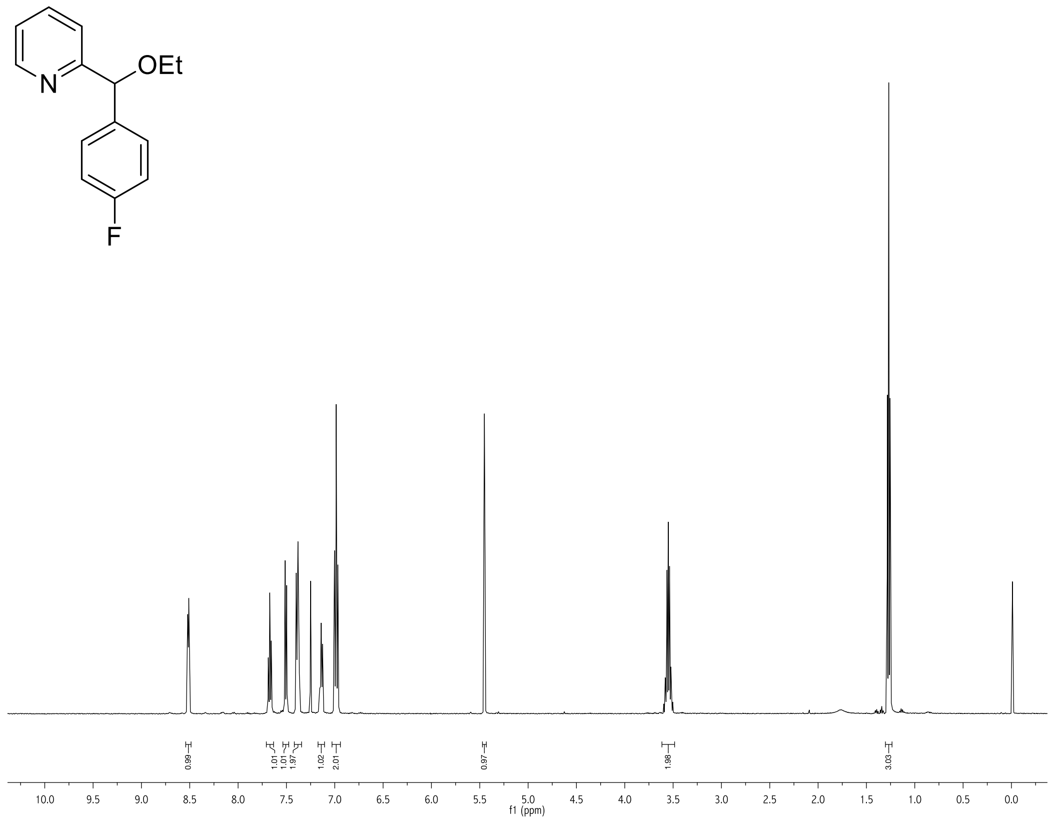

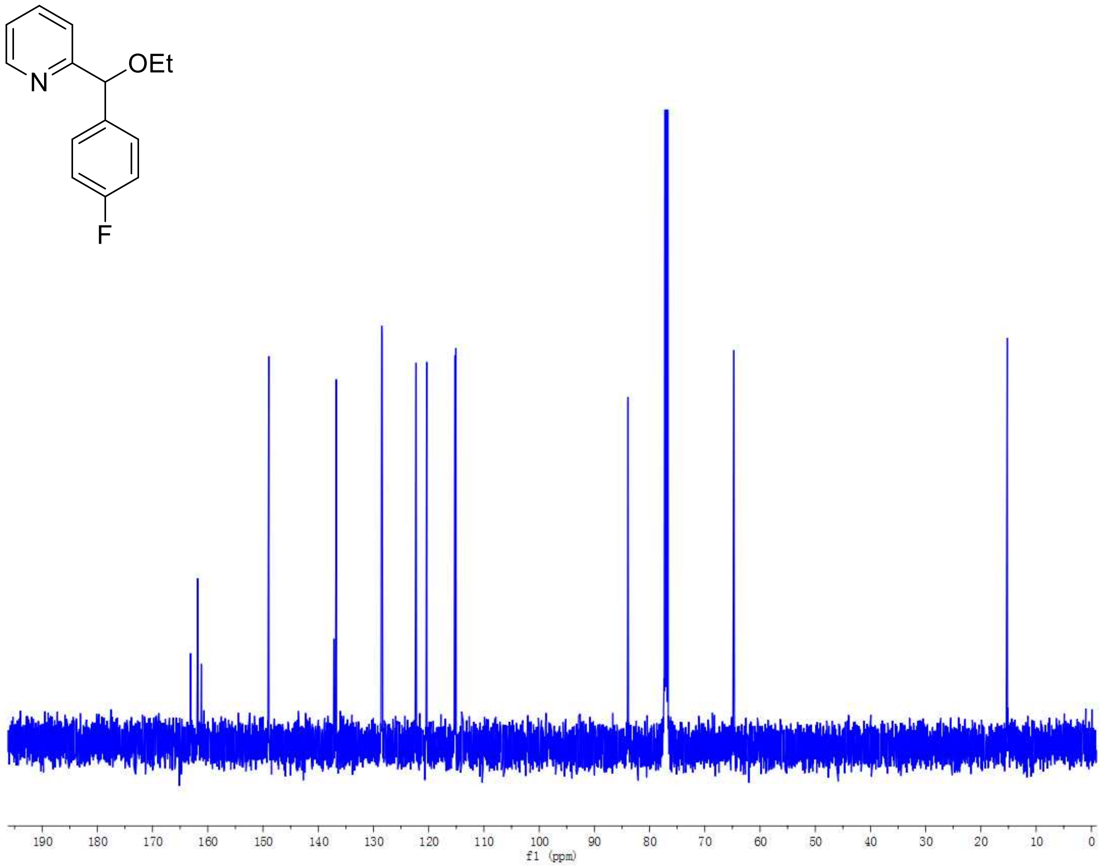

Figure S12. 500 MHz ^{1}H and 125 MHz $^{13}C\{^{1}H\}$ NMR spectra of **4ae** in $CDCl_3$.

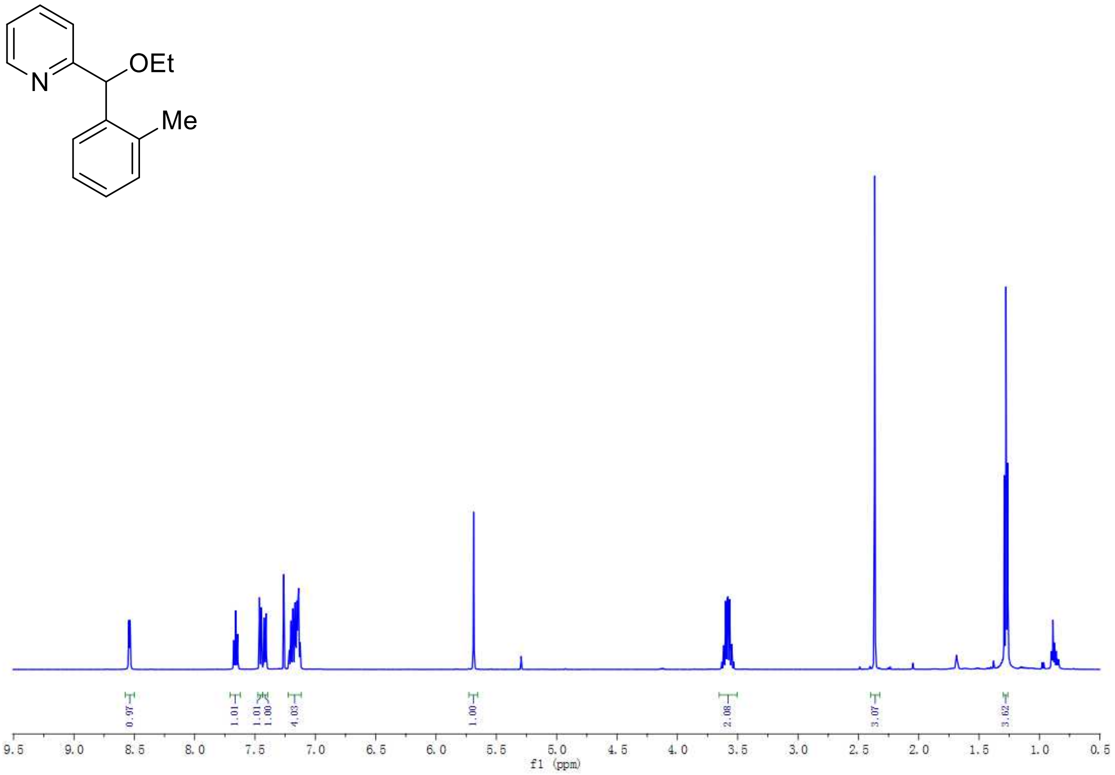

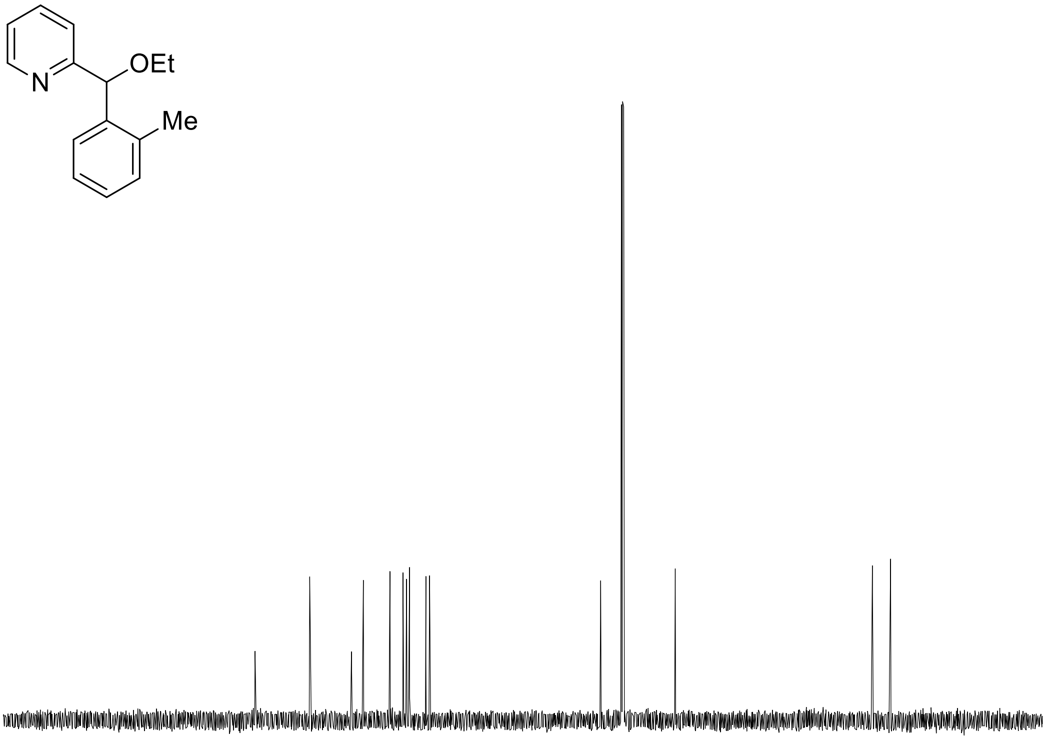

Figure S13. 500 MHz ^{1}H and 125 MHz $^{13}C\{^{1}H\}$ NMR spectra of **4af** in $CDCl_3$.

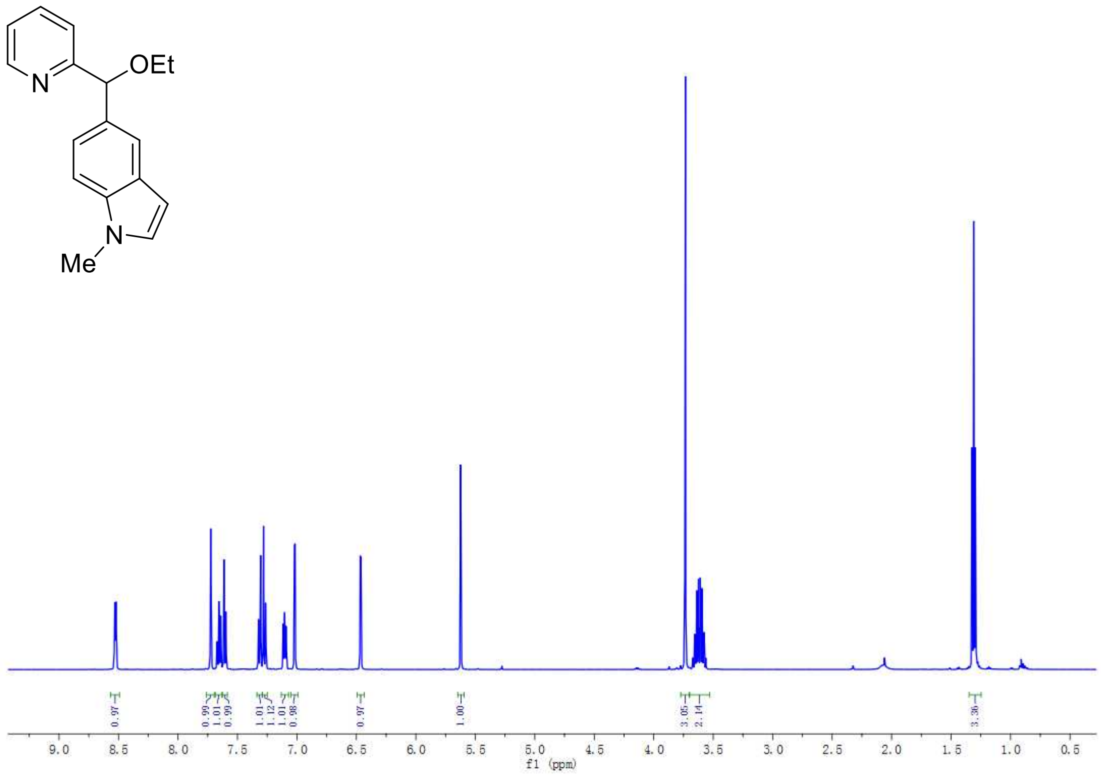

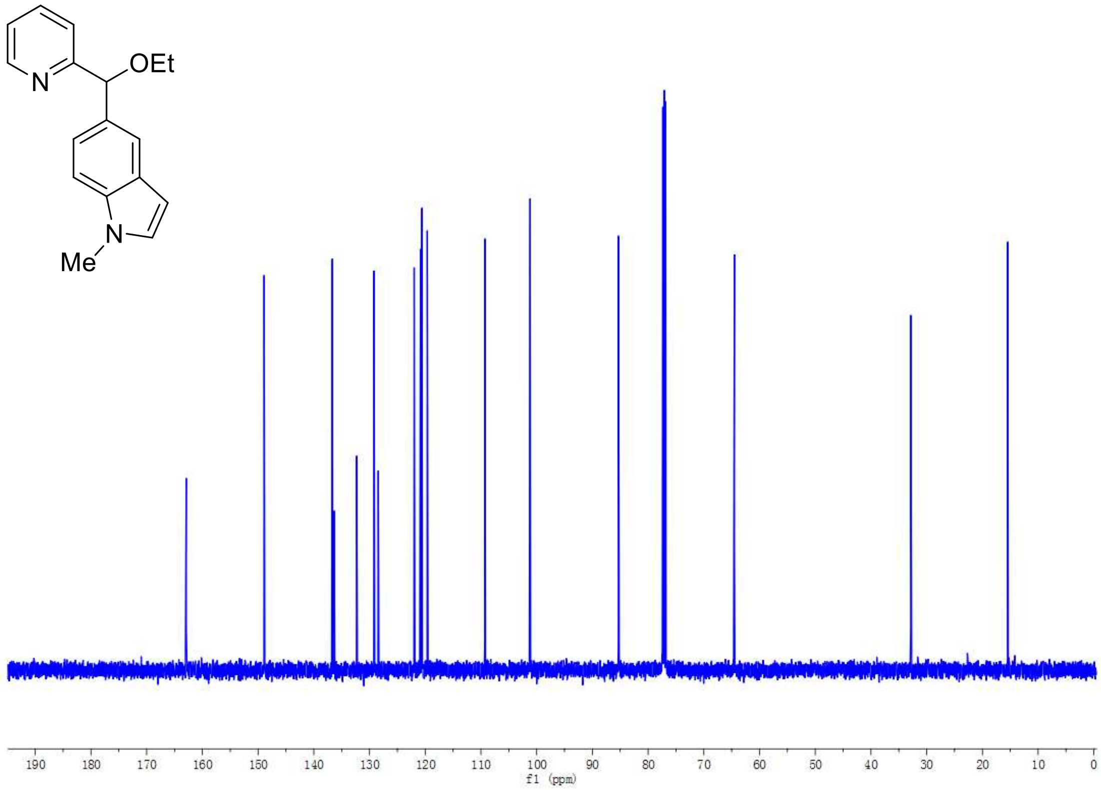

Figure S14. 500 MHz ^{1}H and 125 MHz ^{13}C{^{1}H} NMR spectra of **4ag** in $CDCl_3$.

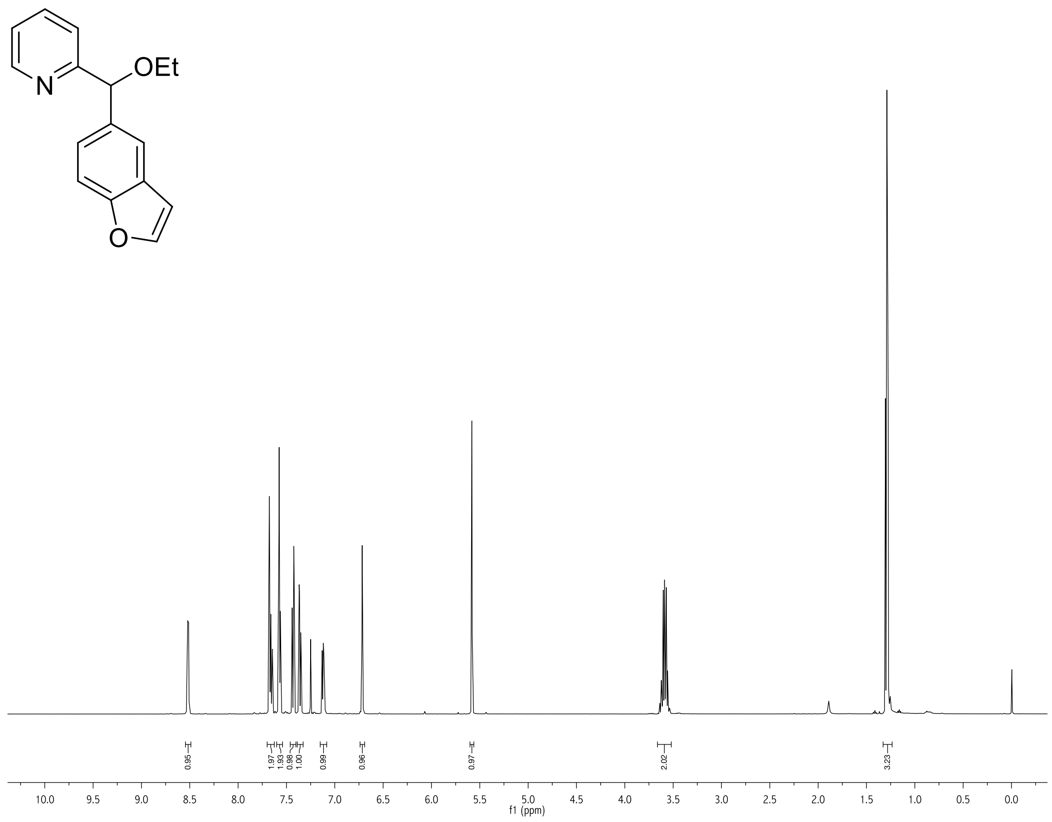

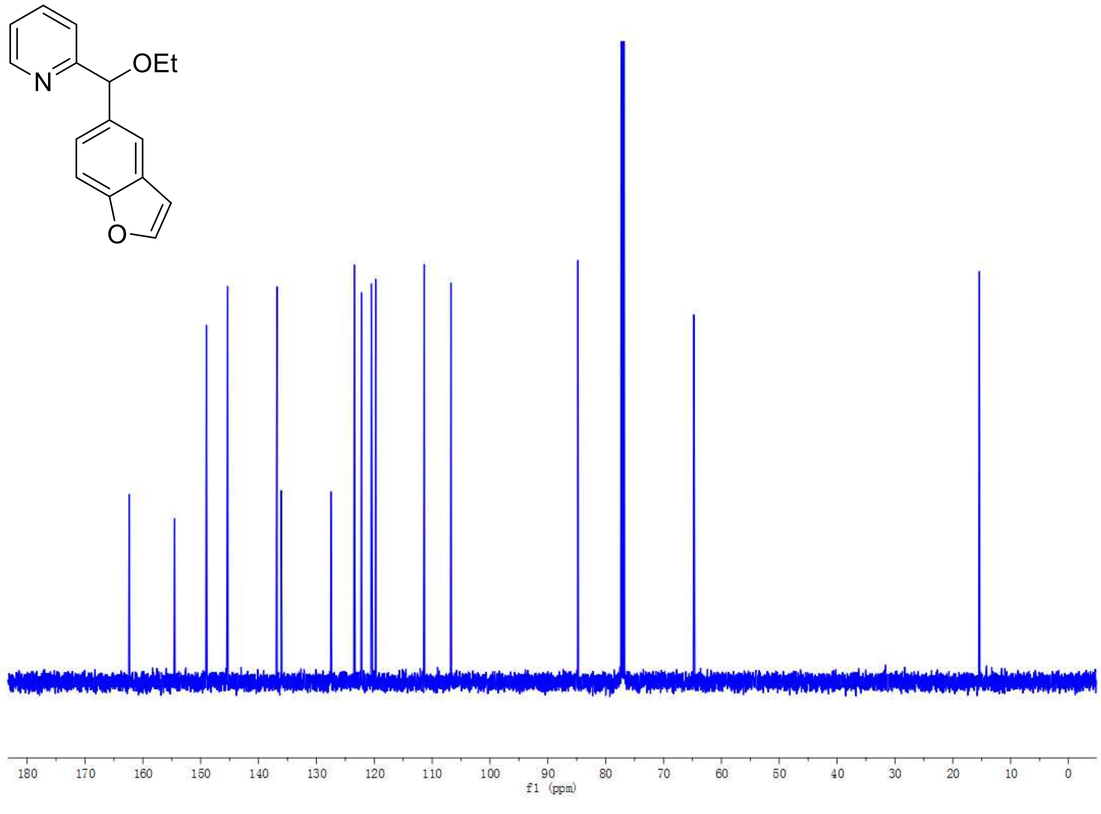

Figure S15. 500 MHz ^{1}H and 125 MHz ^{13}C{^{1}H} NMR spectra of **4ah** in $CDCl_3$.

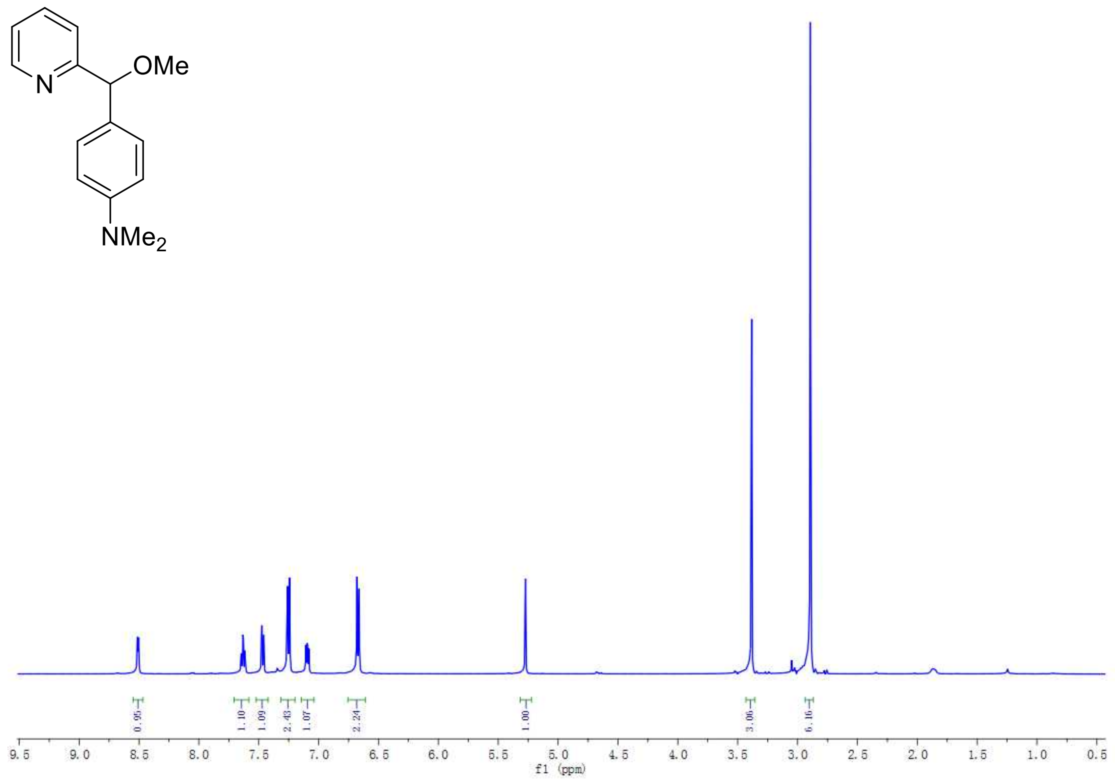

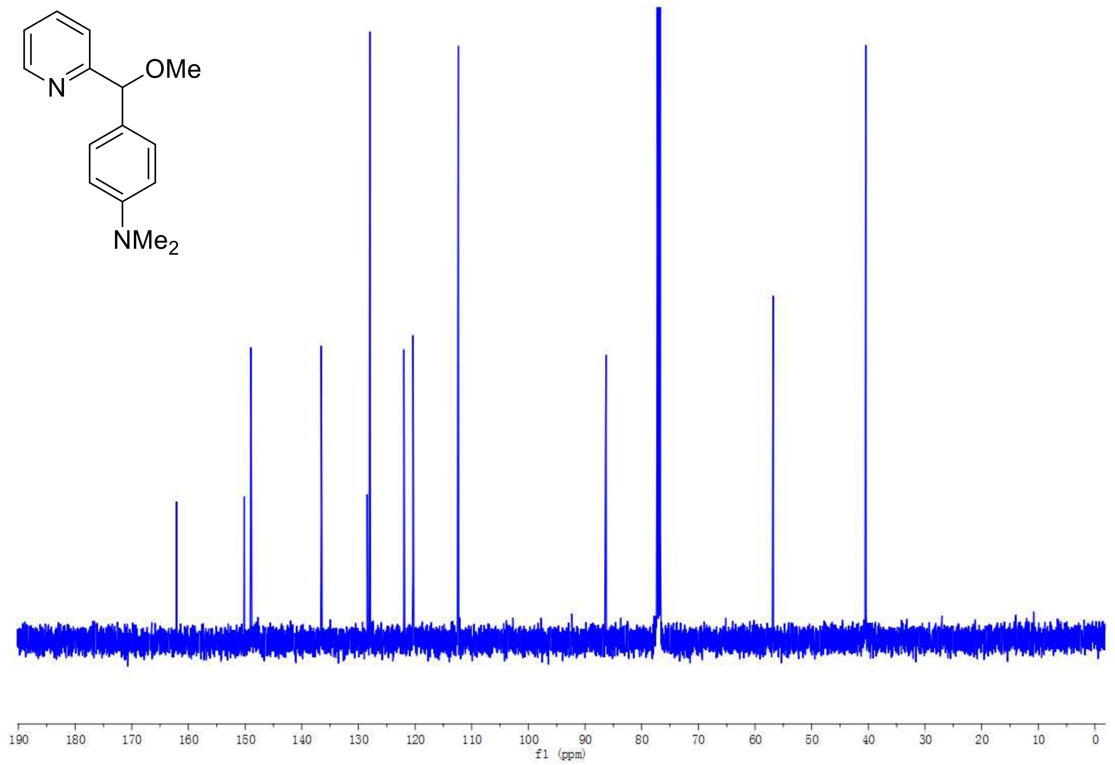

Figure S16. 500 MHz ^{1}H and 125 MHz $^{13}C\{^{1}H\}$ NMR spectra of **4bd** in $CDCl_3$.

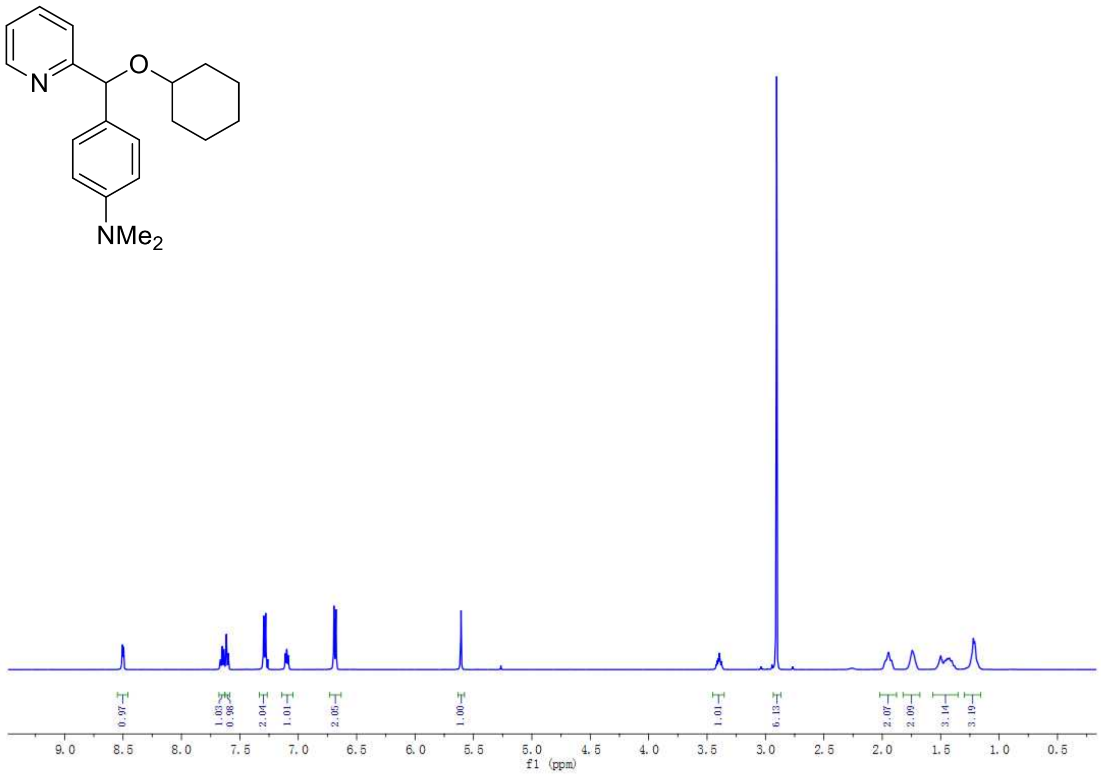

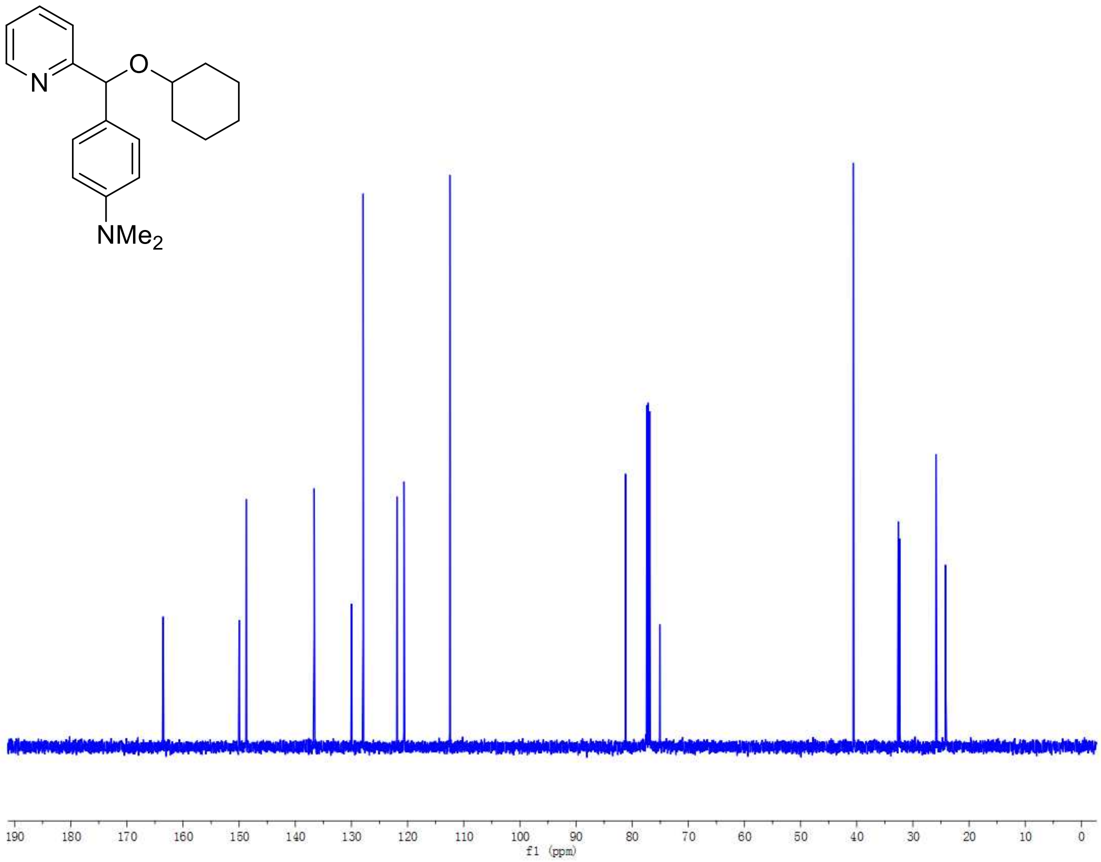

Figure S17. 500 MHz ^{1}H and 125 MHz $^{13}C\{^{1}H\}$ NMR spectra of **4cd** in $CDCl_3$.

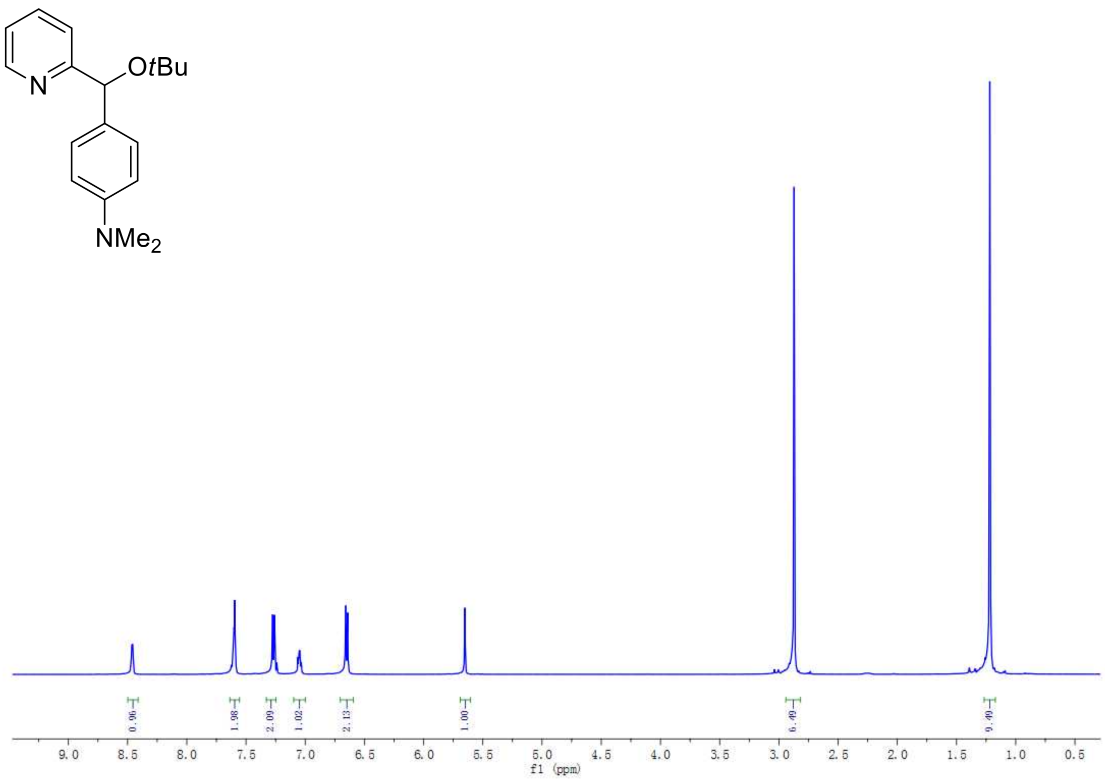

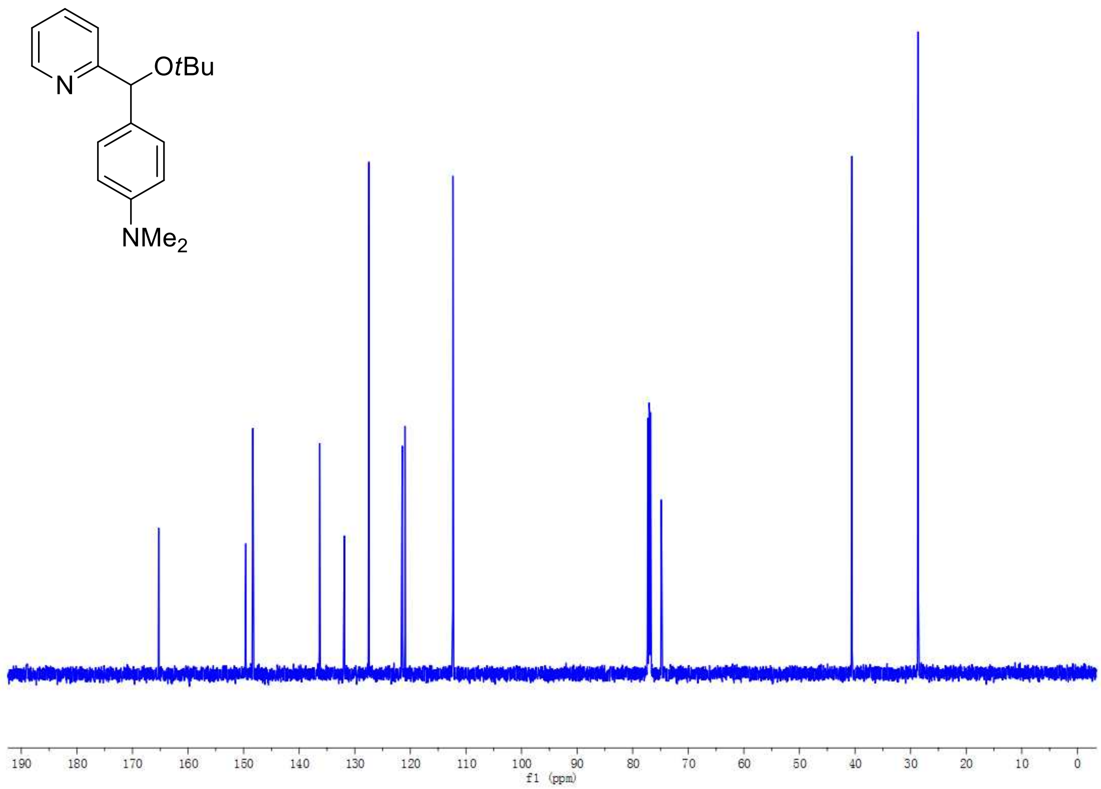

Figure S18. 500 MHz ^{1}H and 125 MHz ^{13}C{^{1}H} NMR spectra of **4dd** in $CDCl_3$.

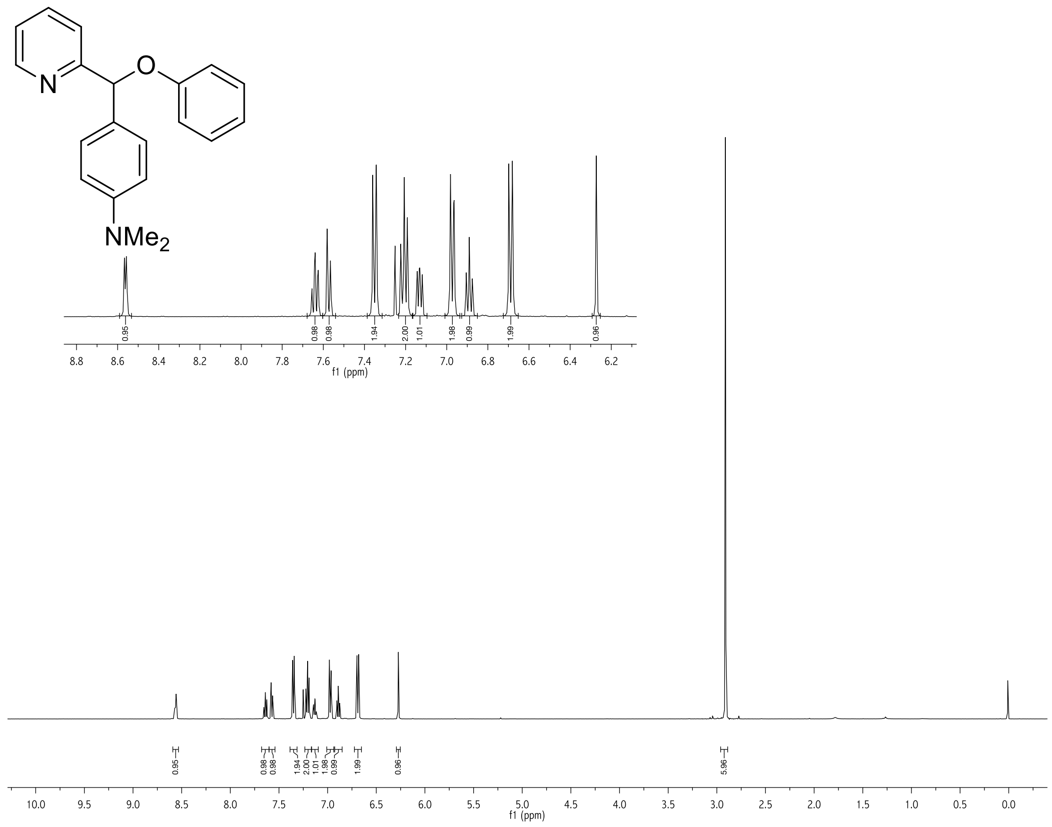

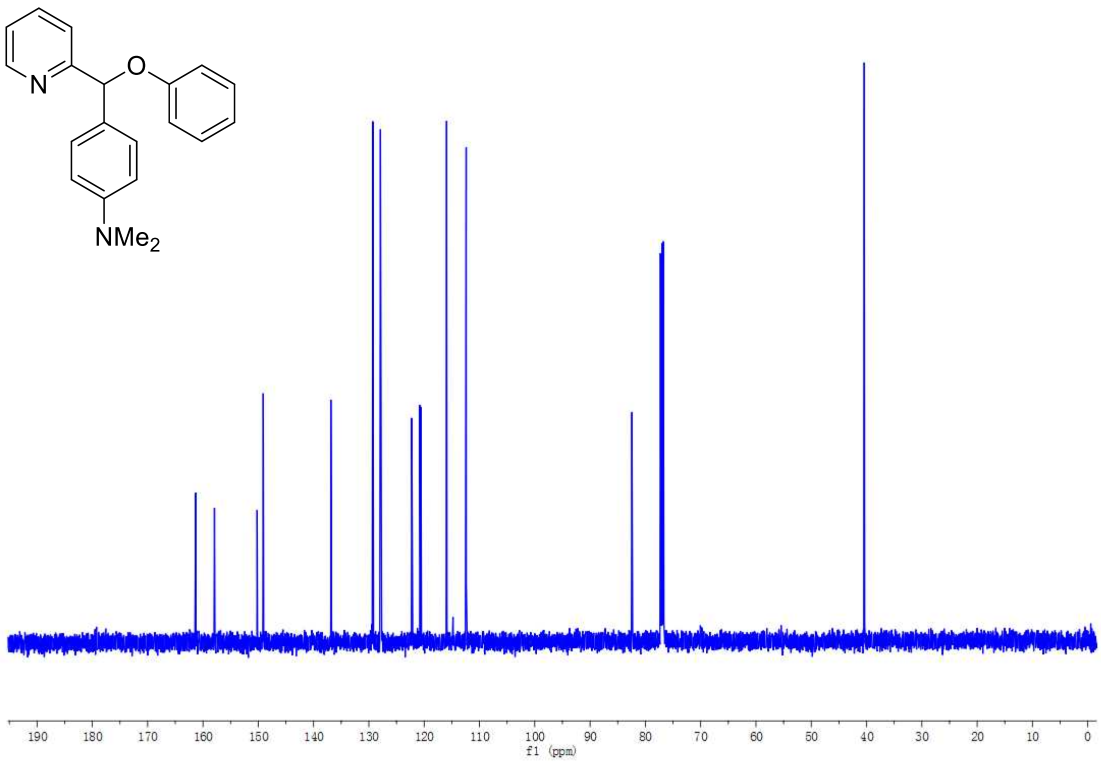

Figure S19. 500 MHz ^{1}H and 125 MHz ^{13}C{^{1}H} NMR spectra of **4ed** in $CDCl_3$

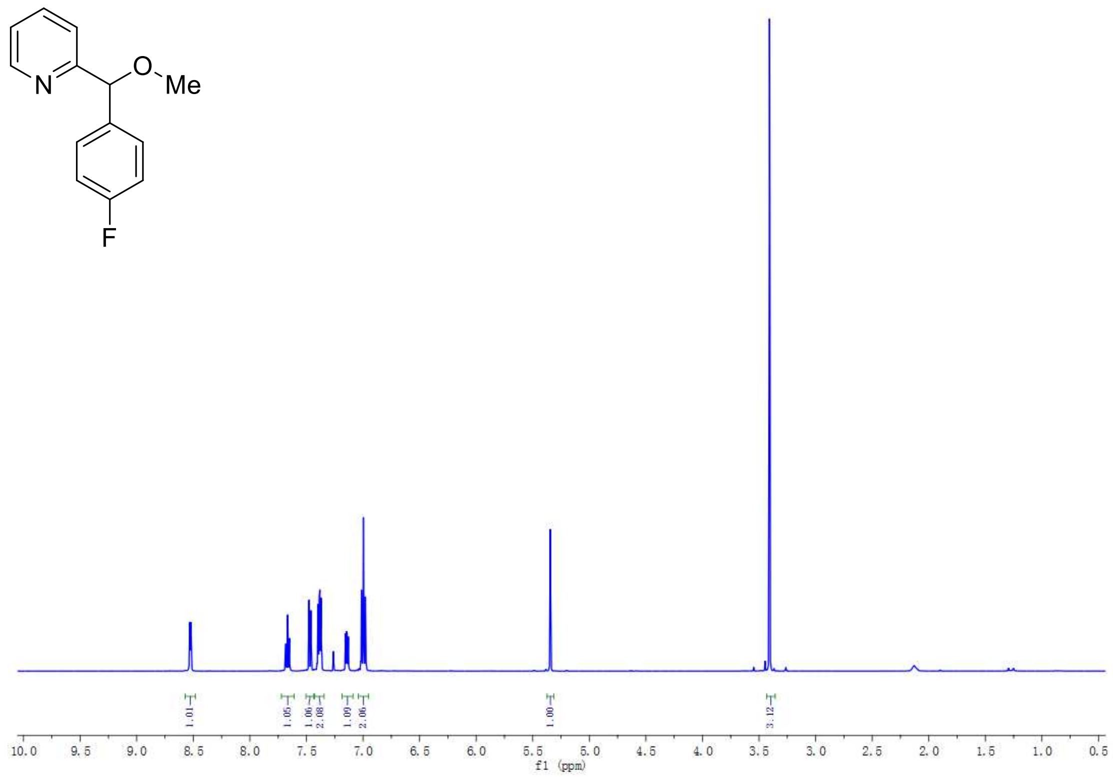

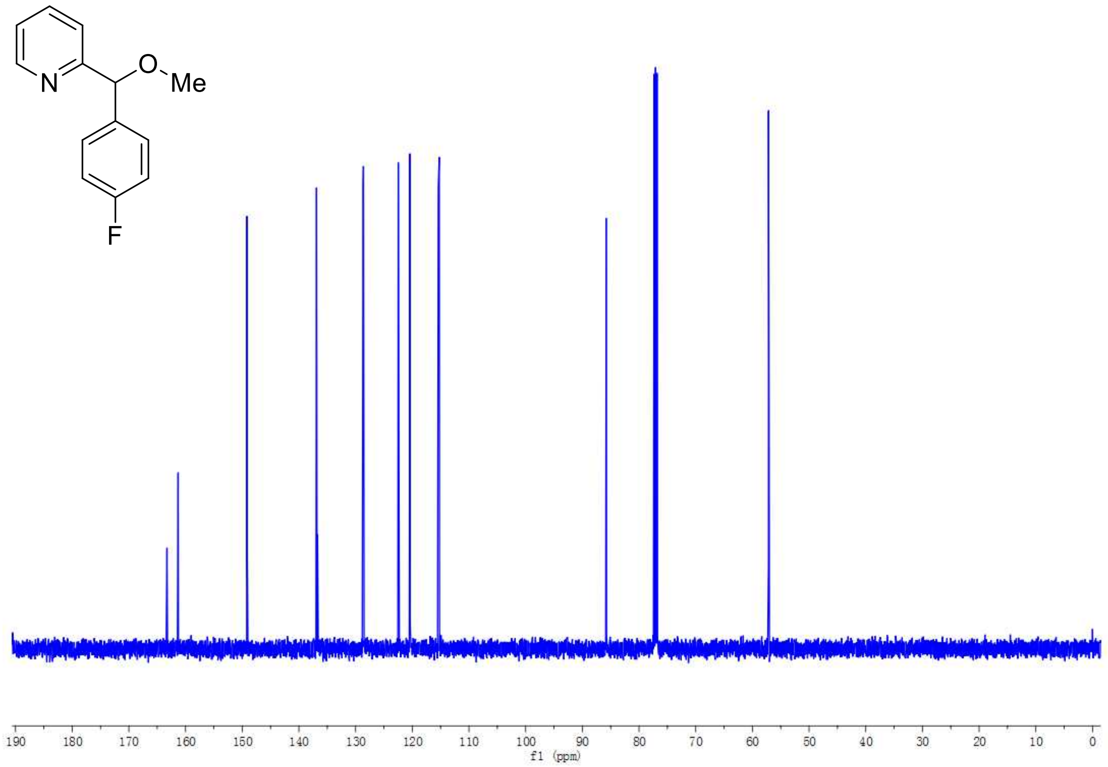

Figure S20. 500 MHz ^{1}H and 125 MHz $^{13}C\{^{1}H\}$ NMR spectra of **4be** in $CDCl_3$

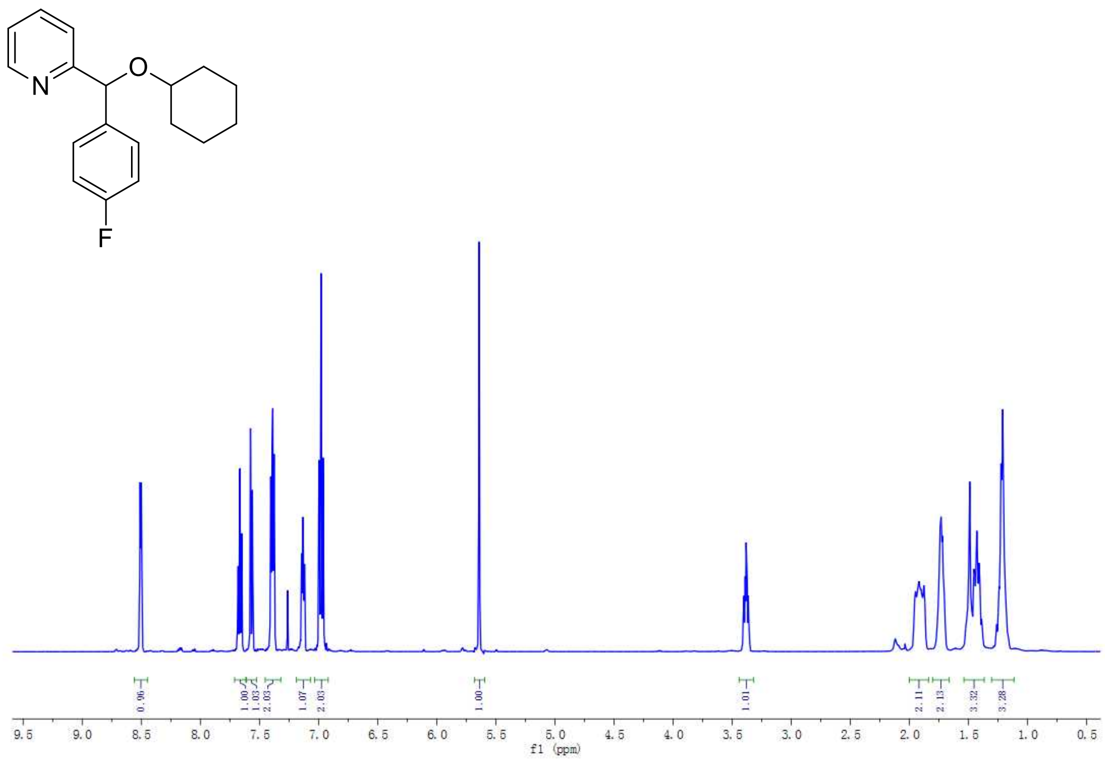

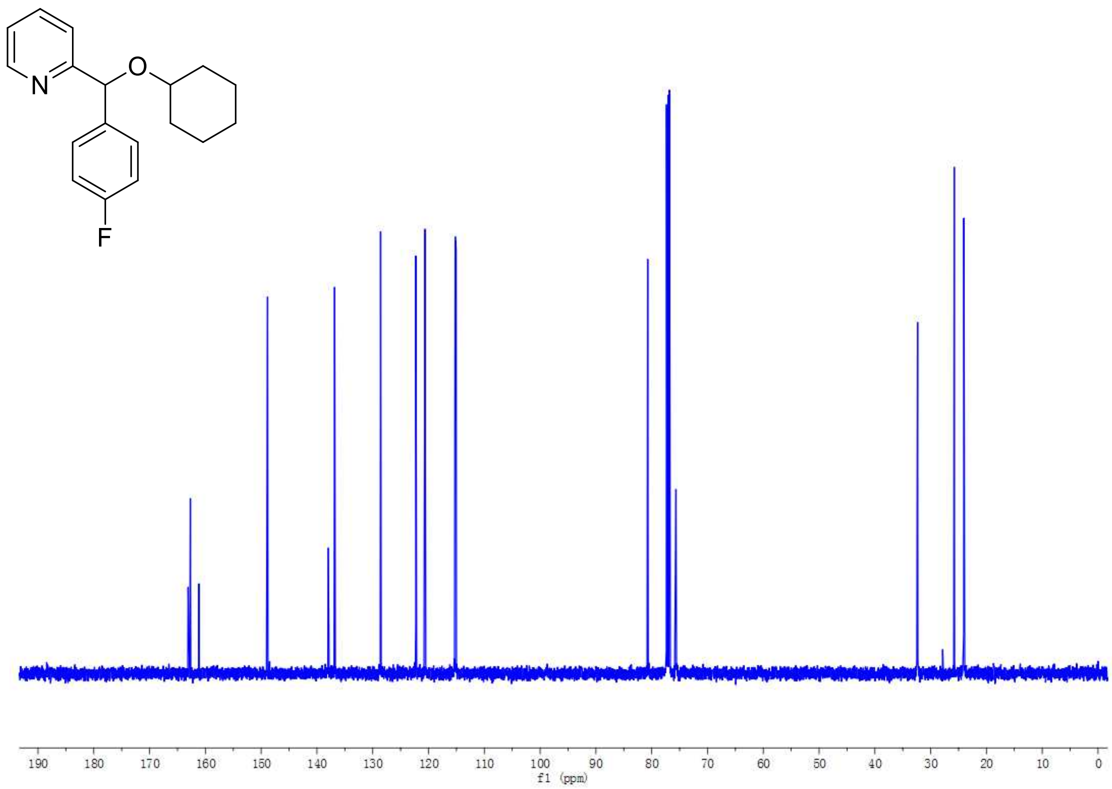

Figure S21. 500 MHz ^{1}H and 125 MHz ^{13}C{^{1}H} NMR spectra of **4ce** in $CDCl_3$

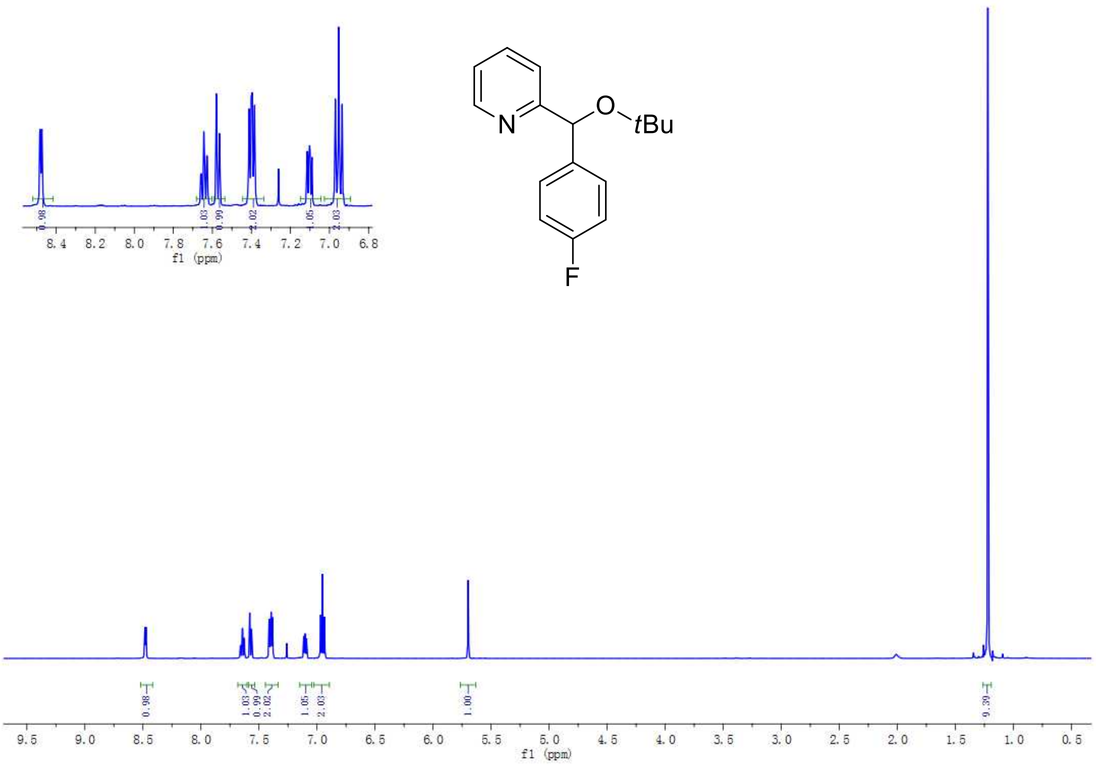

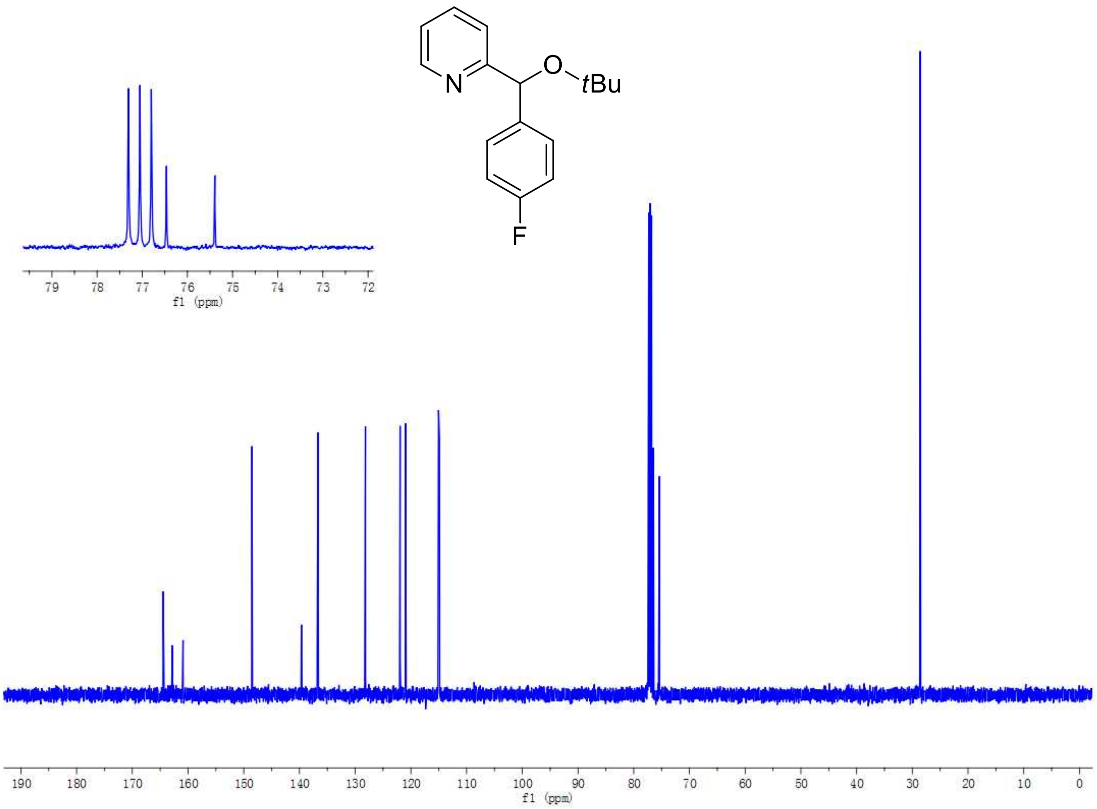

Figure S22. 500 MHz ^{1}H and 125 MHz ^{13}C{^{1}H} NMR spectra of **4de** in $CDCl_3$

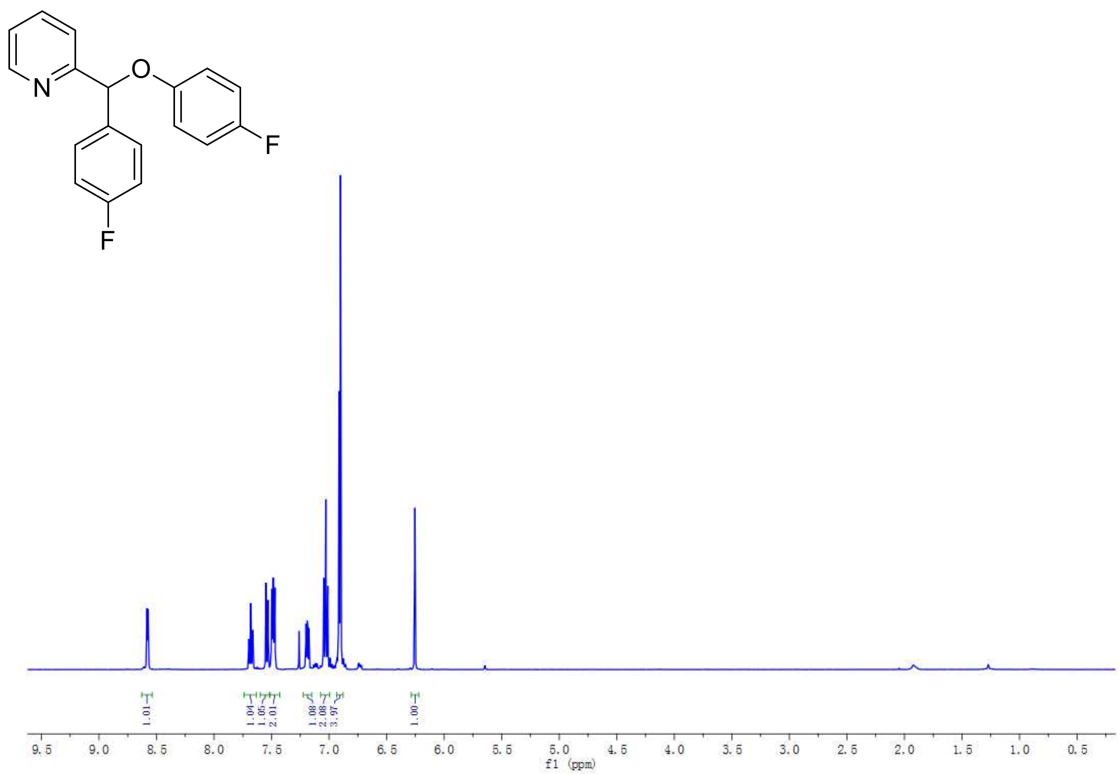

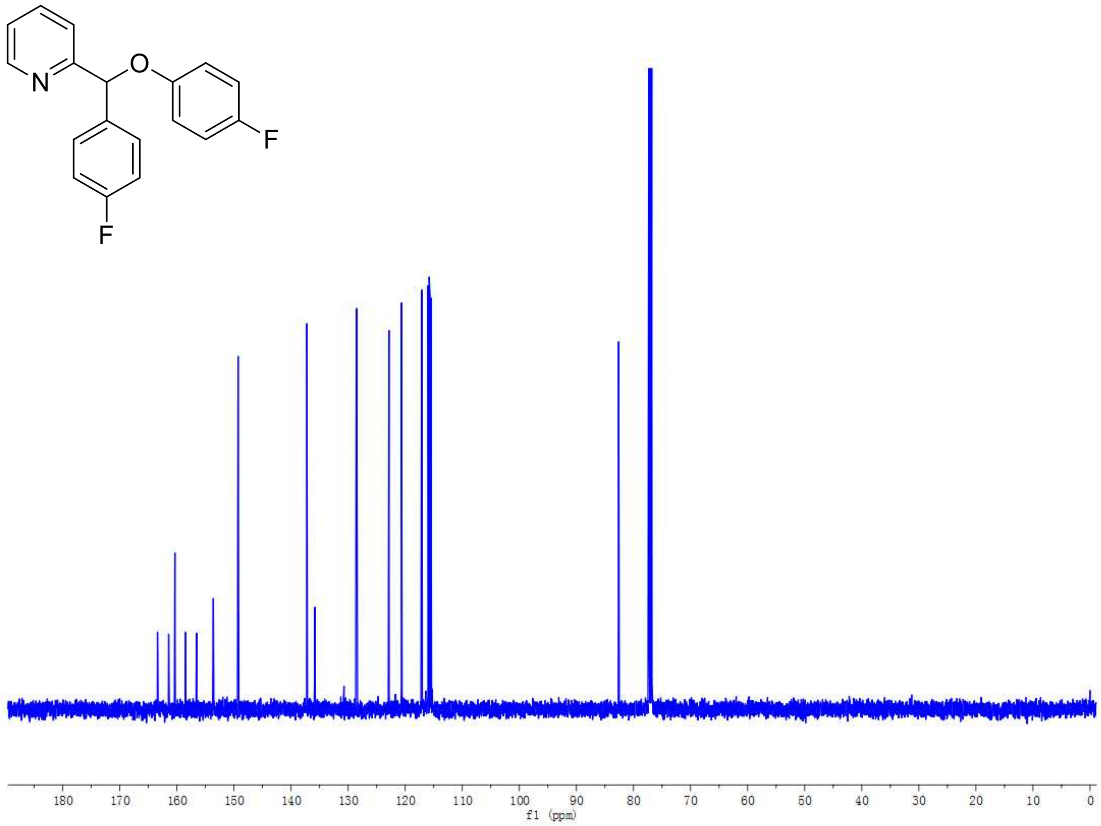

Figure S23. 500 MHz ^{1}H and 125 MHz $^{13}C\{^{1}H\}$ NMR spectra of **4fe** in $CDCl_3$

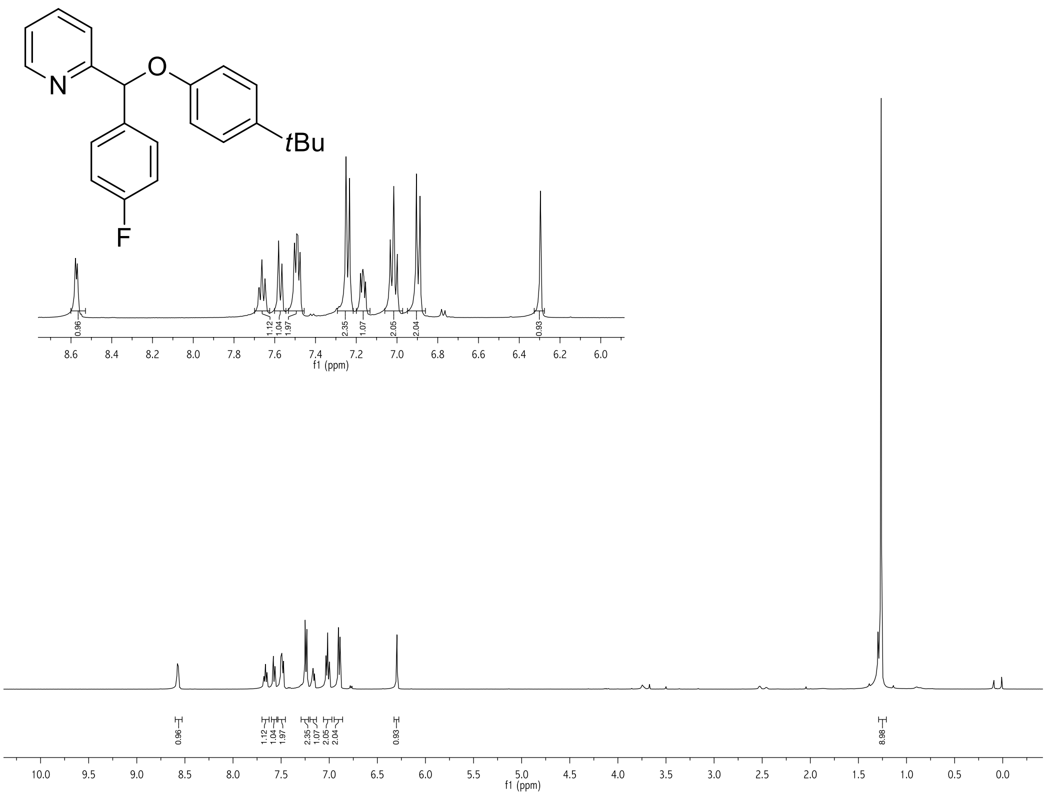

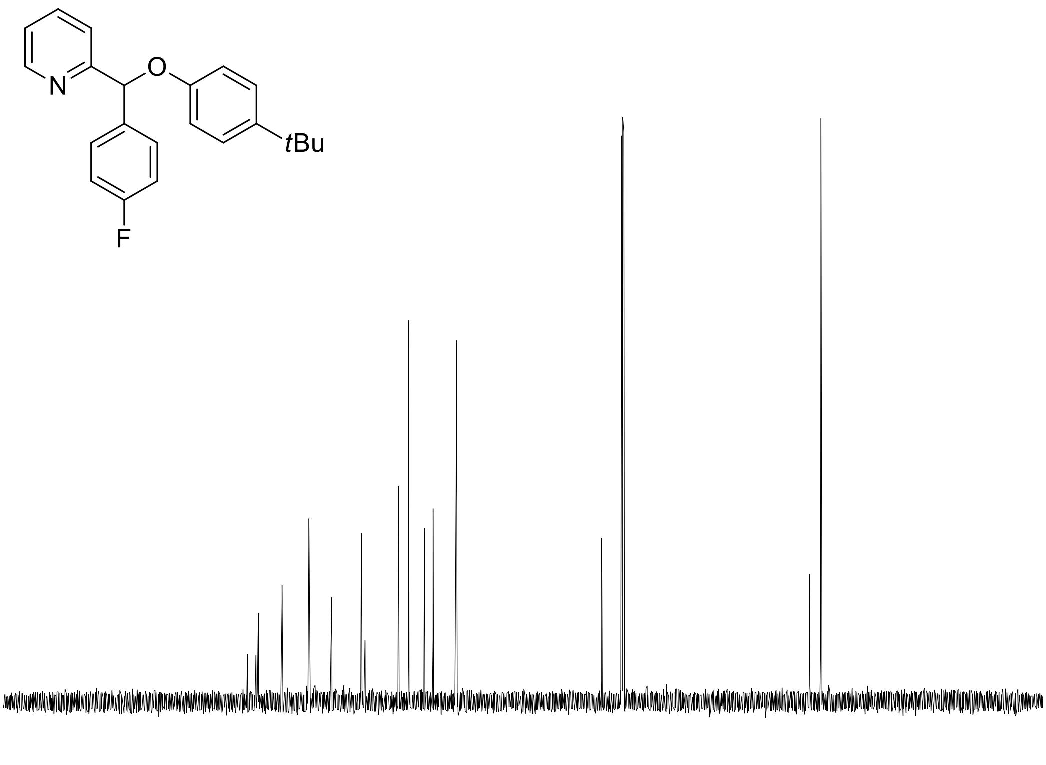

Figure S24. 500 MHz ^{1}H and 125 MHz $^{13}C\{^{1}H\}$ NMR spectra of **4ge** in $CDCl_3$

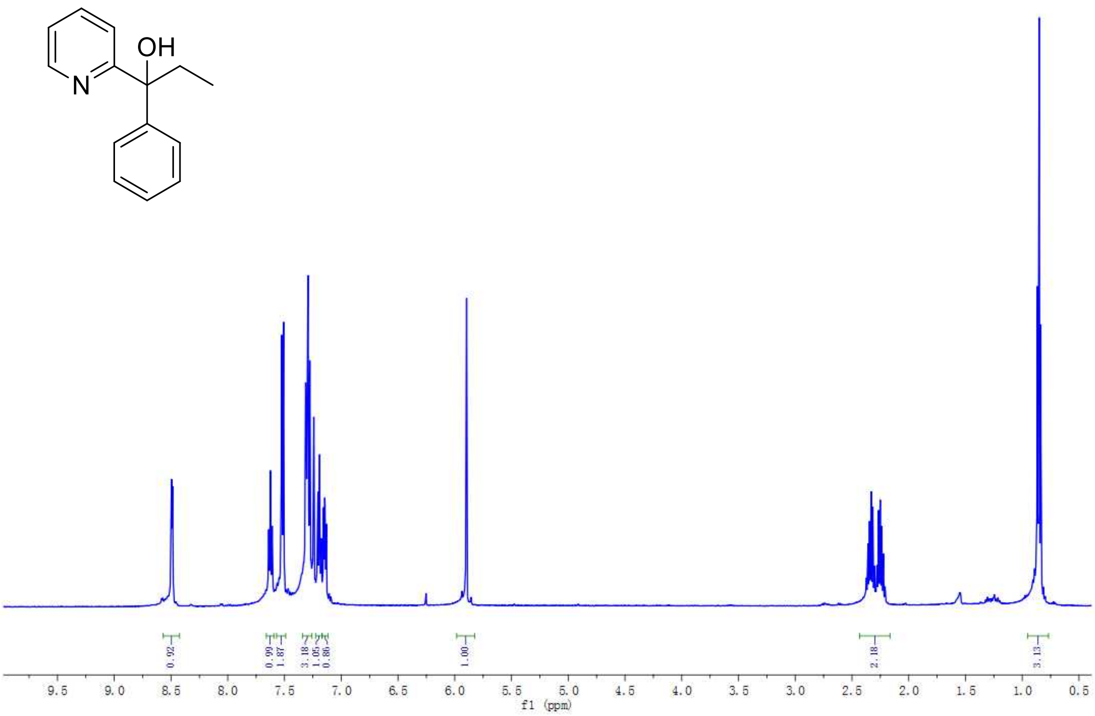

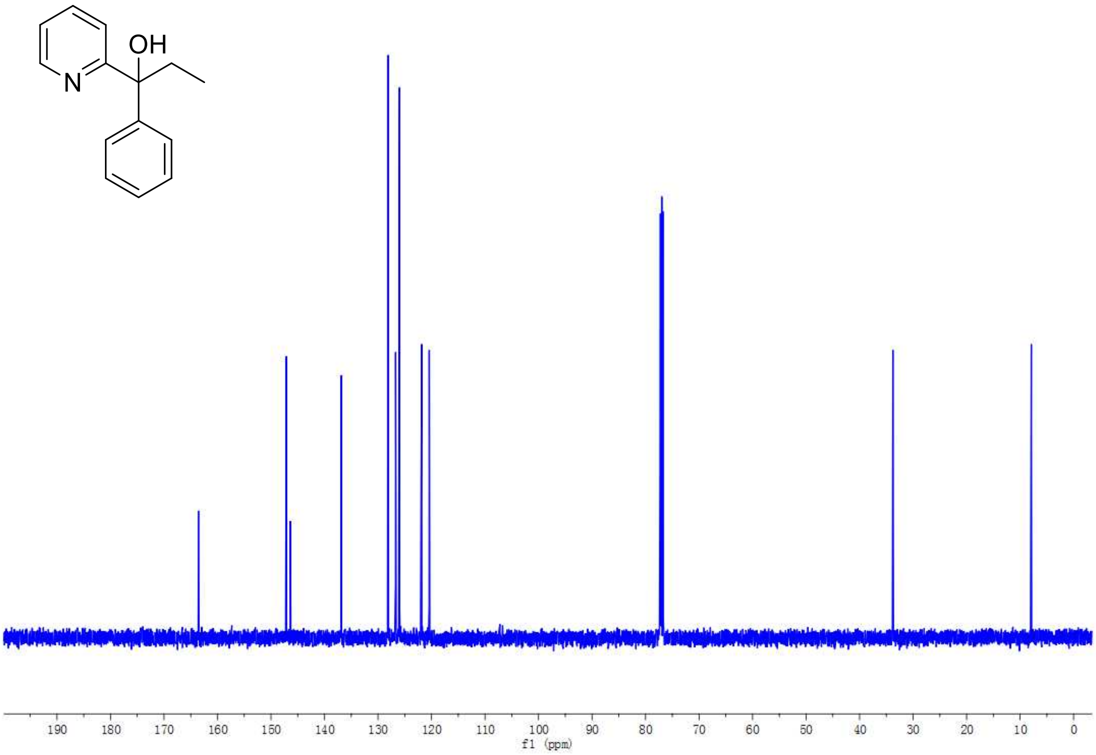

Figure S25. 500 MHz ^{1}H and 125 MHz ^{13}C{^{1}H} NMR spectra of **5aa** in $CDCl_3$

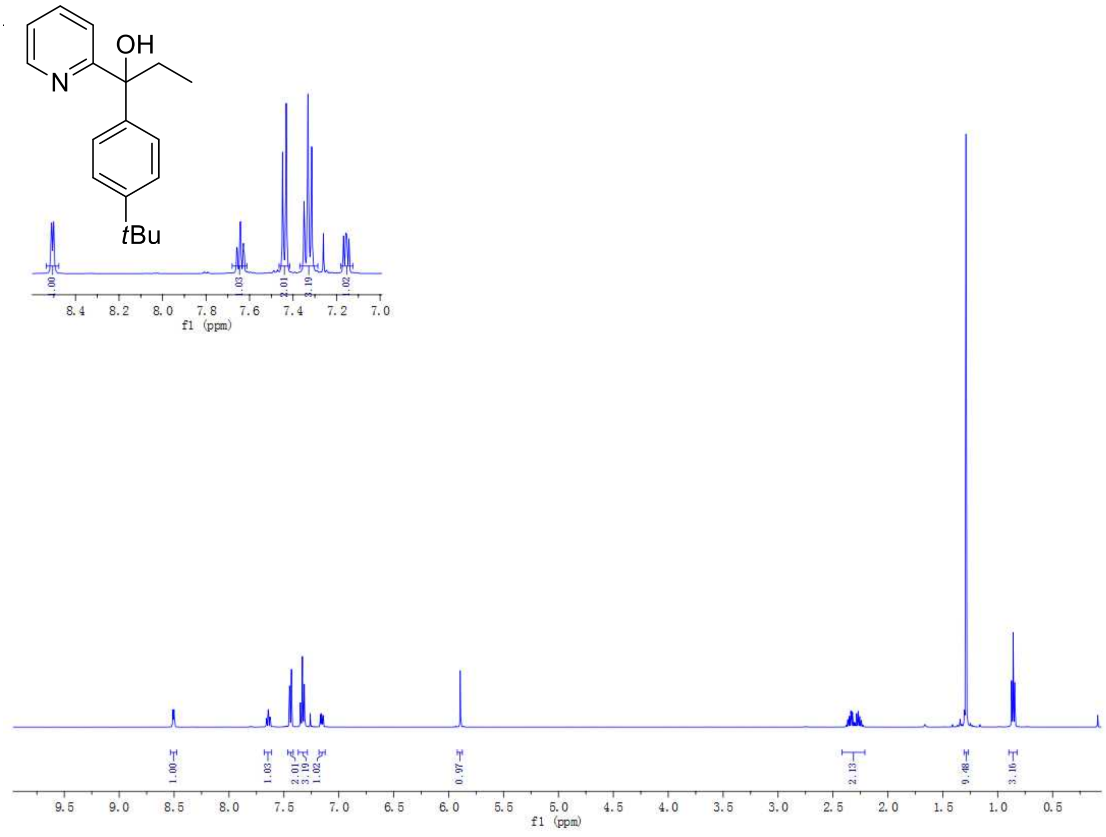

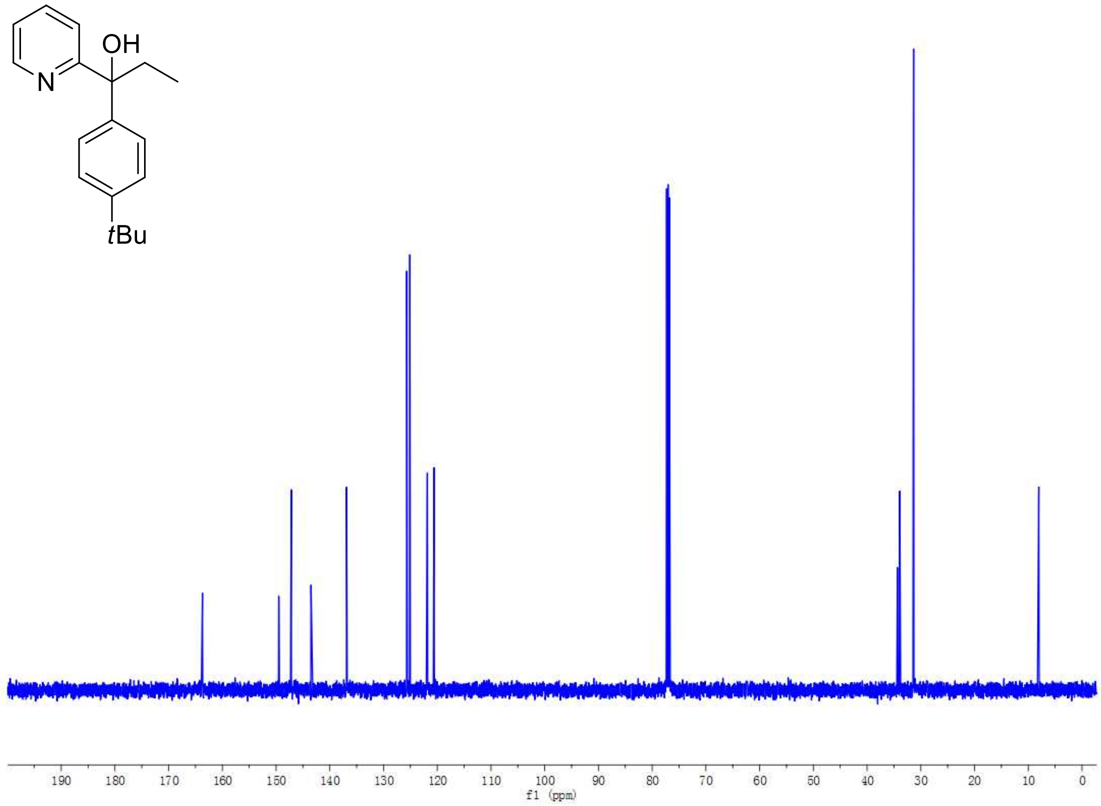

Figure S26. 500 MHz ^{1}H and 125 MHz $^{13}C\{^{1}H\}$ NMR spectra of **5ab** in $CDCl_3$

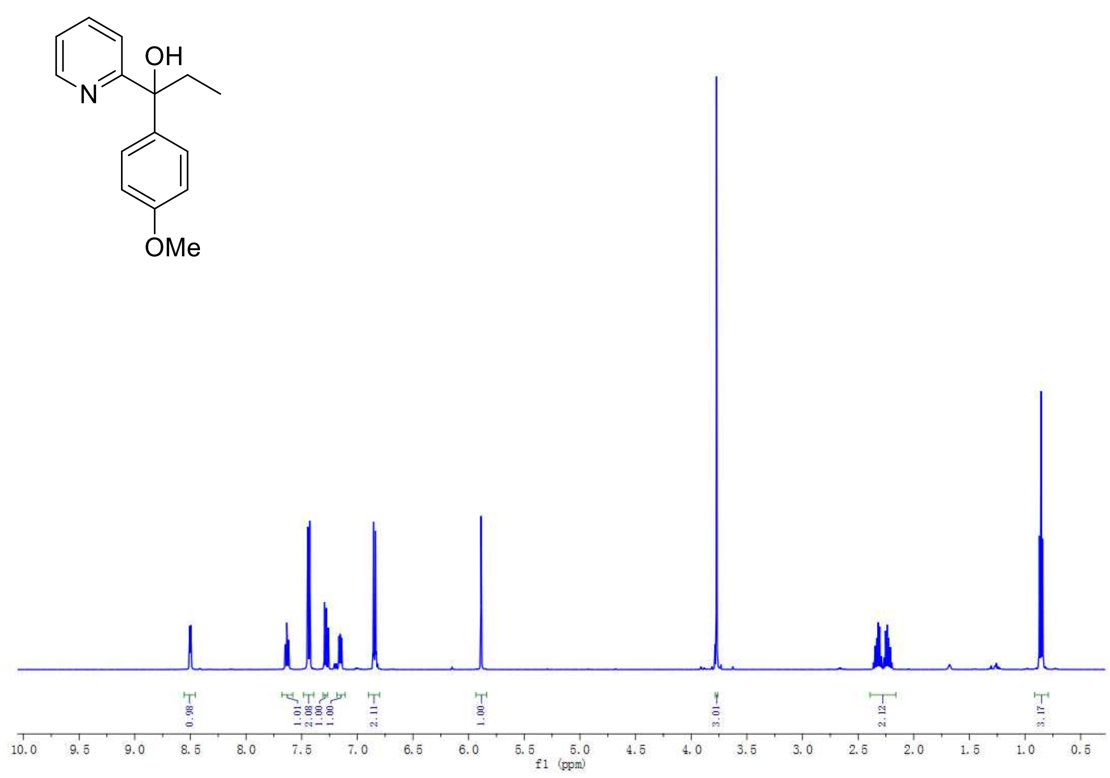

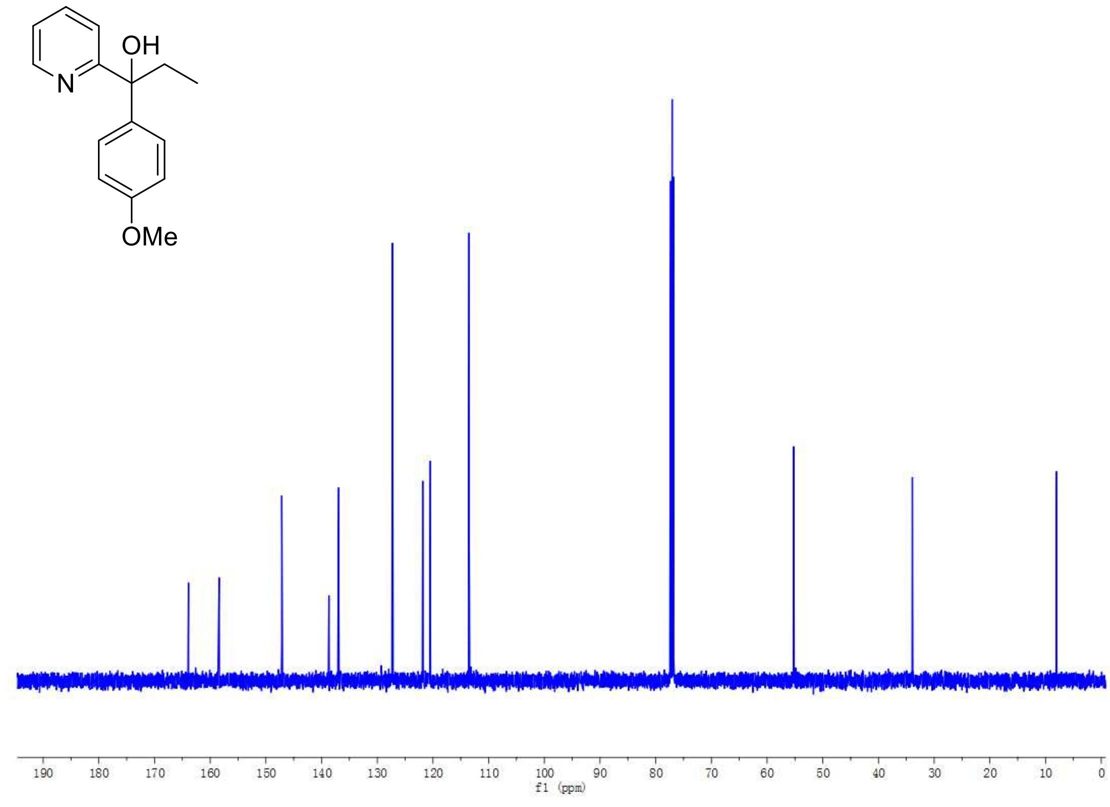

Figure S27. 500 MHz ^{1}H and 125 MHz ^{13}C{^{1}H} NMR spectra of **5ac** in $CDCl_3$

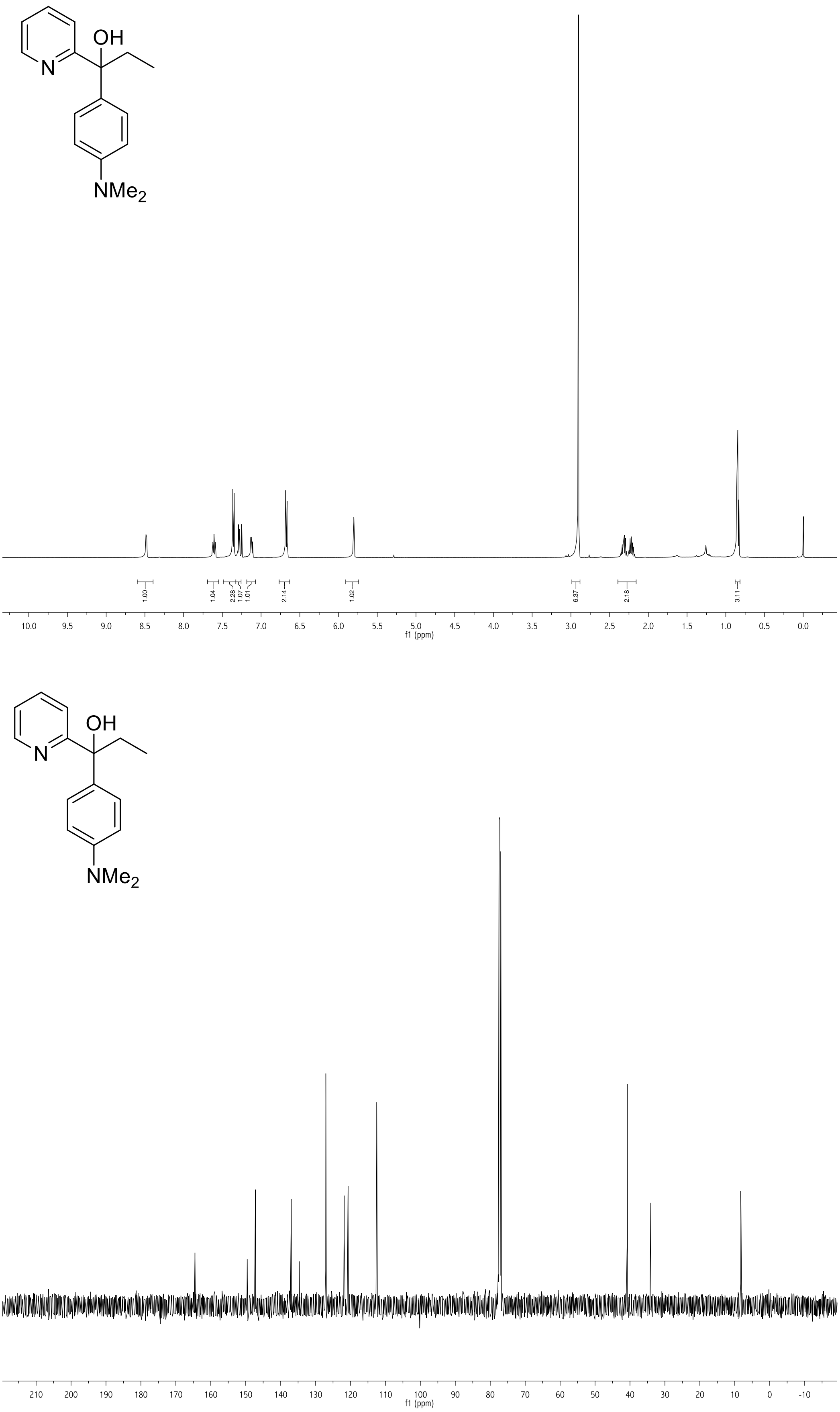

Figure S28. 500 MHz ^{1}H and 125 MHz $^{13}C\{^{1}H\}$ NMR spectra of **5ad** in $CDCl_3$

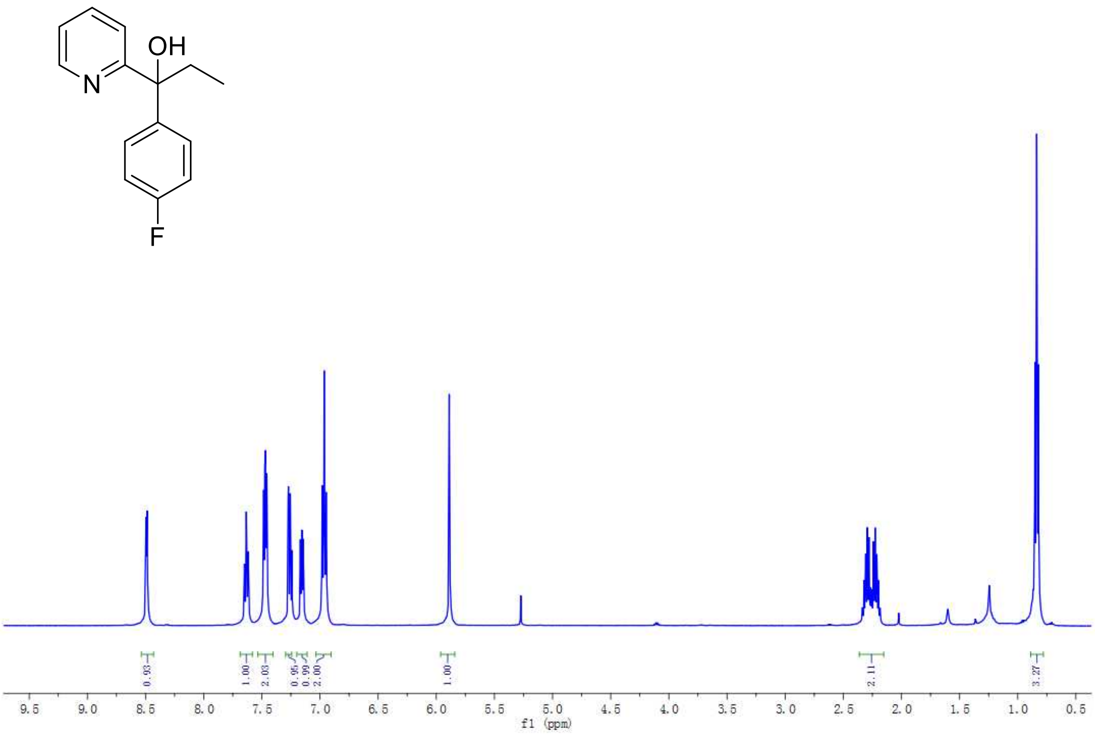

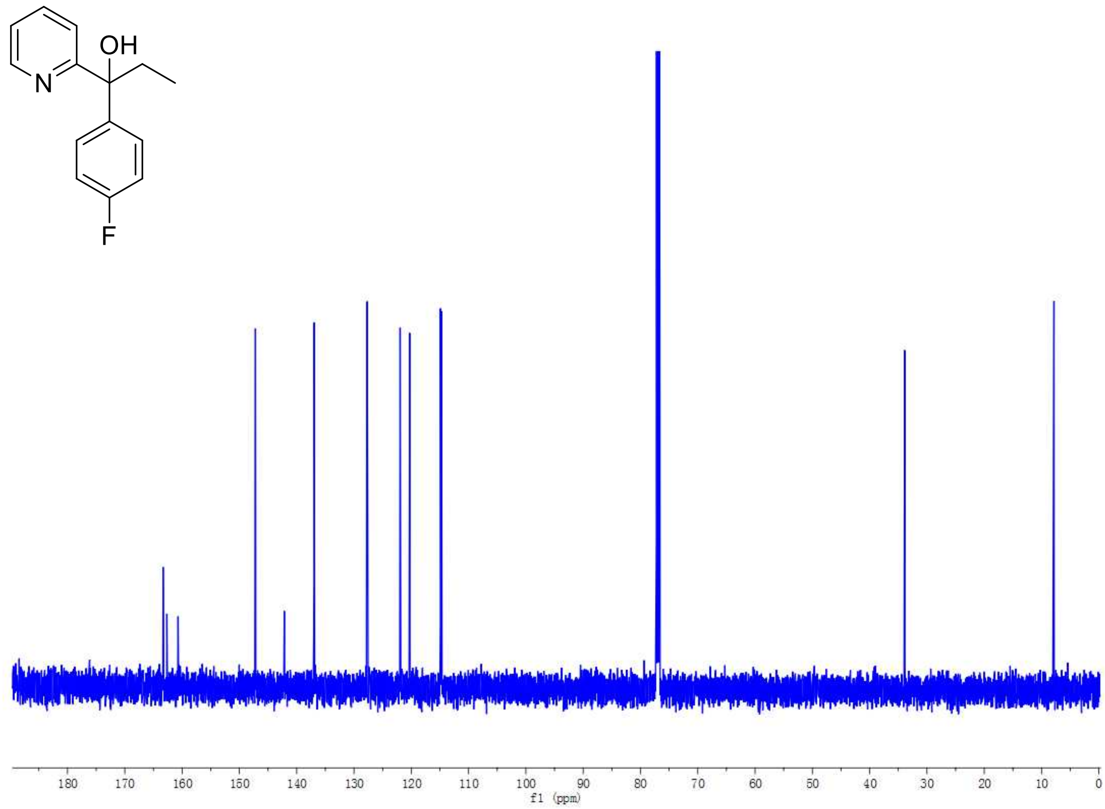

Figure S29. 500 MHz ^{1}H and 125 MHz $^{13}C\{^{1}H\}$ NMR spectra of **5ae** in $CDCl_3$

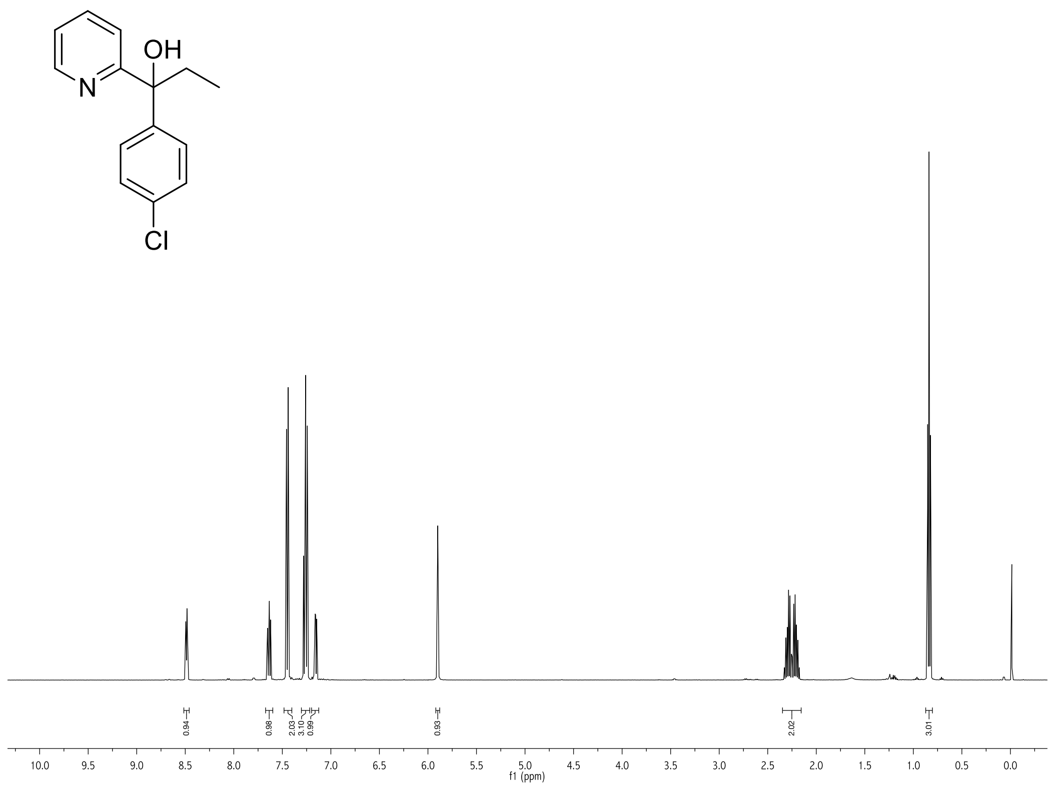

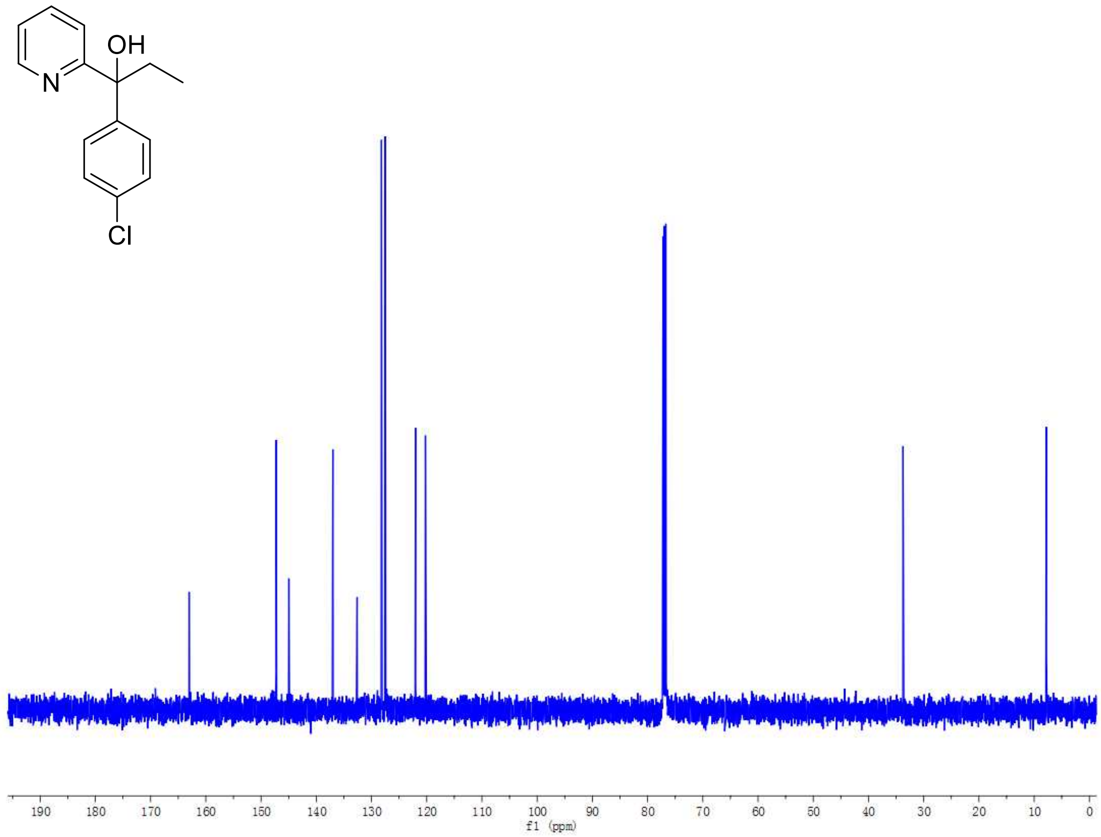

Figure S30. 500 MHz ^{1}H and 125 MHz $^{13}C\{^1H\}$ NMR spectra of 5**ai** in $CDCl_3$

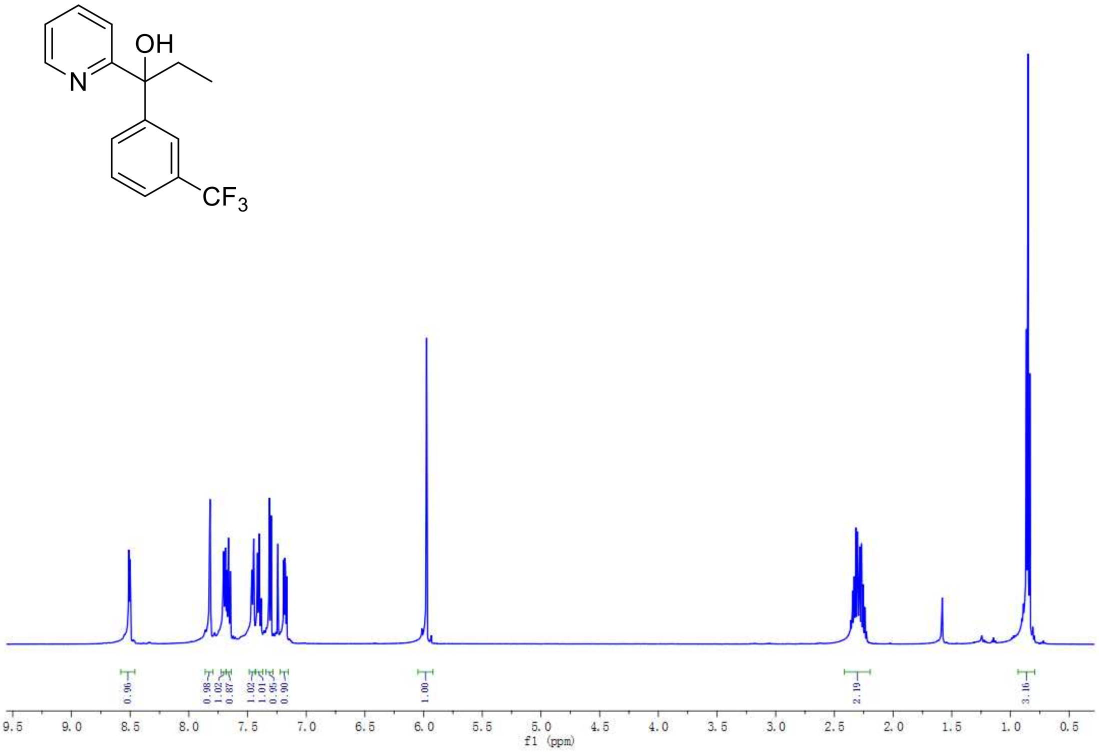

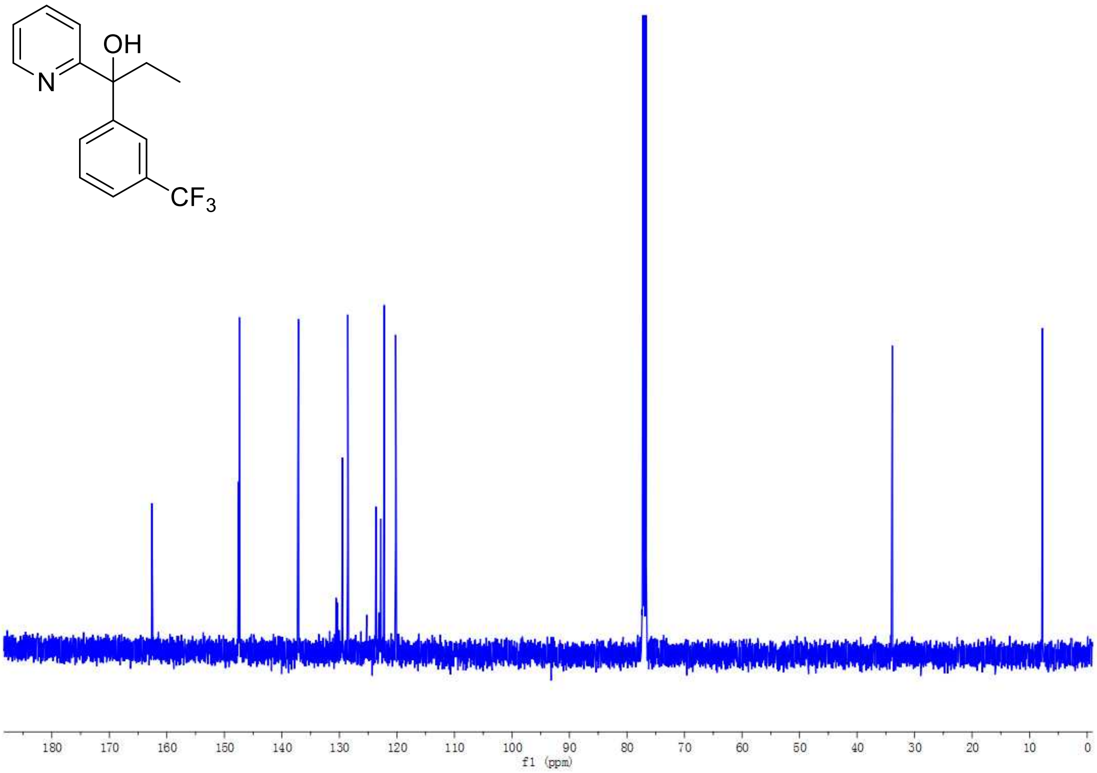

Figure S31. 500 MHz ^{1}H and 125 MHz ^{13}C{^{1}H} NMR spectra of **5aj** in $CDCl_3$

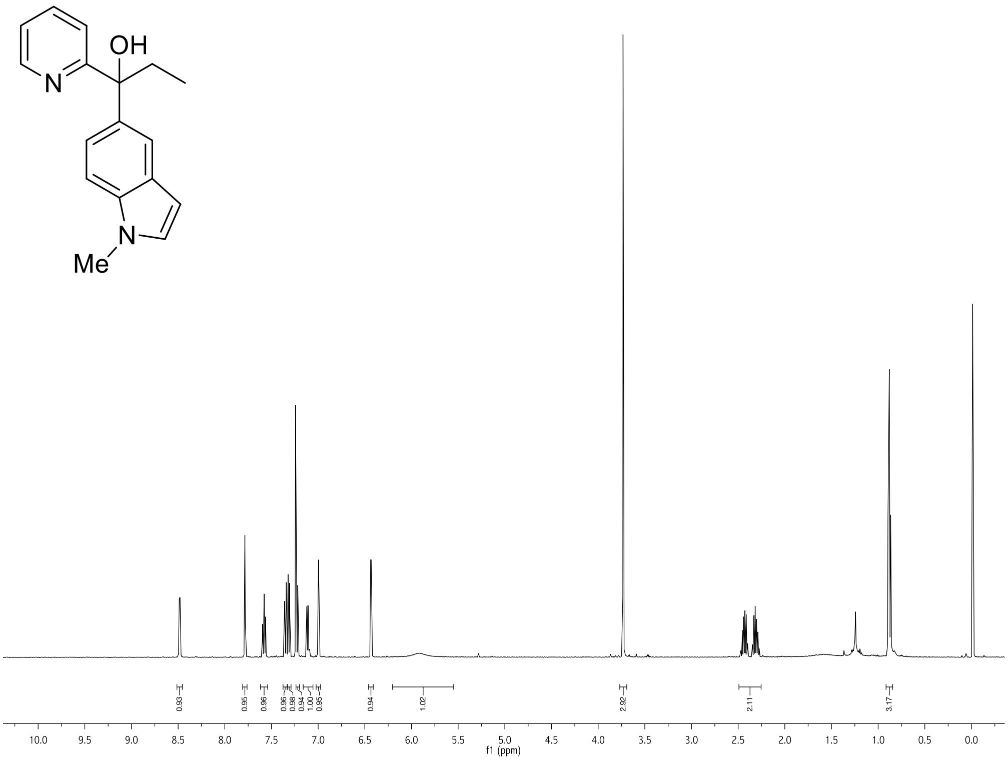

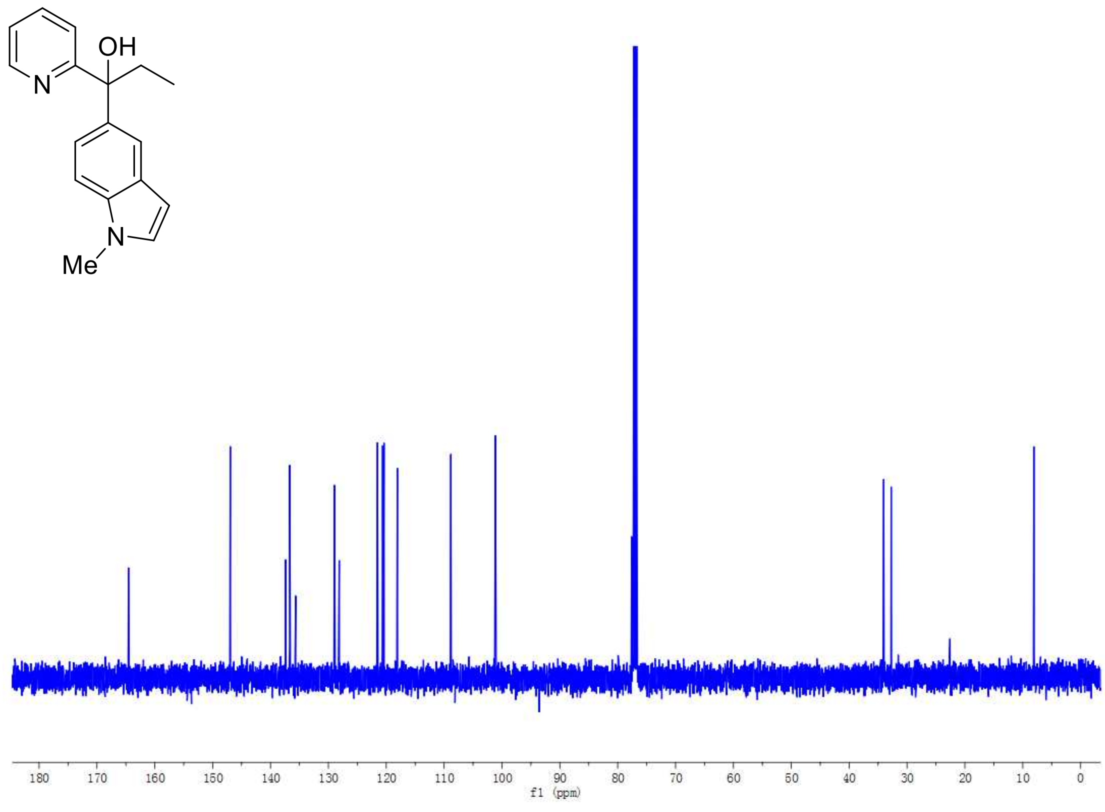

Figure S32. 500 MHz ^{1}H and 125 MHz $^{13}C\{^{1}H\}$ NMR spectra of **5ag** in $CDCl_3$

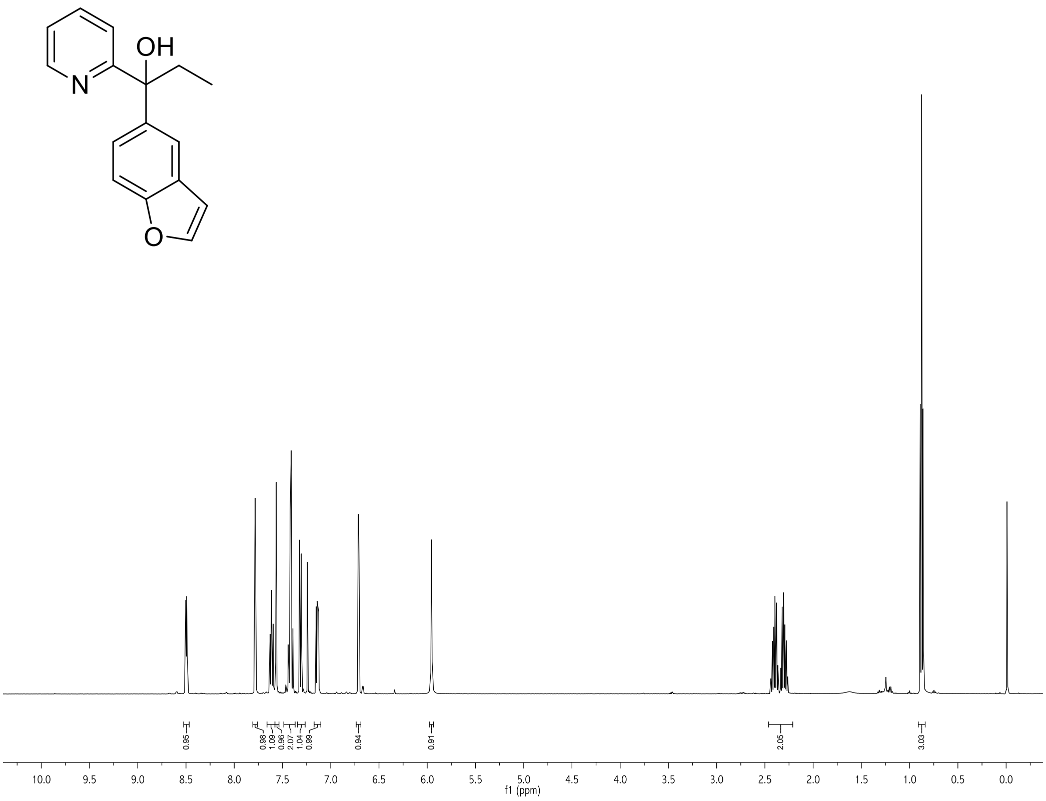

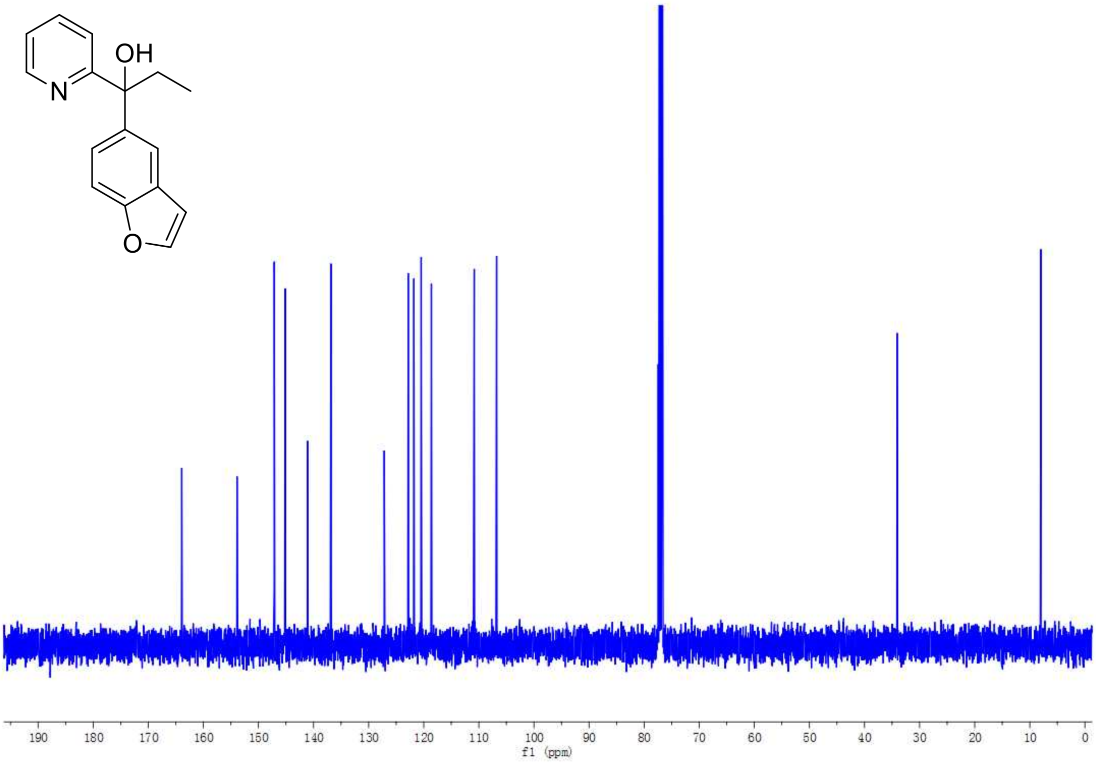

Figure S33. 500 MHz ^{1}H and 125 MHz $^{13}C\{^{1}H\}$ NMR spectra of **5ah** in $CDCl_3$

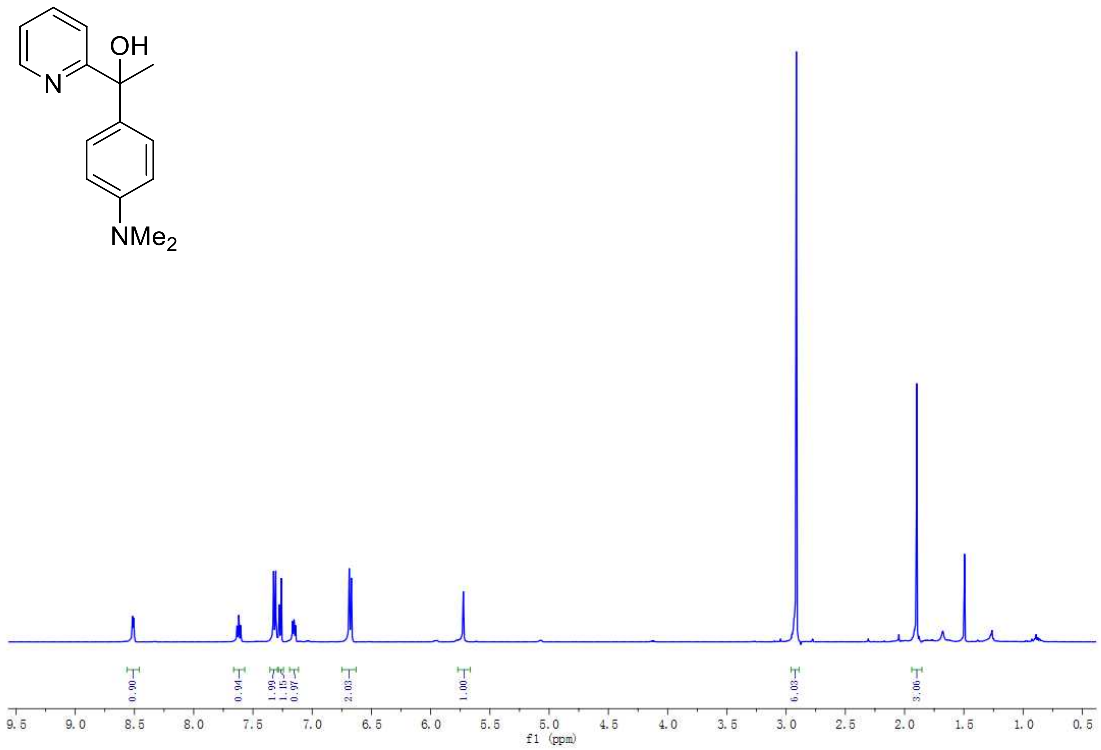

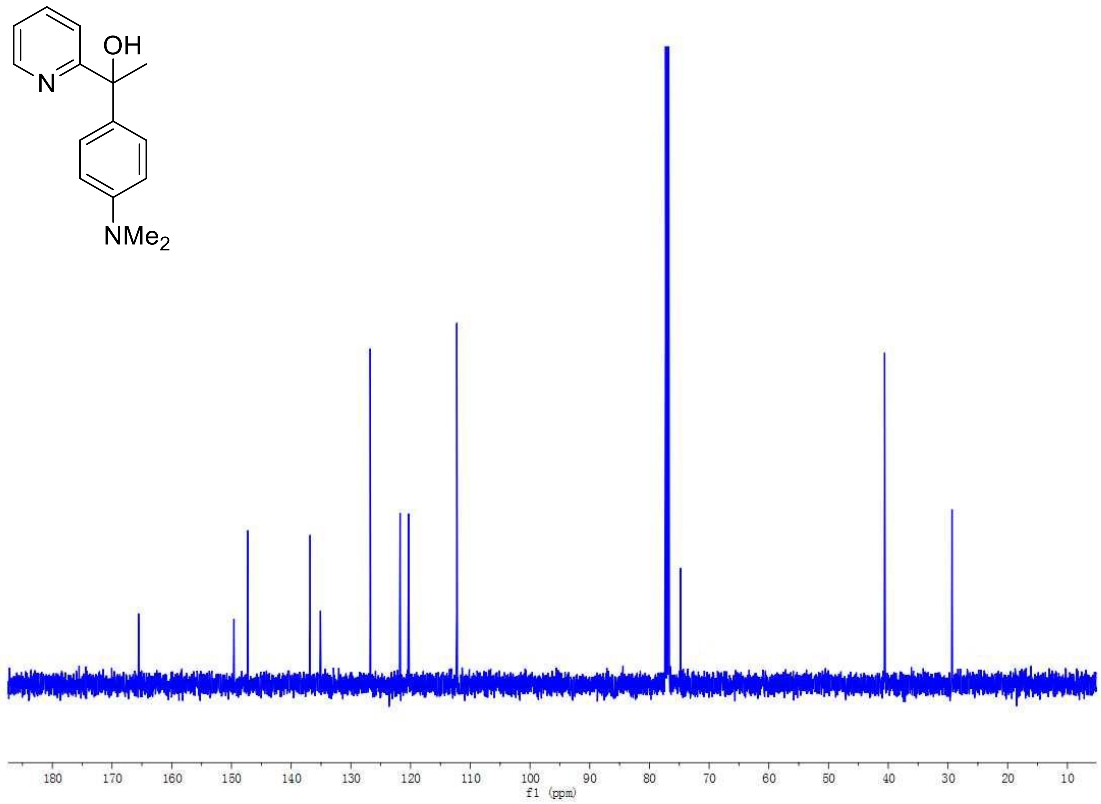

Figure S34. 500 MHz ^{1}H and 125 MHz ^{13}C{^{1}H} NMR spectra of **5bd** in $CDCl_3$

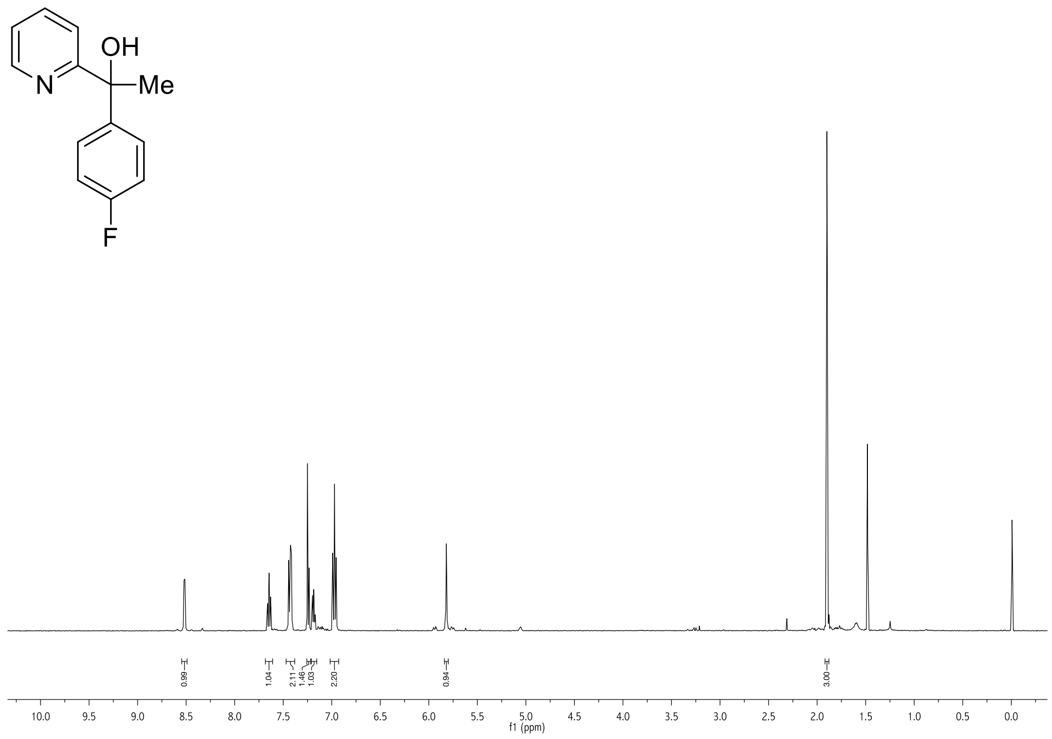

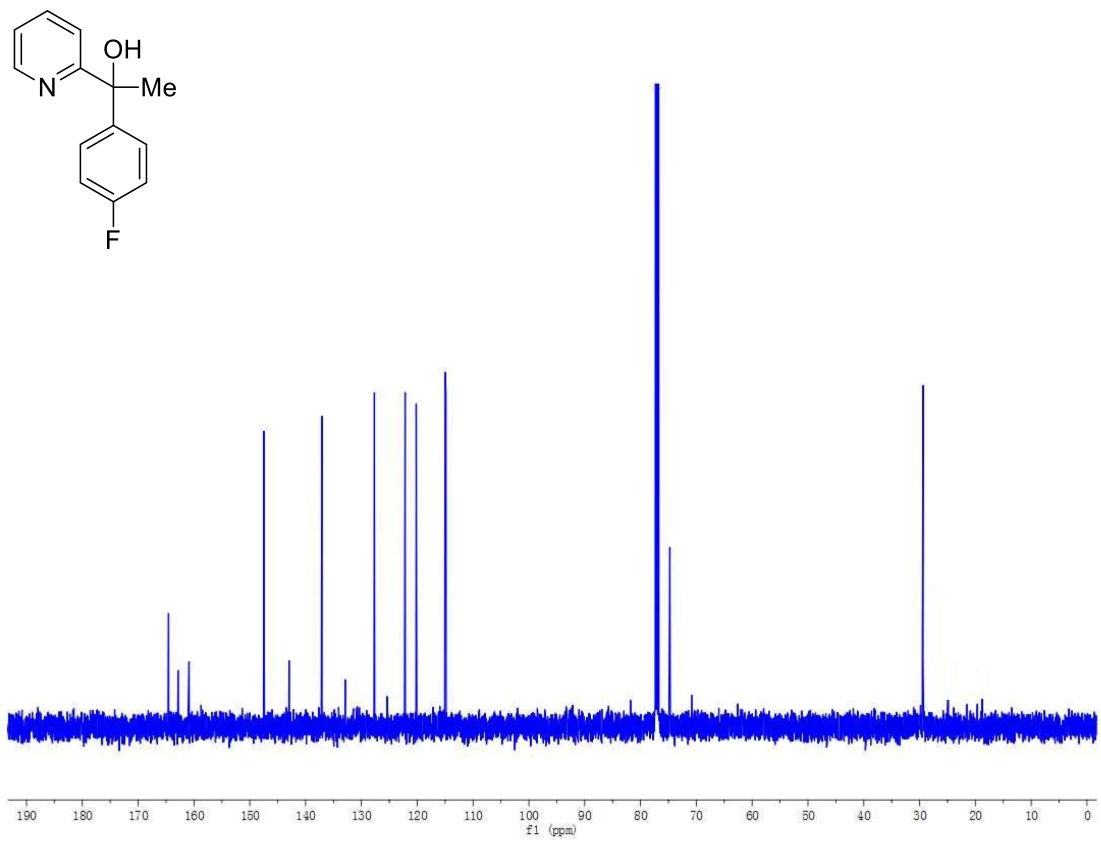

Figure S35. 500 MHz ^{1}H and 125 MHz ^{13}C{^{1}H} NMR spectra of **5be** in $CDCl_{33}$

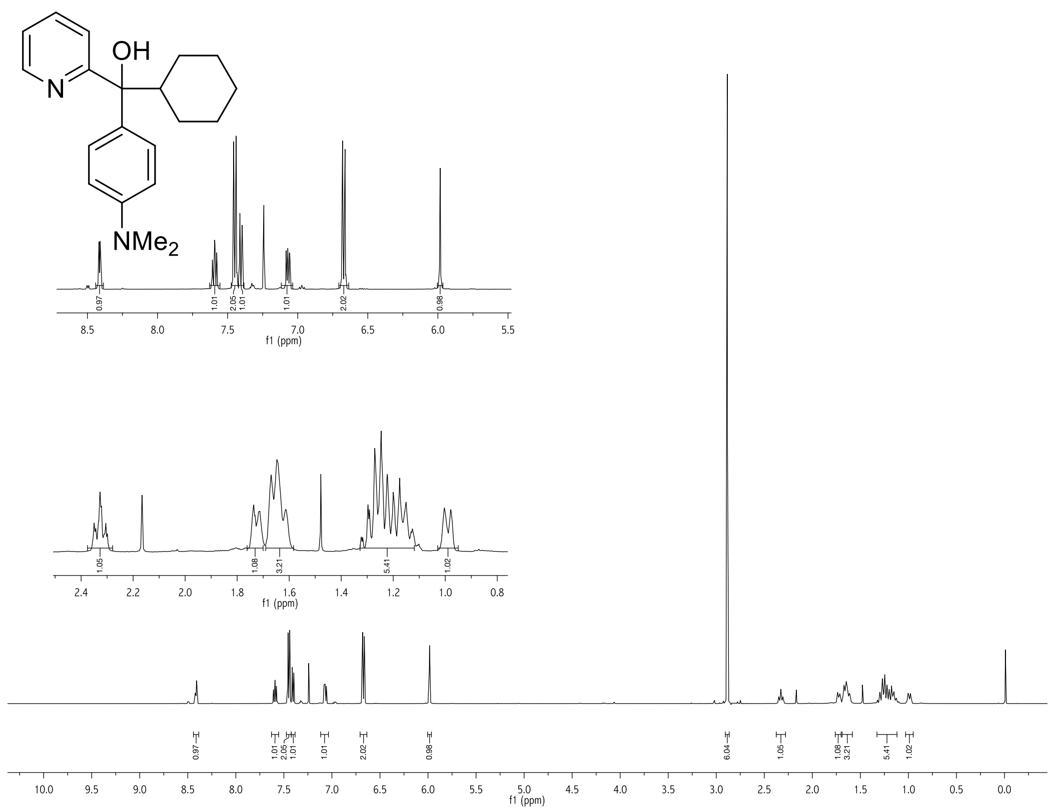

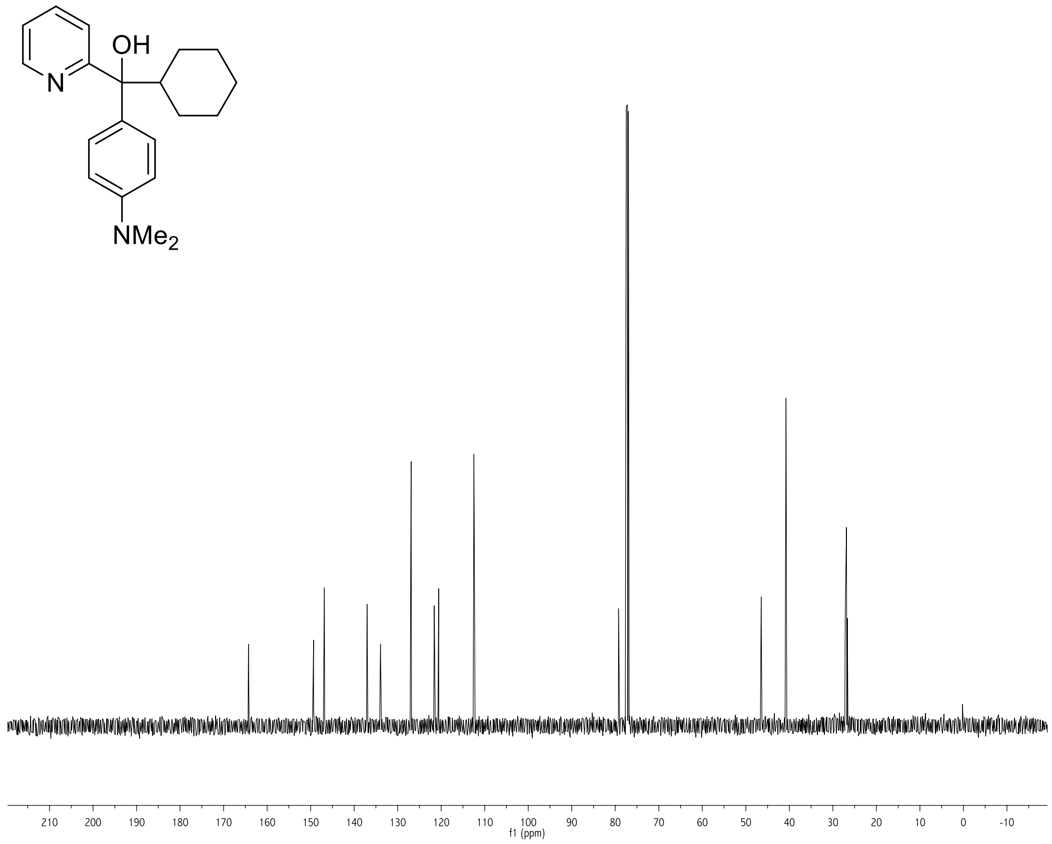

Figure S36. 500 MHz ^{1}H and 125 MHz $^{13}C\{^{1}H\}$ NMR spectra of **5cd** in $CDCl_3$

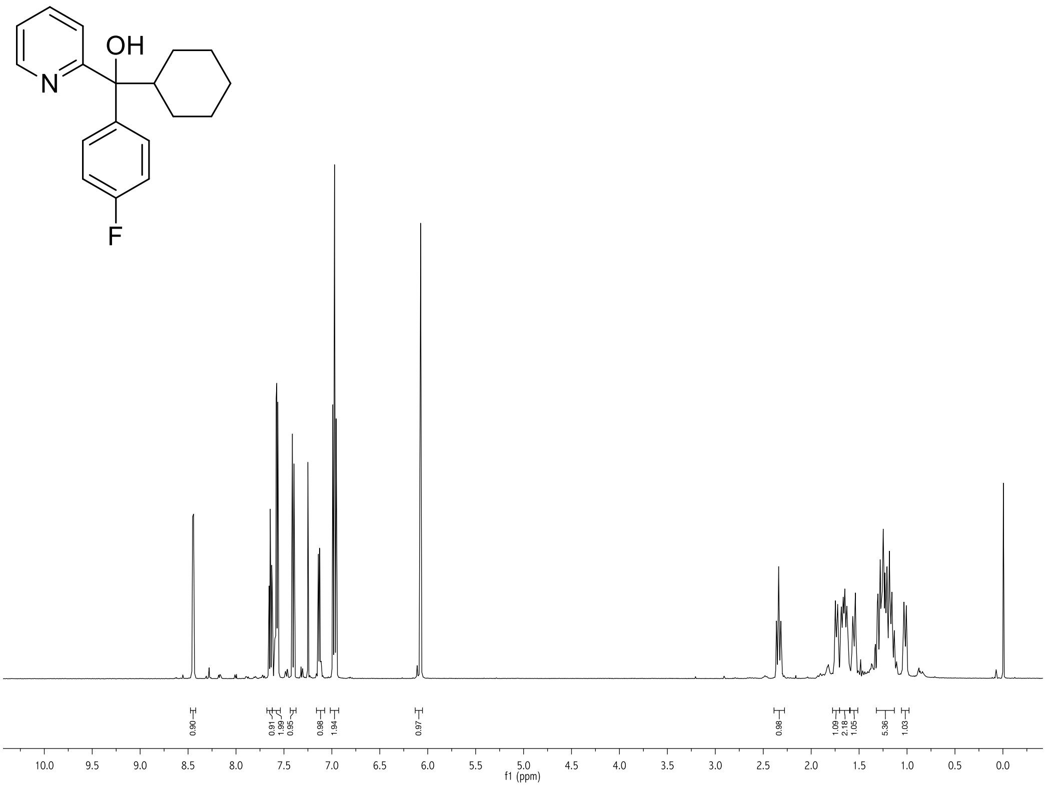

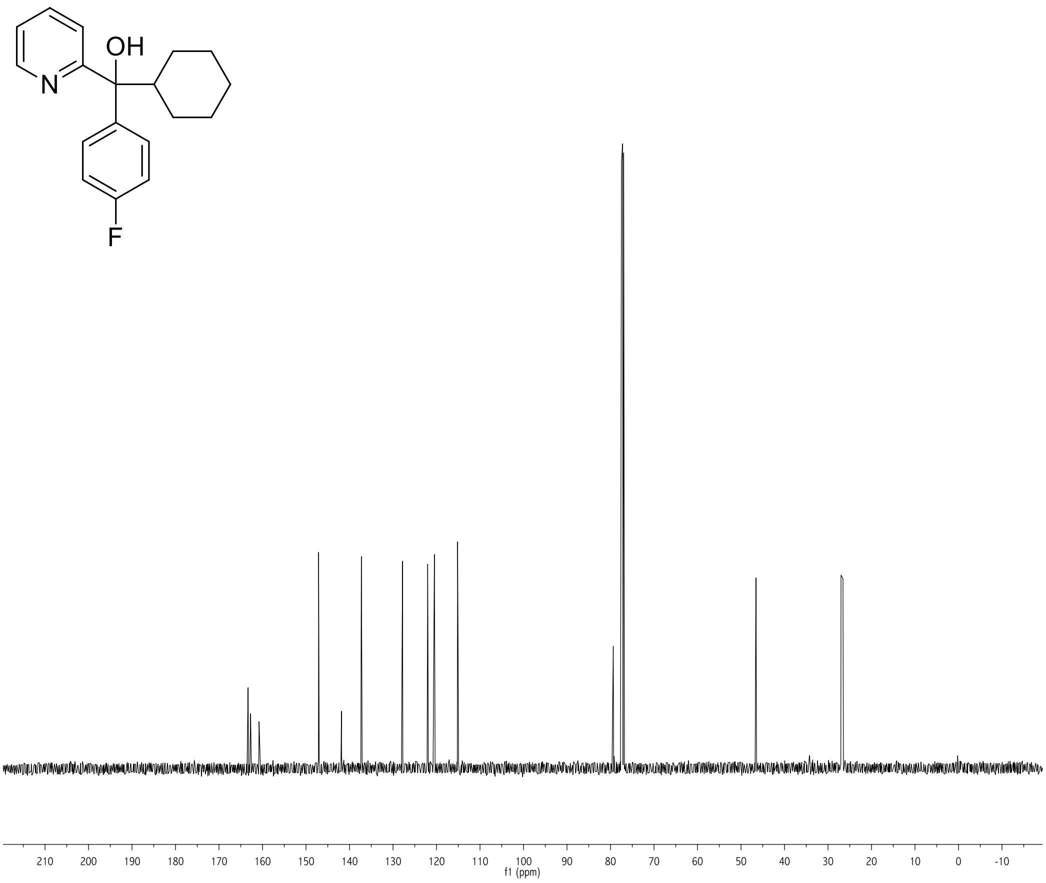

Figure S37. 500 MHz ^{1}H and 125 MHz ^{13}C{^{1}H} NMR spectra of **5ce** in $CDCl_3$

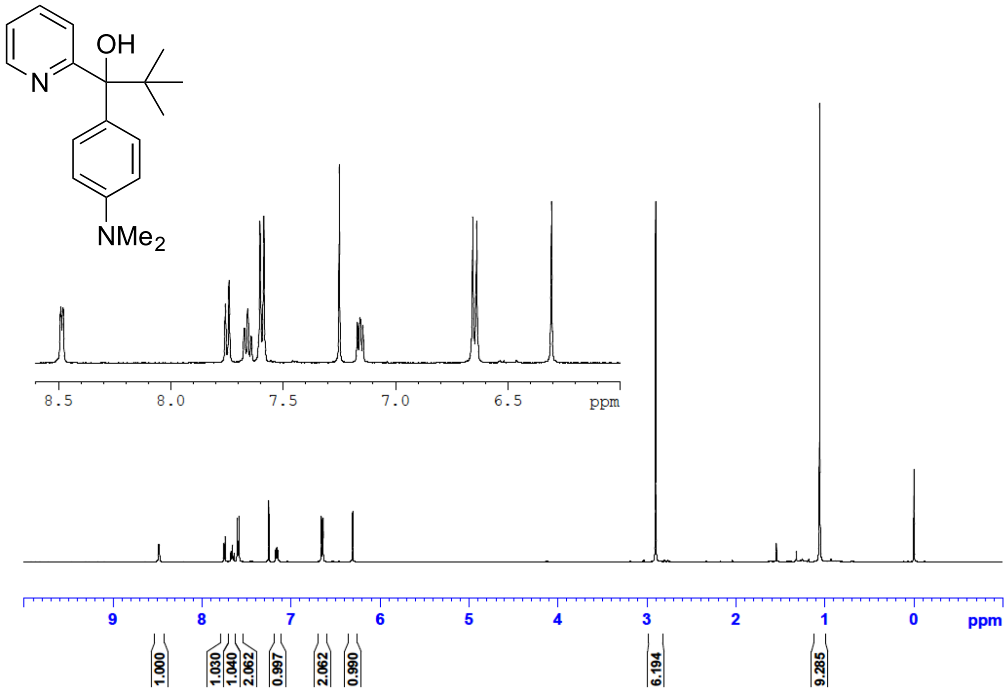

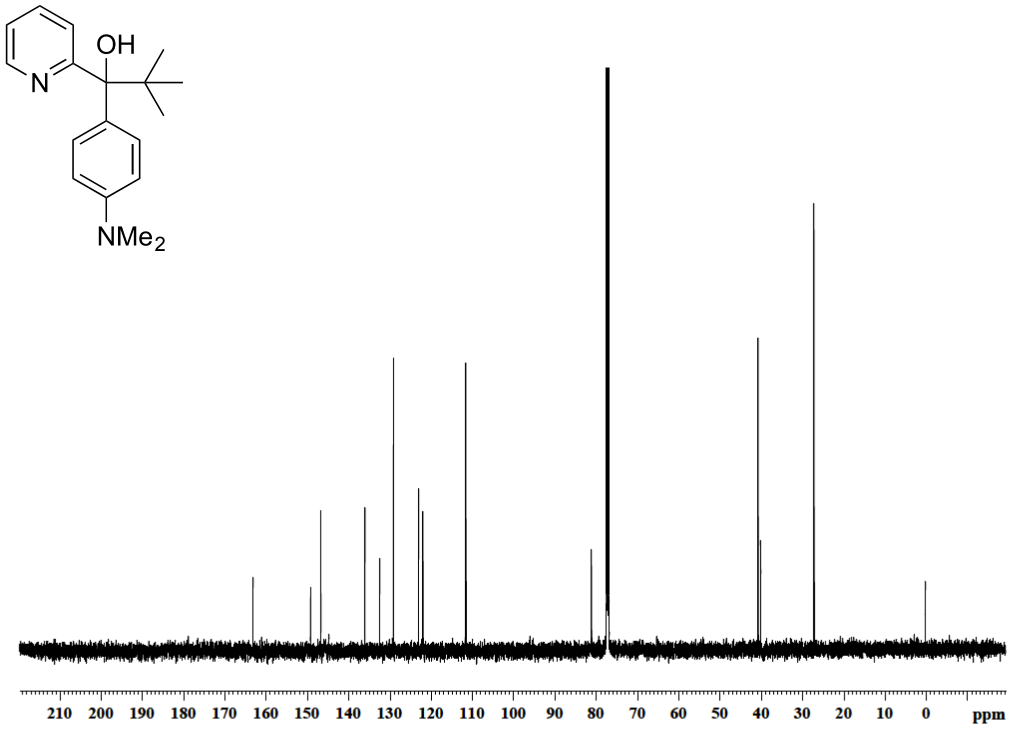

Figure S38. 500 MHz ^{1}H and 125 MHz $^{13}C\{^{1}H\}$ NMR spectra of **5dd** in $CDCl_3$

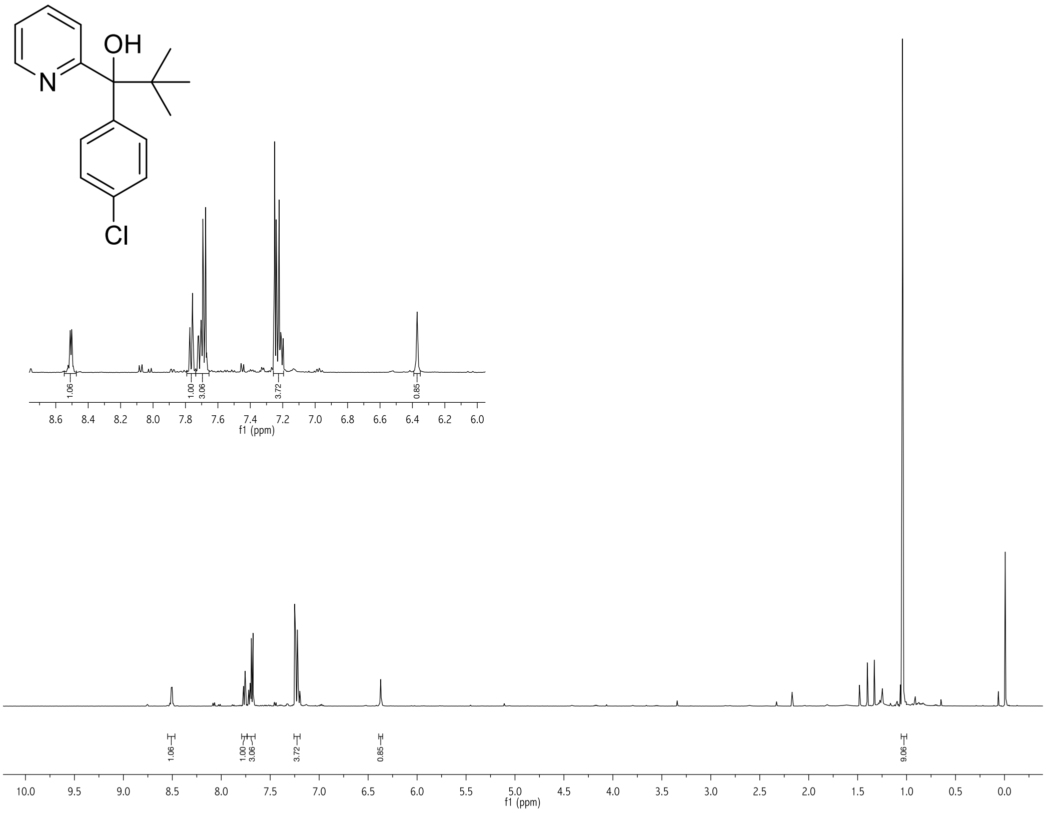

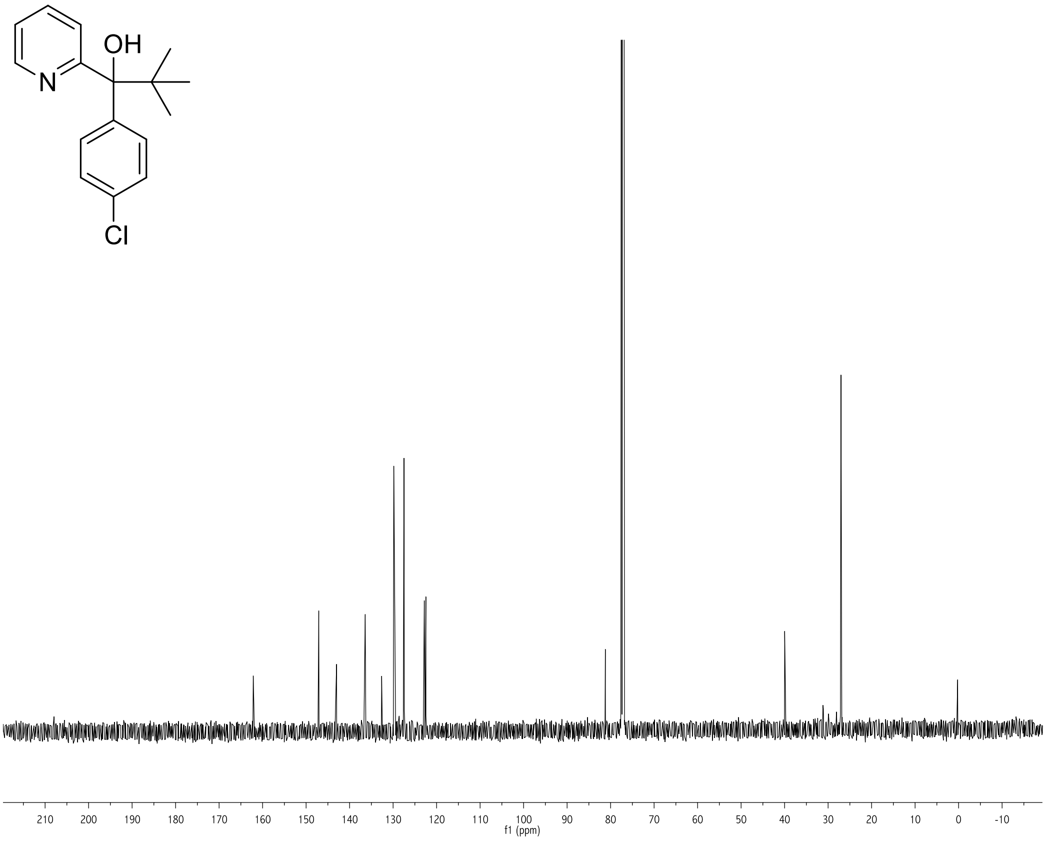

Figure S39. 500 MHz ^{1}H and 125 MHz $^{13}C\{^{1}H\}$ NMR spectra of **5di** in $CDCl_3$

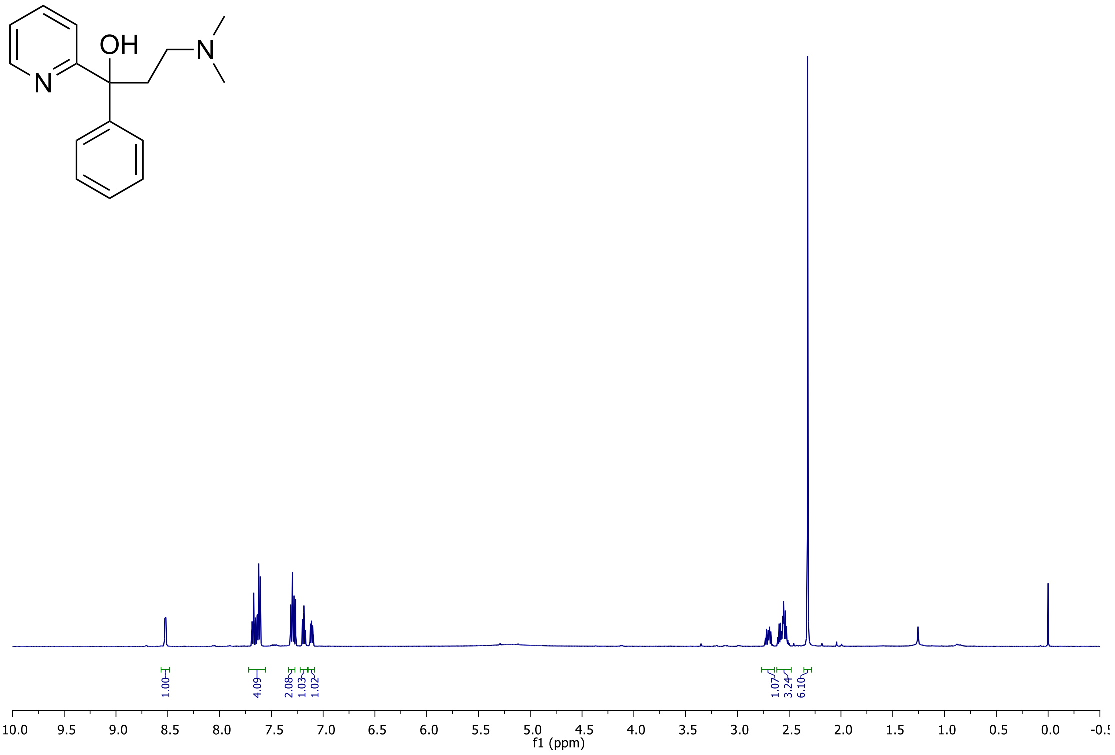

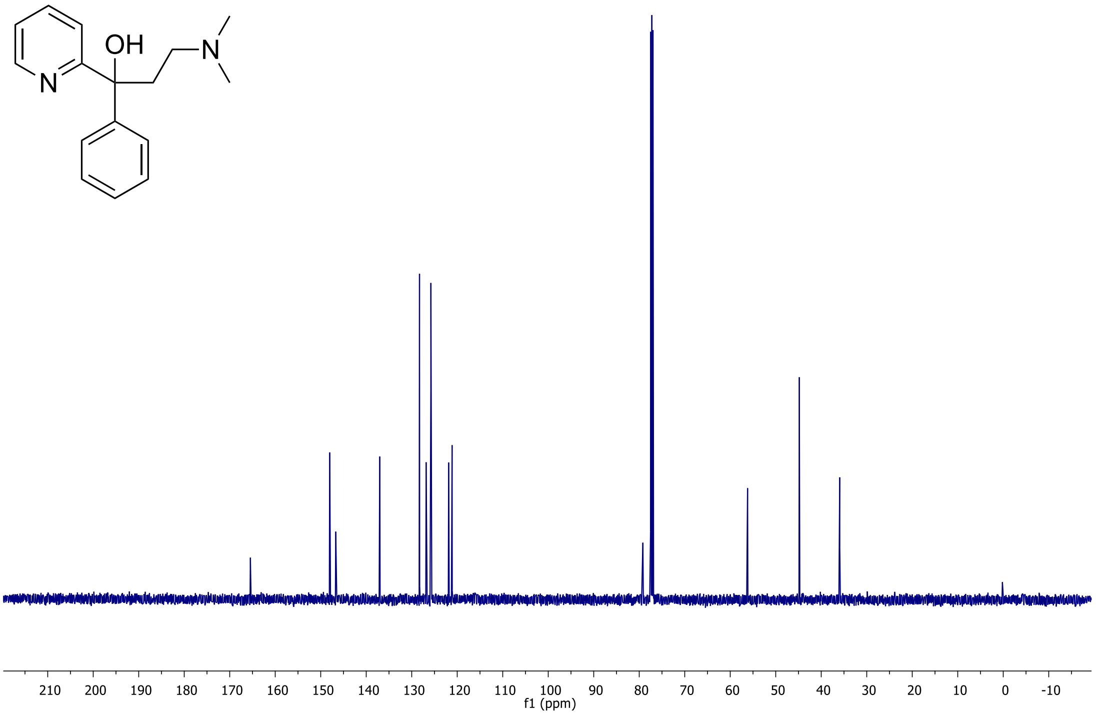

Figure S40. 500 MHz ^{1}H and 125 MHz ^{13}C{^{1}H} NMR spectra of **5ha** in $CDCl_3$

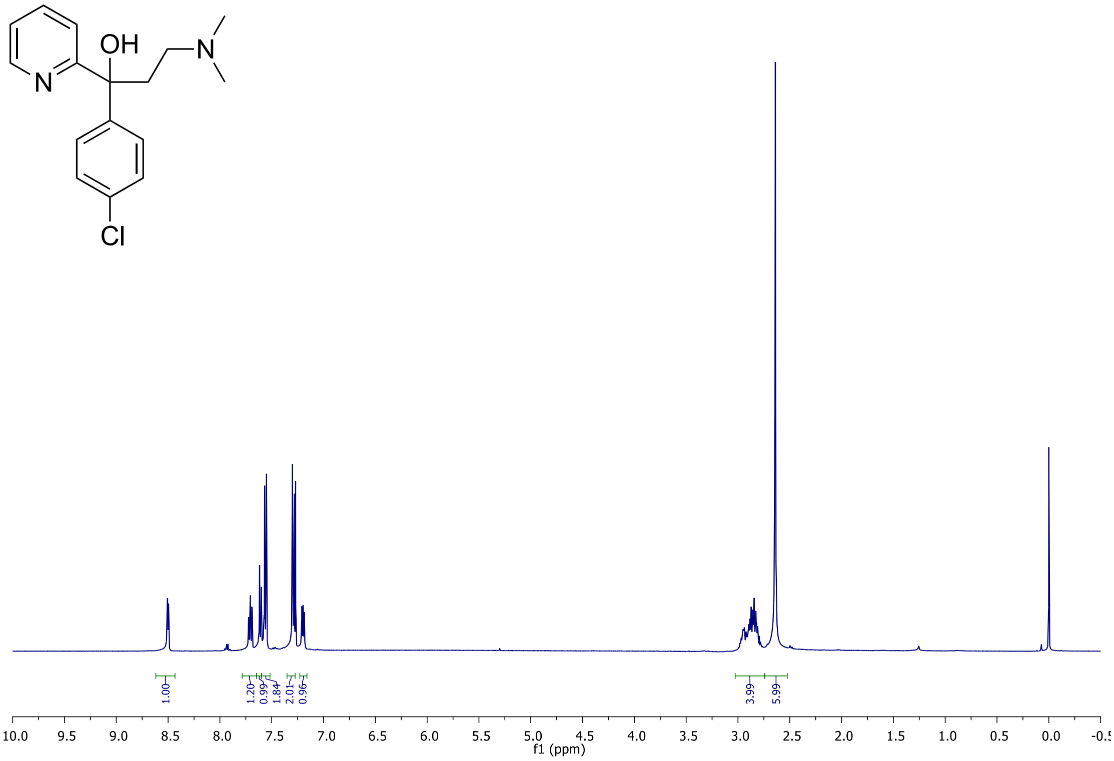

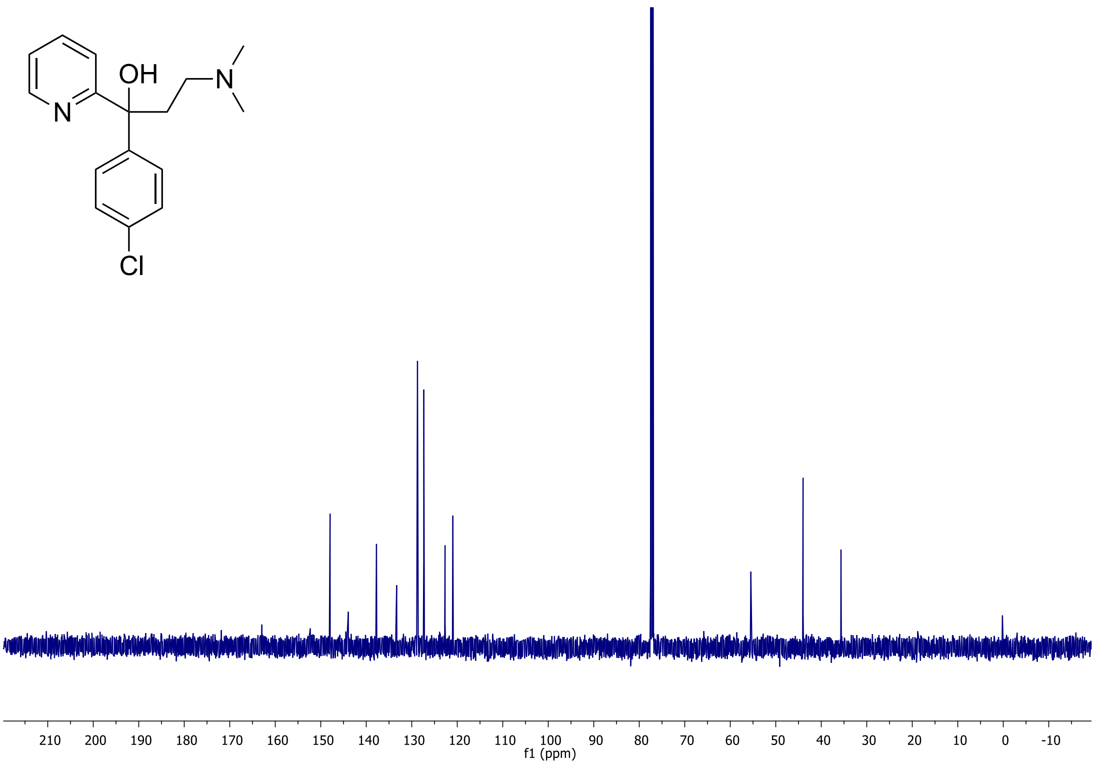

Figure S41. 500 MHz ^{1}H and 125 MHz ^{13}C{^{1}H} NMR spectra of **5hi** in $CDCl_3$

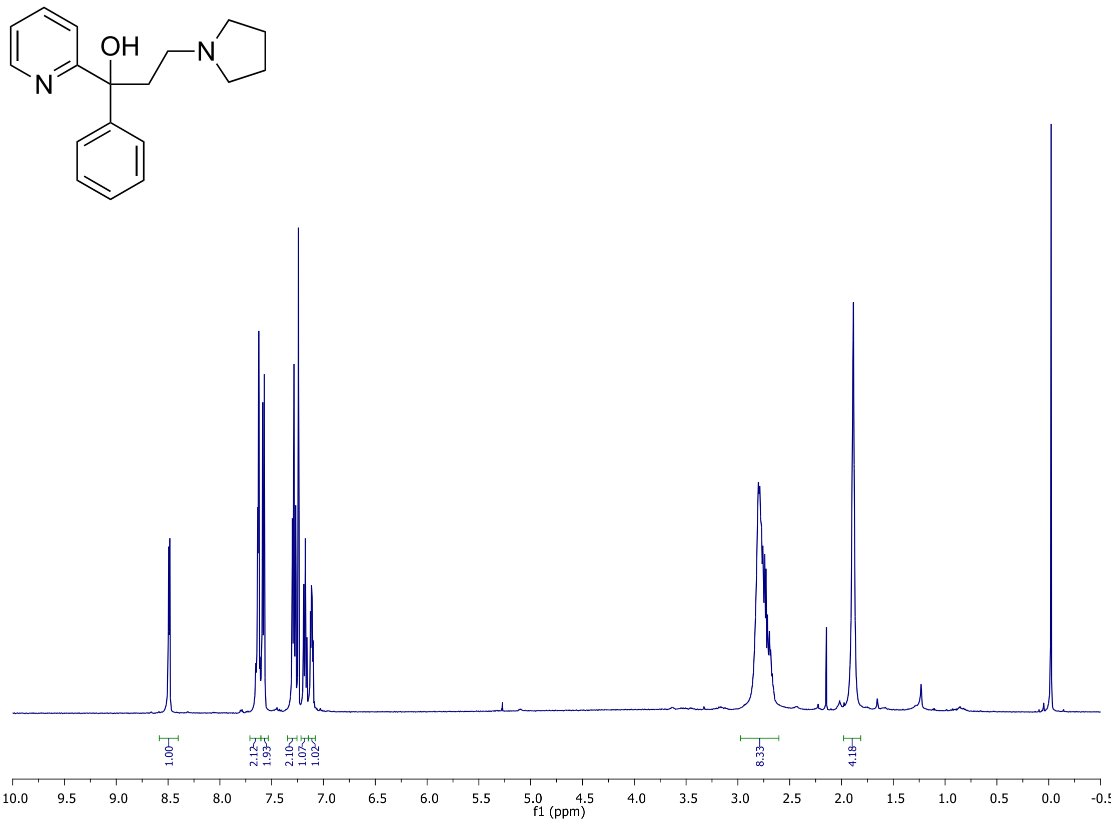

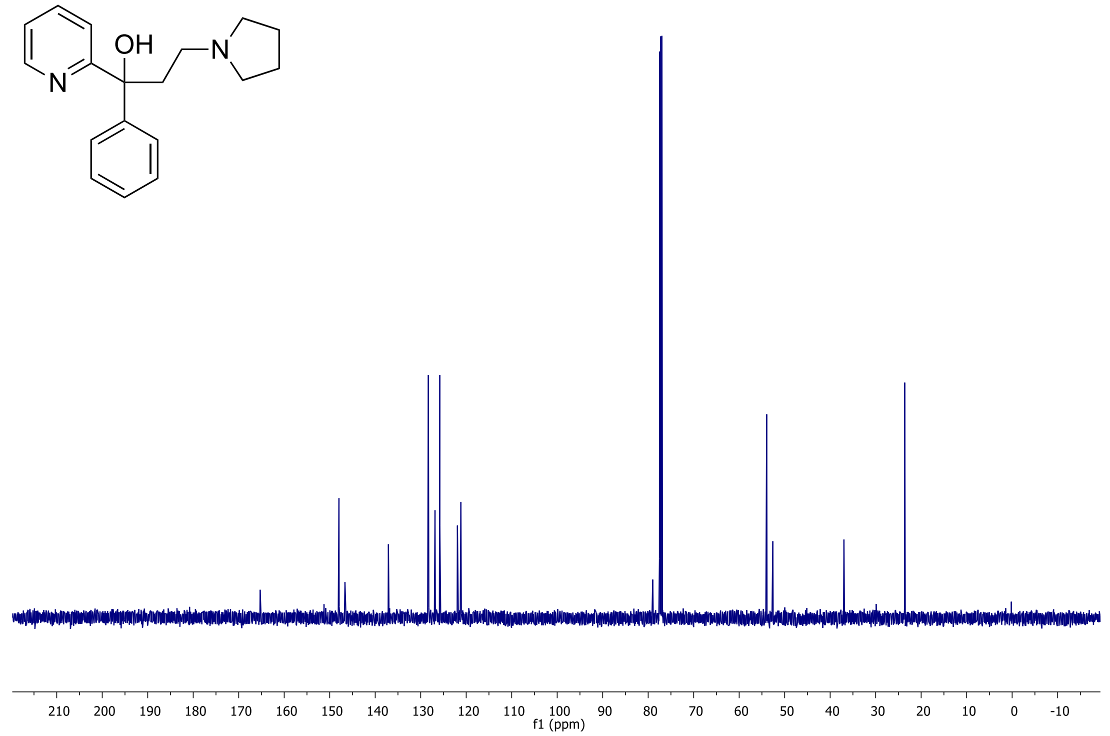

Figure S42. 500 MHz ^{1}H and 125 MHz ^{13}C{^{1}H} NMR spectra of **5ia** in $CDCl_3$

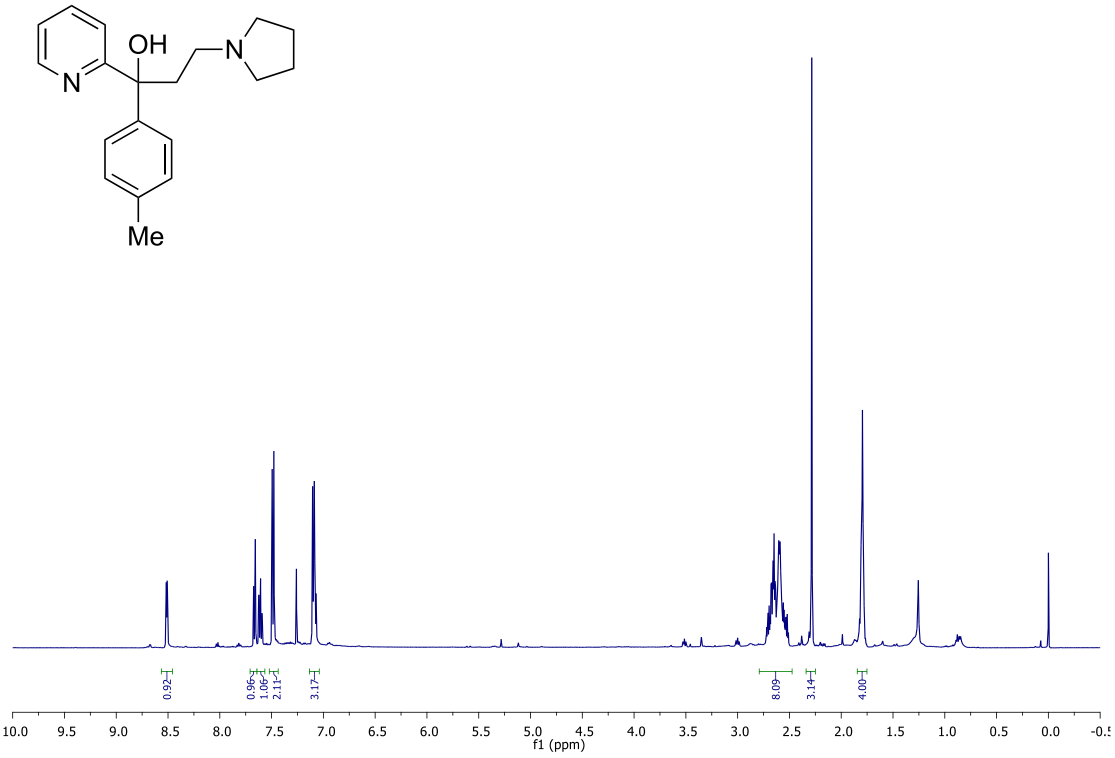

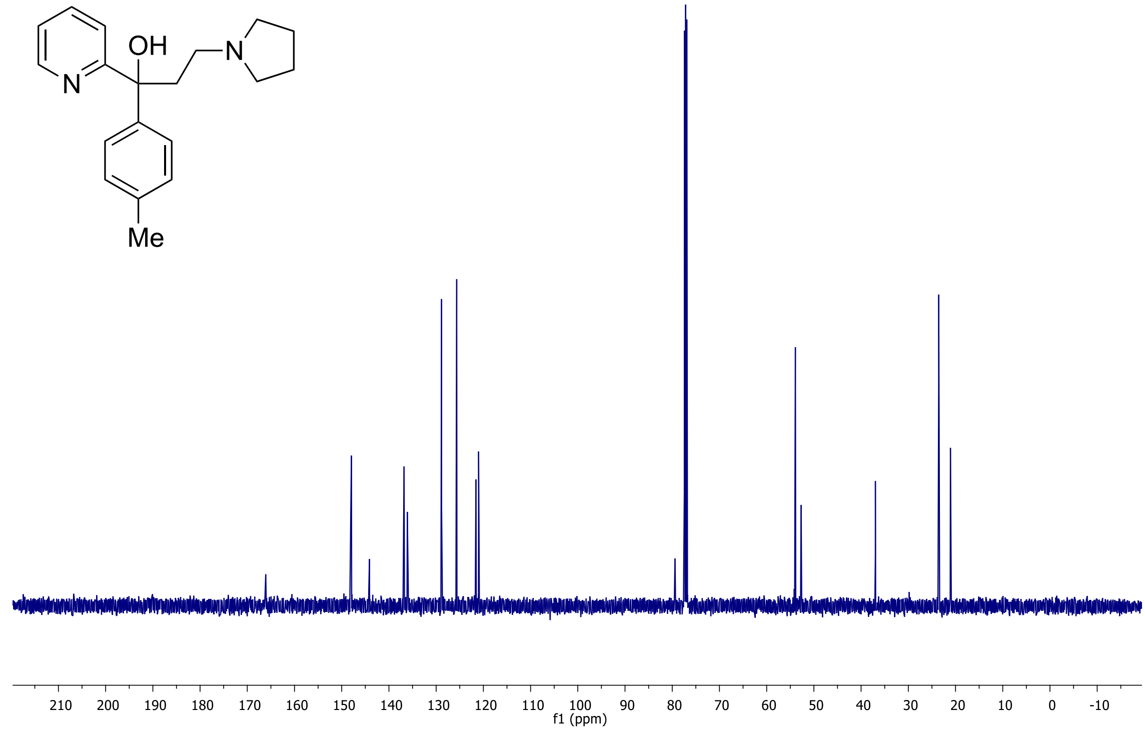

Figure S43. 500 MHz ^{1}H and 125 MHz $^{13}C\{^{1}H\}$ NMR spectra of **5ik** in $CDCl_3$

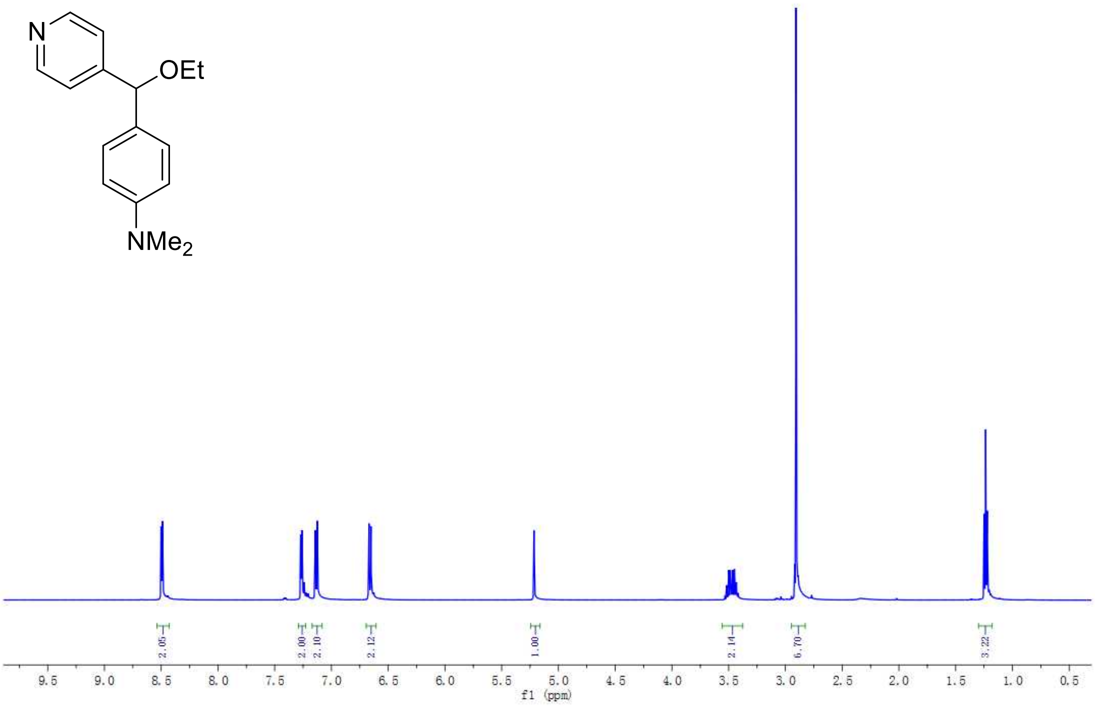

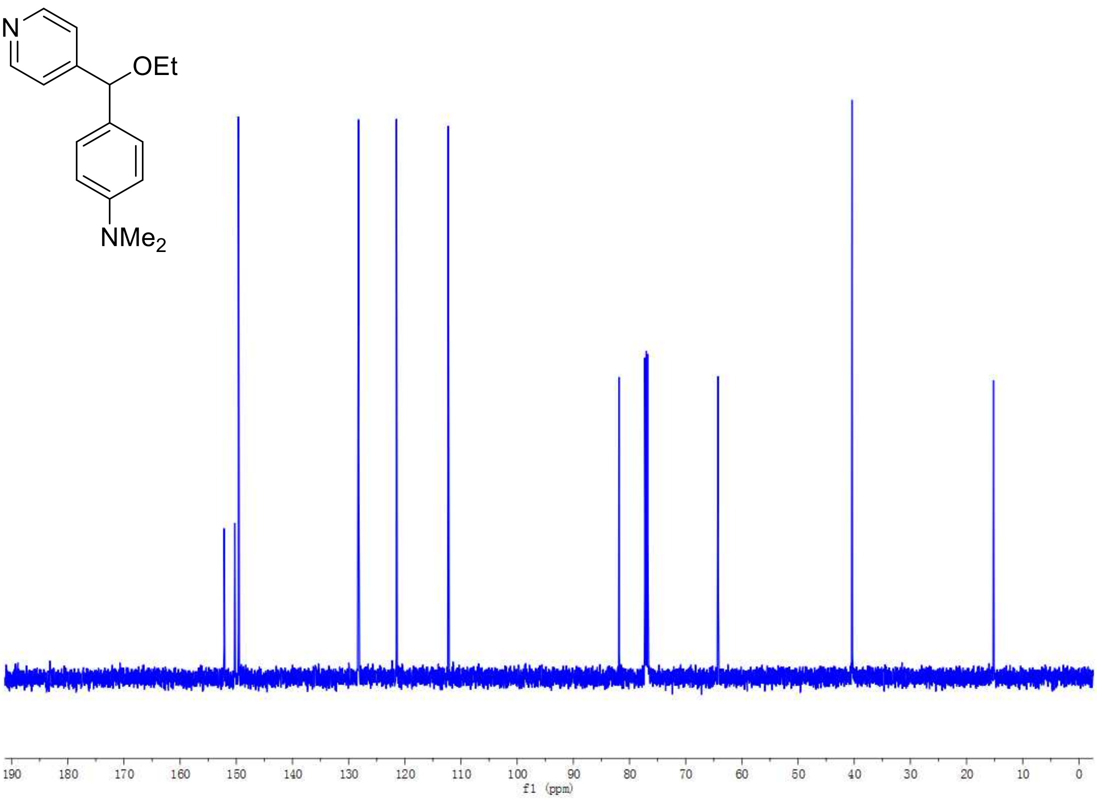

Figure S44. 500 MHz ^{1}H and 125 MHz $^{13}C\{^{1}H\}$ NMR spectra of **4jd** in $CDCl_3$

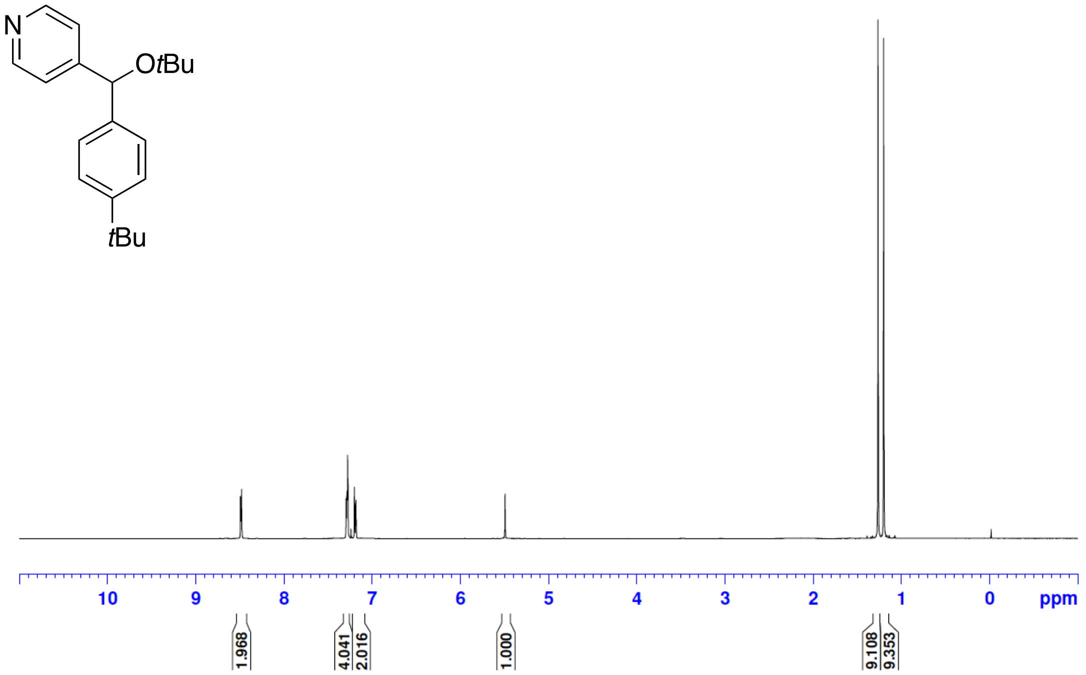

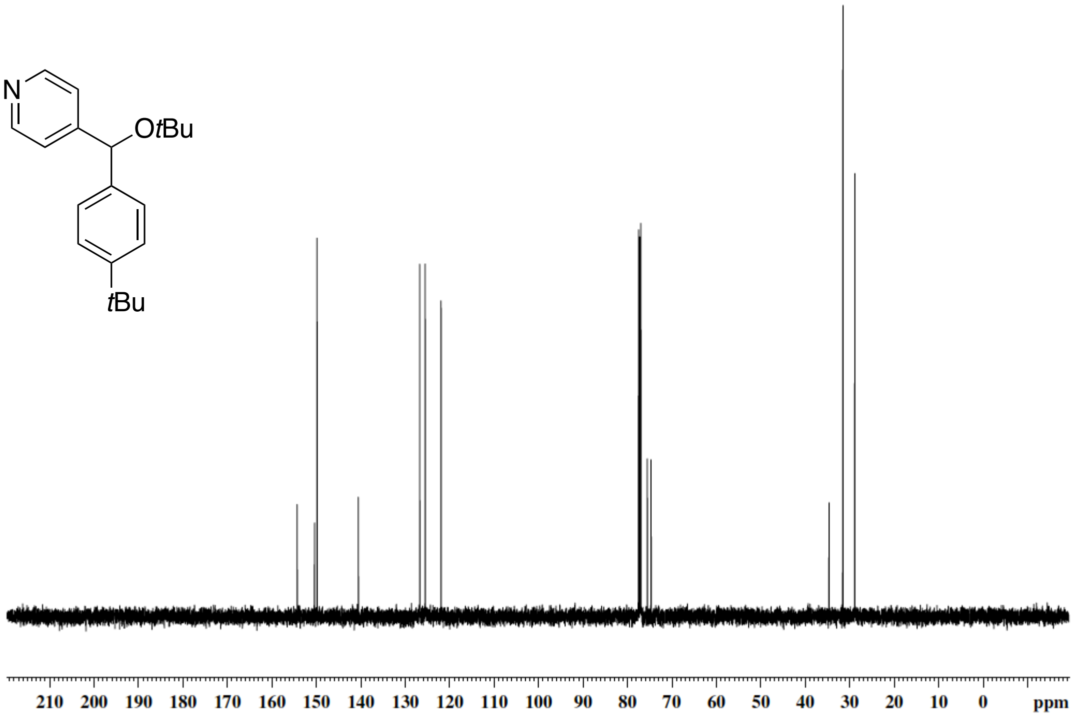

Figure S45. 500 MHz ^{1}H and 125 MHz $^{13}C\{^{1}H\}$ NMR spectra of **4kb** in $CDCl_3$

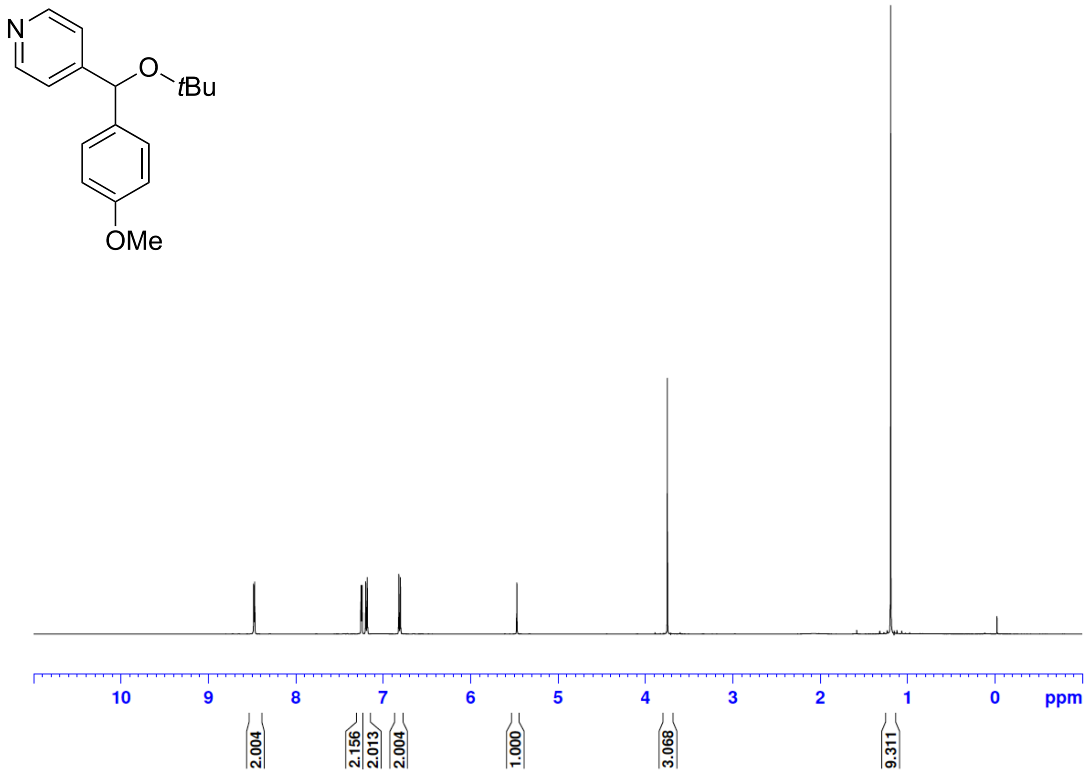

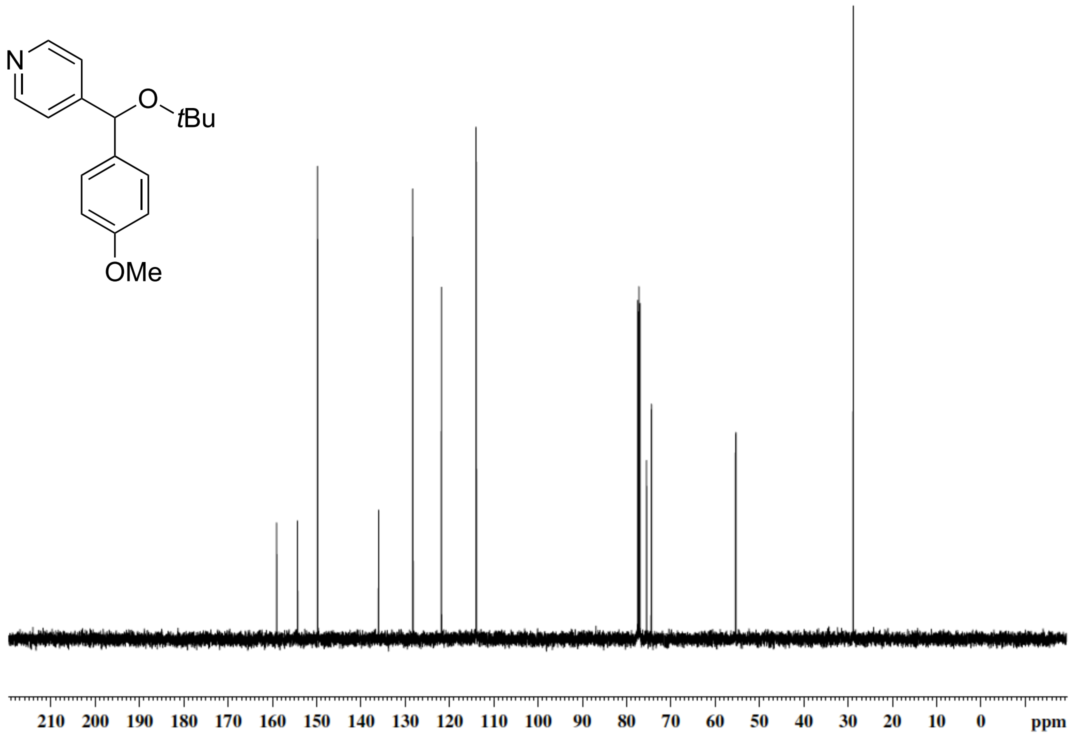

Figure S46. 500 MHz ^{1}H and 125 MHz $^{13}C\{^{1}H\}$ NMR spectra of **4kc** in $CDCl_3$

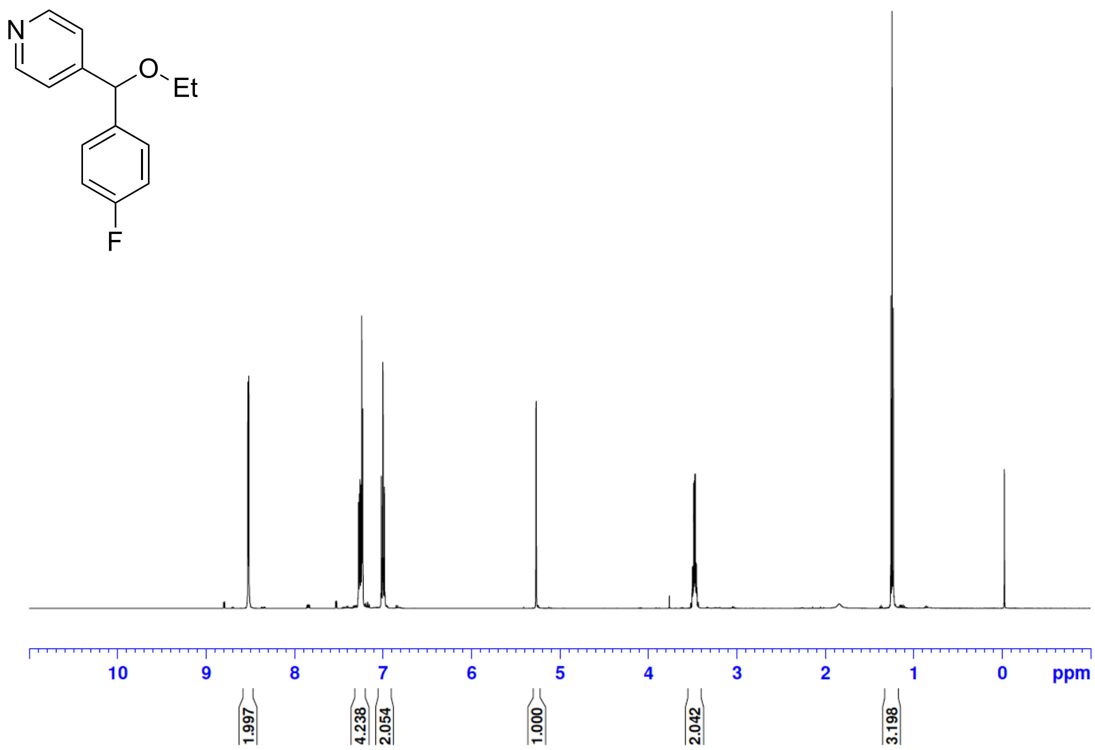

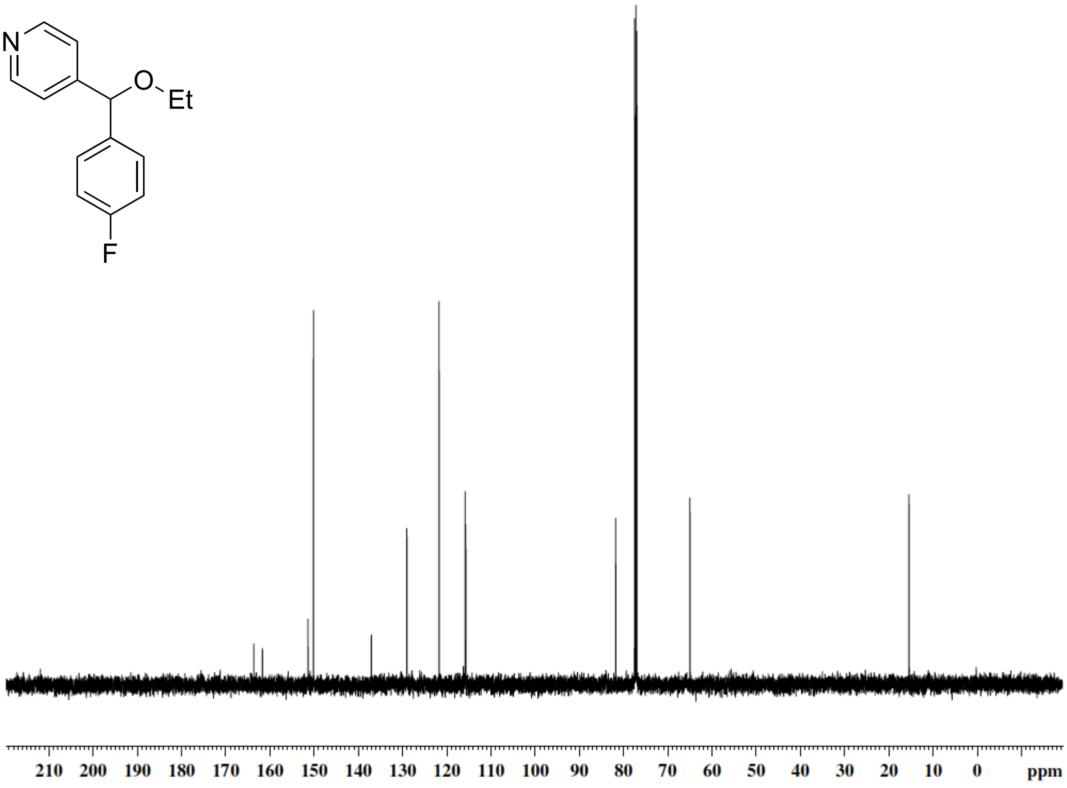

Figure S47. 500 MHz ^{1}H and 125 MHz ^{13}C{^{1}H} NMR spectra of **4je** in $CDCl_3$

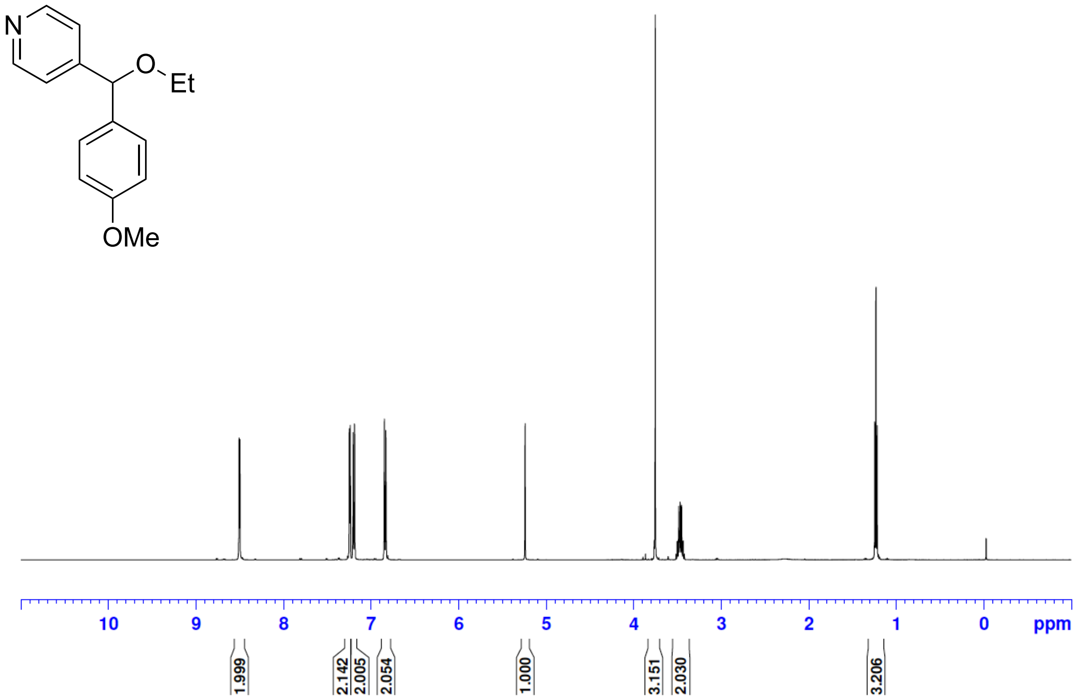

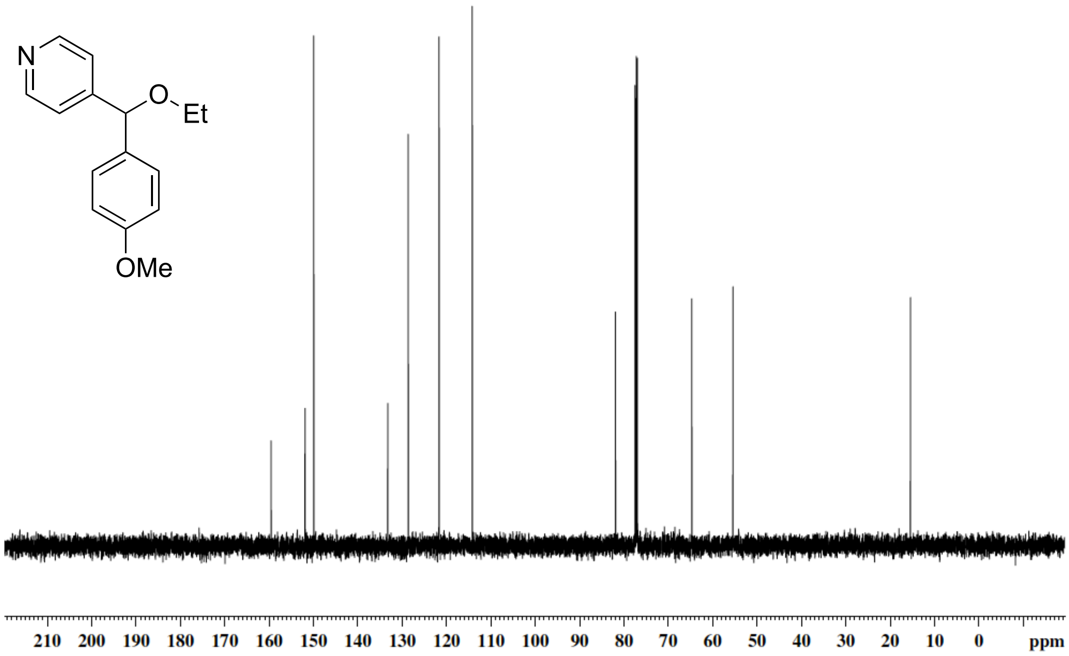

Figure S48. 500 MHz ^{1}H and 125 MHz ^{13}C{^{1}H} NMR spectra of **4jc** in $CDCl_3$

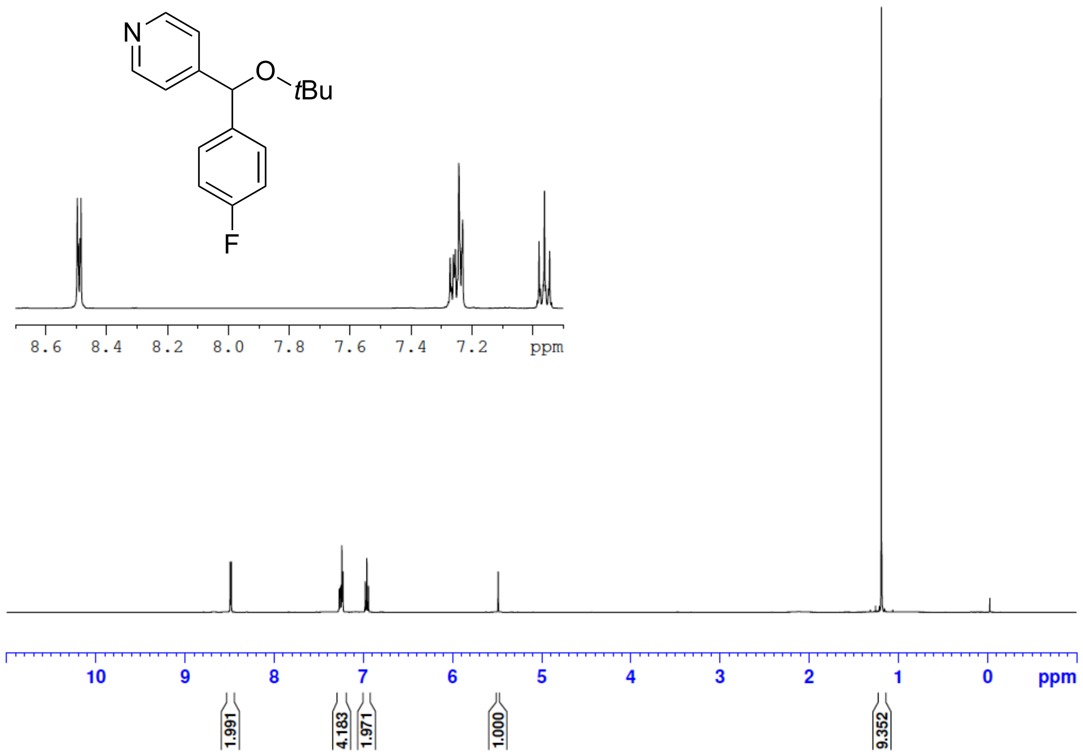

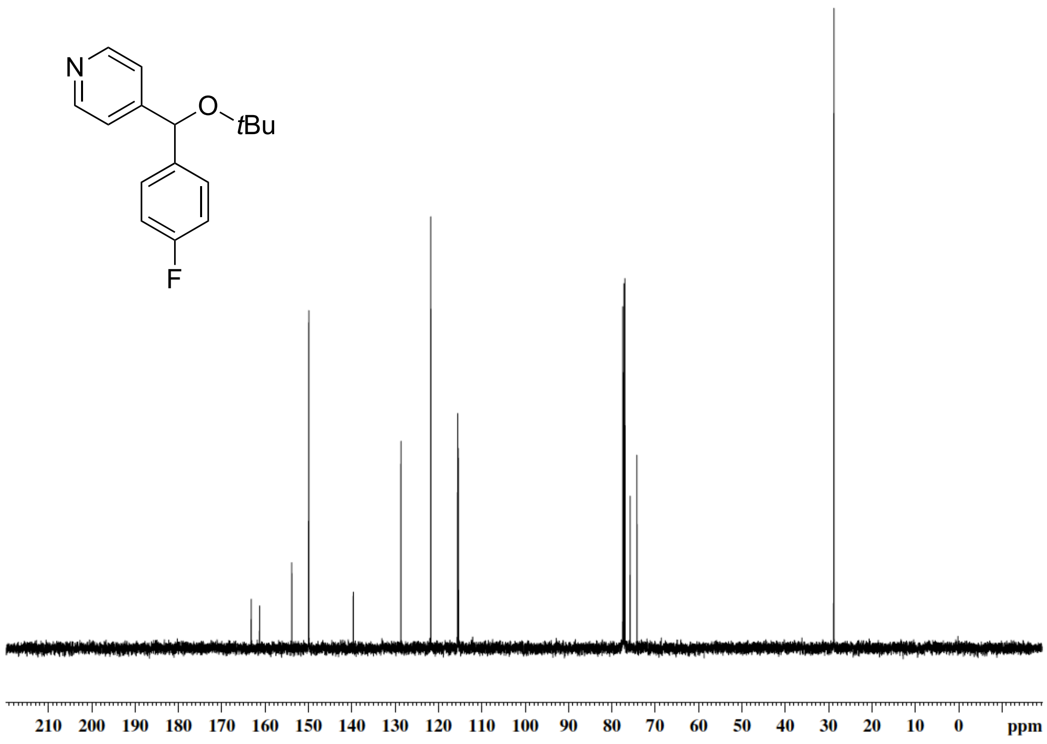

Figure S49. 500 MHz ^{1}H and 125 MHz ^{13}C{^{1}H} NMR spectra of **4ke** in $CDCl_3$

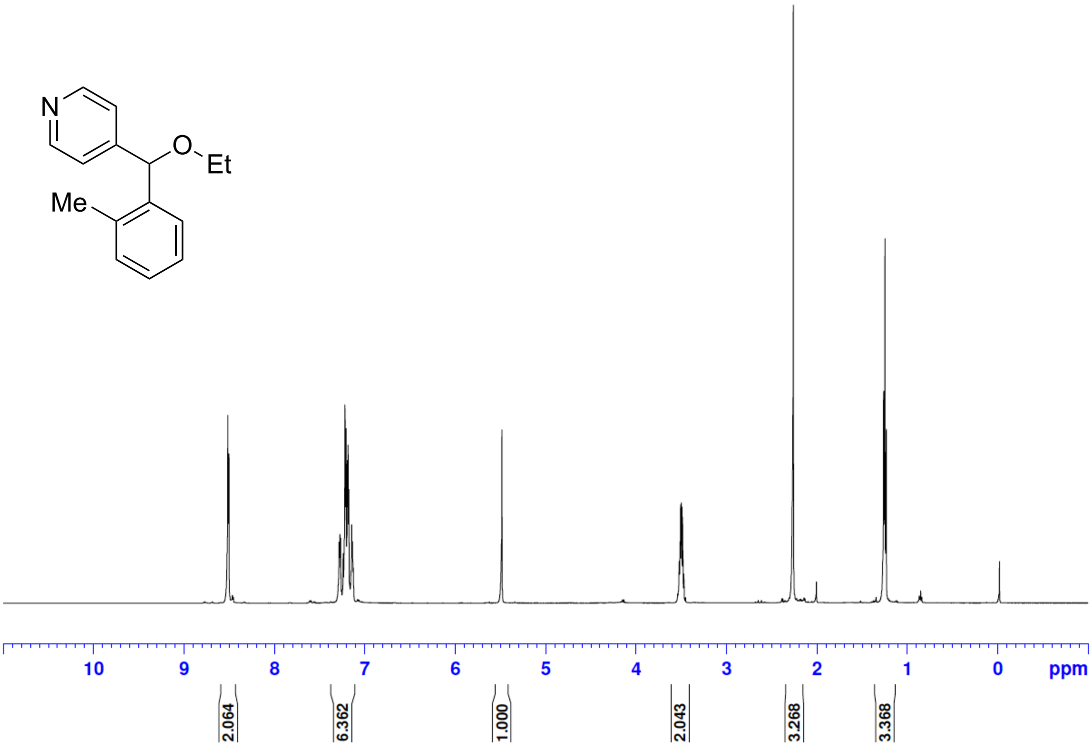

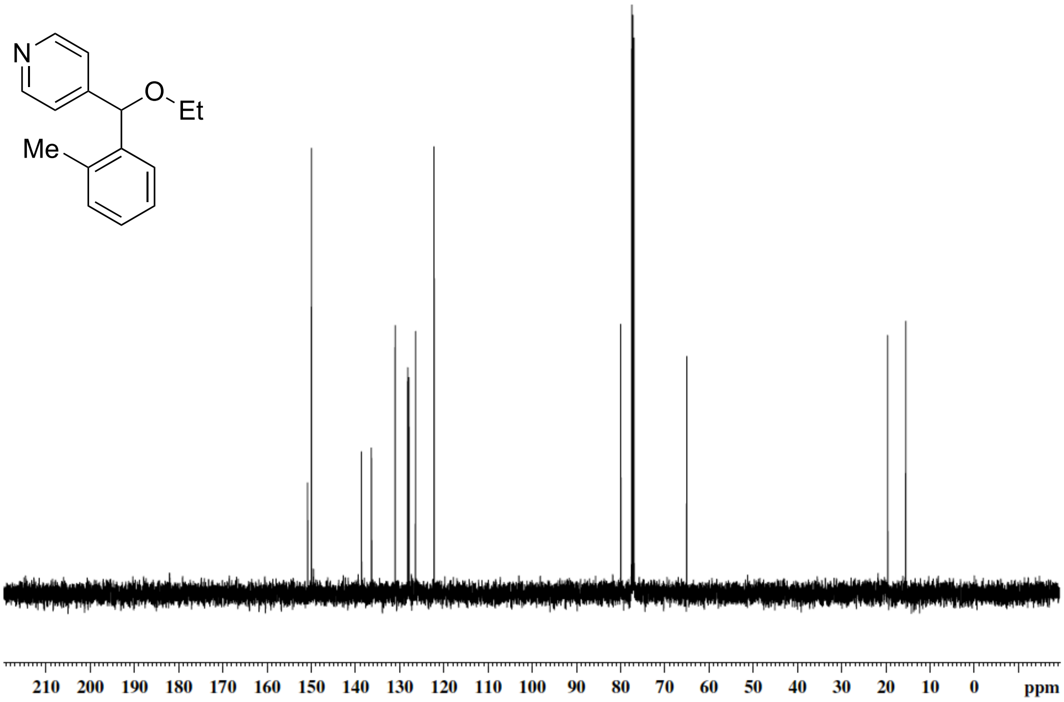

Figure S50. 500 MHz ^{1}H and 125 MHz $^{13}C\{^{1}H\}$ NMR spectra of **4jf** in $CDCl_3$
